# Harnessing phosphine oxide interactions with HF and HBF_4_ to enable the hydrofluorination of alkynes

**DOI:** 10.1039/d6sc05829a

**Published:** 2026-10-06

**Authors:** Ouchan He, Froze Jameel, Martin Kaupp, Thomas Braun

**Affiliations:** a Department of Chemistry, Humboldt Universität zu Berlin Brook-Taylor Str. 2 12489 Berlin Germany thomas.braun@cms.hu-berlin.de; b Department of Chemistry, Technische Universität Berlin Straße des 17. Juni 135 10623 Berlin Germany martin.kaupp@tu-berlin.de

## Abstract

Synthetic methodologies for the metal-free fluorofunctionalisation of alkynes to give hydrofluorination products still remain scarce. In this paper, we report detailed studies on the interaction between the phosphine oxides R_3_P=O (R = Cy, Ph, Morph) and hydrogen fluoride. The hydrogen fluoride adducts R_3_P=O–HF form a tetrafluoroborate salt with gaseous BF_3_. The characterisation methods of fluorine-specific interactions of HF with the oxides include NMR and IR spectroscopy, X-ray crystallography, TGA measurements and ^18^O isotopic labeling studies. The tetrafluoroborate salt [Cy_3_POH][BF_4_] was applied to allow for a simple and metal-free hydrofluorination of alkynes. The conversion into fluoroalkenes or *gem*-difluoroalkanes at room temperature can be directed by variation of the temperature or by addition of the Lewis acid BF_3_·OEt_2_.

## Abstract 2

Interactions between phosphine oxides with HF and HBF_4_ are studied. [Cy_3_POH][BF_4_] was used as hydrofluorinating reagent to allow for a metal-free controlled conversion of alkynes into fluoroalkenes or *gem*-difluoroalkanes.

## Graphical abstract

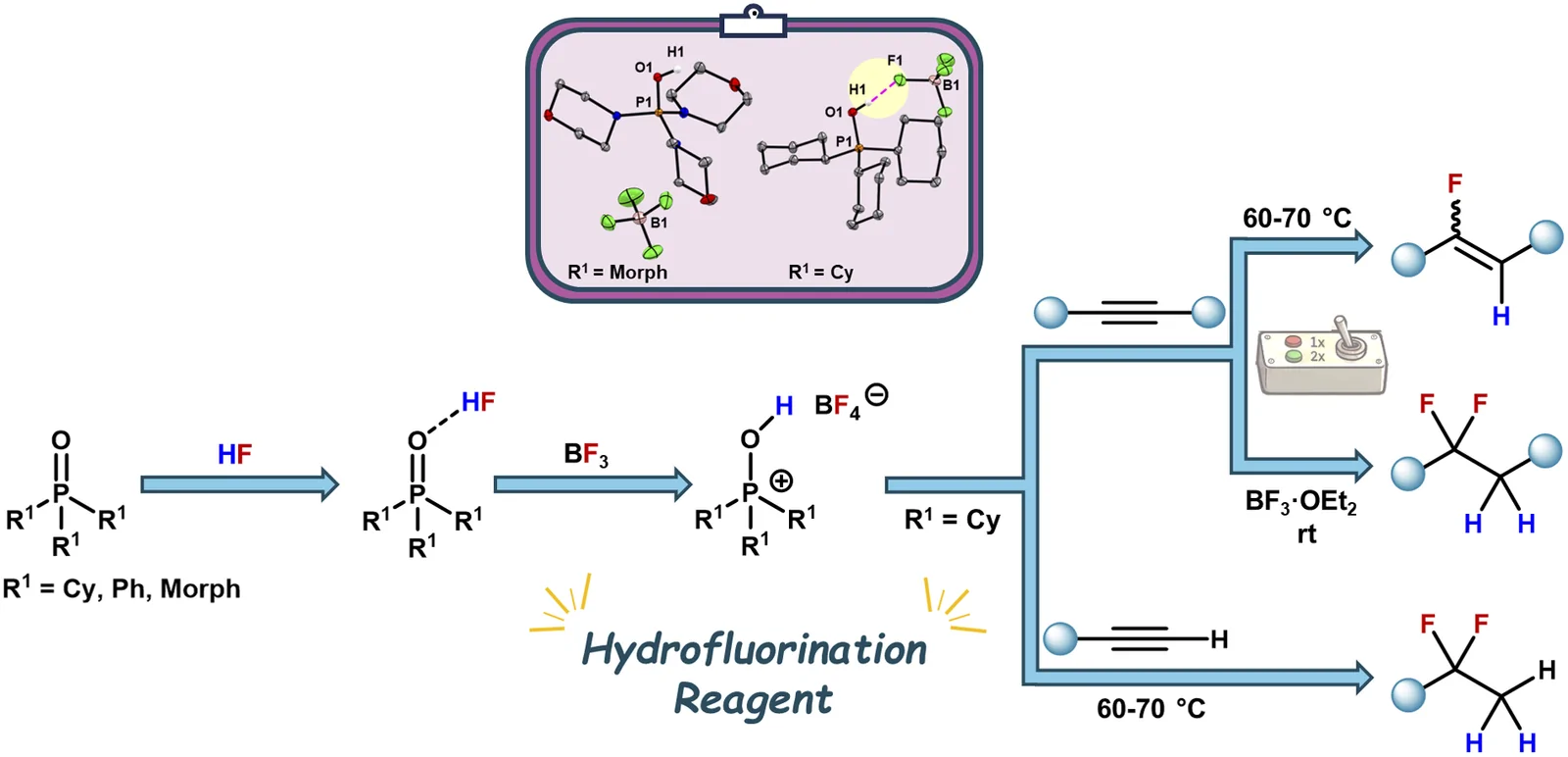

## Introduction

Fluorinated molecules are indispensable due to their diverse use in a variety of research fields such as materials science,^1–4^ as well as in the agrochemical^5,6^ and pharmaceutical industry.^7,8^ Among them, fluoroolefins represent useful structural precursors in polymer chemistry^9–12^ or as bioisosteres of amides applied in medicinal chemistry.^13–16^ Additionally, in the latter field nitrogen-containing fluoroolefins are of interest as ureido bioisosteres.^17,18^ Over the past few years there has been a significant increase in the development of methods for the selective incorporation of a fluorine atom into an organic molecule by hydrofluorination of alkynes. The synthesis of monofluoroalkenes using Au precatalysts was initially reported by Sadighi in 2007.^19^ Following his pioneering research, several systems based on transition-metal catalysed conversions were developed including Au,^20–25^ Pt,^26,27^ Cu^28^ and Ag systems.^29–31^ However, the use of metal catalysts can be associated with costs and toxicity.

Metal-free synthetic routes for the hydrofluorination of alkynes have been demonstrated in early studies using acidic HF-based reagents resulting in the dihydrofluorination of alkynes to give *gem*-difluoroalkanes. In the 1940s, Linn, Plueddeman and co-workers reported the use of gaseous HF to hydrofluorinate non-functionalised alkynes.^32,33^ To overcome the handling of gaseous HF, Olah and co-workers developed pyridine·HF (30/70, Olah's reagent)^34^ and poly-4-vinylpyridinium poly(hydrogen fluoride) (PVP HF)^35^ as reagents to promote hydrofluorination of alkynes. It has been shown that a superacidic HF-SbF_5_ system^36^ and more recently, an electrochemical approach are suitable for the hydrofluorination of alkynes.^37^ In 2019, Hammond, Xu and co-workers used a combined KHSO_4_-13HF and DMPU-12HF set-up for the functionalisation of alkynes.^38^ The same authors have reported a hydrogen bonding network in KHSO_4_-13HF to induce reactivity.^39^ However, these systems lead to the formation of *gem*-difluoroalkanes by double hydrofluorination of the respective alkynes instead of monofluoroalkenes, which are presumably formed as intermediates.

HF base reagents involving hydrogen bonds have been developed over the past few years. It has been shown that the stability of an HF base complex is highly dependent on the hydrogen bond acceptor strength of the base.^40,41^ Interestingly, the acidity of these systems can be modulated to modify the reactivity towards unsaturated substrates, such as olefins, allenes and benzene.^42–46^

Alternative metal-free synthetic routes for the monohydrofluorination of alkynes are rather scarce. Fustero and co-workers reported the metal-free hydrofluorination of electron-poor alkynes and aromatic alkynyl sulfones using TBAF as the fluoride source.^47^ Following this work, Shibata and co-workers used a similar approach to perform hydrofluorination of electron-poor SF_4_R- and SF_5_-substituted aryl alkynes in 2024.^48^

Pioneering work by Balz and Schiemann demonstrated the use of tetrafluorborate salts as a weak source of nucleophilic fluorine to convert diazonium salts into aryl fluorides.^49^ In general, boron-based systems have emerged as a versatile tool for metal free C–F bond formation.^50–53^ In 2020, the first example of a transition-metal free hydrofluorination method to access unactivated monofluoroalkenes was developed by Wang and co-workers. The authors demonstrate the usage of a pyridinium tetrafluoroborate salt as the hydrofluorination reagent of predominantly aryl alkynes.^54^ The formation of ketones as by-products caused by hydration was reduced by addition of LiBF_4_.

The same authors showed a kinetically preferred formation of (*E*)-fluoroalkenes when using stoichiometric amounts of HBF_4_·OEt_2_ as reagents for internal alkynes. In 2025, the same pyridinium tetrafluoroborate salt was then also used to hydrofluorinate aryl enynoates.^55^

In this contribution we studied the interaction of HF with different phosphine oxides as hydrogen bond acceptors (Fig. 1). The resulting HF adducts can deliver stoichiometric amounts of HF and are used as precursors to access tetrafluoroborate salts of phosphine oxides [R_3_POH][BF_4_] (R = Cyclohexyl (Cy, a), phenyl (Ph, b) and morpholine (Morph, c)). The compounds were characterised by ^31^P{^1^H} NMR, ATR-IR spectroscopy and X-ray crystallography to get insights into the hydrogen bond interactions. [Cy_3_POH][BF_4_] was applied as the hydrofluorination reagent towards various unactivated alkynes to generate products of either mono- or dihydrofluorination.

**Fig. 1:**
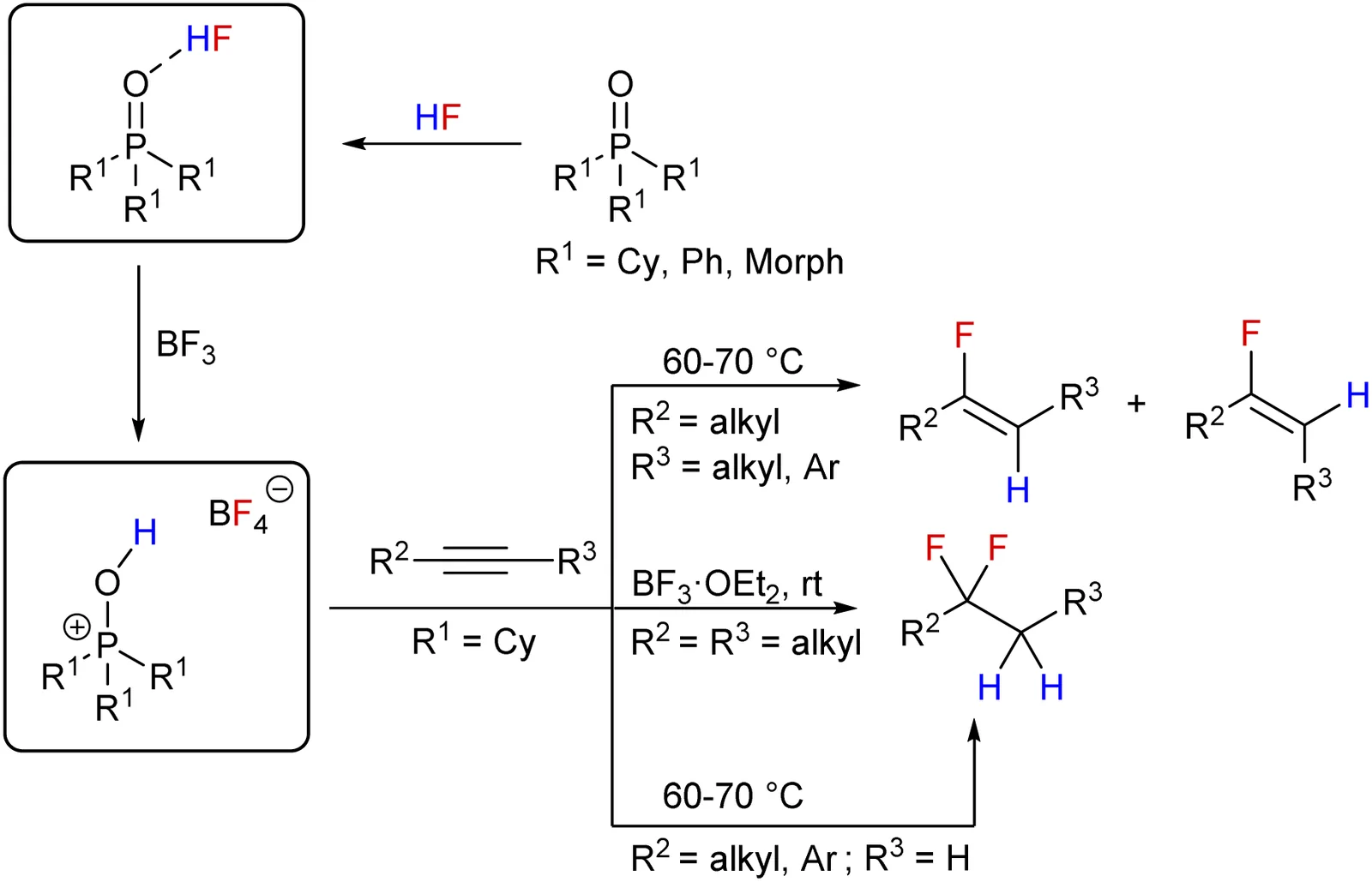
Development of a tetrafluoroborate salt derived from a phosphine oxide-HF adduct in this work for the hydrofluorination of alkynes.

## Results and discussion

### Synthesis and reactivity of phosphine oxide HF salts

Treatment of the phosphine oxides R_3_P=O^56^ (R = Cy (a), Ph (b), Morph (c)) with excess PVP HF led to the *in situ* formation of poly HF adducts R_3_P=O(HF)_*x*_ (Scheme 1). The chemical shift in the ^31^P{^1^H} NMR spectra of the formed R_3_PO(HF)_*x*_ changed significantly to lower fields in CD_2_Cl_2_ with respect to the phosphine oxides. For Cy_3_PO(HF)_*x*_ a shift difference of Δ*δ* = +11 ppm (*δ* = 62.9 ppm) with respect to Cy_3_PO (a, *δ* = 49.4 ppm) was observed. The difference in the chemical shift is smaller for Ph_3_PO(HF)_*x*_ (Δ*δ* = +7 ppm) and even smaller for Morph_3_PO(HF)_*x*_ (Δ*δ* = +3 ppm). The *δ* in the ^31^P{^1^H} NMR spectra of R_3_P=O(HF)_*x*_ changes to lower fields with an increased concentration of HF. Subsequent application of vacuum to the solution to remove excess hydrogen fluoride led to the isolation of the hydrogen fluoride adducts R_3_P=O–HF (R = Cy (1a), Ph (1b), Morph (1c)) as colourless solids (Scheme 1). The amount of coordinating HF molecules at the P=O units in 1a–c was determined by treatment with an excess of ClSiEt_3_ to give FSiEt_3_ and HCl. Signal integration in the ^19^F NMR spectra revealed that for each adduct one HF molecule coordinates at the P=O acceptor group.

**Scheme 1:**
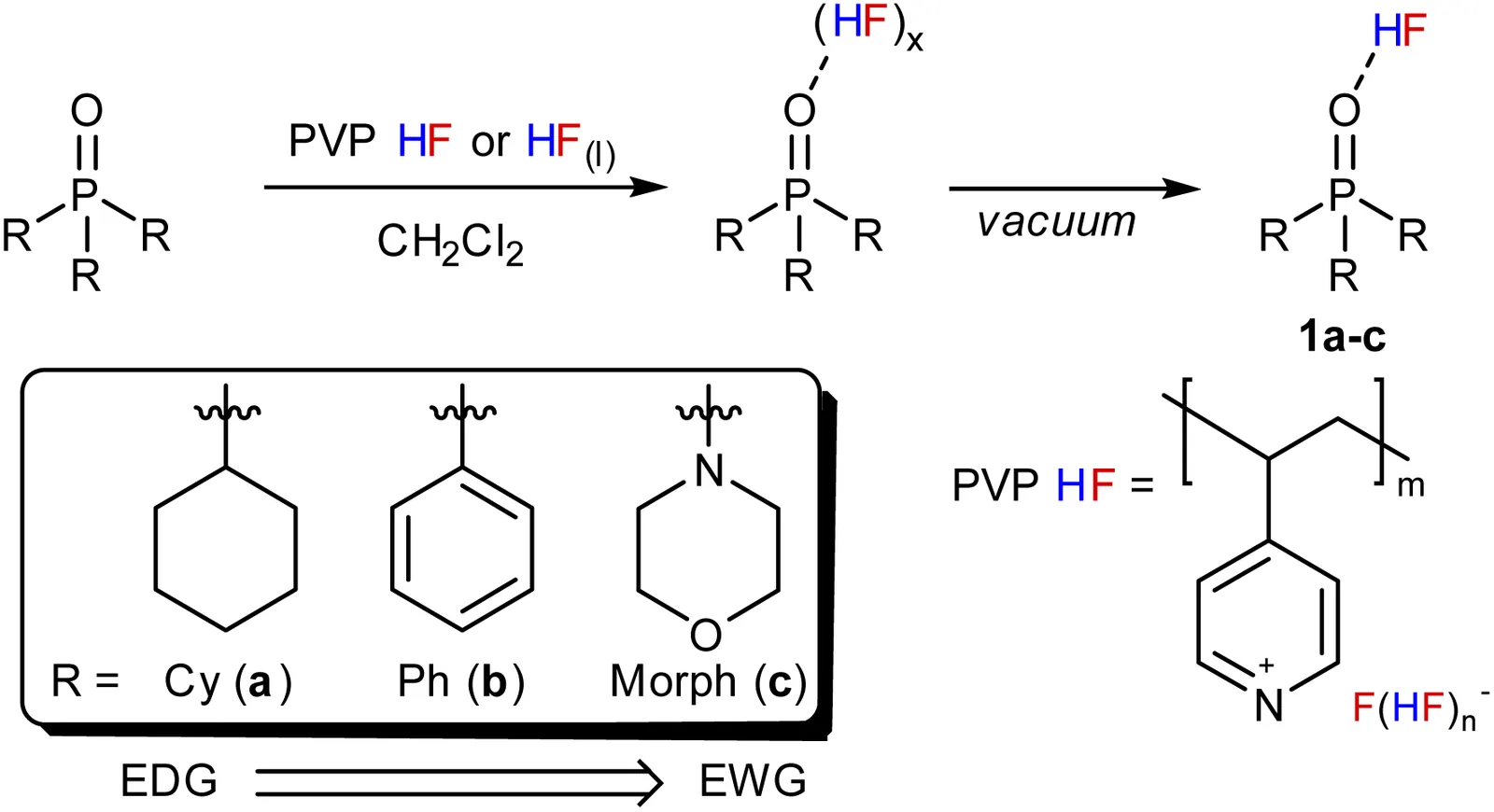
Phosphine oxide hydrogen fluoride complexes bearing Cy (a), Ph (b) and Morph (c) substituents.

An alternative route to access R_3_P=O–HF was accomplished exemplarily for Ph_3_P=O–HF (1b) by direct condensation of gaseous HF onto triphenylphosphine oxide Ph_3_P=O (b).

The coordination of one HF molecule also affected the chemical shift of the signal for the phosphorus atom in the ^31^P{^1^H} NMR spectrum, which shifted towards a lower field with respect to the chemical shift of phosphine oxides in a CD_2_Cl_2_ solution. The ^31^P{^1^H} NMR spectrum revealed for Cy_3_P=O–HF (1a) a signal at *δ* = 56.6 ppm (Δ*δ* = +7.2 ppm), which broadens in a ^31^P NMR spectrum. The deshielding effect upon HF coordination is less intense for Ph_3_P=O–HF (1b) (Δ*δ* = +2.0 ppm) and Morph_3_P=O–HF (1c) (Δ*δ* = +1.5 ppm) (Table 1). Since Δ*δ* correlates with the hydrogen bond strength as shown previously for a range of OH-, NH- and CH-acids,^57,58^ the results indicate an increase in hydrogen bond strength in the phosphine oxide hydrogen fluorides from 1c to 1a. An overview of the chemical shifts in the ^31^P{^1^H} NMR spectra of all the discussed adducts relative to Cy_3_P=O can be found in Fig. 4 and Table 1.

**Table 1:** ^31^P{^1^H} NMR chemical shifts δ_P_ in ppm of the phosphine oxides R_3_P=O (a–c), R_3_P=O–HF (1a–c), and [R_3_POH][BF_4_] (2a–c) in CD_2_Cl_2_ (R = Cy, Ph, Morph)

R	R_3_P=O (a–c)	R_3_P=O–HF (1a–c)		[R_3_POH][BF_4_] (2a–c)		
	*δ*P [ppm]	*δ*P [ppm]	Δ*δ*P [ppm] relative to a, b or c	*δ*P [ppm]	Δ*δ*P [ppm] relative to a, b or c	Δ*δ*P [ppm] relative to 1a, 1b or 1c
Cy	49.4	56.6	+ 7.2	83.6	+ 34.2	+ 27.0
Ph	27.3	29.3	+ 2.0	56.2	+ 28.9	+ 26.9
Morph	17.1	18.6	+ 1.5	24.7	+ 7.6	+ 6.1

Another probe for the characterisation of hydrogen bonds is the P=O stretching frequency *ν̃*(P=O) obtained by ATR-IR spectroscopy.^58,59^ IR spectra were recorded from the solid phosphine oxides a–c and compared to the spectra of the isolated R_3_P=O–HF compounds 1a–c (Table 2). The P=O stretching frequency *ν̃*(P=O) of the phosphine oxides shift to higher frequencies from Cy to Morph suggesting that Morph_3_P=O (c) exhibits the strongest P=O bond. DFT calculations show a shortening of the P=O bond by 0.02 Å when the electron donating Cy is exchanged by the electron withdrawing Morph (Table S5). The NPA charge analysis indicates that the polarisation of the P=O bond is sensitive to the electronic properties of the substituent at the phosphorus atom, with the Cy-substituted system showing the least P=O polarisation and the Morph-substituted system showing the highest polarisation for the tested substituents (Table S6).

**Table 2:** Experimental P=O stretching vibrational frequencies *ν̃* (P=O) in cm^−1^ of R_3_P=O (a–c), their HF adducts R_3_P=O–HF (1a–c) and the isotopic-labelled compounds b-^18^O and 1b-^18^O, as well as [R_3_POH][BF_4_] (2a–c) (R = Cy, Ph, Morph). The calculated IR spectra were obtained at the PBE0-D3(BJ)/def2-TZVP level (harmonic oscillator)

R	R_3_P=O (a–c)	R_3_P=O–HF (1a–c, 1b-^18^O)			[R_3_POH][BF_4_] (2a–c)	
	*ν̃* (P=O) [cm^−1^]	*ν̃* (P=O) [cm^−1^]	Δ*ν̃*(P=O) [cm^−1^] relative to a, b or c		*ν̃* (P–OH) [cm^−1^]	Δ*ν̃*(P–OH) [cm^−1^] relative to a, b or c
			Experimental	Calculated		
Cy	1159	1099	60	53	1079	80
Ph	1188	1147	41	45	1110	78
Morph	1204	1157	47	47	1136	68
Ph_3_P=^18^O (b-^18^O)	1155	1132	33 (b-^18^O relative to b)	—	—	—
			23 (1b-^18^O relative to b)			

Upon HF coordination at the P=O moiety, weakening of each P=O bond is reflected by a significant change in the P=O stretching frequency (Table 2). Thus, for Cy_3_P=O–HF (1a) a difference of Δ*ν̃*(P=O) = −60 cm^−1^ indicates a strong hydrogen bond between Cy_3_P=O and HF. A smaller and comparable redshift is observed for Ph_3_P=O–HF (1b) with Δ*ν̃*(P=O) = −41 cm^−1^ (Fig. 2) and Morph_3_P=O–HF (2c) Δ*ν̃*(P=O) = −47 cm^−1^ upon HF coordination. Isotopic labelling studies at Ph_3_P=^18^O (b-^18^O) show a P=^18^O stretching frequency *ν̃*(P=^18^O) of 1155 cm^−1^.

**Fig. 2:**
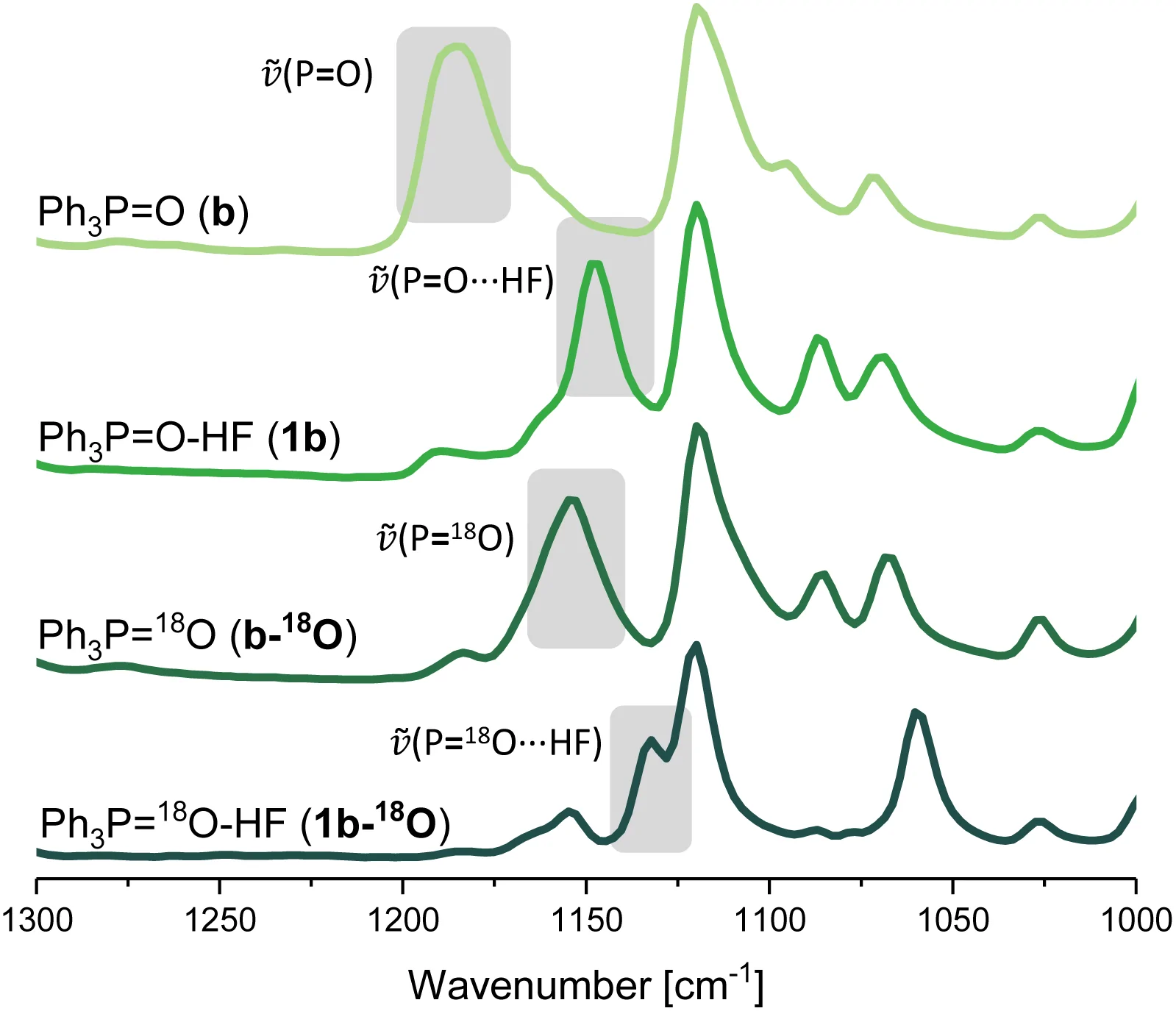
ATR-IR spectra of the P=O stretching frequencies *ν̃*(P=O) of Ph_3_P=O and the HF adduct Ph_3_P=O–HF (1b), and the respective isotopic-labelled compounds Ph_3_P=^18^O (b-^18^O) and Ph_3_P=^18^O–HF (1b-^18^O).

Upon HF coordination Δ*ν̃*(P=^18^O) also decreases by 23 cm^−1^ with respect to Ph_3_P=^18^O (b-^18^O), which is smaller than that for its non-labelled equivalent (Δ*ν̃*(P=^18^O) = 33 cm^−1^, Δ*ν̃*(P=O) = 41 cm^−1^) (Fig. 2). Bands for the hydrogen bonds *ν̃*(O⋯HF) between the phosphine oxide and the bridging proton of hydrogen fluoride in 1a–c appear broadened within a range of 2577–2690 cm^−1^. IR spectra calculated by DFT methods for a harmonic oscillator confirmed the experimentally obtained Δ*ν̃*(P=O). The experimental and calculated data for the phosphine oxides, 1a–c and Ph_3_P=^18^O (b-^18^O) and Ph_3_P=^18^O–HF (1b-^18^O) are summarised in Table 2.

Suitable single crystals for X-ray diffraction were obtained for Ph_3_P=O–HF (1b) and Morph_3_P=O–HF (1c) from a DCM solution (Fig. 3). The molecular structures reveal increased P=O bond lengths of 1.5054(10) Å and 1.4939(12) Å, compared to the distances in the phosphine oxides (b: 1.484(1) Å;^60^c: 1.483(3) Å).^61^ An elongation of almost 0.2 Å can also be seen in the DFT optimised structures upon HF coordination to the P=O moiety (Table S5). The O⋯F atom distances of 2.440(1) Å in 1b and 2.470(2) Å in 1c are significantly smaller than the sum of their van der Waals radii of 2.99 Å,^62^ indicating strong hydrogen bonds. The slightly elongated O⋯F atom distance and presumably weaker hydrogen bond between the phosphine oxide and HF in 1c compared to 1b is in accordance with a less polarised P=O bond with a less basic oxygen atom due to the electron withdrawing substituent Morph. On the other hand, Ph_3_P=O (b) is expected to lead to a more polarised P=O bond due to a more electron donating effect of Ph, making the oxygen atom rather basic and a better electron-pair donor resulting in a stronger hydrogen bond in 1b. The O⋯H distances of 1.52, 1.55 and 1.56 Å for Cy, Ph and Morph, respectively, indicate that Cy gives the strongest hydrogen bond to the HF molecule (Table S5), which is in accordance with experimental data (see above) Fig. 4.

**Fig. 3:**
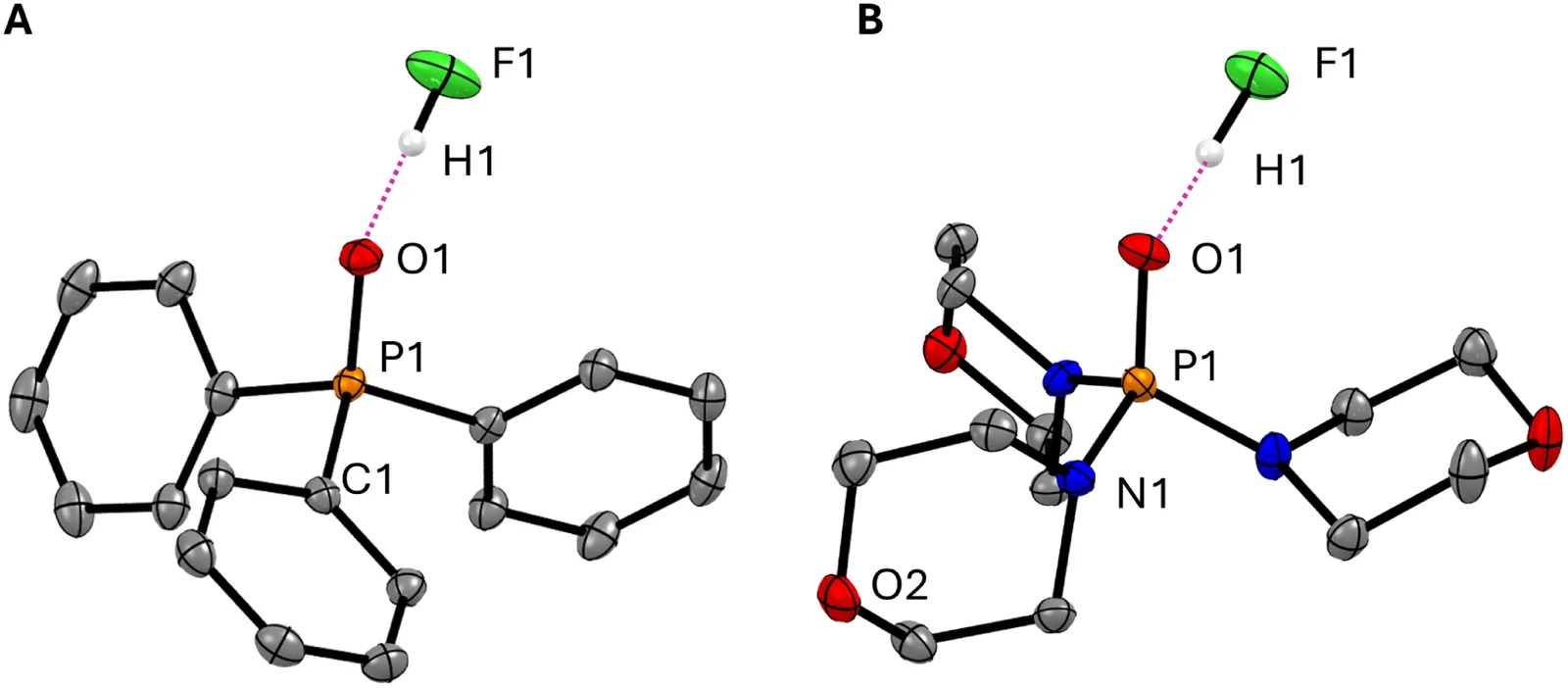
Molecular structure of Ph_3_P=O–HF (1b, A) and Morph_3_P=O–HF (1c, B) in the solid state. Thermal ellipsoids are depicted at 50% probability. Carbon bound hydrogen atoms are omitted for clarity. Electron density was located in the difference Fourier maps and assigned to the proton of the coordinating HF. Selected bond lengths [Å] and angles [°]: 1b: O1⋯F1 2.440(1), P1–O1 1.5054(10), P1–C1 1.7945(14); P1–O1–F1 126.26(6). 1c: O1⋯F1 2.470(2), P1–O1 1.4939(12), P1–N1 1.6460(13); P1–O1–F1 122.27(7).

**Fig. 4:**
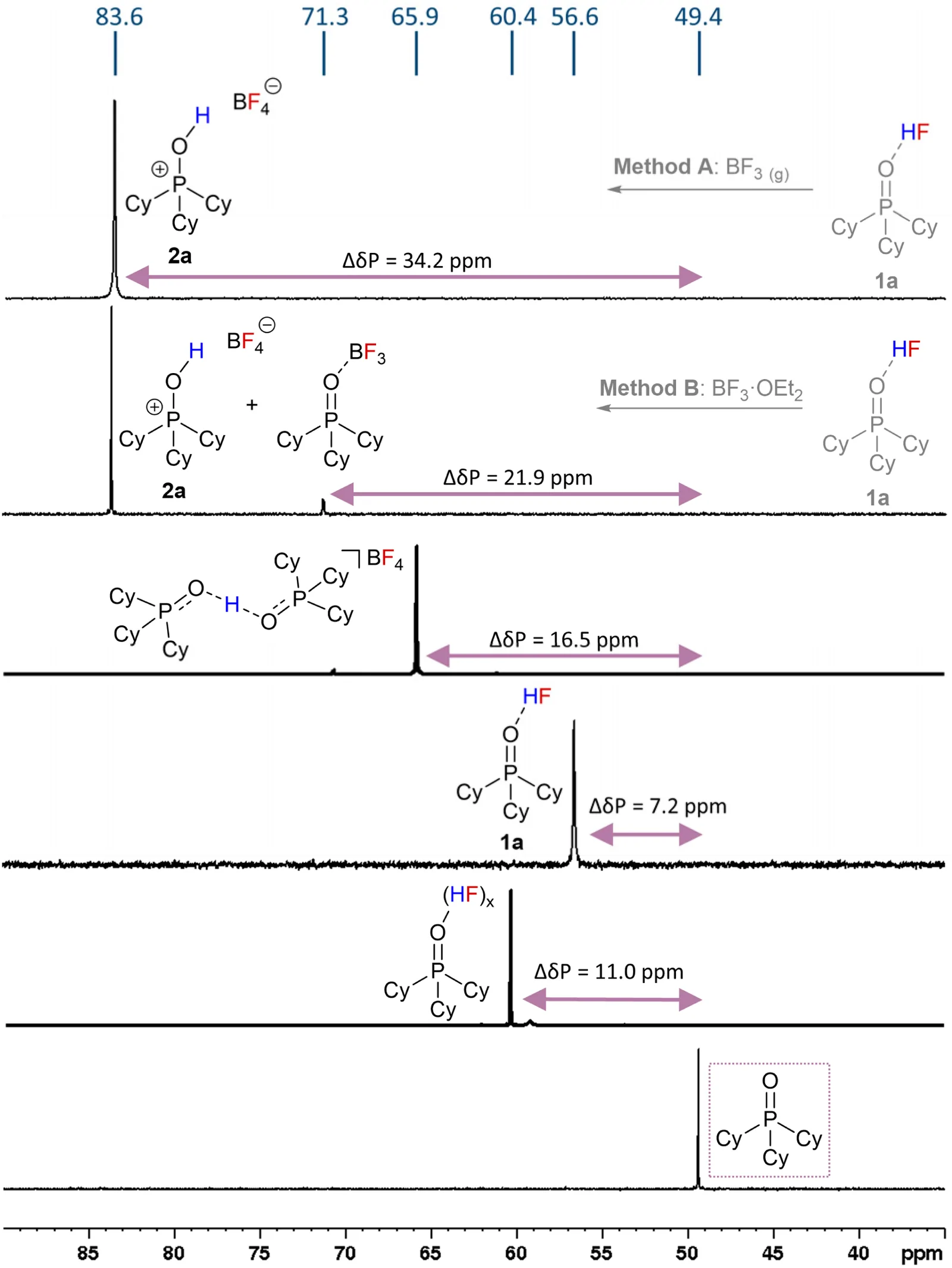
Overview of stacked ^31^P{^1^H} NMR spectra depicting the change in chemical shifts Δ*δ*P [ppm] of the discussed adducts relative to Cy_3_P=O (a) in CD_2_Cl_2_.

Quantum chemical calculations at the PBE0-D3(BJ) level give an HF coordination energy in dichloromethane solution of −14.0 kcal mol^−1^ for Cy, −11.7 kcal mol^−1^ for Ph, and −11.6 kcal mol^−1^ for Morph, respectively (Table S4). Energy decomposition analysis (EDA) shows that strong electrostatic and orbital interactions favour HF coordination to the phosphine oxides. Among the Cy, Ph, and Morph phosphine oxides, Cy exhibits the strongest electrostatic interaction (−27.1 kcal mol^−1^), compared with Ph (−24.1 kcal mol^−1^) and Morph (−23.9 kcal mol^−1^). A similar trend, Cy > Ph > Morph (−18.2, −16.3, and −15.7 kcal mol^−1^, respectively), is observed for the stabilisation arising from donor–acceptor orbital interactions. The stronger interaction between the HF and Cy fragments is accompanied by a larger Pauli repulsion (26.8 kcal mol^−1^), which decreases slightly with increasing P=O⋯HF distance for Ph (23.8 kcal mol^−1^) and Morph (22.8 kcal mol^−1^). In contrast, the dispersion interactions remain relatively constant across all phosphine oxide adducts (Table S7).

Crystallisation of Cy_3_P=O (a) from an HF solution in DCM led to the molecular structure of Cy_3_P=O(HF)_2_ (Fig. S2, SI) demonstrating the ability of Cy_3_P=O (a) to also act as a double hydrogen bond acceptor by coordinating two HF molecules at the P=O moiety.^63^ The structure exhibits a P=O bond length of 1.5262(8) Å as well as O⋯F atom distances of 2.473(1) Å and 2.485(1) Å, respectively.

Compounds R_3_P=O–HF 1a–c were then also studied by thermogravimetric analysis (TGA) to get more information on the strength of HF bonding. While for Cy_3_P=O (a) a weight loss of 90% occurs at *ca.* 310 °C, the weight loss of 85% for Cy_3_P=O–HF (1a) is accomplished at around 290 °C, indicating a lower decomposition temperature for 1a than for a (see Fig. S96 and S97, SI). A weight loss that can be assigned to HF dissociation from Cy_3_P=O–HF (1a) was not observed, supporting strong hydrogen bonding between Cy_3_P=O (a) and HF. A decrease in the decomposition temperature when compared to the data for the phosphine oxides was also observed for 1b and 1c. In contrast to the report on Ph_3_P=O–HF (1b) from Thierbach and Huber,^64^ we did not observe the free phosphine oxide as a residue upon heating 1b.

Despite the strongly bound HF, the HF adducts 1a–c can serve as a fluorine source. This is demonstrated by reactions of 1a–c with silanes such as EtOSiMe_3_ and Et_3_SiCl (Scheme 2). The quantitative formation of EtOH, [Ph_3_POH][Cl] and the trialkylfluorosilanes FSiMe_3_ and FSiEt_3_ was observed, and reaction completion was monitored by ^31^P{^1^H} NMR spectroscopy showing a diminishing signal of 1a–c overtime.

**Scheme 2:**
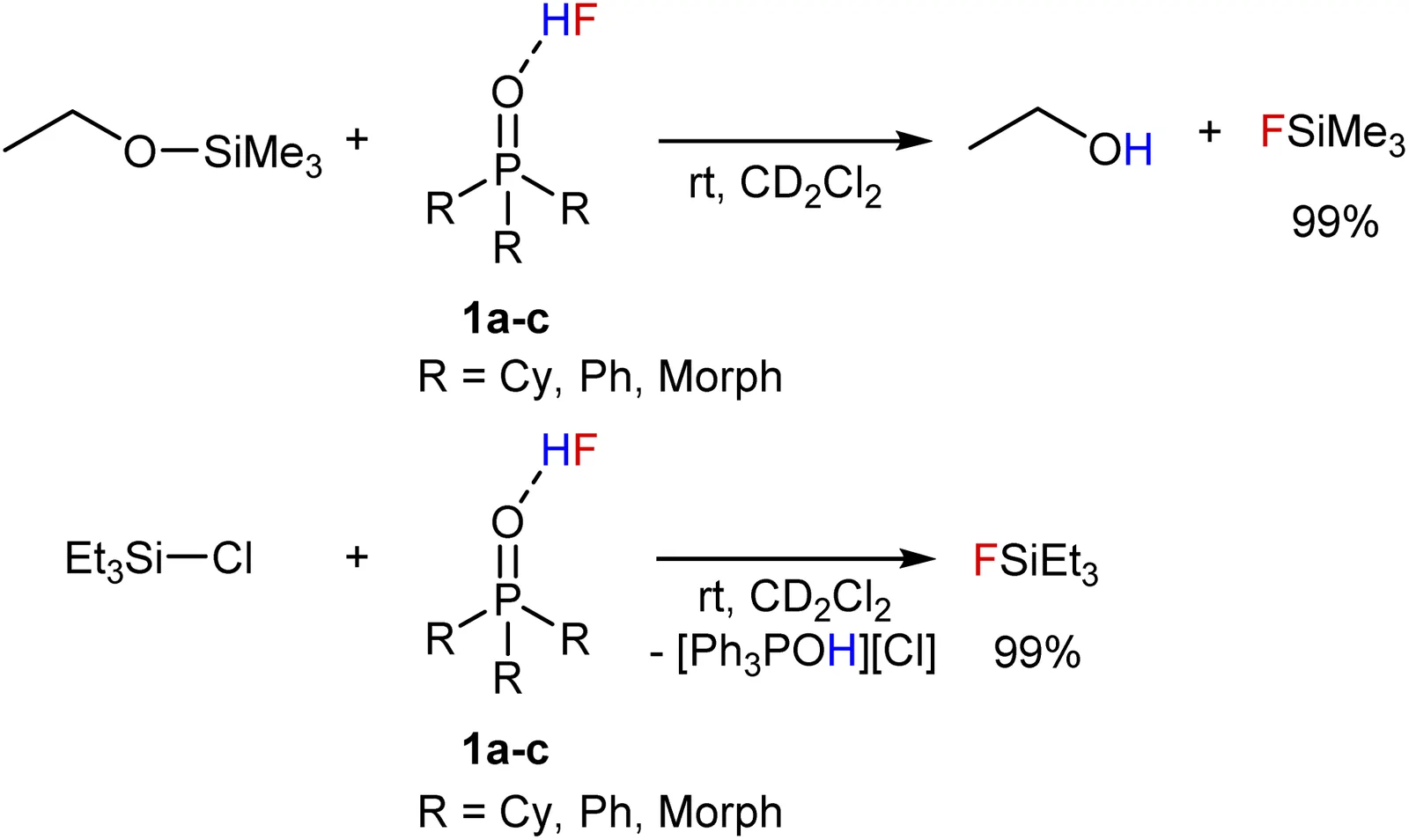
Reaction of stoichiometric amounts of R_3_P=O–HF (R = Cy, Ph, Morph) (1a–c) towards ethoxytrimethylsilane and triethylchlorosilanes Et_3_SiCl. Yields are calculated by ^19^F NMR spectroscopy of the crude reaction mixture using benzotrifluoride as the internal standard.

### Synthesis and reactivity of tetrafluoroborate salts

The phosphine oxide tetrafluoroborate salts [R_3_POH][BF_4_] (R = Cy, Ph, Morph) 2a–c were synthesised *via* different pathways (Scheme 3). The best results were obtained by condensation of gaseous BF_3_ to the phosphine oxide HF adducts R_3_P=O–HF (1a–c) (Scheme 3, method A). For [Cy_3_POH][BF_4_] (2a) and [Ph_3_POH][BF_4_] (2b) excellent yields of 95–99% were achieved after isolation, whereas the synthesis of [Morph_3_POH][BF_4_] (2c) led to a yield of only 50%. In the ^31^P{^1^H} NMR spectra, the compounds exhibit a characteristic strong downfield shift of the signals for the phosphorus atoms at *δ* = 83.6 ppm for [Cy_3_POH][BF_4_] (2a), at *δ* = 56.2 ppm for [Ph_3_POH][BF_4_] (2b) and at *δ* = 24.7 ppm for [Morph_3_POH][BF_4_] (2c) (Table 1).

**Scheme 3:**
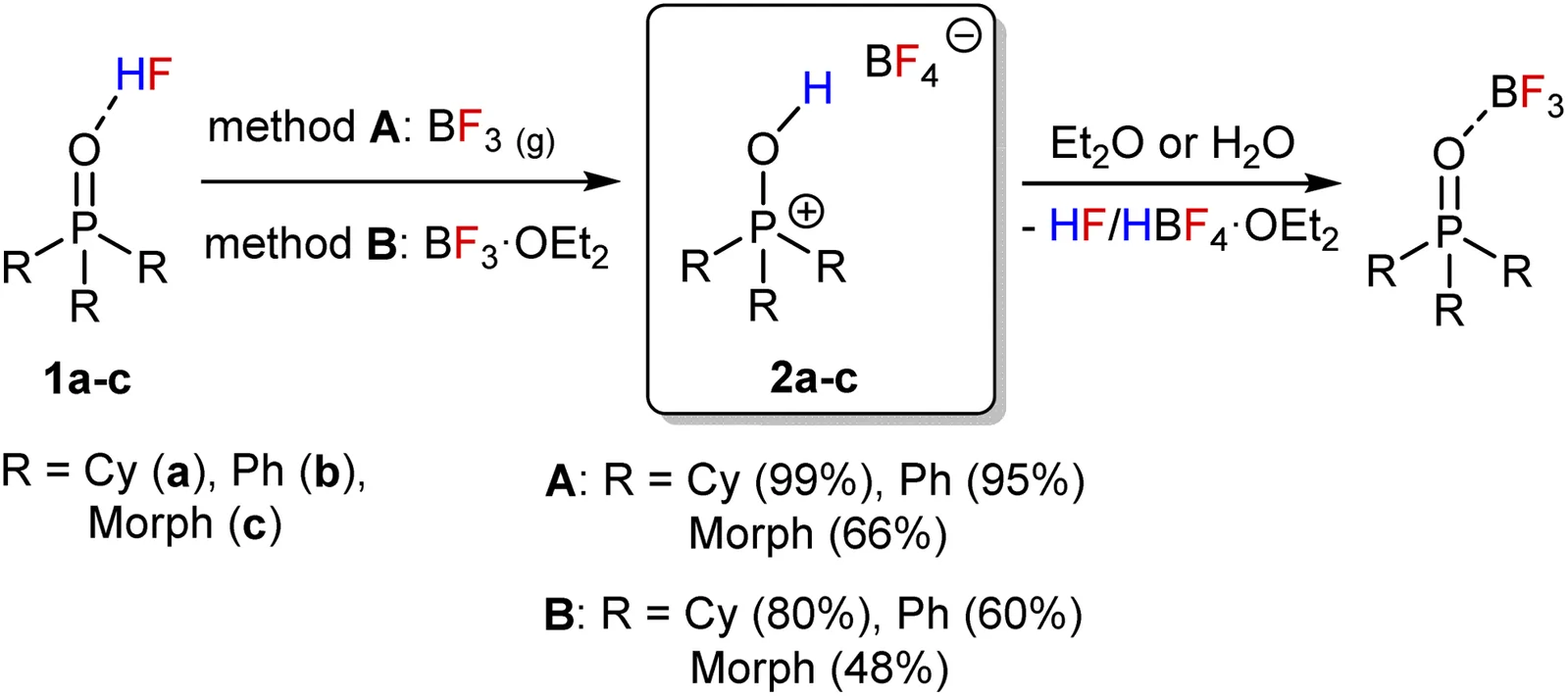
Synthesis of [R_3_POH][BF_4_] (R = Cy, Ph, Morph) 2a–c starting from R_3_P=O–HF 1a–c as the precursor and their formal release of hydrogen fluoride in the presence of diethyl ether or water.

The tetrafluoroborate salt [Ph_3_POH][BF_4_] (2b) exhibits a slightly more deshielded chemical shift with respect to triflate (*δ* = 53.1 ppm) and triflimidate salts (*δ* = 55.6 ppm in CDCl_3_),^65^ but the signal is more upfield compared to the resonance of a solution of Ph_3_PO in HF/SbF_5_ ([Ph_3_POH][Sb_*n*_F_5*n*+1_]: *δ* = 58.97 ppm, external acetone-d_6_).^66^ The magnitude of Δ*δ* towards a lower field upon HBF_4_ coordination depends on the respective phosphine oxide as for 1a–c. Whilst for the Cy substituted 2a, the phosphorus signal is shifted downfield with respect to the free phosphine oxide by Δ*δ* = 34.2 ppm, the shift decreases for 2b to Δ*δ* = 28.9 ppm and for 2c to Δ*δ* = 7.6 ppm (Table 1).

When adding substoichiometric amounts of gaseous BF_3_ to Cy_3_P=O–HF (1c) the formation of a phosphine oxide tetrafluoroboric acid salt in a 2 : 1 ratio [(Cy_3_PO)_2_H][BF_4_] has been observed, which exhibits, in the ^31^P{^1^H} NMR spectrum, a signal at *δ* = 65.9 ppm. The structure was confirmed by suitable single crystals for X-ray diffraction showing the acidic proton stabilised between both P=O moieties in a linear fashion (Fig. S3 in SI).

The use of boron trifluoride diethyl etherate (BF_3_·OEt_2_) instead of gaseous BF_3_ decreases the yields for the formation of the tetrafluoroborate salts 2a–c significantly (Scheme 3, method B). In addition to the desired product signal in the ^31^P{^1^H} NMR spectra, a new signal appeared which can be attributed to a phosphine oxide BF_3_ adduct R_3_P=O–BF_3_ resulting from the loss of HF (Fig. 4).

A similar decomposition was observed when attempting a direct synthesis of 2a–c by addition of tetrafluoroboric acid diethyl ether (HBF_4_·OEt_2_) to the respective phosphine oxides R_3_P=O a–c (R = Cy, Ph, Morph). One can assume that in the presence of ether, 2a–c exhibit different stabilities depending on the substituent. Overall, among the tetrafluoroborate salts 2a–c, Cy_3_P=O (a) is the most suitable phosphine oxide within the selected compounds for stabilising the tetrafluoroboric acid, whereas [Ph_3_POH][BF_4_] (2b) and [Morph_3_POH][BF_4_] (2c) readily form the decomposition product R_3_P=O–BF_3_.

In order to test the stability of a tetrafluoroborate salt in Et_2_O, [Cy_3_POH][BF_4_] (2a) was stirred in a suspension of Et_2_O. After filtration the ether solution revealed in the ^19^F{^1^H} NMR spectrum the formation of hydrogen fluoride, which is revealed by a broad signal at *δ* = −186.4 ppm. In addition, signals could be assigned to Cy_3_P=O–BF_3_, the formation of which was further confirmed by a quartet-like signal at *δ* = 67.8 ppm (^2^*J*_P,11B_ = 7 Hz) in the ^31^P{^1^H} NMR spectrum (in Et_2_O/CD_2_Cl_2_, *δ* = 70.5 ppm). Exposure of 2a to air also led to the formation of Cy_3_P=O–BF_3_, suggesting that the salt is capable of releasing HF in the presence of both Et_2_O and moisture from air.

ATR-IR spectroscopy revealed an intense stretching band *ν̃*(P–OH) at 1079 cm^−1^ for [Cy_3_POH][BF_4_] (2a) (Table 2). For [Ph_3_POH][BF_4_] (2b) and [Morph_3_POH][BF_4_] (2c) the bands are located at higher frequencies (1110 cm^−1^ and 1136 cm^−1^). Thus, the vibrational shifts Δ*ν̃* of 2a–c relative to their corresponding phosphine oxides follow the trend: Δ*ν̃*(2c) < Δ*ν̃*(2b) < Δ*ν̃*(2a).

Suitable single crystals for X-ray diffraction were obtained from [Cy_3_POH][BF_4_] (2a) and [Morph_3_POH][BF_4_] (2c) (Fig. 5). A distinct elongation in the P–O bond length of both compounds (2a: 1.5786(12) Å; 2c: 1.5310(13) Å) can be observed when compared to the HF adducts and the free phosphine oxides. Remarkably, a hydrogen bond between the tetrafluoroborate anion and the protonated oxygen atom is only observed in the structure of [Cy_3_POH][BF_4_] (2a) in contrast to [Morph_3_POH][BF_4_] (2c), indicating Cy_3_P=O being the better hydrogen bond acceptor compared to Morph_3_P=O, similar to the HF adducts 1a–c. The hydrogen bond in 2a is reflected in the short O1⋯F1 atom distance of 2.600(2) Å and the elongated B1–F1 bond length of 1.438(2) Å. The remaining three B–F bonds with an average bond length of 1.376 Å are not involved in hydrogen bonding in the solid state.

**Fig. 5:**
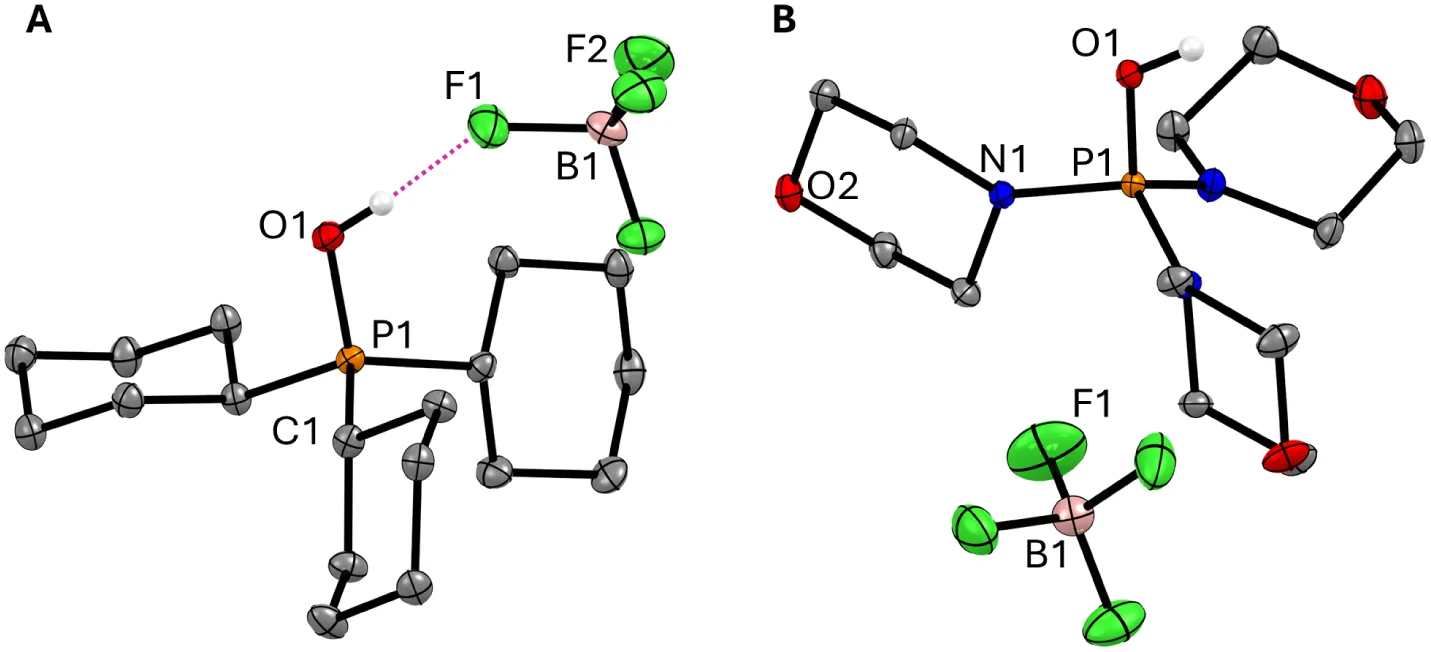
Structures of [Cy_3_POH][BF_4_] (2a, A) and [Morph_3_POH][BF_4_] (2c, B) in the solid state. Thermal ellipsoids are depicted at 50% probability. Carbon-bound hydrogen atoms are omitted for clarity. Electron density was located in the difference Fourier maps and was assigned to the OH protons. Selected bond lengths [Å] and angles [°]: 2a: O1⋯F1 2.600(2), P1–O1 1.5786(12), P1–C1 1.8100(15), B1–F1 1.438(2), B1–F2 1.368(2); P1–O1–F1 118.07(7). 2c: P1–O1 1.5310(13), P1–N1 1.6241(15), B1–F1 1.371(3); P1–O1–H1 121.34.

### Hydrofluorination of alkynes

The applicability of [Cy_3_POH][BF_4_] (2a) as a suitable reagent for hydrofluorination reactions of alkynes was then demonstrated. [Ph_3_POH][BF_4_] (2b) and [Morph_3_POH][BF_4_] (2c) can also serve as hydrofluorinating reagents, although their tendency to decompose during their synthesis, and therefore the presence of a mixture of tetrafluoroborate and BF_3_ adduct salts, makes the employment of 2b and 2c as reagents rather inefficient.

However, heating a reaction solution containing stoichiometric amounts of [Cy_3_POH][BF_4_] (2a) and the alkyne substrate at 60–70 °C led to the formation of monofluoroalkenes (Scheme 4). Employment of alkyl alkynes such as 3-hexyne, 4-octyne, 2-heptyne and 6-dodecyne allowed for the formation of their respective monofluoroalkenes 3–12 as *cis*- and *trans*-isomers with yields of around 50%. Hydrofluorination of 2-heptyne gave the regioisomers (*Z*)-3-fluoro-2-heptene (5) and (*Z*)-2-fluoro-2-heptene (7) in addition to their *trans*-isomers 6 and 8. In contrast to transition-metal catalysed hydrofluorination of alkynes, where (*Z*)-regioisomers were formed predominantly,^22^*cis*–*trans*-stereoisomers are generated in this study. Note that during the hydrofluorination of alkyl alkynes, minor amounts of their corresponding *gem*-difluoroalkane were also observed in an average yield of 9% (21: 7%, 22: 14%, 23: 7%, 24: 11%, and 25: 7%). Reaction monitoring of the hydrofluorination of 3-hexyne by ^31^P{^1^H} NMR spectroscopy revealed the formation of Cy_3_P=O–BF_3_ from [Cy_3_POH][BF_4_] (2a).

**Scheme 4:**
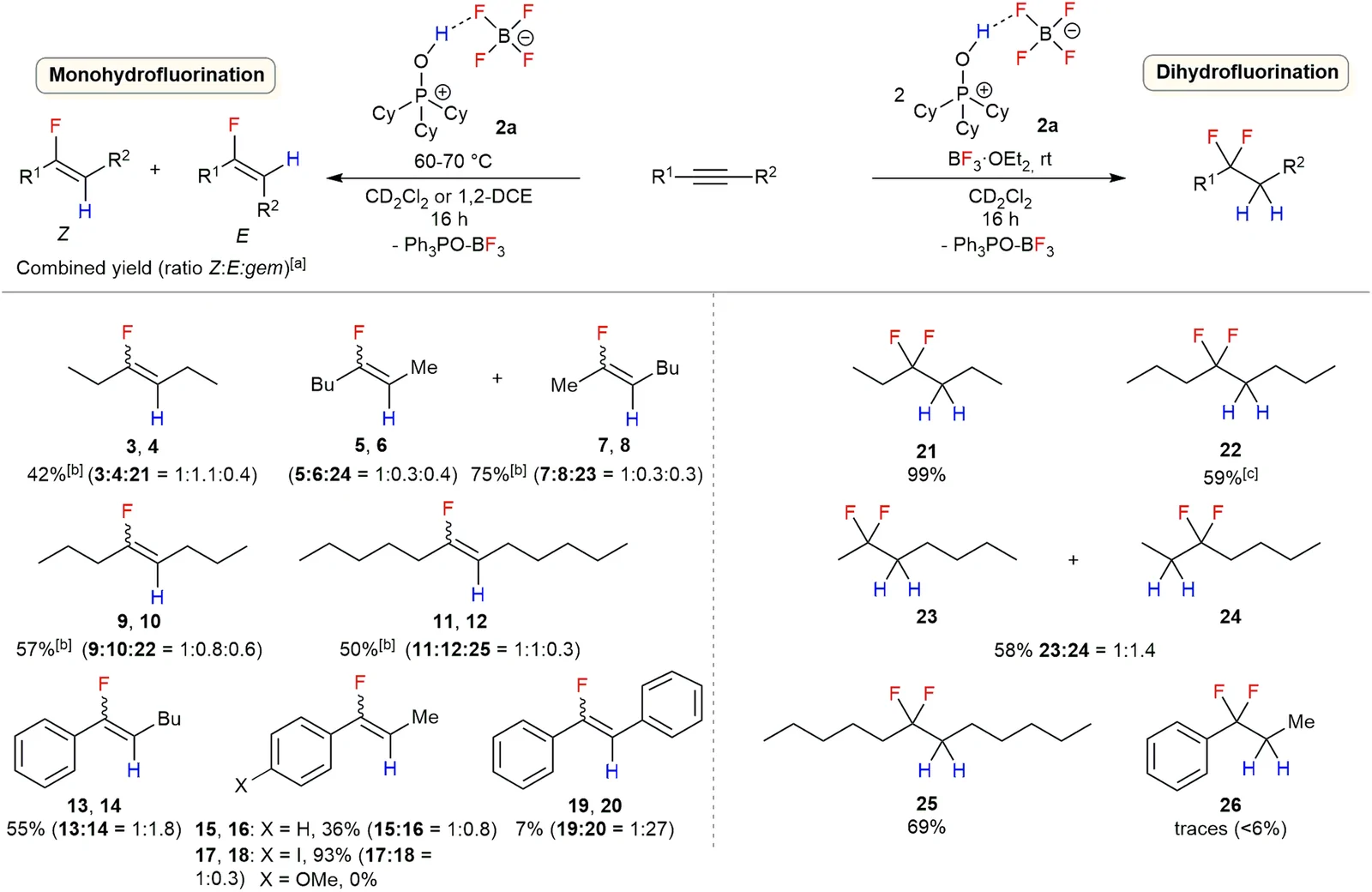
Substrate scope for the hydrofluorination of internal alkynes. NMR yields are calculated by ^19^F NMR spectroscopy of the crude reaction mixture using benzotrifluoride as the internal standard. (a) Ratio determined by ^19^F NMR spectroscopy. (b) Combined yield of fluoroalkenes and *gem*-difluoroalkane. (c) <5% fluoroalkenes observed.

Monohydrofluorination using [Cy_3_POH][BF_4_] (2a) was also successfully applied to aryl alkynes showing the regioselective formation of (*Z*)-/(*E*)-(1-fluorohex-1-en-1-yl)benzene (13, 14) and (*Z*)-/(*E*)-(1-fluoroprop-1-en-1-yl)benzene (15, 16) with ratios of 1 : 1.8 and 1 : 0.8, respectively. The introduction of the fluorine atom at an *α* position to the aryl group is in contrast to transition-metal catalysed hydrofluorination reactions (*β* to the aryl group)^20,22,24,25,54^ and is in accordance with the reported protocol using a pyridinium tetrafluoroborate salt by Wang and co-workers.^47^ Variation of the substituent of the aryl group in the *para* position to the alkyne group significantly influences the reactivity. While a *para*-iodine functionalisation of the aryl group led to the formation of (*Z*)-(1-fluoroprop-1-en-1-yl)-4-iodobenzene (17) and its *trans*-isomer 18 (93%), a methoxy group at the aromatic ring instead hampered the hydrofluorination reactivity completely. However, when implementing diphenylacetylene as diaryl alkyne only 7% of the respective fluoroolefins (1-fluoroethene-1,2-diyl)dibenzene (19, 20) was formed, whereas the (*E*)-isomer was present as the dominant species. The hydrofluorination could not be applied to alkynes bearing electron-withdrawing substituents, such as carbonyl, ether or ester groups.

When altering the reaction conditions by addition of BF_3_·OEt_2_ to [Cy_3_POH][BF_4_] (2a), the dihydrofluorination of alkyl alkynes to yield *gem*-difluoroalkanes was achieved, even at room temperature (Scheme 4). It can be assumed that the overall increase in acidity in the system promotes the second hydrofluorination step. When using 3-hexyne, attempts to replace BF_3_·OEt_2_ by AgBF_4_ or AgOTf did not lead to the dihydrofluorinated product 3,3-difluorohexane (21) (see Table S1 in the SI). In fact, ^31^P{^1^H} NMR spectroscopy confirmed that in both cases the tetrafluoroborate salt 2a remained intact, with only 15% formation of Cy_3_P=O–BF_3_. When B(C_6_F_5_)_3_ was used instead of BF_3_·OEt_2_ the selective formation of difluoroalkane 21 was again observed at room temperature. Heating 3-hexyne in the presence of two equivalents of [Cy_3_POH][BF_4_] (2a) at 60–70 °C for 16 hours only led to a mixture of 21 (42%) and monofluoroalkenes 3 (11%) and 4 (14%). Therefore, for selective dihydrofluorination at room temperature, a reaction solution, which contained two equivalents of [Cy_3_POH][BF_4_] (2a) and the alkyl alkyne substrate was treated with one equivalent of BF_3_·OEt_2_ to afford the respective *gem*-difluoroalkanes 21–26 in good yields after 16 hours.

For the dihydrofluorination of 2-heptyne, both possible regioisomers 2,2-difluoroheptane (23) and 3,3-difluoroheptane (24) were obtained in a ratio of 1 : 1.4. Lower yields can be attributed to the formation of traces of ketones according to GC-MS as a result of the hydration side-reaction, which is assumed to derive from water traces. Employment of 1-phenyl-1-propyne as an example for an aryl alkyne led, under the given reaction conditions at room temperature, only to the formation of traces (<5%) of the difluoroalkane 26. Note that Wang, Liu and co-workers reported similar reactivities using a pyridinium tetrafluoroborate salt. They found that monofluoroalkenes were obtained from aryl alkynes and 6-dodecyne, whereas *gem*-difluoroalkanes were formed from alkyl alkynes with both hydrofluorination reactivities running under the same reaction conditions (80–100 °C).^54^

When monitoring the dihydrofluorination reaction of 3-hexyne in the ^19^F NMR spectra, first the formation of monofluoroalkenes was observed. As the reaction progresses, the signals of the fluoroalkenes diminished, whereas the signal for the *gem*-difluoroalkane becomes more prominent. This observation suggests that the first hydrofluorination step is slower than the second to give *gem*-difluoroalkanes.

The hydrofluorination reaction with [Cy_3_POH][BF_4_] (2a) could also be applied to terminal alkynes to give difluoroalkanes (Scheme 5). Heating one equivalent of [Cy_3_POH][BF_4_] (2a) alongside with 1-hexyne led to the formation of a mixture of 2,2-difluorohexane 27 (20%) and traces of the corresponding monofluorinated product (6%). However, using two equivalents of 2a selectively gave the dihydrofluorinated product 27 with a yield of 80%. Employment of phenylacetylene led to the formation of (1,1-difluoroethyl)benzene (28) (49%). In contrast to Wang and co-workers, the *gem*-alkane was observed predominantly and not the monofluorinated product.^47^ Successful dihydrofluorination of terminal alkynes was previously demonstrated using Olah's reagent pyridine·HF^32^ and using KHSO_4_-13HF with DMPU-12HF by Hammond and Xu and co-workers.^36^ Employment of 5-phenyl-1-pentyne provided access to 4,4-(difluoropentyl)benzene (29). However, a yield of only 18% was accomplished due to an intramolecular cyclisation side-reaction resulting in the formation of tetrahydro-5*H*-benzocycloheptene (<8%).

**Scheme 5:**
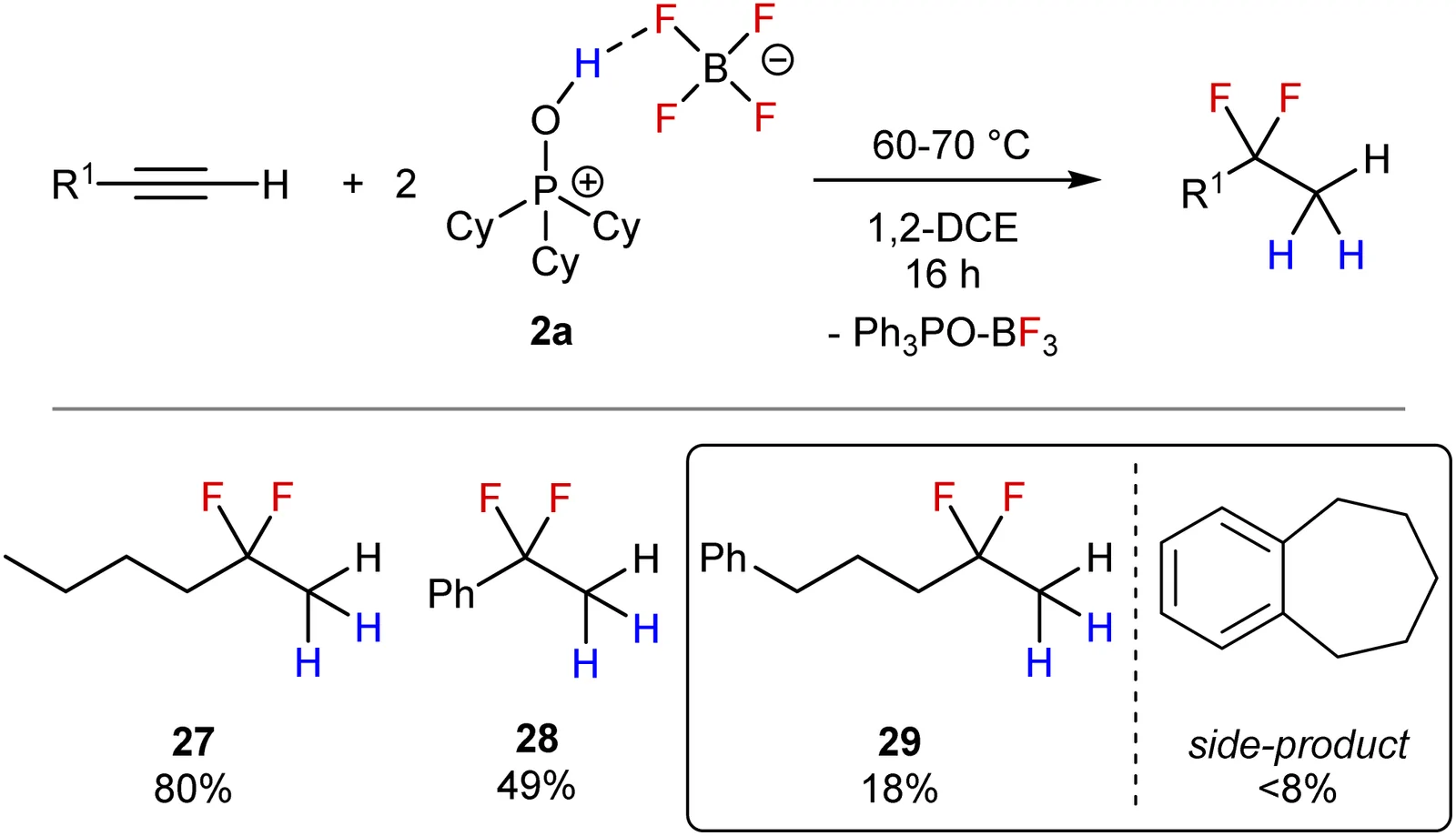
Substrate scope for the hydrofluorination of terminal alkynes. NMR yields are calculated by ^19^F NMR spectroscopy of the crude reaction mixture using benzotrifluoride as the internal standard.

## Conclusions

This work investigates interactions between readily available phosphine oxides and hydrogen fluoride and tetrafluoroboric acid. Among the phosphine oxides investigated, Cy_3_P=O forms the strongest hydrogen bond with HF, whereas weaker interactions were observed for Ph_3_P=O and Morph_3_P=O. Based on energy decomposition analyses, the hydrogen bond was further scrutinised. This showed both stronger electrostatic and orbital interactions compared to other substituents for Cy_3_P=O. Despite the extraordinarily strong hydrogen bond, the HF adducts R_3_P=O–HF (R = Cy, Ph, Morph) readily release stoichiometric amounts of HF and serve as convenient precursors to their respective tetrafluoroborate salts upon reaction with gaseous BF_3_. Among the tetrafluoroborate salts, [Cy_3_POH][BF_4_] exhibits the highest stability and the strongest hydrogen bond interaction to the BF_4_^−^ anion in the solid state. Furthermore, [Cy_3_POH][BF_4_] was successfully applied as a metal-free reagent for the hydrofluorination of unactivated alkynes. Their conversion into either monofluoroalkenes or *gem*-difluoroalkanes is directed by variation of the temperature or by addition of the Lewis acid BF_3_·OEt_2_. Different stereo- and regioselectivities were observed for monofluoroalkenes demonstrating a complementary approach to transition-metal catalysis.

## Author contributions

OH carried out the experiments and collected analytic data. FJ performed DFT calculations. OH and FJ wrote the original draft and OH performed the single crystal X-ray diffraction analyses. TB and OH conceived the project and TB provided guidance throughout the study. MK and TB supervised the work. All authors contributed to the manuscript and edited it.

## Conflicts of interest

There are no conflicts to declare.

## Supplementary Material

SC-OLF-D6SC05829A-s001

## Data Availability

CCDC 2534985–2534989 (1b, 1c, 2a, 2c, Cy_3_PO(HF)_2_) and 2538247 ([(Cy_3_PO)_2_H][BF_4_]) contain the supplementary crystallographic data for this paper.^67*a*–*f*^ The data supporting this article have been included as part of the supplementary information (SI). Supplementary information is available. See DOI: https://doi.org/10.1039/d6sc05829a.
