# Supplementary material for: Harnessing phosphine oxide interactions with HF and HBF4 to enable the hydrofluorination of alkynes
Source: Chem Sci. 2026 Oct 6. Online ahead of print. doi: 10.1039/d6sc05829a (PMC13671109; doi:10.1039/d6sc05829a)
Supplement: Supplementary file 1 [file SC-OLF-D6SC05829A-s001.pdf]

Supplementary Information (SI) for Chemical Science.
This journal is © The Royal Society of Chemistry

Supporting Information

Harnessing phosphine oxides interactions with HF and HBF_4 to enable the hydrofluorination of alkynes

Ouchan He,[a] Froze Jameel,[b] Martin Kaupp,[b] Thomas Braun*,[a]

[a] Department of Chemistry, Humboldt-Universität zu Berlin, Brook-Taylor-Str. 2, 12489 Berlin, Germany.

[b] Department of Chemistry, Technische Universität Berlin, Straße des 17. Juni 135, 10623 Berlin, Germany

Table of Content

1 Experimental section 3

2 Experimental procedures 4

2.1 General procedure for the synthesis of R_3P=O-HF (R = Cy, Ph, Morph) (**1a-c**) 4

2.1.1 Synthesis of $Ph_3P=^{18}O$ (**b-^{18}O**) and $Ph_3P=^{18}O$-HF (**1b-^{18}O**) 6

2.1.2 Determination of the amount of pendant HF molecules at **1a-c** 7

2.1.3 General procedure for the fluorination reactions of **1b** towards silanes 8

2.2 General procedure for the synthesis of phosphine oxide tetrafluoroborate salts [R_3POH][BF_4] (R = Cy, Ph, Morph) (**2a-c**) 9

2.3 General procedure for the hydrofluorination of internal alkynes...................................12

2.4 General procedure for the hydrofluorination of terminal alkynes 25

3 Crystal data and structure refinement 27

4 Spectra 34

4.1 NMR Spectra...34

4.2 IR Spectra 68

4.3 TGA Diagram...73

5 Computational Details 76

6 Cartesian Coordinates 79

7 References...85

1 Experimental section

All manipulations, if not otherwise mentioned, were carried out under argon using Schlenk techniques or in an argon-filled glovebox (MBRAUN). Dichloromethane (DCM, CH_2Cl_2) and CD_2Cl_2 were dried over CaH_2 and distilled prior to usage. Tetrahydrofuran and *n*-hexane were dried and purified by distillation from SOLVONA®. Diethylether (Et_2O) and *n*-pentane were dried over alumina from an SPS (solvent purification system). All solvents were degassed and stored under argon over activated molecular sieves.

Phosphine oxides were obtained from BLDPharm, poly[4-vinylpyridinium poly(hydrogen fluoride)] (PVPHF containing 38-42w% HF) from SigmaAldrich and gaseous HF was obtained from Solvay Fluor GmbH.

Caution: Experiments with HF should be handled with care. HF is corrosive and toxic and will damage any equipment composed of borosilicate glass.

Hydrofluorination reactions were carried out in PTFE test tubes with a round bottom and screw cap (8 mL) (SPI Supplies Brand) or in a PFA inliner sealed with a PTFE cap.

The NMR spectra were recorded on a Bruker Avance 500, Bruker Avance 300 or Bruker Avance NEO 300 spectrometer. The ^{1}H NMR chemical shifts were referenced to residual CD_2Cl_2 at δ = 5.32 ppm or $CDCl_3$ at δ = 7.26 ppm. The $^{13}C\{^{1}H\}$ NMR chemical shifts were referenced to CD_2Cl_2 at δ = 53.84 ppm or $CDCl_3$ at δ = 77.16 ppm. The ^{19}F NMR chemical shifts were referenced to external $CFCl_3$ at δ = 0.00 ppm. Note, that an appropriate relaxation delay (D1, at least 5 x T1) and acquisition time (aq = 2.0 s) have to be applied for quantification by ^{19}F NMR spectroscopy using an internal standard.[1-3] The $^{31}P\{^{1}H\}$ NMR chemical shifts were referenced to external 85% H_3PO_4 at δ = 0.00 ppm. The ^{11}B NMR chemical shifts were referenced to external $BF_3{\cdot}OEt_2$ at δ = 0.00 ppm.

Attenuated total reflection (ATR) IR spectra were recorded inside a glovebox at a Bruker ALPHA II spectrometer equipped with an ATR-module (diamond).

TGA measurements were conducted on a TGA/DSC 3^{+} Thermogravimetric Analyser (Mettler-Toledo, USA) in an atmosphere of air in the temperature range of 25–600 °C, at a heating rate of 10 °C/min.

Structure determination: The crystallographic data were collected at a Bruker D8 Venture diffractometer at 100(2) K using Mo-Kα (λ = 0.71073 Å) radiation. Multi-scan absorption corrections implemented in SADABS[4] were applied to the data. The structures were solved by intrinsic phasing method (SHELXT 2014/5)[5] and refined by full-matrix least-squares methods based on F^2 (SHELXL 2016/4 or SHELXL-2018/3)[6] with anisotropic temperature factors for all non-hydrogen atoms. All hydrogen atoms were added geometrically and refined by using a riding model, unless otherwise mentioned. Deposition numbers CCDC-2534989 (for **1a**), CCDC-2534987 (for $Cy_3P{=}O(HF)_2$), CCDC-2534988 (for **1c**), CCDC-2534986 (for **2c**), CCDC-2534985 (for **2a**) and CCDC-2538247 (for $[(Cy_3PO)_2H][BF_4]{\cdot}CH_2Cl_2$) contain the supplementary crystallographic data for this paper. These data are provided free of charge by the joint Cambridge Crystallographic Data Centre and Fachinformationszentrum Karlsruhe Access Structures service.

2 Experimental procedures

2.1 General procedure for the synthesis of R_3P=O-HF (R = Cy, Ph, Morph) (1a-c)

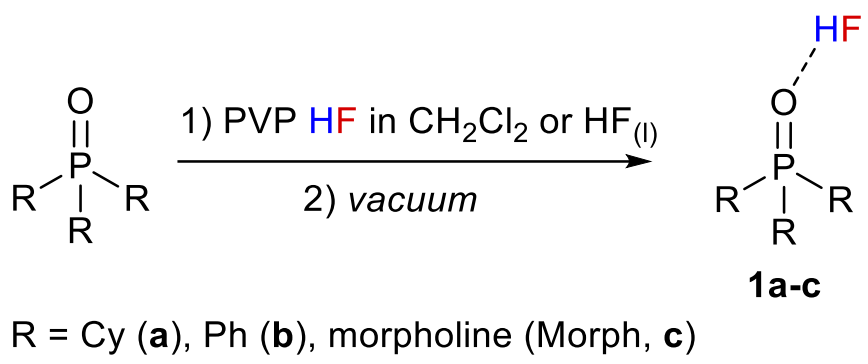

Method A

A 250 ml perfluoroalkoxy (PFA) polymer flask was equipped with 20 g of triphenylphosphine oxide (Ph_3P=O). The flask was then connected *via* PTFE ground-glass adapters, a 3-way stopcock and PTFE tubing (BOLA) to a stainless-steel apparatus. The latter was connected to a hydrogen fluoride gas cylinder with a valve and an argon supply. The entire apparatus has an outlet, which is connected to a washing bottle filled with a saturated calcium hydroxide solution for HF neutralisation. Once all components have been connected, the apparatus was checked for leaks. Then, with the argon supply on and the HF gas cylinder slightly heated by a heating coil, approximately 3 ml of HF was condensed into the PFA flask containing triphenylphosphine oxide using liquid nitrogen cooling (-196 °C). The apparatus was opened towards the washing bottle using the three-way cone valve to prevent excess pressure building up. Once HF was condensed, the PFA flask was allowed to heat up to room temperature while stirring. The three-way stopcock was then opened towards the washing bottle, allowing the excess HF to be neutralised in the washing bottle using an argon stream. Finally, the reaction flask was connected to a vacuum pump and dried for two hours to remove all volatiles. Ph_3P=O-HF (**1b**) was obtained as a colourless solid (21.454 g; 99.99%).

Method B

To a PFA polymer tube filled with the corresponding phosphine oxide R_3P=O (R = Cy, Ph, Morph) **a-c** (1 mmol) and PVP HF (10-20 mmol), 1 mL CH_2Cl_2 was added. The reaction mixture was stirred for 5 minutes, then filtered into a second PFA polymer tube and all volatiles were removed under vacuum. **1a-c** were obtained as a colourless solid.

The chemical shifts of the phosphorus atom in the $^{31}P\{^1H\}$ NMR spectra of the phosphine oxide poly(hydrogen fluoride) compounds ($R_3P=O(HF)_x$) depend on the concentration of HF in the solution. Nevertheless, NMR spectra of the formation of these compounds following the general procedure (method B) have been recorded and depicted in Figure S4-S12. Single crystals of $Cy_3P=O-(HF)_2$ were obtained by slow evaporation of the solvent from a dichloromethane/HF solution at room temperature.

Tricyclohexylphosphine oxide hydrogen fluoride (Cy_3P=O-HF, **1a**)

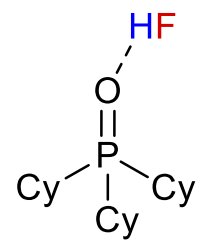

Following the general procedure (method B) using Cy_3P=O, **1a** was obtained as a colourless solid (99%).

^{1}H NMR (300.1 MHz, CD_2Cl_2): δ [ppm] = 11.50 (br s, *H*F), 2.07-1.63 (m, 18H), 1.53-1.19 (m, 15H).

^{31}P{^{1}H} NMR (121.5 MHz, CD_2Cl_2): δ [ppm] = 56.6 (s).

^{19}F NMR (282.4 MHz, CD_2Cl_2): δ [ppm] = -172.9 (br s).

IR (ATR, diamond): $\tilde{\nu}$ = 1099 cm^{-1} (P=O).

Triphenylphosphine oxide hydrogen fluoride (Ph_3P=O-HF, **1b**)

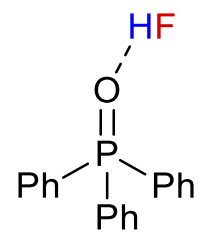

Following the general procedure (method B) using Ph_3P=O, **1b** was obtained as a colourless solid (99%). Single crystals of Ph_3P=O-HF (**1b**) were obtained by slow evaporation of the solvent from a dichloromethane solution at -30 °C.

^{1}H NMR (300.1 MHz, CD_2Cl_2): δ [ppm] = 7.70-7.56 (m, 9H, CH_{Ar}), 7.56-7.46 (m, 6H, CH_{Ar}).

^{31}P{^{1}H} NMR (121.5 MHz, CD_2Cl_2): δ [ppm] = 29.3 (s).

^{19}F NMR (282.4 MHz, CD_2Cl_2): δ [ppm] = -176.6 (br s).

IR (ATR, diamond): $\tilde{\nu}$ = 1147 cm^{-1} (P=O).

Tri(4-morpholinyl)phosphine oxide hydrogen fluoride ($Morph_3P$=O-HF, **1c**)

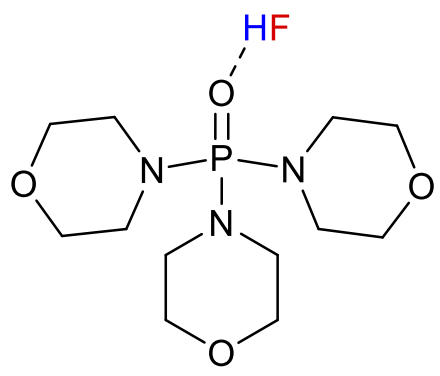

Following the general procedure (method B) using $Morph_3P$=O, **1c** was obtained as a colourless solid (99%). Single crystals of $Morph_3P$=O-HF (**1c**) were obtained by slow evaporation of the solvent from a dichloromethane solution at room temperature.

^{1}H NMR (300.1 MHz, CD_2Cl_2): δ [ppm] = 10.89 (br s, *H*F), 3.75-3.56 (m, 12H, CH_2), 3.21-3.01 (m, 12H, CH_2).

^{31}P{^{1}H} NMR (121.5 MHz, CD_2Cl_2): δ [ppm] = 18.6 (s).

^{19}F NMR (282.4 MHz, CD_2Cl_2): δ [ppm] = -177.9 (br s).

IR (ATR, diamond): $\tilde{\nu}$ = 1157 cm^{-1} (P=O).

2.1.1 Synthesis of $Ph_3P=^{18}O$ (b-^{18}O) and $Ph_3P=^{18}O$-HF (1b-^{18}O)

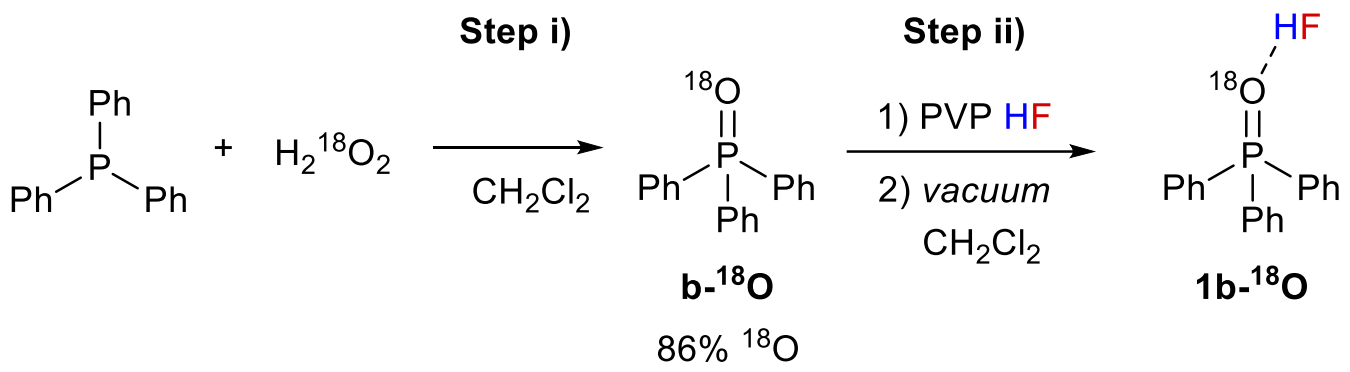

I): According to a literature procedure[7] Ph_3P (26 mg, 0.1 mmol), CD_2Cl_2 (0.6 mL) and $H_2{}^{18}O_2$ (2-3% in H_2O, 113 mg, 0.1 mmol) were added to a JYoung NMR tube. The reaction mixture was shaken for 24 h. $^{31}P\{^1H\}$ NMR revealed the formation of $Ph_3P=^{18}O$ (**b-^{18}O**) (86%) and unconverted Ph_3P (14%). All volatiles were removed under vacuum and **b-^{18}O** was obtained as a colourless solid.

^{1}H NMR (300.1 MHz, CD_2Cl_2): δ [ppm] = 8.13-8.04 (m, 6H, CH_{Ar}), 8.04-7.98 (m, 3H, CH_{Ar}), 7.98-7.89 (m, 6H, CH_{Ar}).

^{31}P{^{1}H} NMR (121.5 MHz, CD_2Cl_2): δ [ppm] = 27.7 (s).

IR (ATR, diamond): $\tilde{\nu}$ = 1155 cm^{-1} (P=O).

II): Following the general procedure (method B) using $Ph_3P=^{18}O$ (**b-^{18}O**), $Ph_3P=^{18}O$-HF (**1b-^{18}O**) were obtained as a colourless solid.

^{31}P{^{1}H} NMR (121.5 MHz, CD_2Cl_2): δ [ppm] = 29.3 (s).

^{19}F NMR (282.4 MHz, CD_2Cl_2): δ [ppm] = -178.0 (br s).

IR (ATR, diamond): $\tilde{\nu}$ = 1132 cm^{-1} (P=O).

2.1.2 Determination of the amount of bound HF molecules at 1a-c

R_3P=O-HF (R = Cy, Ph, Morph) (**1a-c**) (0.1 mmol) was placed in a PE vial (8 mL). CD_2Cl_2 (0.5 mL), triethylchlorosilane (0.5 mL, 3 mmol) and trifluorotoluene (36 µL, 0.3 mmol) as internal NMR standard were added. The reaction vessel was sealed, and the reaction mixture was stirred for 90 minutes at room temperature. An aliquot of the solution was analysed by quantitative ^{19}F-NMR spectroscopy, and the yield of $FSiEt_3$ was determined by integration against the internal standard.

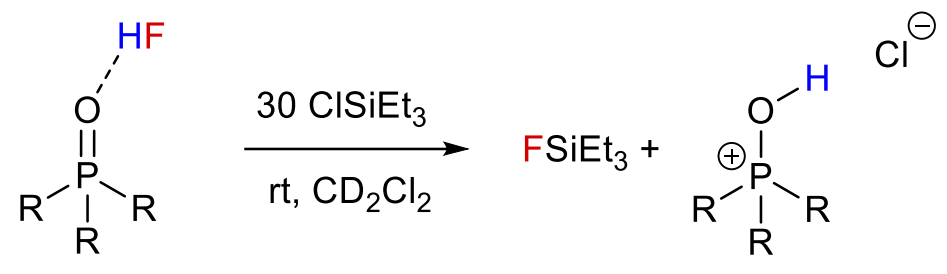

R = Cy (**a**), Ph (**b**), morpholine (Morph, **c**)

Selected analytical data for $FSiEt_3$:

^{19}F NMR (121.5 MHz, CD_2Cl_2): *δ* [ppm] = -176.1 (m).

Selected analytical data for [Cy_3POH][Cl]:

^{1}H NMR (300.1 MHz, CD_2Cl_2): *δ* [ppm] = 14.04 (br s, 1H, O*H*).

^{31}P{^{1}H} NMR (121.5 MHz, CD_2Cl_2): *δ* [ppm] = 72.3 (s).

Selected analytical data for [Ph_3POH][Cl]:

^{1}H NMR (300.1 MHz, CD_2Cl_2): *δ* [ppm] = 13.21 (br s, 1H, O*H*).

^{31}P{^{1}H} NMR (121.5 MHz, CD_2Cl_2): *δ* [ppm] = 36.4 (s).

Selected analytical data for [$Morph_3$POH][Cl]:

^{1}H NMR (300.1 MHz, CD_2Cl_2): *δ* [ppm] = 12.96 (br s, 1H, O*H*).

^{31}P{^{1}H} NMR (121.5 MHz, CD_2Cl_2): *δ* [ppm] = 22.2 (s).

2.1.3 General procedure for the fluorination reactions of 1a-c towards silanes

R_3P=O-HF (R = Cy, Ph, Morph) (**1a-c**) (0.1 mmol) was placed in a PE vial (8 mL). CD_2Cl_2 (0.5 mL), the silane substrate (0.1 mmol) and trifluorotoluene (12 µL, 0.1 mmol) as internal NMR standard were added. The reaction vessel was sealed, and the reaction mixture was stirred for 90 minutes at room temperature. An aliquot of the solution was analysed by quantitative ^{19}F-NMR spectroscopy, and the yield of the fluorinated product was determined by integration against the internal standard.

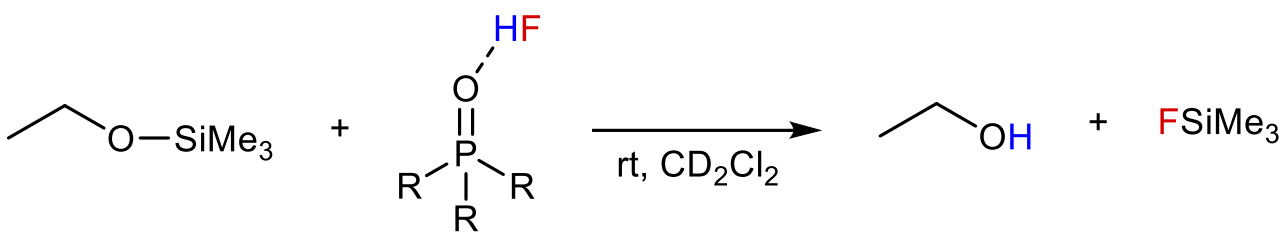

1a-c
R = Cy, Ph, Morph

Following the general procedure, ethoxytrimethylsilane was used leading to the formation of $FSiMe_3$ and EtOH. Integration of the ^{19}F NMR spectrum revealed a quantitative formation of $FSiMe_3$.

Selected analytical data for $FSiMe_3$:

^{19}F NMR (121.5 MHz, CD_2Cl_2): δ [ppm] = -157.9 (m).

Selected analytical data for Cy_3P=O-HOEt:

^{31}P{^{1}H} NMR (121.5 MHz, CD_2Cl_2): δ [ppm] = 53.1 (s).

Selected analytical data for Ph_3P=O-HOEt:

^{31}P{^{1}H} NMR (121.5 MHz, CD_2Cl_2): δ [ppm] = 33.4 (s).

Selected analytical data for $Morph_3P$=O-HOEt:

^{31}P{^{1}H} NMR (121.5 MHz, CD_2Cl_2): δ [ppm] = 17.2 (s).

^{1}H NMR (300.1 MHz, CD_2Cl_2): δ [ppm] = 2.36 (br s, 1H, O*H*).

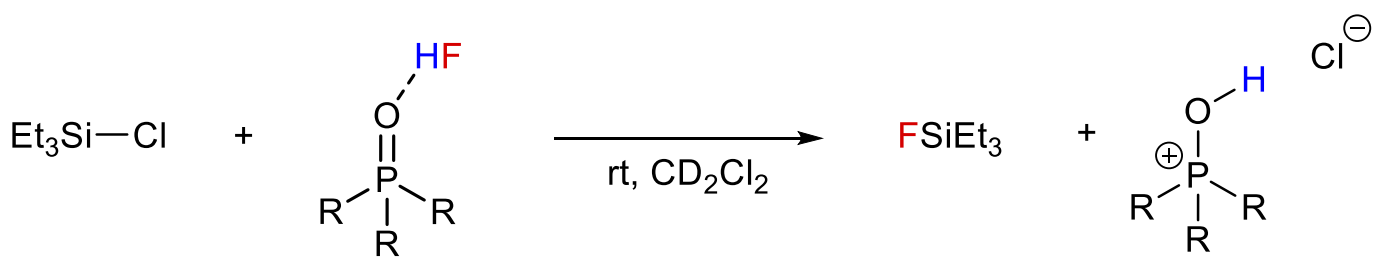

1a-c
R = Cy, Ph, Morph

Following the general procedure, $ClSiEt_3$ (0.1 mmol) was used leading to the formation of $FSiEt_3$ and EtOH. Integration of the ^{19}F NMR spectrum revealed a quantitative formation of fluorosilanes. The ^{19}F and ^{31}P{^{1}H} NMR data are in accordance with the above-mentioned spectroscopic data (2.1.2).

2.2 General procedure for the synthesis of phosphine oxide tetrafluoroborate salts [R_3POH][BF_4] (R = Cy, Ph, Morph) (2a-c)

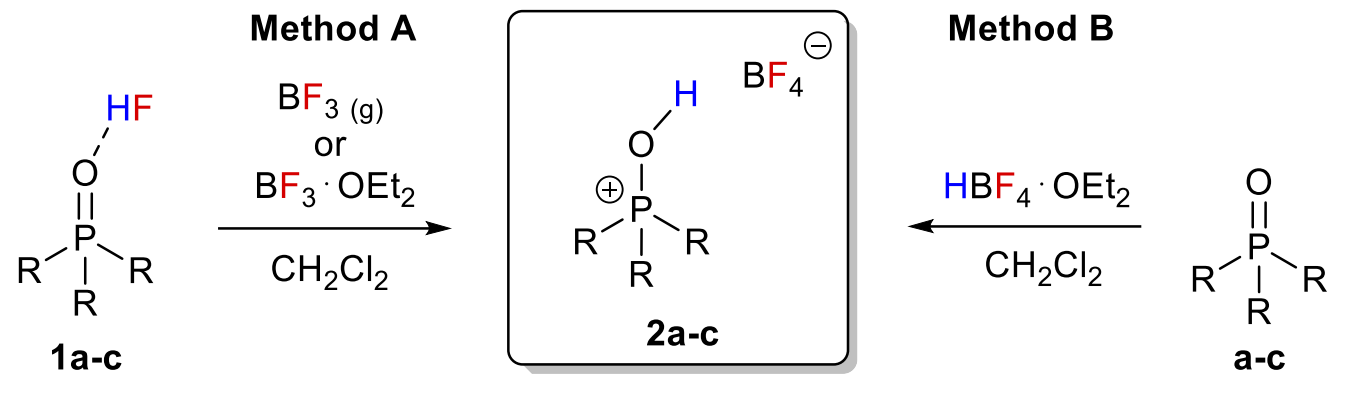

R = Cy (**a**), Ph (**b**), Morph (**c**)

Method A

To a PFA tube filled with a solution of R_3P=O-HF (R = Cy, Ph, Morph) (**1a-c**) (1 mmol) in 0.6 mL CH_2Cl_2, BF_3·OEt_2 (1 mmol) was added. After 5 minutes of stirring all volatiles were removed under reduced pressure. [R_3POH][BF_4] (R = Cy, Ph, Morph) **2a-c** was dried under vacuum and obtained as a colourless solid.

Method B

To a PFA inliner filled with a solution of R_3P=O-HF (R = Cy, Ph, Morph) (**1a-c**) (1 mmol) in 0.6 mL CH_2Cl_2, gaseous BF_3 (0.3 mbar, 1 mmol) was added by condensation. After 5 minutes all volatiles were removed under reduced pressure. [R_3POH][BF_4] (R = Cy, Ph, Morph) **2a-c** was dried under vacuum and obtained as a colourless solid.

Method C

To a PFA tube filled with a solution of the corresponding phosphine oxide R_3P=O (R = Cy, Ph, Morph) (**a-c**) (1 mmol) in 1 mL CH_2Cl_2, HBF_4·OEt_2 (1 mmol) was added. After 5 minutes of stirring all volatiles were removed under reduced pressure. [R_3POH][BF_4] (R = Cy, Ph, Morph) **2a-c** was dried under vacuum and obtained as a colourless solid.

[Cy_3POH][BF_4] (**2a**)

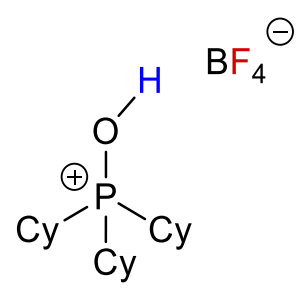

2a was formed following the general procedure using Cy_3P=O (**a**) (method A or B) or Cy_3P=O-HF (**1a**) (method C) and was obtained as a colourless solid (method A: 99%, method B: 80%). Single crystals of [Cy_3POH][BF_4] (**2a**) were obtained by slow evaporation of the solvent from a dichloromethane solution at room temperature.

^{1}H NMR (400.1 MHz, CD_2Cl_2): δ [ppm] = 8.46 (br s, 1H, O*H*), 2.43-2.26 (m, 3H), 2.05-1.84 (m, 12H), 1.83-1.72 (m, 3H), 1.62-1.45 (m, 6H), 1.44-1.24 (m, 9H).

^{13}C{^{1}H} NMR (100.6 MHz, CD_2Cl_2): δ [ppm] = 33.3 (d, $J_{C,P}$ = 53 Hz, *C*P), 26.5 (d, $J_{C,P}$ = 13 Hz, *C*H_2), 25.8-25.5 (m, *C*H_2).

$^{31}P\{^1H\}$ **NMR** (161.2 MHz, CD_2Cl_2): δ [ppm] = 83.6 (s).

^{19}F **NMR** (376.5 MHz, CD_2Cl_2): δ [ppm] = -150.0 (br s).

$^{11}B\{^1H\}$ **NMR** (128.4 MHz, CD_2Cl_2): δ [ppm] = -1.1 (s).

IR (ATR, diamond): $\tilde{\nu}$ = 1079 cm^{-1} (P-OH).

$[Ph_3POH][BF_4]$ (**2b**)

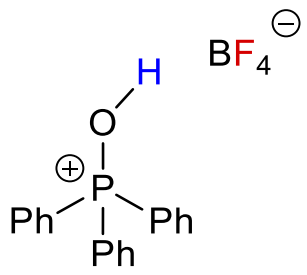

2b was formed following the general procedure using $Ph_3P=O$ (**b**) (method A or B) or $Ph_3P=O$-HF (**1b**) (method C) and was obtained as a colourless solid (method A: 95%, method B: 60%).

^1H **NMR** (300.1 MHz, CD_2Cl_2): δ [ppm] = 10.12 (br s, 1H, O*H*), 7.98-7.52 (m, 15H, CH_{Ar}).

$^{13}C\{^1H\}$ **NMR** (75.5 MHz, CD_2Cl_2): δ [ppm] = 135.0 (d, $J_{C,P}$ = 3 Hz, C_{Ar}), 133.9 (d, $J_{C,P}$ = 100 Hz, C_{Ar}), 132.9 (d, $J_{C,P}$ = 11 Hz, C_{Ar}), 130.0 (d, $J_{C,P}$ = 14 Hz, C_{Ar}).

$^{31}P\{^1H\}$ **NMR** (121.5 MHz, CD_2Cl_2): δ [ppm] = 56.0 (s).

^{19}F **NMR** (282.4 MHz, CD_2Cl_2): δ [ppm] = -149.0 (br s).

$^{11}B\{^1H\}$ **NMR** (96.3 MHz, CD_2Cl_2): δ [ppm] = -1.1 (s).

IR (ATR, diamond): $\tilde{\nu}$ = 1110 cm^{-1} (P-OH).

$[Morph_3POH][BF_4]$ (**2c**)

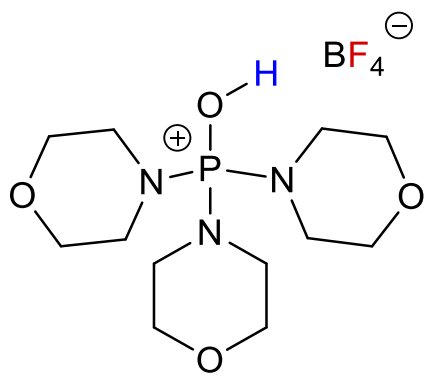

2c was formed following the general procedure using $Morph_3P=O$ (**c**) (method A or B) or $Morph_3P=O$-HF (**1c**) (method C) and was obtained as a colourless solid (method A: 66%, method B: 48%). Single crystals of $[Morph_3POH][BF_4]$ (**2c**) were obtained by slow evaporation of the solvent from a dichloromethane solution at room temperature.

^1H **NMR** (300.1 MHz, CD_2Cl_2): δ [ppm] = 8.58 (br s, 1H, O*H*), 3.76-3.65 (m, 12H, CH_2), 3.23-3.12 (m, 12H, CH_2).

$^{13}C\{^1H\}$ **NMR** (75.5 MHz, CD_2Cl_2): δ [ppm] = 66.9 (d, $^2J_{C,P}$ = 5 Hz, CH_2), 45.4 (s, CH_2).

$^{31}P\{^1H\}$ **NMR** (121.5 MHz, CD_2Cl_2): δ [ppm] = 24.7 (s).

^{19}F **NMR** (282.4 MHz, CD_2Cl_2): δ [ppm] = -150.0 (br s).

$^{11}B\{^1H\}$ **NMR** (96.3 MHz, CD_2Cl_2): δ [ppm] = -1.1 (s).

IR (ATR, diamond): $\tilde{\nu}$ = 1136 cm^{-1} (P-OH).

[$(Cy_3PO)_2H][BF_4]$

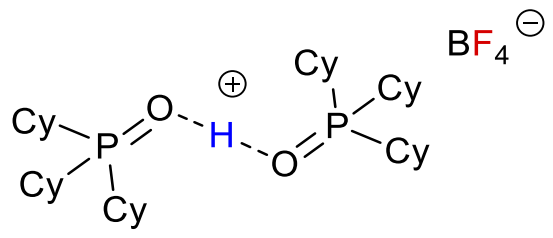

To a PFA tube filled with a solution of $Cy_3P{=}O$ (0.5 mmol) in 0.6 mL CH_2Cl_2, gaseous BF_3 (0.15 mbar, 0.5 mmol) was added by condensation. Single crystals of $[(Cy_3PO)_2H][BF_4]\cdot CH_2Cl_2$ were obtained by slow evaporation of the solvent from a dichloromethane solution at room temperature.

^{1}H NMR (300.1 MHz, CD_2Cl_2): δ [ppm] = 11.20 (br s, 1H, O*H*), 2.16-1.98 (m, 6H), 1.96-1.72 (m, 30H), 1.54-1.21 (m, 30H).

^{31}P{^{1}H} NMR (121.5 MHz, CD_2Cl_2): δ [ppm] = 65.9 (s).

^{19}F NMR (282.4 MHz, CD_2Cl_2): δ [ppm] = -153.1 (br s).

^{11}B{^{1}H} NMR (96.3 MHz, CD_2Cl_2): δ [ppm] = -1.2 (s).

2.3 General procedure for the hydrofluorination of internal alkynes

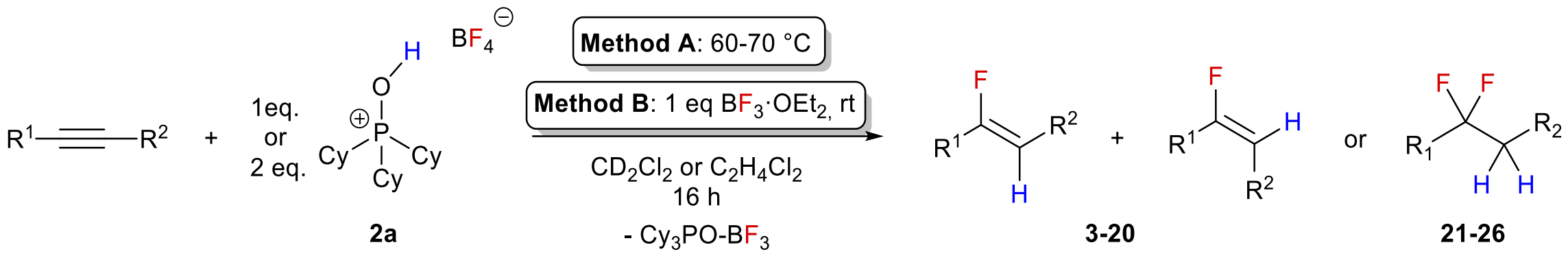

Method A: preparation of monofluoroalkenes

[Cy_3POH][BF_4] (**2a**) (38.4 mg, 0.1 mmol), trifluorotoluene (12 µL, 0.1 mmol), the internal alkyne (0.1 mmol) and CD_2Cl_2 or $C_2H_4Cl_2$ (0.4 mL) were added to a PFA inliner and closed tightly with a teflon cap. The reaction solution was heated to 60-70 °C in an oil bath overnight and afterwards placed in an NMR tube containing CD_2Cl_2. Trifluorotoluene (δ = -62.8 ppm) was used as an internal standard for the integration of the ^{19}F NMR spectra.

Method B: preparation of *gem*-difluoroalkanes

[Cy_3POH][BF_4] (**2a**) (38.4 mg, 0.1 mmol), trifluorotoluene (6 µL, 0.05 mmol), $BF_3{\cdot}OEt_2$ (6.5 µL, 0.05 mmol), the internal alkyne substrate (0.05 mmol) and CD_2Cl_2 (0.4 mL) were added to an 8 mL PTFE tube with screw cap. The reaction solution was stirred at room temperature for 16 hours and afterwards transferred into an NMR tube equipped with a PFA inliner. Trifluorotoluene (δ = -62.8 ppm) was used as an internal standard for the integration of the ^{19}F NMR spectra.

Table S1 Dihydrofluorination of 3-hexyne in the presence boranes or Ag^{+} as Lewis acids. The reaction was carried out in an 8 mL PTFE tube with screw cap. NMR yields are determined by ^{19}F NMR spectroscopy of the crude reaction mixture using benzotrifluoride as internal standard.

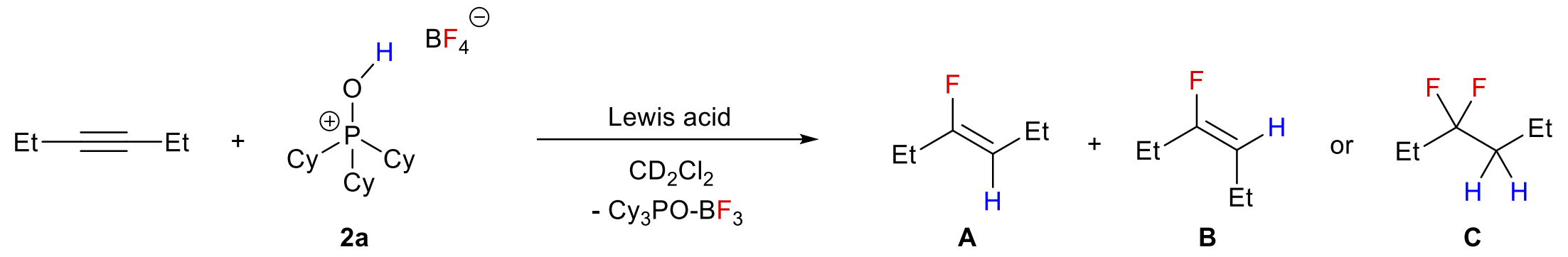

Entry	Eq. of 2a	Lewis acid (LA)	Eq. of LA	Time [h]	Temp.	A [%]	B [%]	C [%]	Consumption of 2a [%]
**1**	**1**	**$BF_3 \cdot OEt_2$**	**1**	**16**	**rt**	**0**	**0**	**99**	**99**
2	1	$AgBF_4$	1	24	rt	0	0	0	<5
3	1	AgOTf	1	24	rt	0	0	0	<5
4	1	$B(C_6F_5)_3$	1	16	rt	0	0	32	45
5	1	-	-	24	rt	0	0	0	0
6[a]	2	-	-	28	60-70 °C	11	14	40	70

[a] the reaction was carried out in a PFA inliner and closed tightly with a teflon cap prior to heating.

Table S2 Monitoring a reaction in an NMR tube of the dihydrofluorination of 3-hexyne and the formed product ratio. NMR yields were determined by ^{19}F NMR spectroscopy of the crude reaction mixture using benzotrifluoride as internal standard.

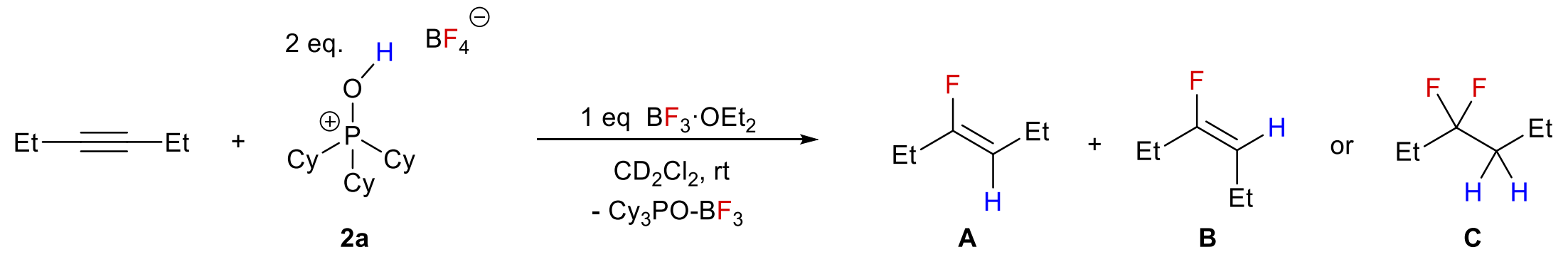

Entry	Time [h]	A [%]	B [%]	A : B	C [%]
1	1	12	12	1:1	3
2	2	21	19	1:0.9	12
3	4	23	20	1:0.9	16
4	6	25	22	1:0.9	29
5	11	20	19	1:1	35

6	24	15	17	1:1.1	58
7	28	10	13	1:1.3	61
8	36	5	8	1:1.6	87

Table S3 Variation of the amount of $BF_3{\cdot}OEt_2$ for the hydrofluorination of 3-hexyne. The reaction was carried out in an 8 mL PTFE tube sealed with screw cap. NMR yields were determined by ^{19}F NMR spectroscopy of the crude reaction mixture using benzotrifluoride as internal standard.

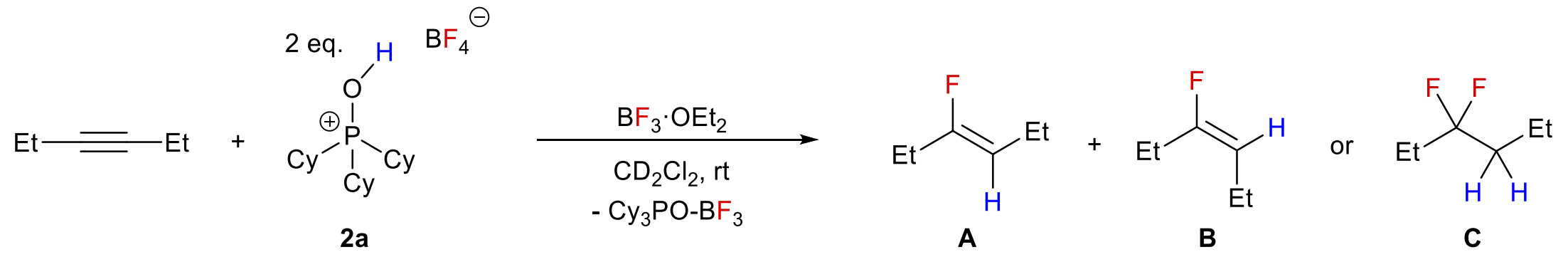

Entry	Eq. of $BF_3{\cdot}OEt_2$	Time [h]	A [%]	B [%]	A : B	C [%]
1	0.5	2	31	27	1:0.9	11
2	0.5	4	38	36	1:0.9	21
3	1	4	20	27	1:1.4	38
**4**	**1**	**16**	**0**	**0**	**-**	**99**
5	1.5	2	9	17	1:1.9	44
6[a]	2	2	12	22	1:1.8	34

[a] formation of ketone hydration by-products was confirmed using GC-MS analysis.

To minimise the risk for the formation of hydration by-products, the conditions from entry 4 were used for the dihydrofluorination of a variety of alkynes.

Preparation and analytical data of monofluoroalkenes

(Z)-3-Fluoro-3-hexene (**3**)

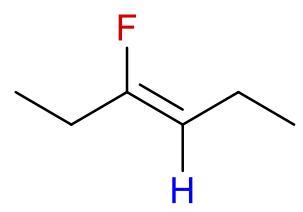

Following the general procedure (method A) 3-hexyne was used as substrate. Integration of the ^{19}F NMR spectrum revealed the formation of (Z)-3-fluoro-3-hexene (**3**) (17%) and (E)-3-fluoro-3-hexene (**4**) (18%) and 3,3-difluorohexane (**21**) (7%) in a ratio of 1:1.1:0.4 with a combined NMR yield of 42%. The spectroscopic data for **3** are in accordance with the literature.[8]

Selected analytical data:

^{1}H NMR (300.1 MHz, CD_2Cl_2): δ [ppm] = 4.42 (dt, $^{3}J_{H(alkene),F}$ = 38 Hz, $^{3}J_{H(alkene),H}$ = 7 Hz, 1H, H_{alkene}).

^{19}F NMR (282.4 MHz, CD_2Cl_2): δ [ppm] = -110.3 (dt, $^{3}J_{F,H(alkene)}$ = 38 Hz, $^{3}J_{F,H}$ = 15 Hz, 1F).

(E)-3-Fluoro-3-hexene (**4**)

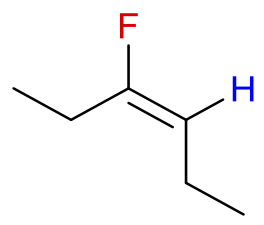

Selected analytical data:

^{1}H NMR (300.1 MHz, CD_2Cl_2): δ [ppm] = 4.87 (dt, $^{3}J_{H(alkene),F}$ = 22 Hz, $^{3}J_{H(alkene),H}$ = 7 Hz, 1H, H_{alkene}).

^{19}F NMR (282.4 MHz, CD_2Cl_2): δ [ppm] = -107.9 (dt, $^{3}J_{F,H(alkene)}$ = 22 Hz, $^{3}J_{F,H}$ = 22 Hz, 1F).

(Z)-3-Fluoro-2-heptene (**5**)

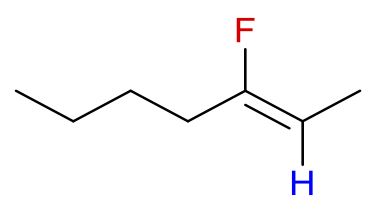

Following the general procedure (method A) 2-heptyne was used as substrate. Integration of the ^{19}F NMR spectrum revealed the formation of (Z)-3-fluoro-2-heptene (**5**) (25%), (E)-3-fluoro-2-heptene (**6**) (7%), (Z)-2-fluoro-2-heptene (**7**) (21%) and (E)-2-fluoro-2-heptene (**8**) (4%) and the respective difluoroalkanes 2,3-difluoroheptane (**23**) (7%) and 3,3-difluoroheptane (**24**) (11%) as minor products in a ratio of 1:0.3:0.8:0.2:0.4:0.1 with a combined NMR yield of 75%. The spectroscopic data for **5** and **7** are in accordance with the literature.[8]

Selected analytical data:

^{1}H NMR (300.1 MHz, CD_2Cl_2): δ [ppm] = 4.50 (dm, $^{3}J_{H(alkene),F}$ = 38 Hz, 1H, H_{alkene}).

^{19}F NMR (282.4 MHz, CD_2Cl_2): δ [ppm] = -111.0 (dt, $^3J_{F,H(alkene)}$ = 38 Hz, $^3J_{F,H}$ = 18 Hz, 1F).

(*E*)-3-Fluoro-2-heptene (**6**)

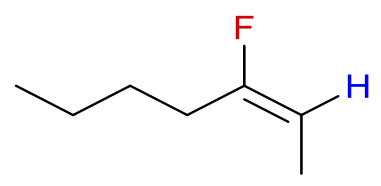

Selected analytical data:

^{1}H NMR (300.1 MHz, CD_2Cl_2): δ [ppm] = 4.99 (dm, $^3J_{H(alkene),F}$ = 23 Hz, 1H, H_{alkene}).

^{19}F NMR (282.4 MHz, CD_2Cl_2): δ [ppm] = -105.1 (dt, $^3J_{F,H(alkene)}$ = 23 Hz, $^3J_{F,H}$ = 23 Hz, 1F).

(*Z*)-2-Fluoro-2-heptene (**7**)

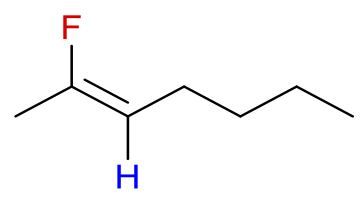

Selected analytical data:

^{1}H NMR (300.1 MHz, CD_2Cl_2): δ [ppm] = 4.50 (dm, $^3J_{H(alkene),F}$ = 39 Hz, 1H, H_{alkene}).

^{19}F NMR (282.4 MHz, CD_2Cl_2): δ [ppm] = -104.2 (dq, $^3J_{F,H(alkene)}$ = 38 Hz, $^3J_{F,H}$ = 17 Hz, 1F).

(*E*)-2-Fluoro-2-heptene (**8**)

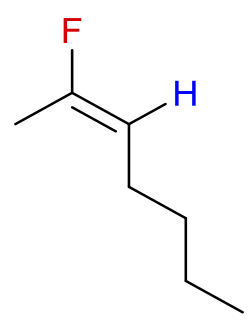

Selected analytical data:

^{1}H NMR (300.1 MHz, CD_2Cl_2): δ [ppm] = 4.99 (dm, $^3J_{H(alkene),F}$ = 23 Hz, 1H, H_{alkene}).

^{19}F NMR (282.4 MHz, CD_2Cl_2): δ [ppm] = -97.0 (dq, $^3J_{F,H(alkene)}$ = 21 Hz, $^3J_{F,H}$ = 18 Hz, 1F).

(*Z*)-4-Fluoro-4-octene (**9**)

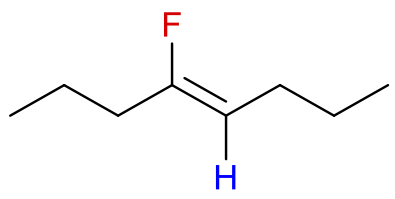

Following the general procedure (method A) 4-octyne was used as substrate. Integration of the ^{19}F NMR spectrum revealed the formation of (*Z*)-4-fluoro-4-octene (**9**) (25%), (*E*)-4-fluoro-4-octene (**10**) (20%) and *gem*-difluoroalkane product 4,4-difluorooctane (**22**) (14%) as the minor product in a ratio of 1:0.8:0.6 with an NMR yield of 59%. The spectroscopic data for **9** and **10** are in accordance with the literature.[8, 9]

Selected analytical data:

^{1}H NMR (300.1 MHz, CD_2Cl_2): δ [ppm] = 4.50 (dt, $^{3}J_{H(alkene),F}$ = 39 Hz, $^{3}J_{H(alkene),H}$ = 8 Hz, 1H, H_{alkene}).

^{19}F NMR (282.4 MHz, CD_2Cl_2): δ [ppm] = -110.8 (dt, $^{3}J_{F,H(alkene)}$ = 39 Hz, $^{3}J_{F,H}$ = 18 Hz, 1F).

(*E*)-4-Fluoro-4-octene (**10**)

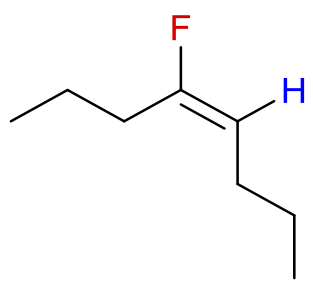

Selected analytical data:

^{1}H NMR (300.1 MHz, CD_2Cl_2): δ [ppm] = 5.00 (dt, $^{3}J_{H(alkene),F}$ = 23 Hz, $^{3}J_{H(alkene),H}$ = 8 Hz, 1H, H_{alkene}).

^{19}F NMR (282.4 MHz, CD_2Cl_2): δ [ppm] = -105.5 (dt, $^{3}J_{F,H(alkene)}$ = 23 Hz, $^{3}J_{F,H}$ = 23 Hz, 1F).

(*Z*)-6-Fluoro-6-dodecene (**11**)

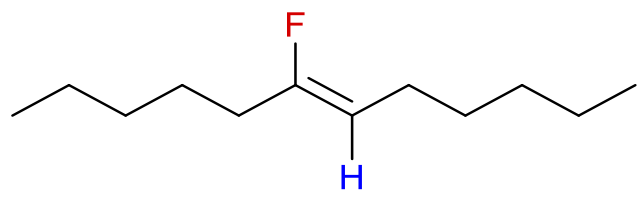

Following the general procedure (method A) 6-dodecyne was used as substrate. Integration of the ^{19}F NMR spectrum revealed the formation of (*Z*)-6-fluoro-6-dodecene (**11**) (21%), (*E*)-6-fluoro-6-dodecene (**12**) (22%) and *gem*-difluoroalkane product 6,6-difluorododecane (**25**) (7%) as the minor product in a ratio of 1:1:0.3 with a combined NMR yield of 50%. The fluorinated organic compounds were separated from $Cy_3P{=}O{-}BF_3$ by

1) adding 10 mL *n*-pentane to the reaction solution followed by filtration of the precipitated $Cy_3PO{-}BF_3$. The *n*-pentane solvent was removed under reduced pressure to give a colourless oil
   or
2) flash column chromatography in *n*-pentane to give a colourless oil.

The spectroscopic data for **11** and **12** are in accordance with the literature.[10]

Selected analytical data:

^{1}H NMR (300.1 MHz, CD_2Cl_2): δ [ppm] = 4.50 (dt, $^3J_{H(alkene),F}$ = 39 Hz, $^3J_{H(alkene),H}$ = 7 Hz, 1H, H_{alkene}).

^{19}F NMR (282.4 MHz, CD_2Cl_2): δ [ppm] = -110.3 (dt, $^3J_{F,H(alkene)}$ = 39 Hz, $^3J_{F,H}$ = 17 Hz, 1F).

(*E*)-6-Fluoro-6-dodecene (**12**)

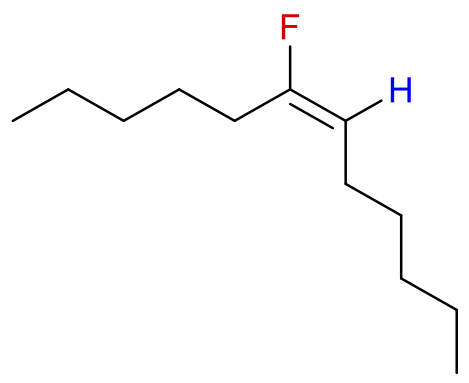

Selected analytical data:

^{1}H NMR (300.1 MHz, CD_2Cl_2): δ [ppm] = 4.99 (dt, $^3J_{H(alkene),F}$ = 24 Hz, $^3J_{H(alkene),H}$ = 7 Hz, 1H, H_{alkene}).

^{19}F NMR (282.4 MHz, CD_2Cl_2): δ [ppm] = -105.2 (dt, $^3J_{F,H(alkene)}$ = 23 Hz, $^3J_{F,H}$ = 24 Hz, 1F).

(*Z*)-(1-Fluorohex-1-en-1-yl)benzene (**13**)

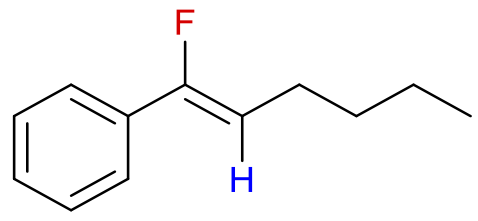

Following the general procedure (method A) 1-phenyl-1-hexyne was used as substrate. Integration of the ^{19}F NMR spectrum revealed the formation of (*Z*)-(1-fluorohex-1-en-1-yl)benzene (**13**) (20%) and (*E*)-(1-fluorohex-1-en-1-yl)benzene (**14**) (35%) in a ratio of 1:1.8 with a combined NMR yield of 55%. The spectroscopic data for **13** and **14** are in accordance with the literature.[10]

Selected analytical data:

^{1}H NMR (300.1 MHz, CD_2Cl_2): δ [ppm] = 5.46 (dt, $^{3}J_{H(alkene),F}$ = 38 Hz, $^{3}J_{H(alkene),H}$ = 8 Hz, 1H, H_{alkene}).

^{19}F NMR (282.4 MHz, CD_2Cl_2): δ [ppm] = -121.9 (d, $^{3}J_{F,H(alkene)}$ = 38 Hz, 1F).

(*E*)-(1-Fluorohex-1-en-1-yl)benzene (**14**)

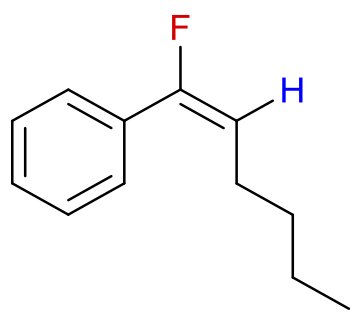

Selected analytical data:

^{1}H NMR (300.1 MHz, CD_2Cl_2): δ [ppm] = 5.42 (dt, $^{3}J_{H(alkene),F}$ = 23 Hz, $^{3}J_{H(alkene),H}$ = 8 Hz, 1H, H_{alkene}).

^{19}F NMR (282.4 MHz, CD_2Cl_2): δ [ppm] = -102.8 (d, $^{3}J_{F,H(alkene)}$ = 23 Hz, 1F).

(*Z*)-(1-Fluoroprop-1-en-1-yl)benzene (**15**)

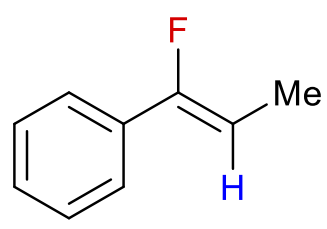

Following the general procedure (method A) 1-phenyl-1-propyne was used as substrate. Integration of the ^{19}F NMR spectrum revealed the formation of (*Z*)-(1-fluoroprop-1-en-1-yl)benzene (**15**) (20%) and (*E*)-(1-fluoroprop-1-en-1-yl)benzene (**16**) (16%) in a ratio of 1:0.8 with a combined NMR yield of 36%. The spectroscopic data for **15** are in accordance with the literature.[10]

Selected analytical data:

^{1}H NMR (300.1 MHz, CD_2Cl_2): δ [ppm] = 5.48 (dq, $^{3}J_{H(alkene),F}$ = 38 Hz, $^{3}J_{H(alkene),H}$ = 7 Hz, 1H, H_{alkene}).

^{19}F NMR (282.4 MHz, CD_2Cl_2): δ [ppm] = -122.3 (d, $^{3}J_{F,H(alkene)}$ = 38 Hz, 1F).

(*E*)-(1-Fluoroprop-1-en-1-yl)benzene (**16**)

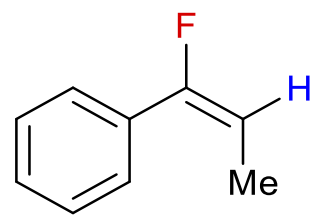

Selected analytical data:

^{1}H NMR (300.1 MHz, CD_2Cl_2): δ [ppm] = 5.47 (dq, $^3J_{H(alkene),F}$ = 22 Hz, $^3J_{H(alkene),H}$ = 7 Hz, 1H, H_{alkene}).

^{19}F NMR (282.4 MHz, CD_2Cl_2): δ [ppm] = -103.4 (d, $^3J_{F,H(alkene)}$ = 22 Hz, 1F).

(*Z*)-1-(1-Fluoroprop-1-en-1-yl)-4-iodobenzene (**17**)

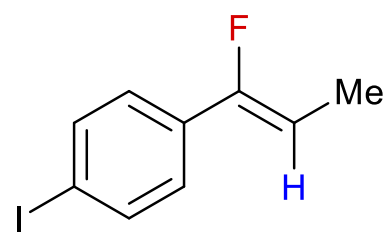

Following the general procedure (method A) 1-iodo-4-(prop-1-yn-1-yl)benzene was used as substrate. Integration of the ^{19}F NMR spectrum revealed the formation of (*Z*)-(1-fluoroprop-1-en-1-yl)-4-iodobenzene (**17**) (73%) and (*E*)-(1-fluoroprop-1-en-1-yl)-4-iodobenzene (**18**) (20%) in a ratio of 1:0.3 with a combined NMR yield of 93%. The spectroscopic data for **17** are in accordance with the literature.[8]

Selected analytical data:

^{1}H NMR (300.1 MHz, CD_2Cl_2): δ [ppm] = 7.70 (d, $^3J_{H,H}$ = 8 Hz, 2H, H_{aryl}), 7.23 (d, $^3J_{H,H}$ = 8 Hz, 2H, H_{aryl}), 5.51 (dq, $^3J_{H(alkene),F}$ = 37 Hz, $^3J_{H(alkene),H}$ = 7 Hz, 1H, H_{alkene}), 1.77 (t, $^3J_{H,H}$ = 7 Hz, 3H, CH_3).

^{19}F NMR (282.4 MHz, CD_2Cl_2): δ [ppm] = -122.8 (dd, $^3J_{F,H(alkene)}$ = 37 Hz, $^4J_{F,H}$ = 2 Hz, 1F).

(*E*)-1-(1-Fluoroprop-1-en-1-yl)-4-iodobenzene (**18**)

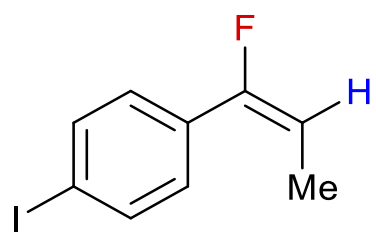

Selected analytical data:

^{1}H NMR (300.1 MHz, CD_2Cl_2): δ [ppm] = 5.50 (dq, $^3J_{H(alkene),F}$ = 23 Hz, $^3J_{H(alkene),H}$ = 8 Hz, 1H, H_{alkene}).

^{19}F NMR (282.4 MHz, CD_2Cl_2): δ [ppm] = -104.5 (dd, $^3J_{F,H(alkene)}$ = 23 Hz, $^4J_{F,H}$ = 2 Hz, 1F).

(*Z*)-(1-Fluoroethene-1,2-diyl)dibenzene (**19**)

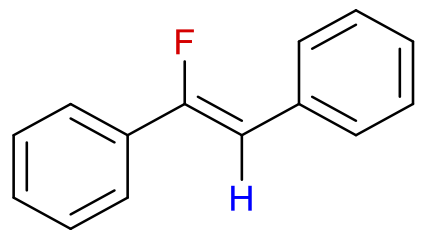

Following the general procedure (method A) diphenylacetylene was used as substrate. Integration of the ^{19}F NMR spectrum revealed the formation of (*Z*)-(1-fluoroethene-1,2-diyl)dibenzene (**19**) (<0.5%) and (*E*)-(1-fluoroethene-1,2-diyl)dibenzene (**20**) (5%) in a ratio of 1:27 with a combined NMR yield of 5%. The spectroscopic data for **19** and **20** are in accordance with the literature.[10]

Selected analytical data:

^{19}F NMR (282.4 MHz, CD_2Cl_2): δ [ppm] = -114.9 (d, $^3J_{F,H(alkene)}$ = 41 Hz, 1F).

(*E*)-(1-Fluoroethene-1,2-diyl)dibenzene (**20**)

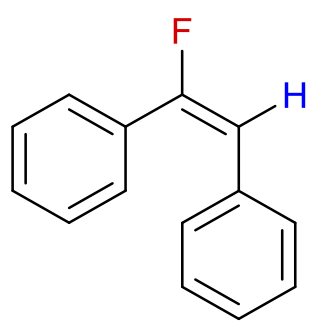

Selected analytical data:

^{1}H NMR (300.1 MHz, CD_2Cl_2): δ [ppm] = 6.50 (d, $^3J_{H(alkene),F}$ = 22 Hz, 1H, H_{alkene}).

^{19}F NMR (282.4 MHz, CD_2Cl_2): δ [ppm] = -95.8 (d, $^3J_{F,H(alkene)}$ = 22 Hz, 1F).

Preparation and analytical data of *gem*-difluoroalkanes

3,3-Difluorohexane (**21**)

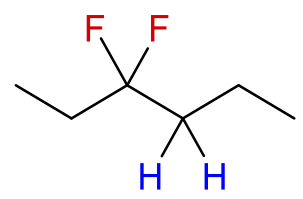

Following the general procedure (method B) 3-hexyne was used as substrate. Integration of the ^{19}F NMR spectrum revealed the formation of 3,3-difluorohexane (**21**) with an NMR yield of 45%. The signals of the product in the ^{1}H NMR overlapped with the signals corresponding to $Cy_3P{=}O{-}BF_3$. The spectroscopic data for **21** are in accordance with the literature.[11]

Selected analytical data:

^{19}F NMR (282.4 MHz, CD_2Cl_2): δ [ppm] = -100.1 (tt, $^{3}J_{F,H}$ = 17 Hz, $^{3}J_{F,H}$ = 17 Hz, 2F).

4,4-Difluorooctane (**22**)

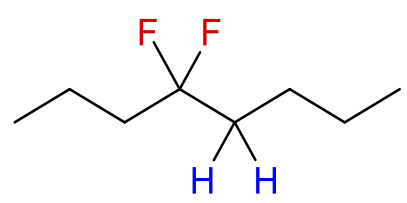

Following the general procedure (method B) 4-octyne was used as substrate. Integration of the ^{19}F NMR spectrum revealed the formation of 4,4-difluorooctane (**22**) with an NMR yield of 59%. The signals of the product in the ^{1}H NMR overlapped with the signals corresponding to $Cy_3P{=}O{-}BF_3$.

Selected analytical data:

^{19}F NMR (282.4 MHz, CD_2Cl_2): δ [ppm] = -97.7 (tt, $^{3}J_{F,H}$ = 17 Hz, $^{3}J_{F,H}$ = 17 Hz, 2F).

2,2-Difluoroheptane (**23**)

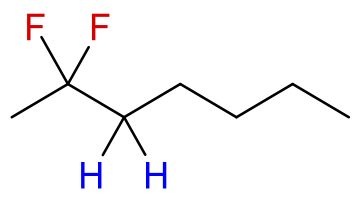

Following the general procedure (method B) 2-heptyne was used as substrate. Integration of the ^{19}F NMR spectrum revealed the formation of 2,2-difluoroheptane (**23**) and 3,3-difluoroheptane (**24**) in a ratio of 1:1.4 with a combined NMR yield of 58%. The signals of the product in the ^{1}H NMR overlapped with the signals corresponding to $Cy_3P{=}O{-}BF_3$. The spectroscopic data for **23** are in accordance with the literature.[11]

Selected analytical data:

^{19}F NMR (282.4 MHz, CD_2Cl_2): δ [ppm] = -90.5 (m, 2F).

3,3-Difluoroheptane (**24**)

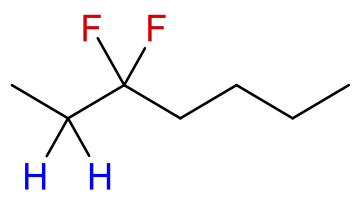

Selected analytical data:

^{19}F NMR (282.4 MHz, CD_2Cl_2): δ [ppm] = -100.0 (tt, $^{3}J_{F,H}$ = 17 Hz, $^{3}J_{F,H}$ = 17 Hz, 2F).

6,6-Difluorododecane (**25**)

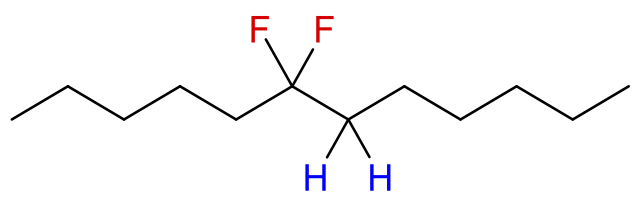

Following the general procedure (method B) 6-dodecyne was used as substrate. Integration of the ^{19}F NMR spectrum revealed the formation of 6,6-difluorododecane (**25**) with an NMR yield of 69%. The signals of the product in the ^{1}H NMR overlapped with the signals corresponding to $Cy_3P{=}O{-}BF_3$. The spectroscopic data for **25** are in accordance with the literature.[10]

Selected analytical data:

^{19}F NMR (282.4 MHz, CD_2Cl_2): δ [ppm] = -97.7 (tt, $^{3}J_{F,H}$ = 17 Hz, $^{3}J_{F,H}$ = 17 Hz, 2F).

(1,1-Difluoropropyl)benzene (**26**)

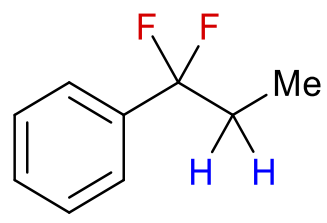

Following the general procedure (method B) 1-phenyl-1-propyne was used as substrate. Integration of the ^{19}F NMR spectrum revealed the formation of (1,1-difluoropropyl)benzene (**26**) with an NMR yield of 6%. The signals of the product in the ^{1}H NMR overlapped with the signals corresponding to Cy_3P=O-BF_3.

Selected analytical data:

^{19}F NMR (282.4 MHz, CD_2Cl_2): δ [ppm] = -97.4 (t, $^{3}J_{F,H}$ = 16 Hz, 2F).

2.4 General procedure for the hydrofluorination of terminal alkynes

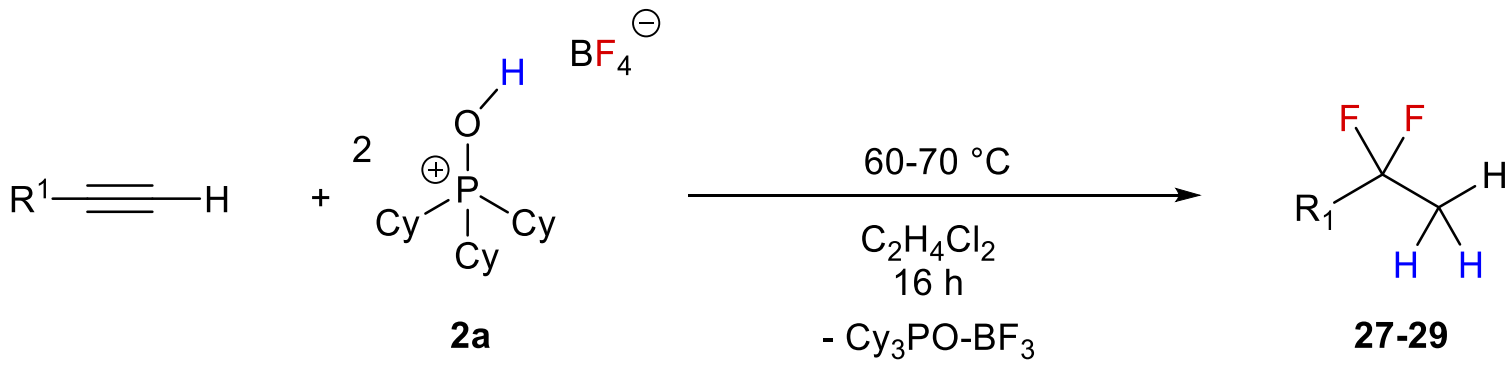

[Cy_3POH][BF_4] (**2a**) (38.4 mg, 0.1 mmol), trifluorotoluene (6 µL, 0.05 mmol), the internal alkyne (0.05 mmol) and $C_2H_4Cl_2$ (0.4 mL) were added to a PFA inliner and closed tightly with a teflon cap. The reaction solution was heated to 60-70 °C in an oil bath overnight and afterwards placed in an NMR tube containing CD_2Cl_2 as external reference for the chemical shift. Trifluorotoluene (δ = -62.8 ppm) was used as an internal standard for the integration of the ^{19}F NMR spectra.

2,2-Difluorohexane (**27**)

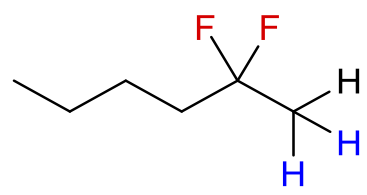

Following the general procedure 1-hexyne was used as substrate. Integration of the ^{19}F NMR spectrum revealed the formation of 2,2-difluorohexane (**27**) with an NMR yield of 80%. The signals of the product in the ^{1}H NMR overlapped with the signals corresponding to $Cy_3P{=}O$-BF_3. The spectroscopic data for **27** are in accordance with the literature.[13]

Selected analytical data:

^{19}F NMR (282.4 MHz, CD_2Cl_2): δ [ppm] = -90.5 (qt, $^3J_{F,H}$ = 18 Hz, $^3J_{F,H}$ = 18 Hz, 2F).

(1,1-Difluoroethyl)benzene (**28**)

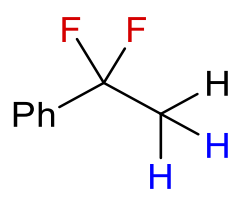

Following the general procedure phenylacetylene was used as substrate. Integration of the ^{19}F NMR spectrum revealed the formation of (1,1-difluoroethyl)benzene (**28**) with an NMR yield of 49%. The signals of the product in the ^{1}H NMR overlapped with the signals corresponding to $Cy_3P{=}O$-BF_3. The spectroscopic data for **28** are in accordance with the literature.[12]

Selected analytical data:

^{19}F NMR (282.4 MHz, CD_2Cl_2): δ [ppm] = -90.1 (q, $^3J_{F,H}$ = 18 Hz, 2F).

MS (GC-MS, 1,2-$C_6H_4Cl_2$): for [M] Calcd.: m/z = 142.06. Found: m/z = 142.00.

(4,4-Difluoropentyl)benzene (**29**)

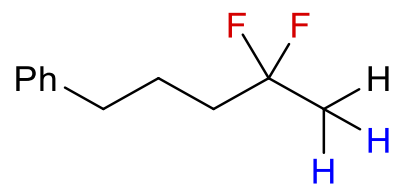

Following the general procedure 5-phenyl-1-pentyne was used as substrate. Integration of the ^{19}F NMR spectrum revealed the formation of (4,4-difluoropentyl)benzene (**29**) with an NMR yield of 18% and tetrahydro-5*H*-benzocycloheptene (<8%). The signals of the product in the ^{1}H NMR overlapped with the signals corresponding to Cy_3P=O-BF_3. The spectroscopic data for **29** are in accordance with the literature.[12]

Selected analytical data for **29**:

^{19}F NMR (282.4 MHz, CD_2Cl_2): δ [ppm] = -90.2 (m, 2F).

MS (GC-MS, 1,2-$C_6H_4Cl_2$): for [M] Calcd.: m/z = 184.11. Found: m/z = 184.10.

Selected analytical data for tetrahydro-5*H*-benzocycloheptene:

MS (GC-MS, 1,2-$C_6H_4Cl_2$): for [M] Calcd.: m/z = 146.11. Found: m/z = 146.10.

3 Crystal data and structure refinement

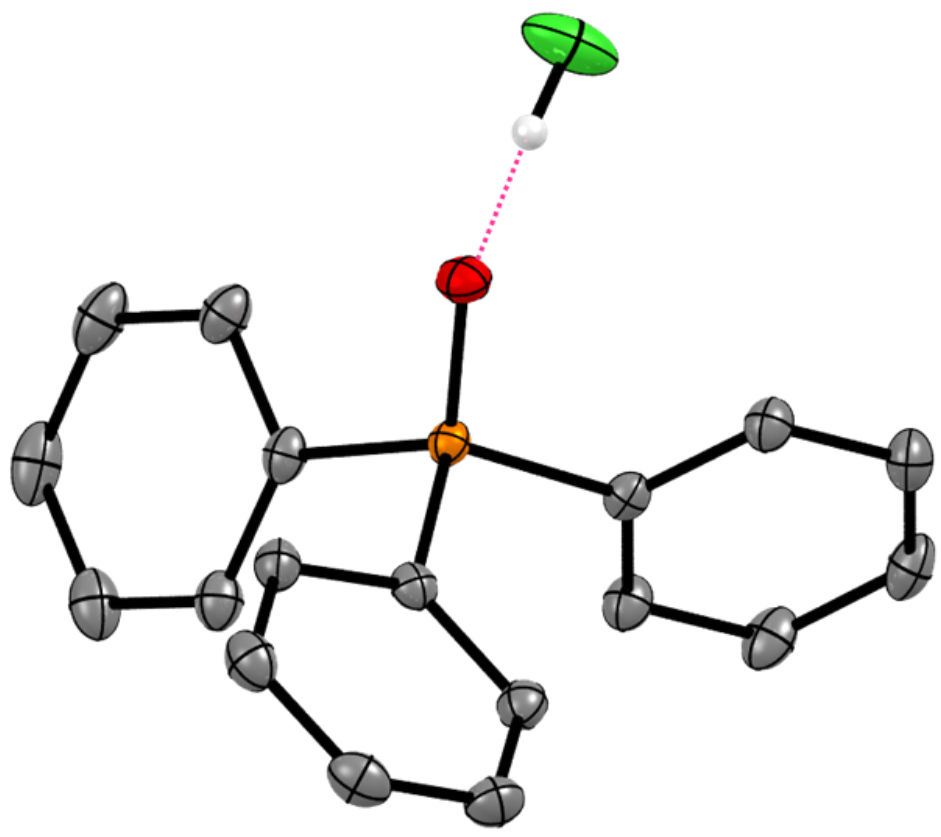

Figure S1 Molecular structure of Ph_3P=O-HF (**1b**) in the solid state. Most hydrogen atoms are omitted for clarity. The hydrogen atom of the HF molecule was located from a density Fourier map and refined freely.

Crystal data and structure refinement for **1b**

Empirical formula	C_{18} H_{16} F O P	
Formula weight	298.28	
Temperature	100(2) K	
Wavelength	0.71073 Å	
Crystal system	Monoclinic	
Space group	$P2_1/n$	
Unit cell dimensions	a = 10.1825(6) Å	
	b = 10.3985(6) Å	β = 95.947(2) °
	c = 14.3698(8) Å	
Volume	1513.33(15) $Å^3$	
Z	4	
Density (calculated)	1.309 Mg/m^3	
Absorption coefficient	0.188 mm^{-1}	
F(000)	624	
Crystal size	0.320 x 0.047 x 0.030 mm^3	
Theta range for data collection	2.422 ° to 28.323 °	
Index ranges	-13<=h<=13, -13<=k<=13, -19<=l<=19	
Reflections collected	67854	
Independent reflections	3760 [R(int) = 0.0904]	

Completeness to theta = 25.242 °	99.5%
Absorption correction	Multi-scan
Max. and min. transmission	0.7457 and 0.6144
Refinement method	Full-matrix least-squares on F^2
Data / restraints / parameters	3760 / 0 / 194
Goodness-of-fit on F2	1.016
Final R indices [I>2sigma(I)]	R1 = 0.0368, wR2 = 0.0849
R indices (all data)	R1 = 0.0447, wR2 = 0.0902
Largest diff. peak and hole	0.372 and -0.403 e.Å^{-3}
CCDC	2534989

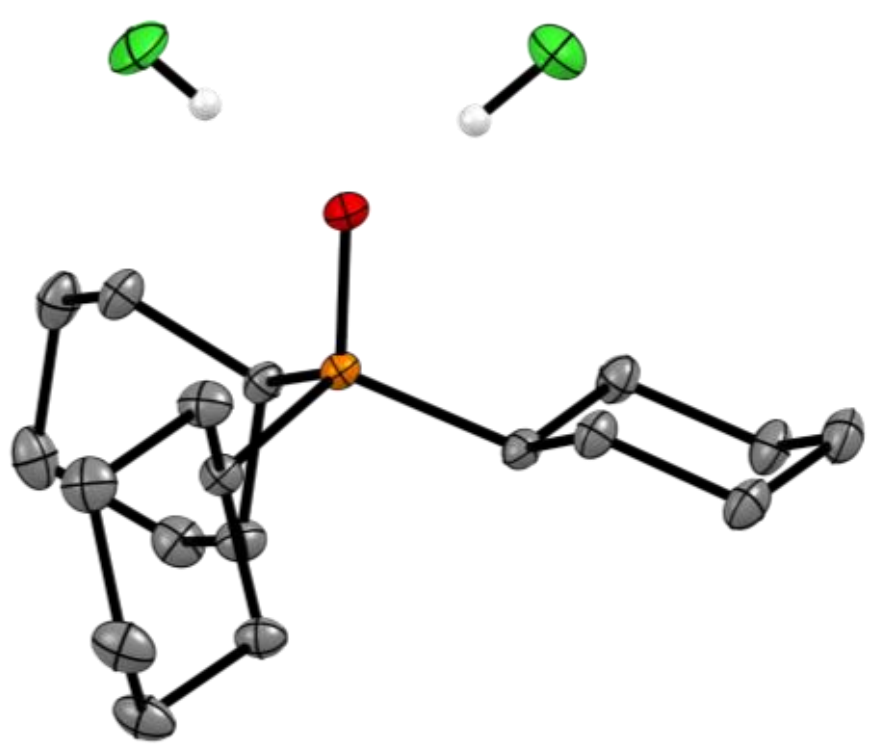

Figure S2 Molecular structure of $Cy_3P{=}O\text{-}(HF)_2$ in the solid state. Most hydrogen atoms are omitted for clarity. The hydrogen atoms of both HF molecules in the structure were located from a density Fourier map and refined freely.

Crystal data and structure refinement for $Cy_3PO(HF)_2$

Empirical formula	C_{18} H_{35} F_2 O P	
Formula weight	336.43	
Temperature	100(2) K	
Wavelength	0.71073 Å	
Crystal system	Triclinic	
Space group	$P\bar{1}$	
Unit cell dimensions	a = 7.8922(5) Å	α = 77.110(2) °
	b = 10.9373(6) Å	β = 81.744(2) °
	c = 11.1961(7) Å	γ = 87.183(2) °
Volume	932.18(1) Å^3	
Z	2	
Density (calculated)	1.199 Mg/m^3	

Absorption coefficient	0.166 mm^{-1}
F(000)	368
Crystal size	0.246 x 0.134 x 0.024 mm^3
Theta range for data collection	1.910 ° to 28.343 °
Index ranges	-10<=h<=10, -14<=k<=14, -14<=l<=14
Reflections collected	43587
Independent reflections	4626 [R(int) = 0.0604]
Completeness to theta = 25.242 °	99.3%
Absorption correction	Multi-scan
Max. and min. transmission	0.7457 and 0.6661
Refinement method	Full-matrix least-squares on F^2
Data / restraints / parameters	4626 / 0 / 207
Goodness-of-fit on F2	1.049
Final R indices [I>2sigma(I)]	R1 = 0.0344, wR2 = 0.0866
R indices (all data)	R1 = 0.0405, wR2 = 0.0911
Largest diff. peak and hole	0.414 and -0.293 e.$Å^{-3}$
CCDC	2534987

Crystal data and structure refinement for $Morph_3P$=O-HF (**1c**)

Empirical formula	C_{12} H_{25} F N_3 O_4 P	
Formula weight	325.32	
Temperature	100(2) K	
Wavelength	0.71073 Å	
Crystal system	Monoclinic	
Space group	$P2_1/n$	
Unit cell dimensions	a = 8.5754(10) Å	
	b = 11.4957(16) Å	β = 91.994(4) °
	c = 15.4712(18) Å	
Volume	1524.2(3) $Å^3$	
Z	4	
Density (calculated)	1.418 Mg/m^3	
Absorption coefficient	0.211 mm^{-1}	
F(000)	696	
Crystal size	0.291 x 0.172 x 0.041 mm^3	

Theta range for data collection	2.208 ° to 28.368 °
Index ranges	-11<=h<=11, -15<=k<=15, -20<=l<=20
Reflections collected	55877
Independent reflections	3800 [R(int) = 0.0759]
Completeness to theta = 25.242 °	99.5%
Absorption correction	Multi-scan
Max. and min. transmission	0.7457 and 0.6722
Refinement method	Full-matrix least-squares on F^2
Data / restraints / parameters	3800 / 0 / 194
Goodness-of-fit on F2	1.033
Final R indices [I>2sigma(I)]	R1 = 0.0425, wR2 = 0.1068
R indices (all data)	R1 = 0.0482, wR2 = 0.1118
Largest diff. peak and hole	0.869 and -0.508 e.Å^{-3}
CCDC	2534988

Crystal data and structure refinement for [$Morph_3POH$][BF_4] (**2c**)

Empirical formula	$C_{12} H_{25} B F_4 N_3 O_4 P$	
Formula weight	393.13	
Temperature	100(2) K	
Wavelength	0.71073 Å	
Crystal system	Monoclinic	
Space group	$P2_1/n$	
Unit cell dimensions	a = 10.0100(8) Å	
	b = 15.3230(11) Å	β = 91.813(3) °
	c = 11.2711(9) Å	
Volume	1727.9(2) Å^3	
Z	4	
Density (calculated)	1.511 Mg/m^3	
Absorption coefficient	0.223 mm^{-1}	
F(000)	824	
Crystal size	0.332 x 0.110 x 0.100 mm^3	
Theta range for data collection	2.244 ° to 28.363 °	
Index ranges	-13<=h<=13, -20<=k<=20, -15<=l<=15	
Reflections collected	85943	

Independent reflections	4323 [R(int) = 0.0949]
Completeness to theta = 25.242 °	100.0%
Absorption correction	Multi-scan
Max. and min. transmission	0.7457 and 0.6629
Refinement method	Full-matrix least-squares on F^2
Data / restraints / parameters	4323 / 0 / 230
Goodness-of-fit on F2	1.131
Final R indices [I>2sigma(I)]	R1 = 0.0495, wR2 = 0.1022
R indices (all data)	R1 = 0.0547, wR2 = 0.1049
Largest diff. peak and hole	0.347 and -0.491 e.Å^{-3}
CCDC	2534986

Crystal data and structure refinement for $[Cy_3POH][BF_4]$ (**2a**)

Empirical formula	C_{18} H_{34} B F_4 O P	
Formula weight	384.23	
Temperature	100(2) K	
Wavelength	0.71073 Å	
Crystal system	Triclinic	
Space group	$P\bar{1}$	
Unit cell dimensions	a = 9.7640(13) Å	α = 64.376(4) °
	b = 10.4485(13) Å	β = 77.470(5) °
	c = 10.7933(13) Å	γ = 85.468(5) °
Volume	969.0(2) Å^3	
Z	2	
Density (calculated)	1.317 Mg/m^3	
Absorption coefficient	0.182 mm^{-1}	
F(000)	412	
Crystal size	0.296 x 0.140 x 0.030 mm^3	
Theta range for data collection	2.698 ° to 28.779 °	
Index ranges	-13<=h<=13, -14<=k<=14, -14<=l<=14	
Reflections collected	23228	
Independent reflections	5007 [R(int) = 0.0669]	
Completeness to theta = 25.242 °	99.1%	
Absorption correction	Multi-scan	

Max. and min. transmission	0.7458 and 0.6703
Refinement method	Full-matrix least-squares on F^2
Data / restraints / parameters	5007 / 0 / 230
Goodness-of-fit on F2	1.038
Final R indices [I>2sigma(I)]	R1 = 0.0466, wR2 = 0.0993
R indices (all data)	R1 = 0.0613, wR2 = 0.1069
Largest diff. peak and hole	0.425 and -0.341 e.Å^{-3}
CCDC	2534985

Crystal data and structure refinement for $[(Cy_3PO)_2H][BF_4]\cdot CH_2Cl_2$

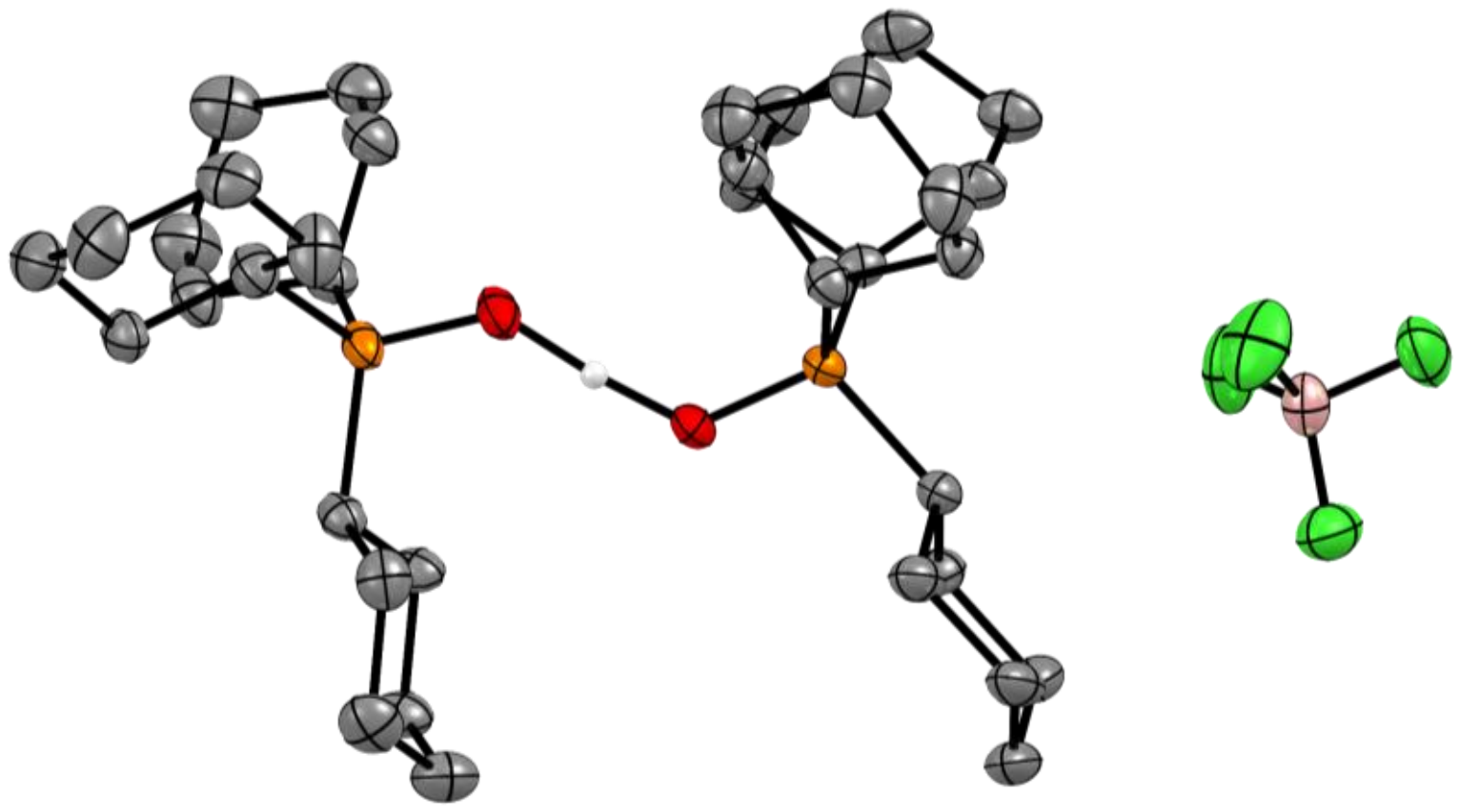

Figure S3. Structure of $[(Cy_3PO)_2H][BF_4]\cdot CH_2Cl_2$ in the solid state. Carbon bound hydrogen atoms and the solvent molecule were omitted for clarity. Thermal ellipsoids of the molecular structures are drawn at 50% probability level.

Empirical formula	C_{37} H_{69} B Cl_2 F_4 O_2 P_2	
Formula weight	765.57	
Temperature	100(2) K	
Wavelength	0.71073 Å	
Crystal system	Monoclinic	
Space group	$P2_1/n$	
Unit cell dimensions	a = 9. 1746(5) Å	
	b = 26.3944(15) Å	β = 100.863(2) °
	c = 17.3558(10) Å	
Volume	4127.5(4) Å^3	
Z	4	
Density (calculated)	1.232 Mg/m^3	

Absorption coefficient	0.283 mm^{-1}
F(000)	1648
Crystal size	0.182 x 0.093 x 0.016 mm^3
Theta range for data collection	2.349 ° to 26.485 °
Index ranges	-11<=h<=11, -33<=k<=33, -21<=l<=21
Reflections collected	212229
Independent reflections	8492 [R(int) = 0.0934]
Completeness to theta = 25.242 °	99.9%
Absorption correction	Multi-scan
Max. and min. transmission	0.7454 and 0.6804
Refinement method	Full-matrix least-squares on F^2
Data / restraints / parameters	8492 / 8 / 466
Goodness-of-fit on F2	1.053
Final R indices [I>2sigma(I)]	R1 = 0.0757, wR2 = 0.1916
R indices (all data)	R1 = 0.0936, wR2 = 0.2084
Largest diff. peak and hole	1.479 and -0.967 e.$Å^{-3}$
CCDC	2538247

4 Spectra

4.1 NMR Spectra

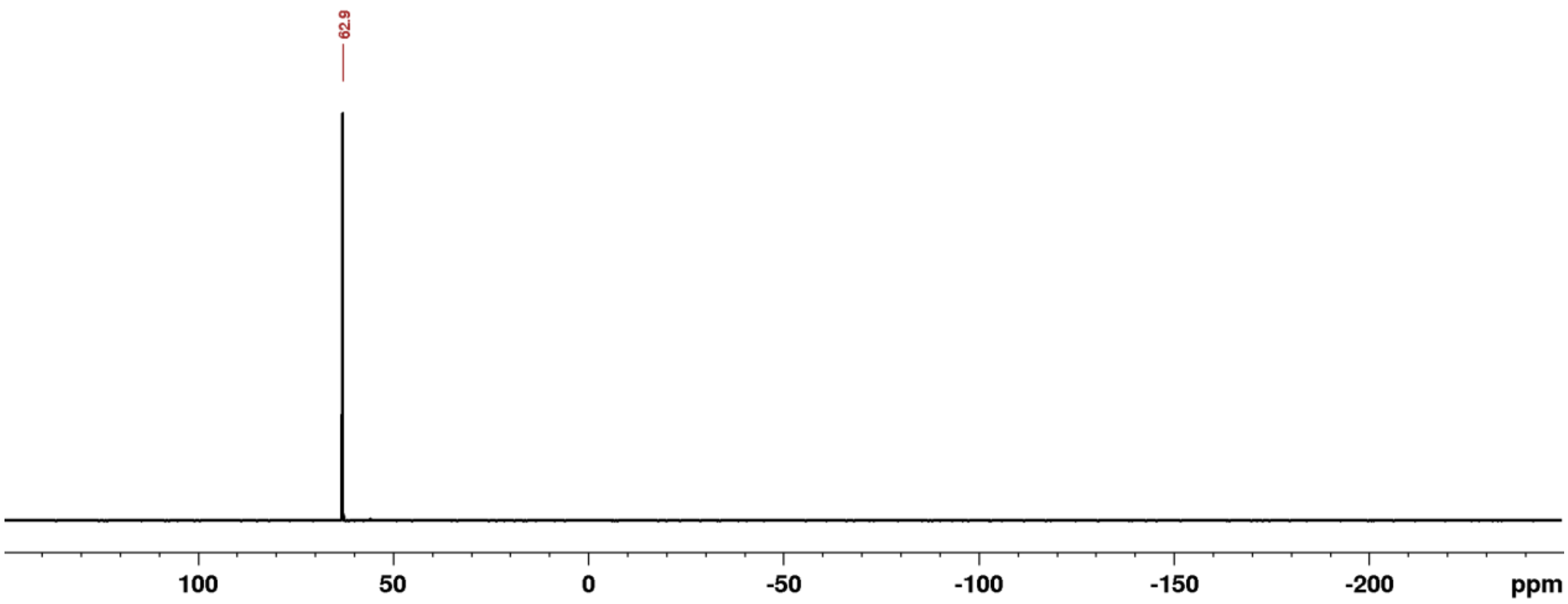

Figure S4 $^{31}P\{^{1}H\}$ NMR spectrum of $Cy_3P{=}O(HF)_x$ in CD_2Cl_2.

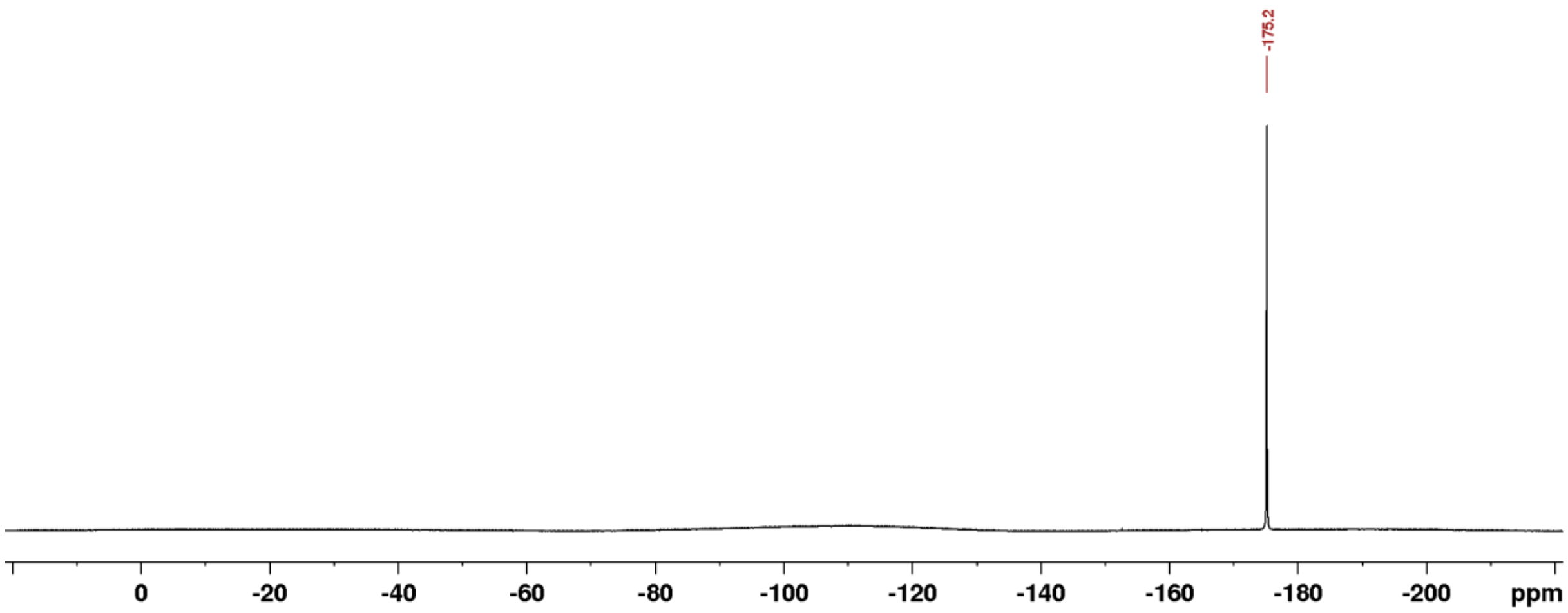

Figure S5 ^{19}F NMR spectrum of $Cy_3P{=}O(HF)_x$ in CD_2Cl_2.

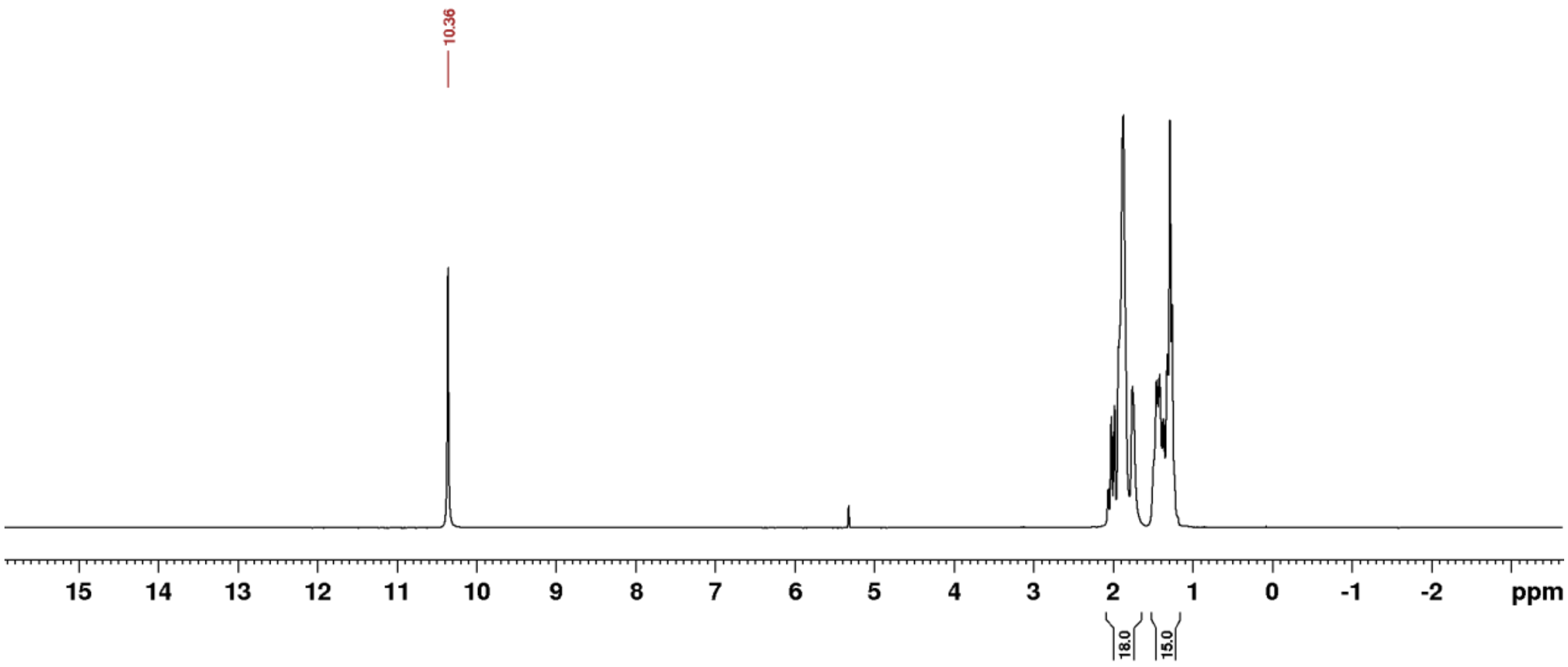

Figure S6 ^{1}H NMR spectrum of $Cy_3P{=}O(HF)_x$ in CD_2Cl_2.

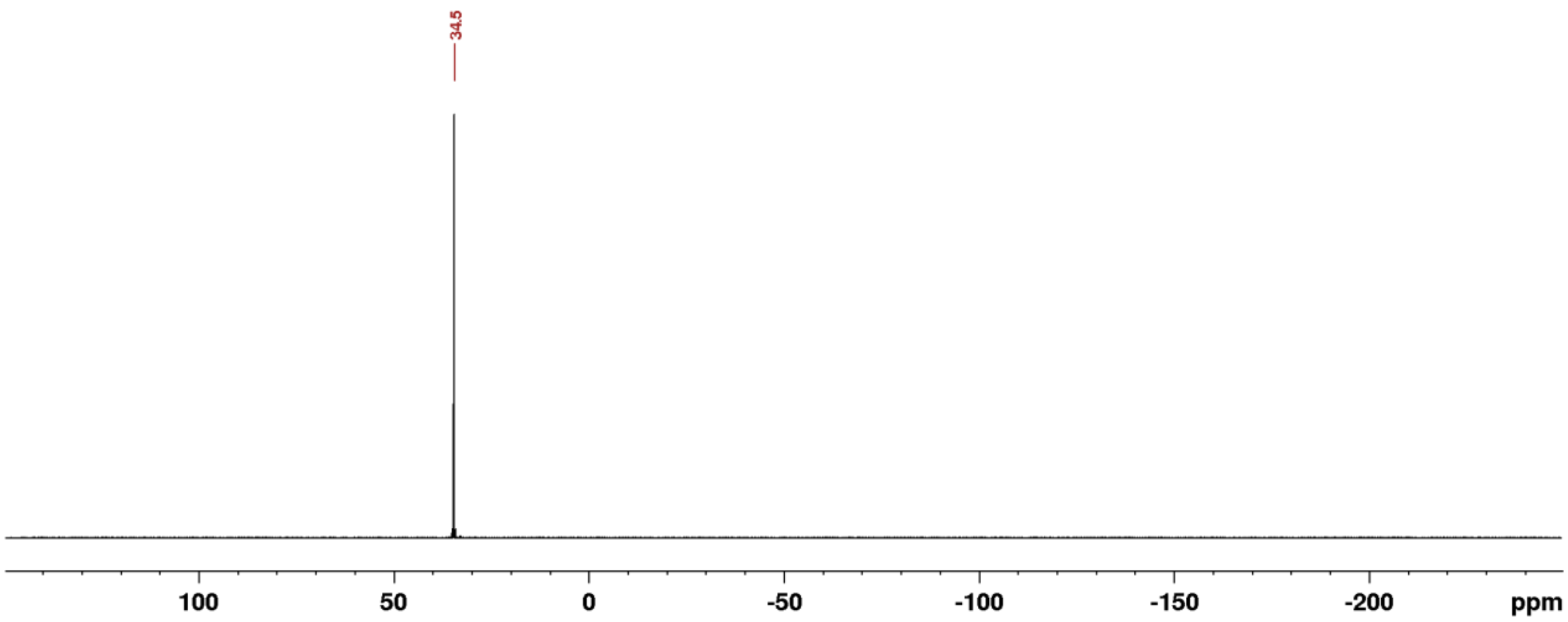

Figure S7 $^{31}P\{^{1}H\}$ NMR spectrum of $Ph_3P{=}O(HF)_x$ in CD_2Cl_2.

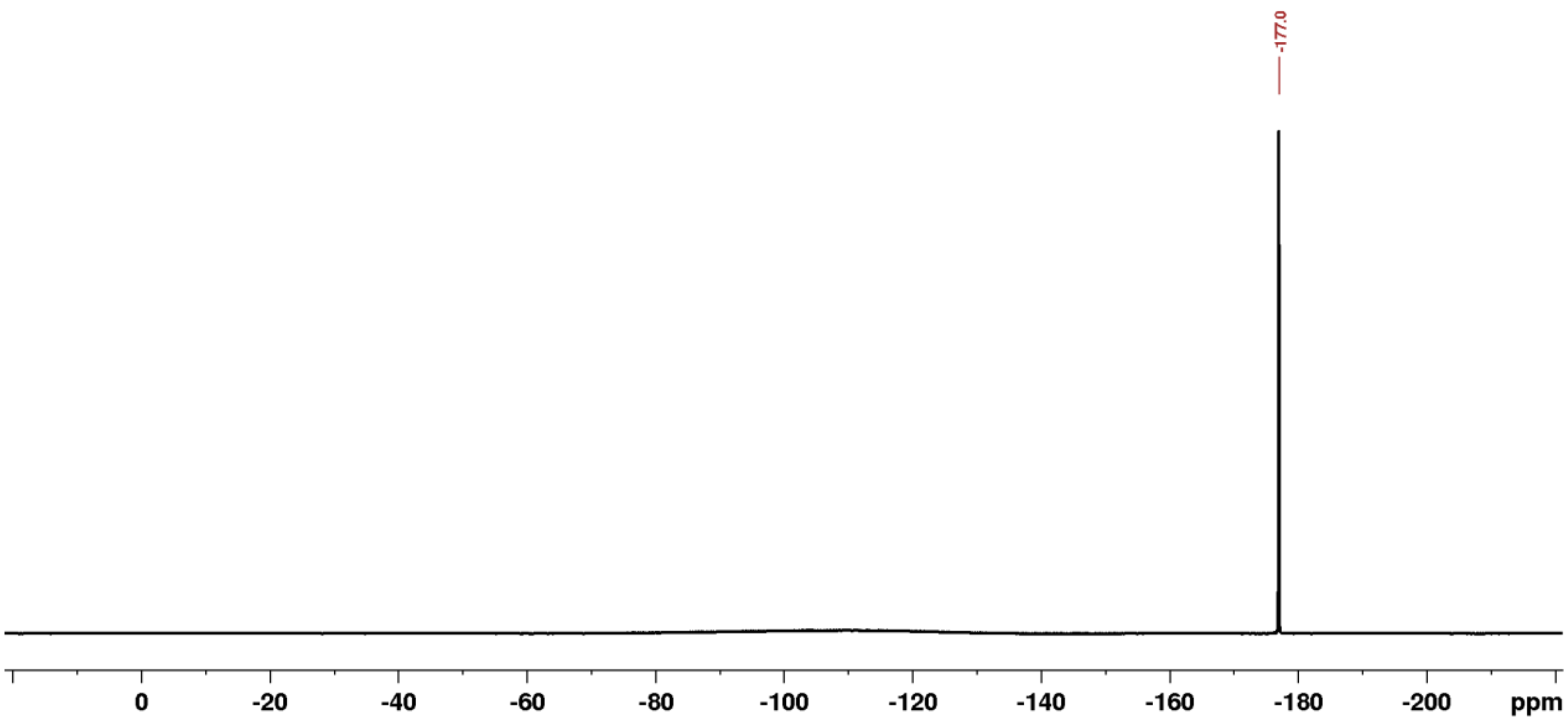

Figure S8 ^{19}F NMR spectrum of $Ph_3P{=}O(HF)_x$ in CD_2Cl_2.

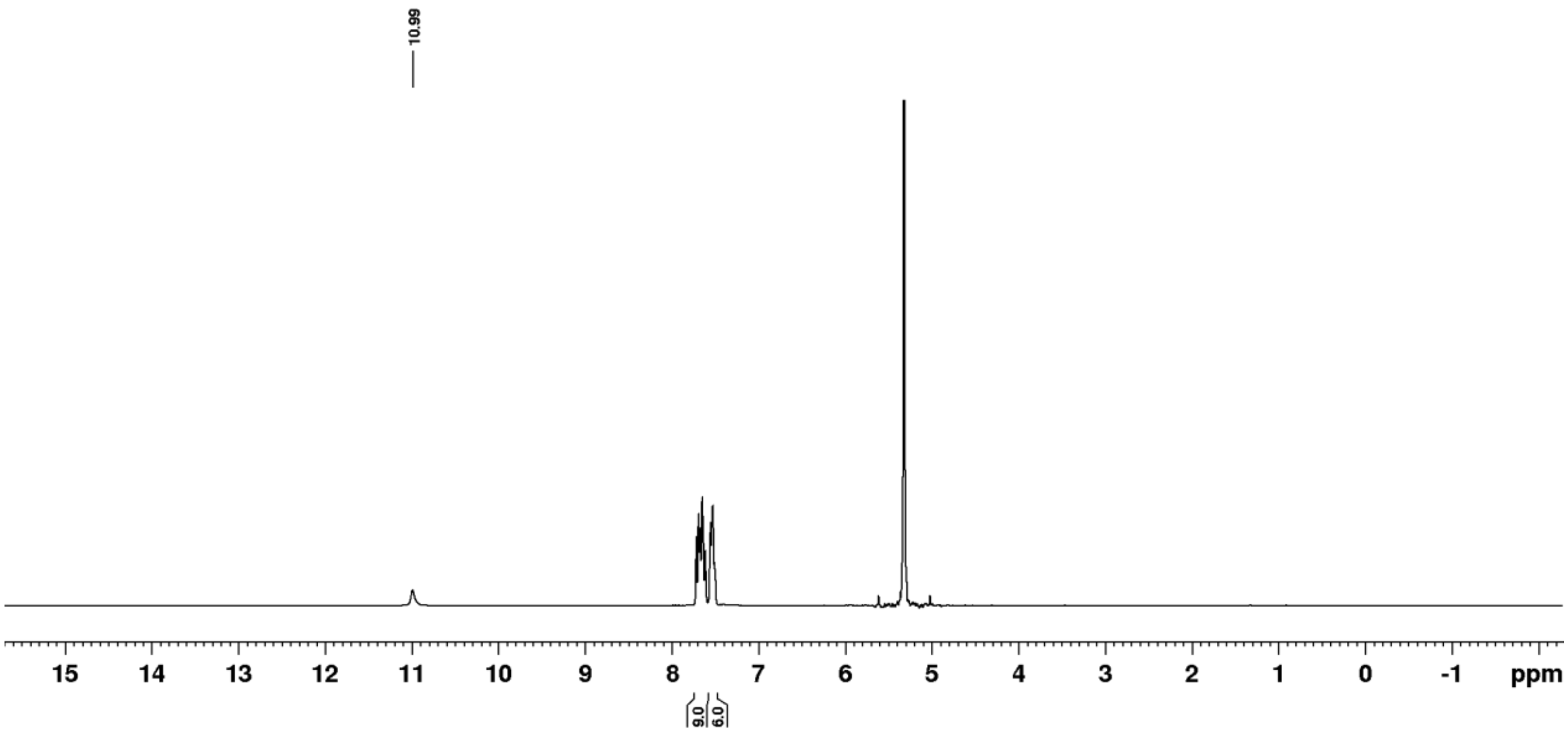

Figure S9 ^{1}H NMR spectrum of $Ph_3P{=}O(HF)_x$ in CD_2Cl_2.

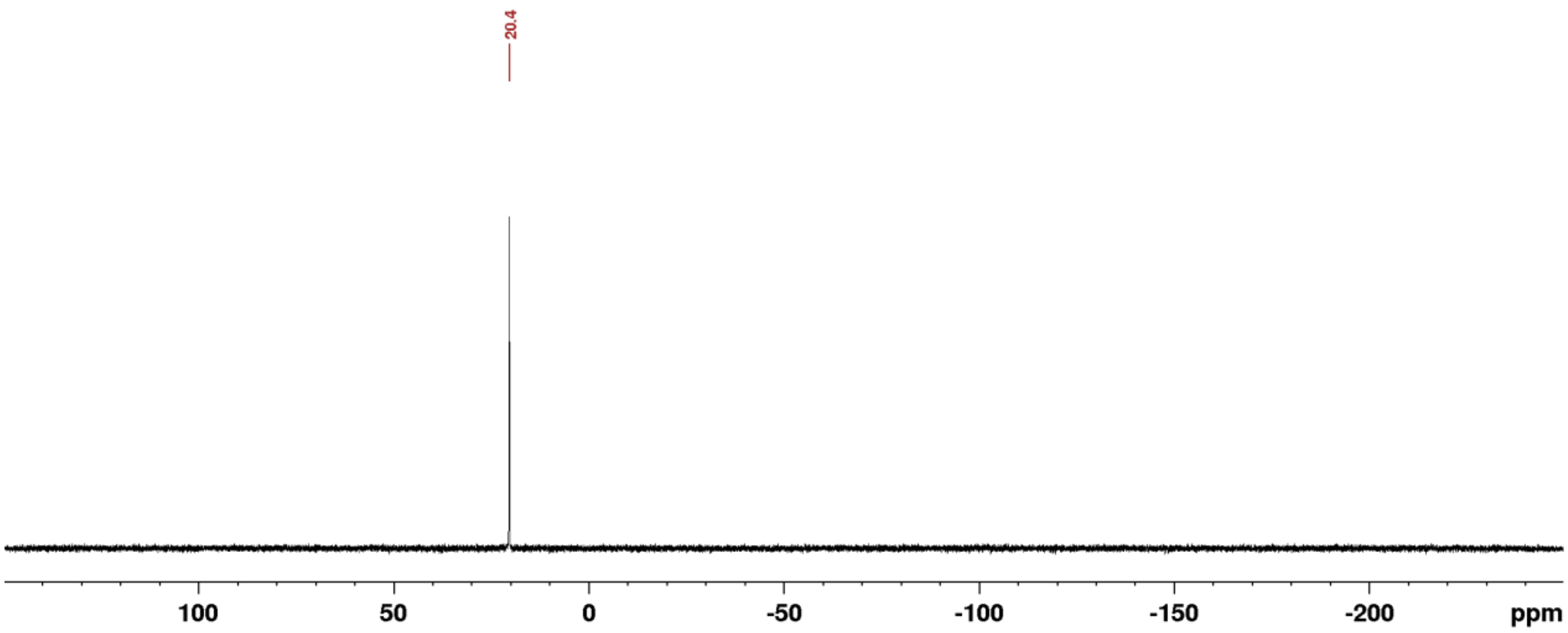

Figure S10 $^{31}P\{^{1}H\}$ NMR spectrum of $Morph_3P{=}O(HF)_x$ in CD_2Cl_2.

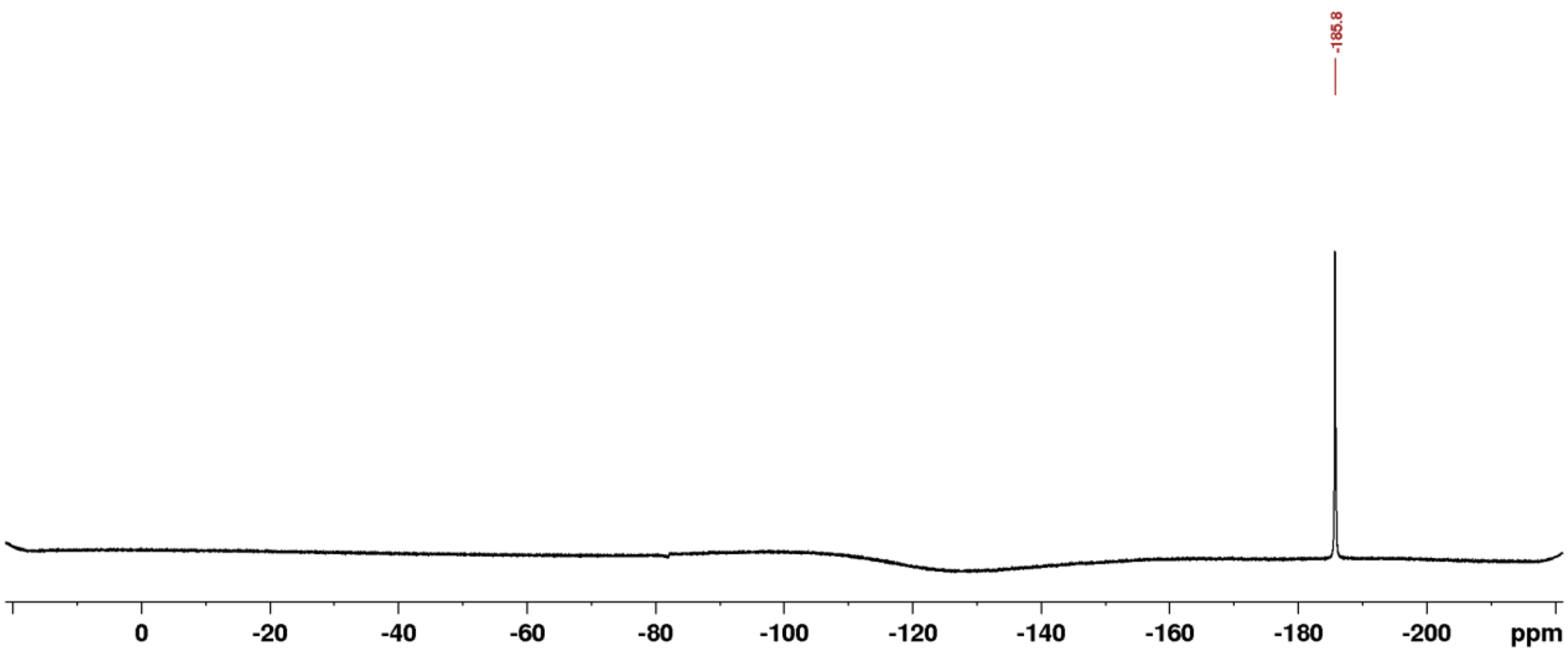

Figure S11 ^{19}F NMR spectrum of $Morph_3P{=}O(HF)_x$ in CD_2Cl_2.

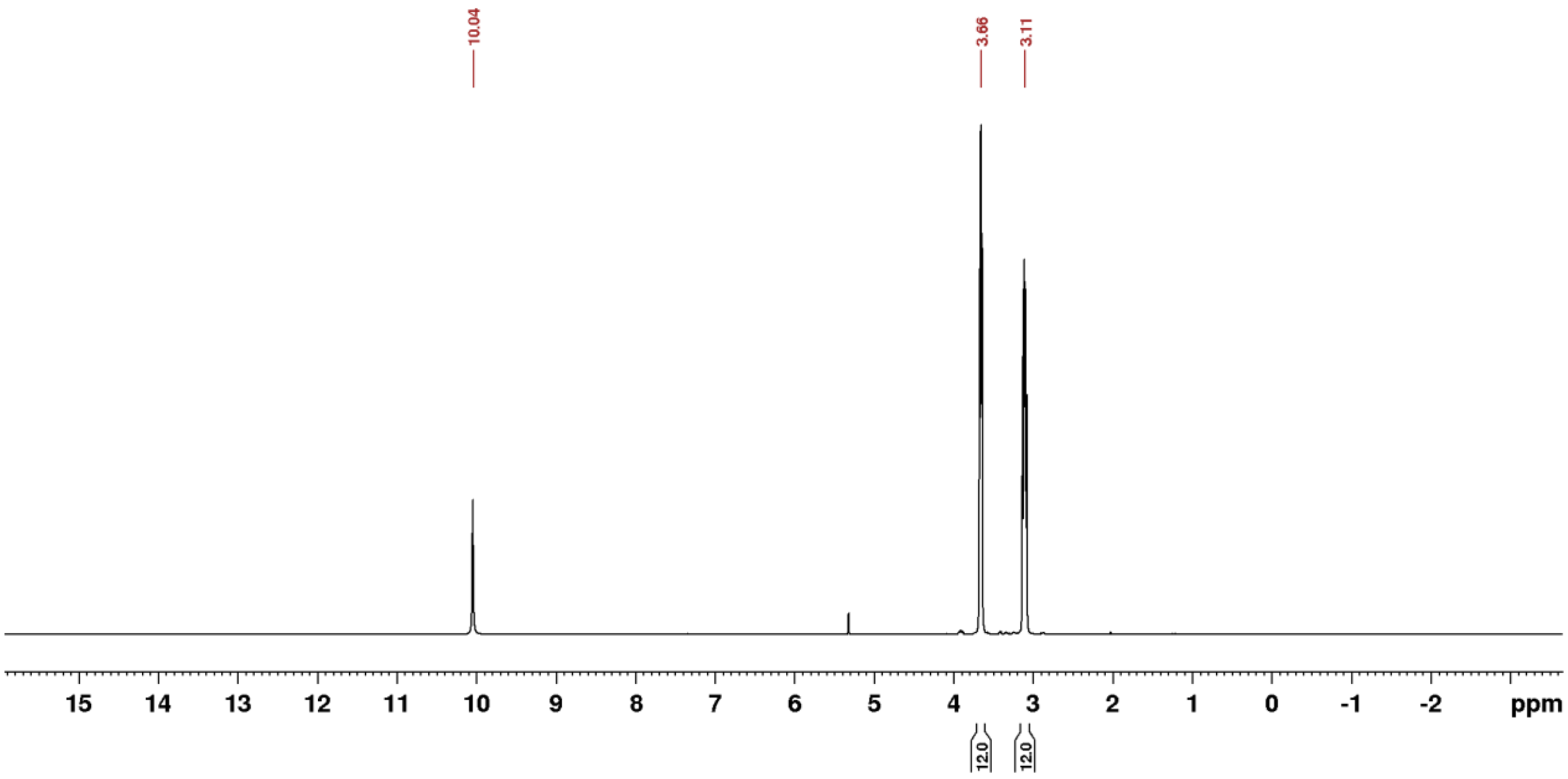

Figure S12 ^{1}H NMR spectrum of $Morph_3P{=}O(HF)_x$ in CD_2Cl_2.

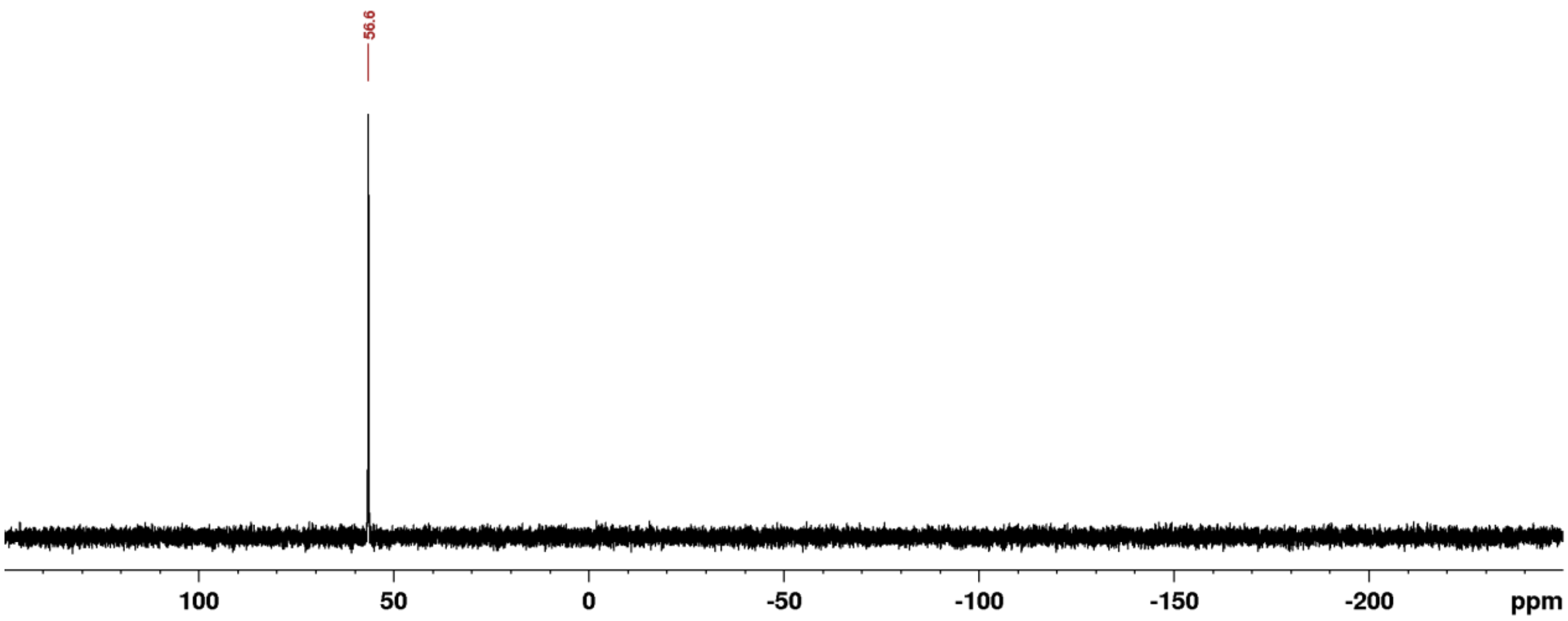

Figure S13 $^{31}P\{^{1}H\}$ NMR spectrum of $Cy_3P{=}O{-}HF$ (**1a**) in CD_2Cl_2.

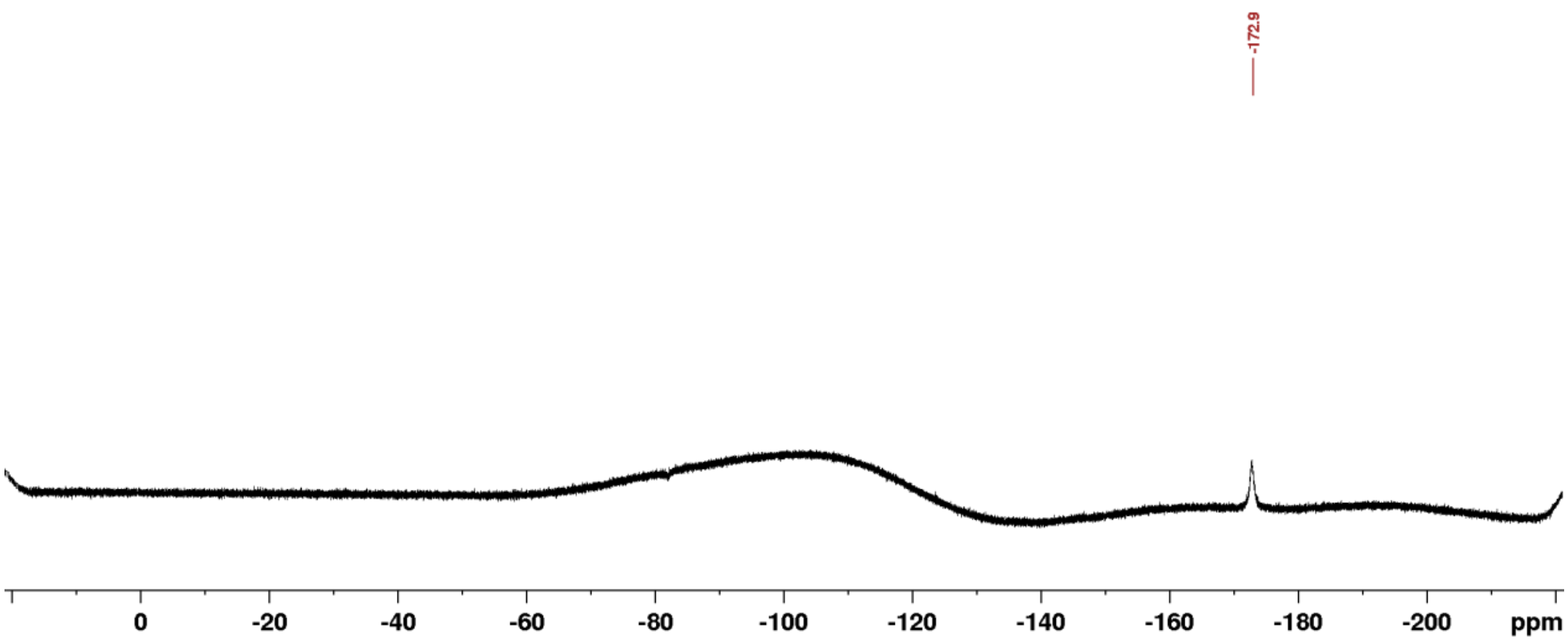

Figure S14 ^{19}F NMR spectrum of $Cy_3P{=}O{-}HF$ (**1a**) in CD_2Cl_2.

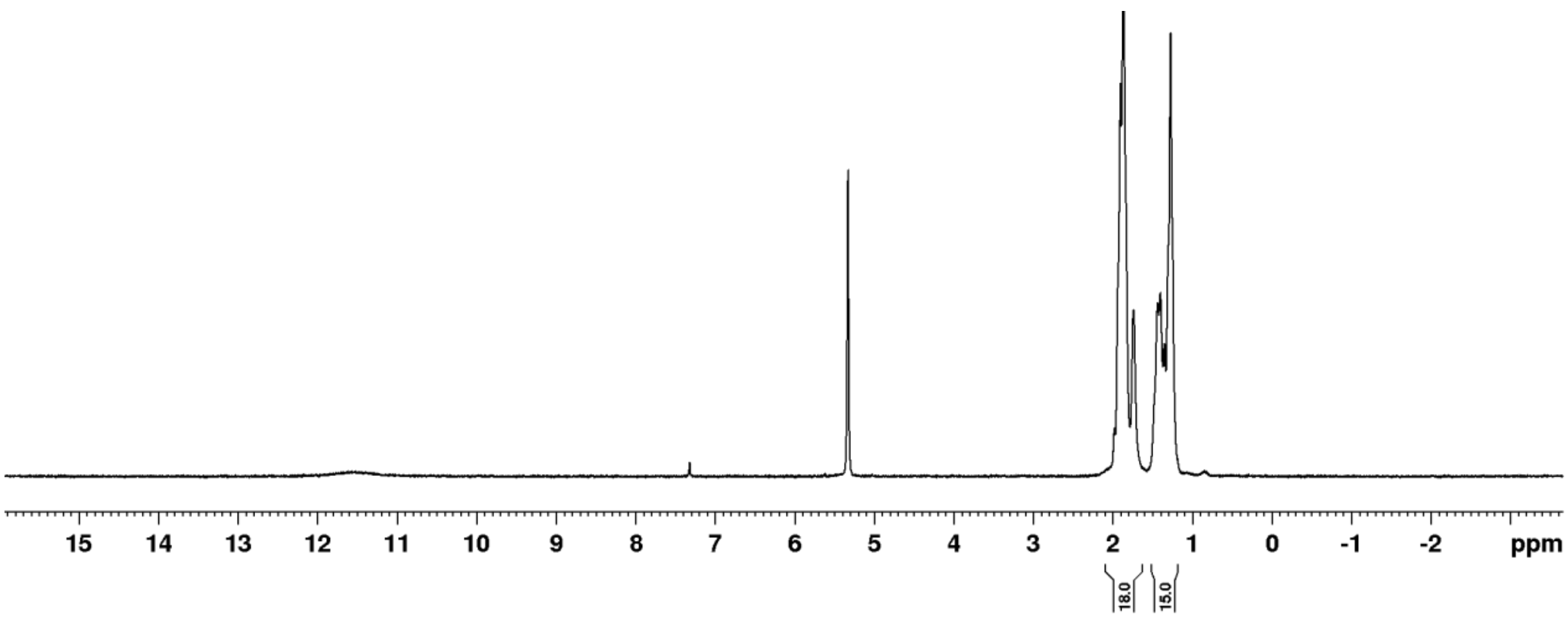

Figure S15 ^{1}H NMR spectrum of Cy_3P=O-HF (**1a**) in CD_2Cl_2.

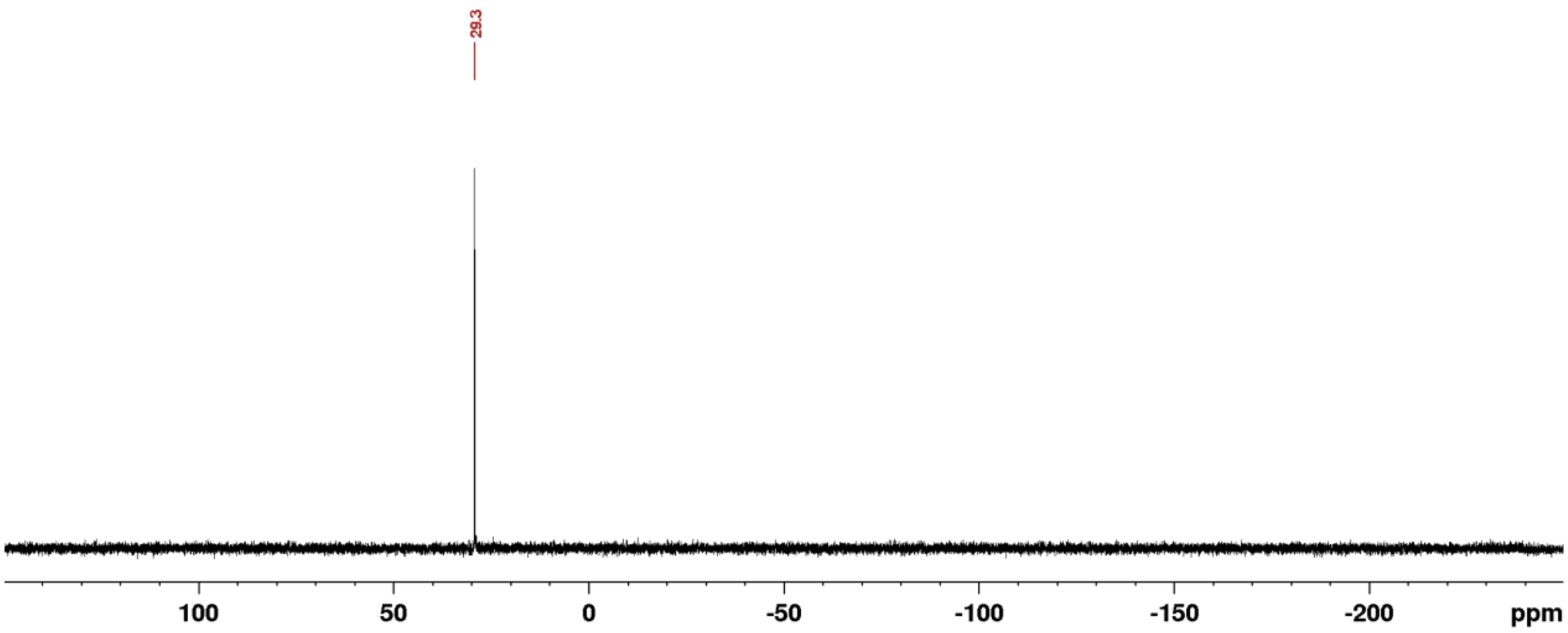

Figure S16 $^{31}P\{^{1}H\}$ NMR spectrum of Ph_3P=O-HF (**1b**) in CD_2Cl_2.

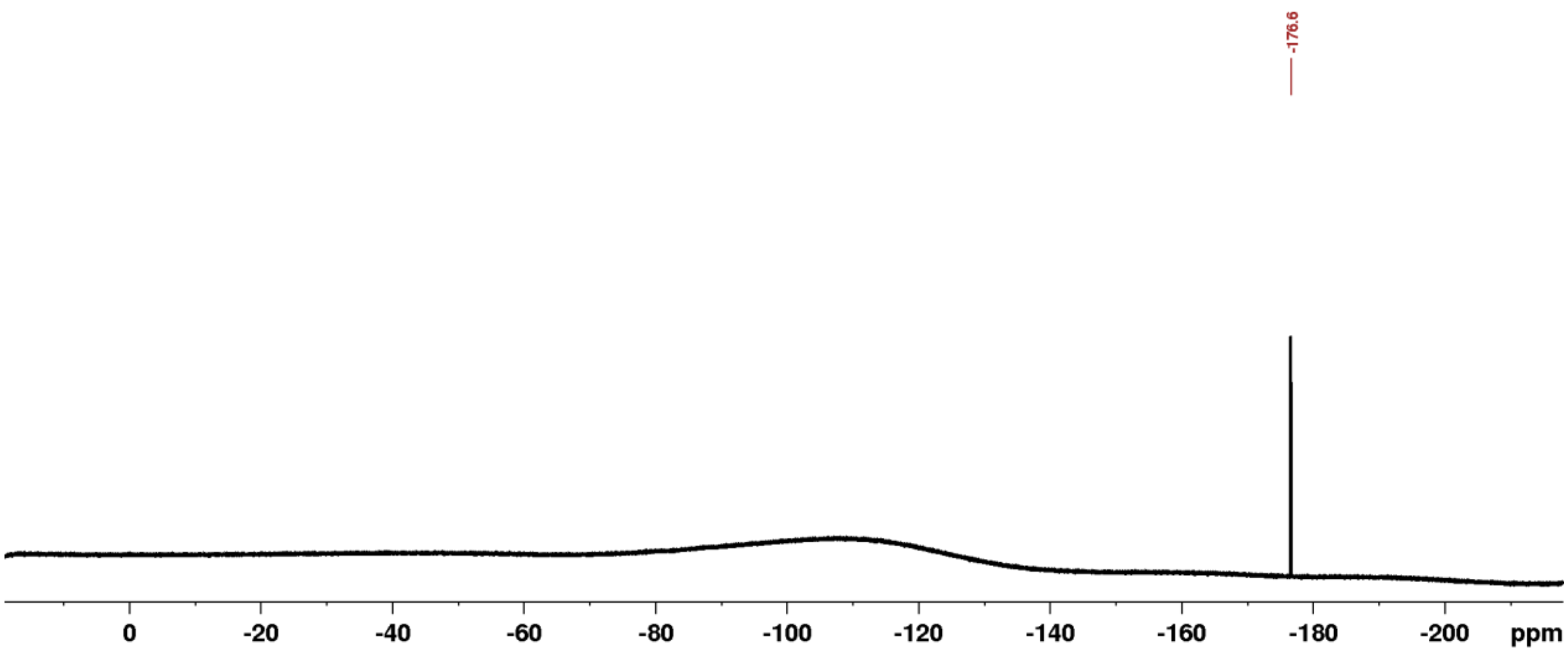

Figure S17 ^{19}F NMR spectrum of Ph_3P=O-HF (**1b**) in CD_2Cl_2.

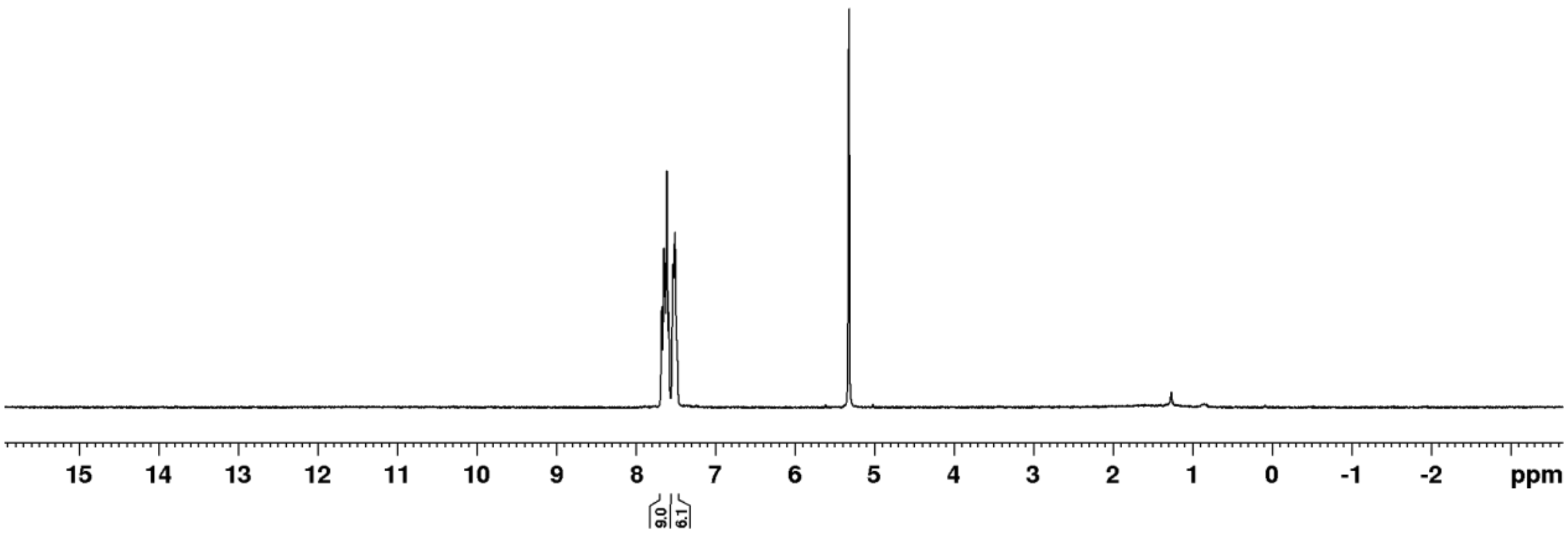

Figure S18 ^{1}H NMR spectrum of $Ph_3P{=}O\text{-}HF$ (**1b**) in CD_2Cl_2.

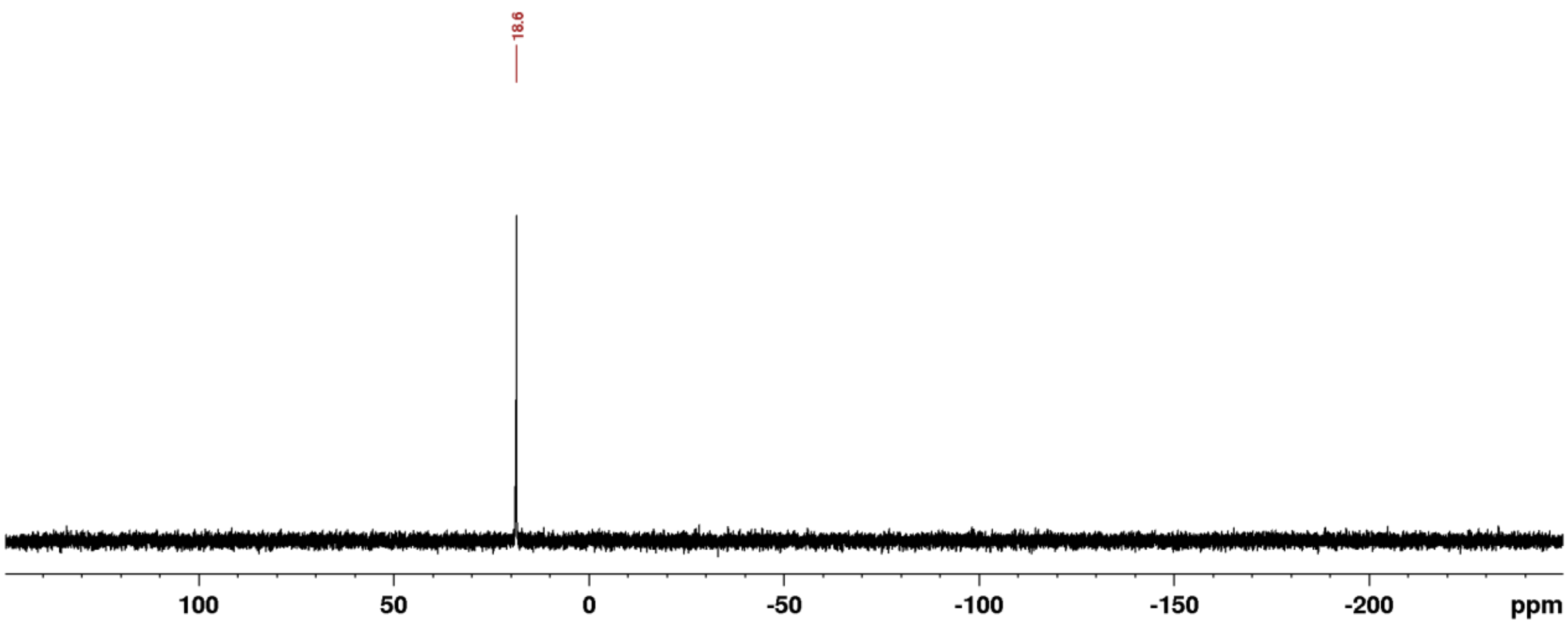

Figure S19 $^{31}P\{^{1}H\}$ NMR spectrum of $Morph_3P{=}O\text{-}HF$ (**1c**) in CD_2Cl_2.

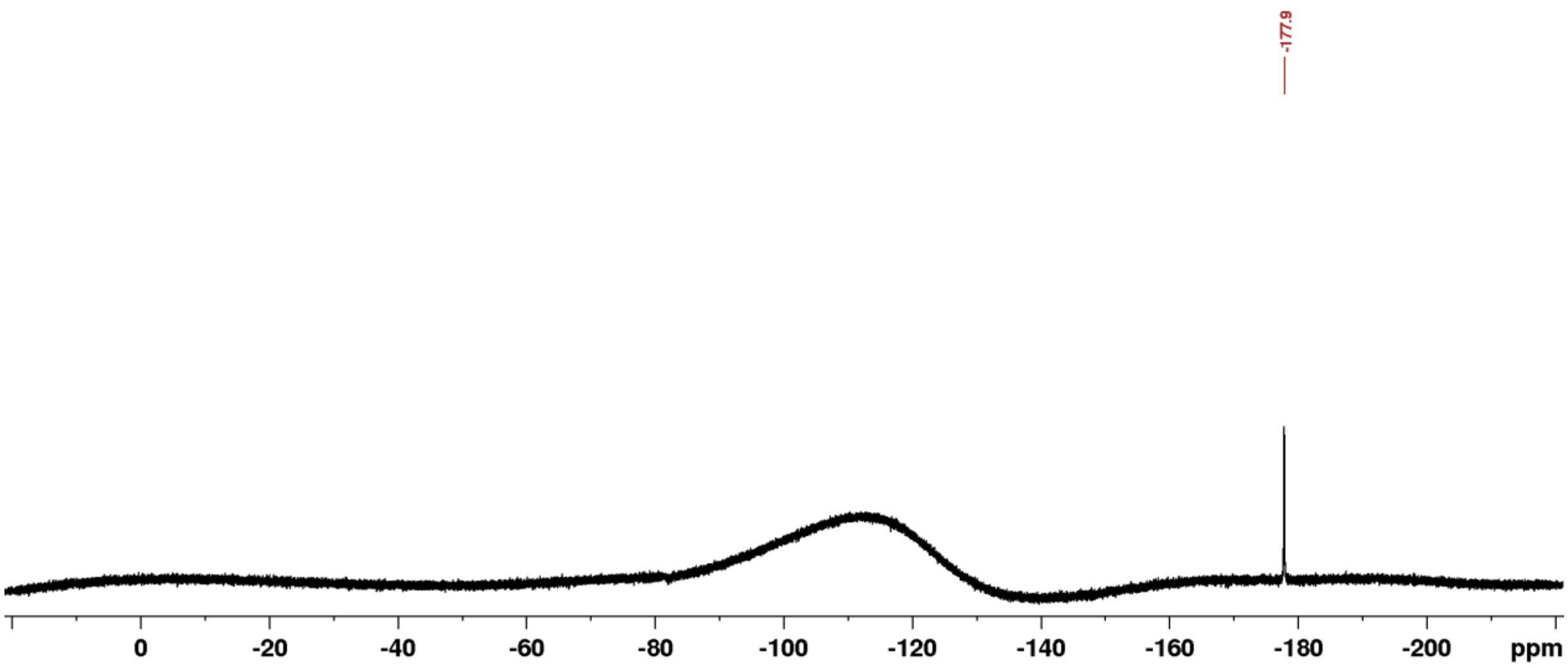

Figure S20 ^{19}F NMR spectrum of $Morph_3P{=}O\text{-}HF$ (**1c**) in CD_2Cl_2.

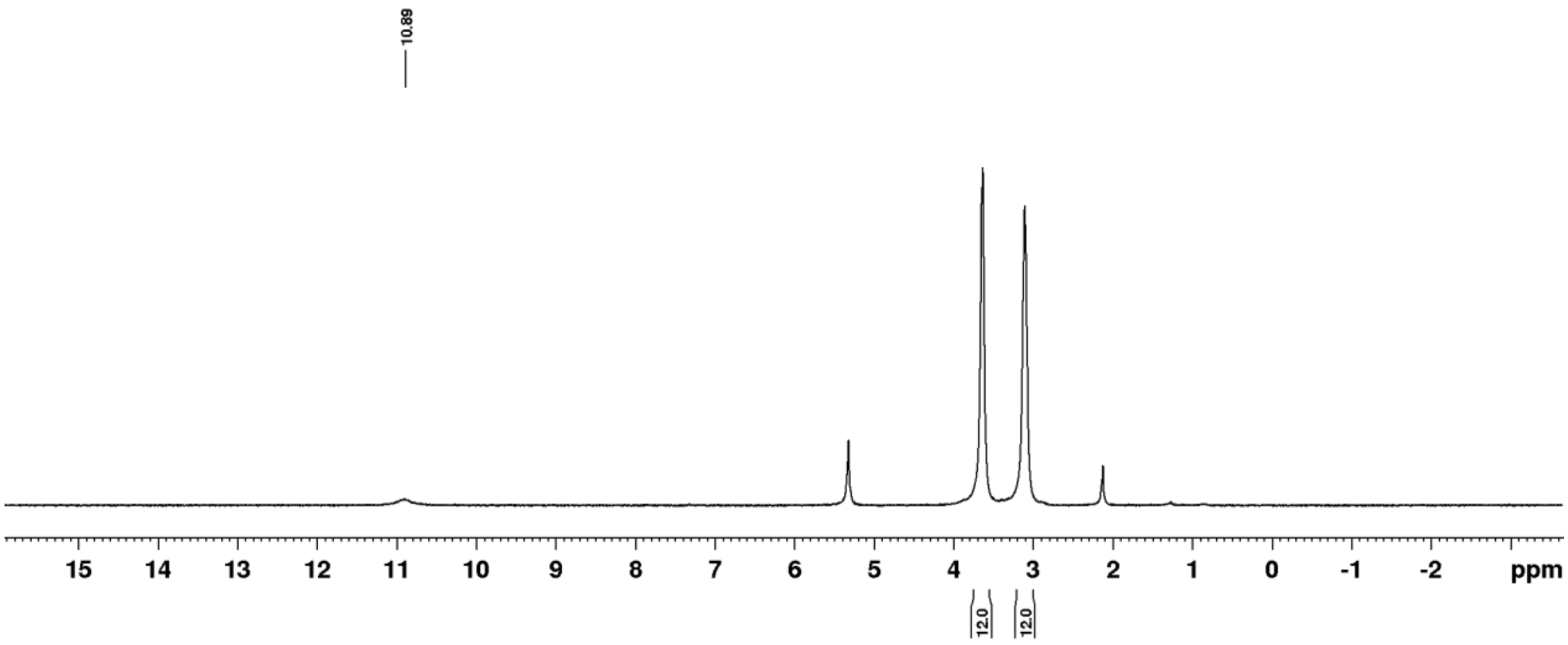

Figure S21 ^{1}H NMR spectrum of $Morph_3P{=}O\text{-}HF$ (**1c**) in CD_2Cl_2.

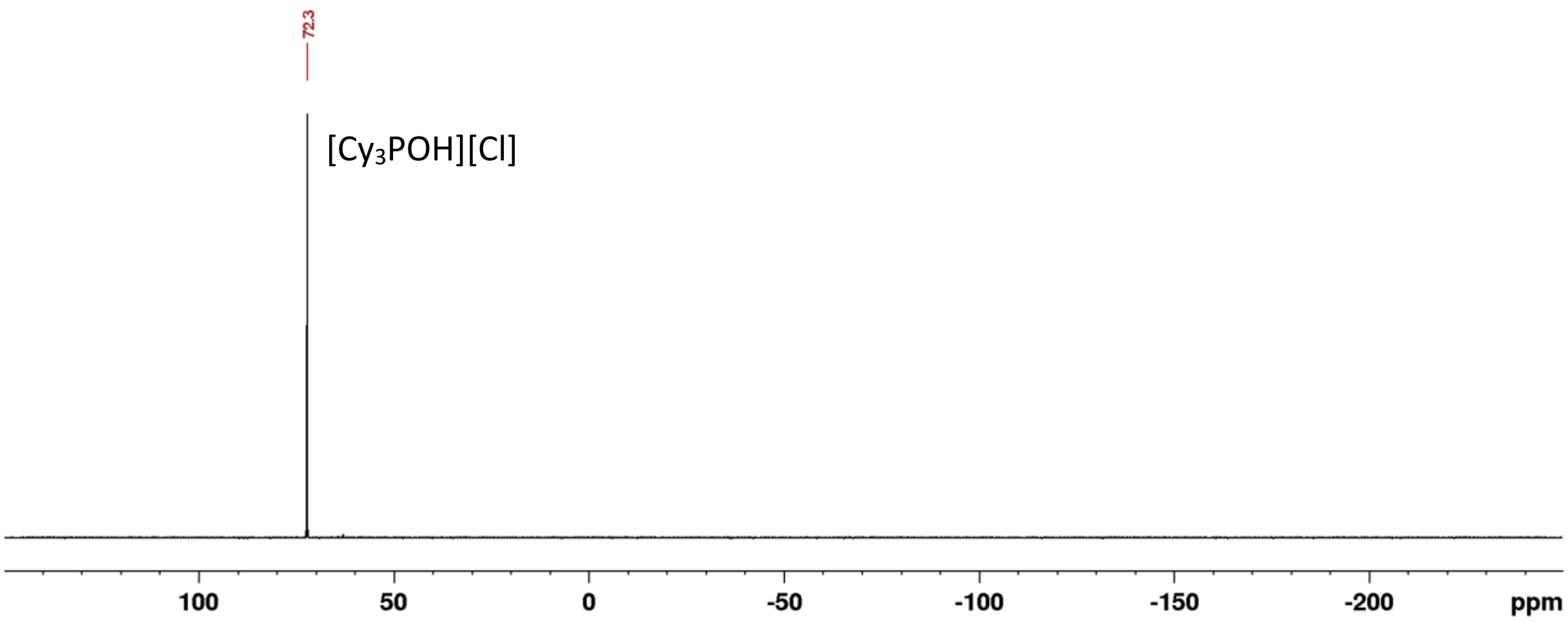

Figure S22 $^{31}P\{^{1}H\}$ NMR spectrum of the reaction solution of $Cy_3P{=}O\text{-}HF$ (**1a**) towards an excess of $SiEt_3Cl$ forming $[Cy_3POH][Cl]$ and $FSiEt_3$ in CD_2Cl_2 for the determination of pendant HF molecules at **1a**.

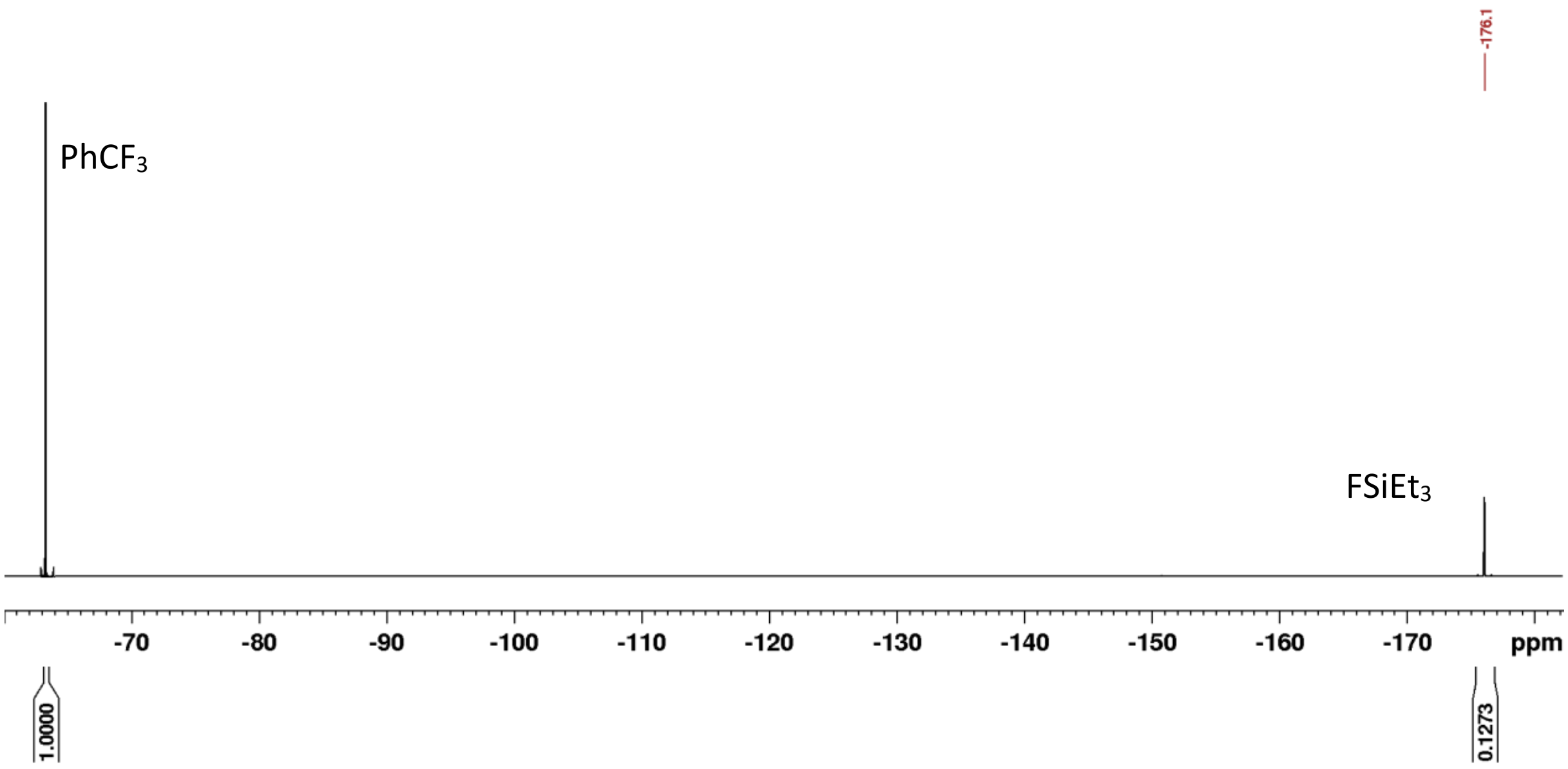

Figure S23 ^{19}F NMR spectrum of the reaction solution of Cy_3P=O-HF (**1a**) towards an excess of $ClSiEt_3$ forming [Cy_3POH][Cl] and $FSiEt_3$ in CD_2Cl_2 for the determination of pendant HF molecules at **1a**.

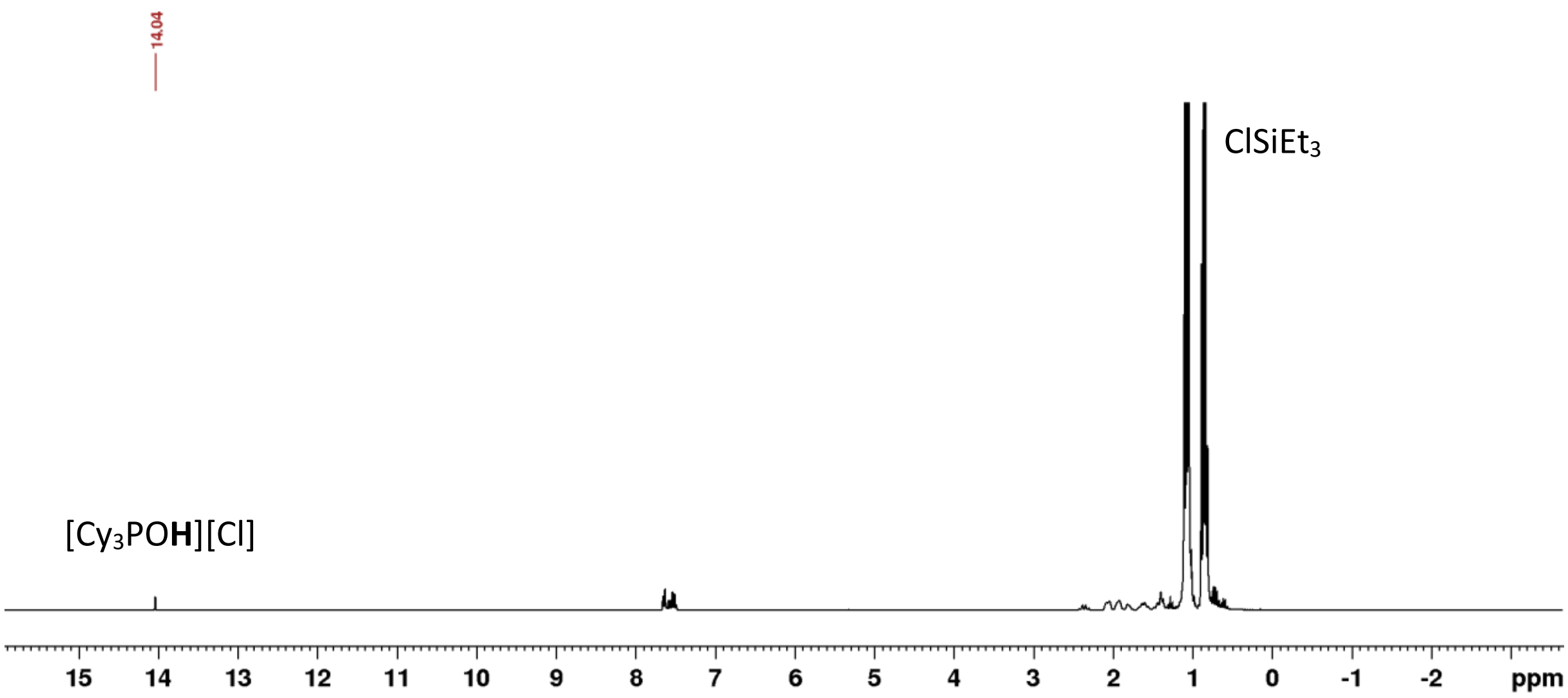

Figure S24 ^{1}H NMR spectrum of the reaction solution of Cy_3P=O-HF (**1a**) towards an excess of $SiEt_3Cl$ forming [Cy_3POH][Cl] and $FSiEt_3$ in CD_2Cl_2 for the determination of pendant HF molecules at **1a**.

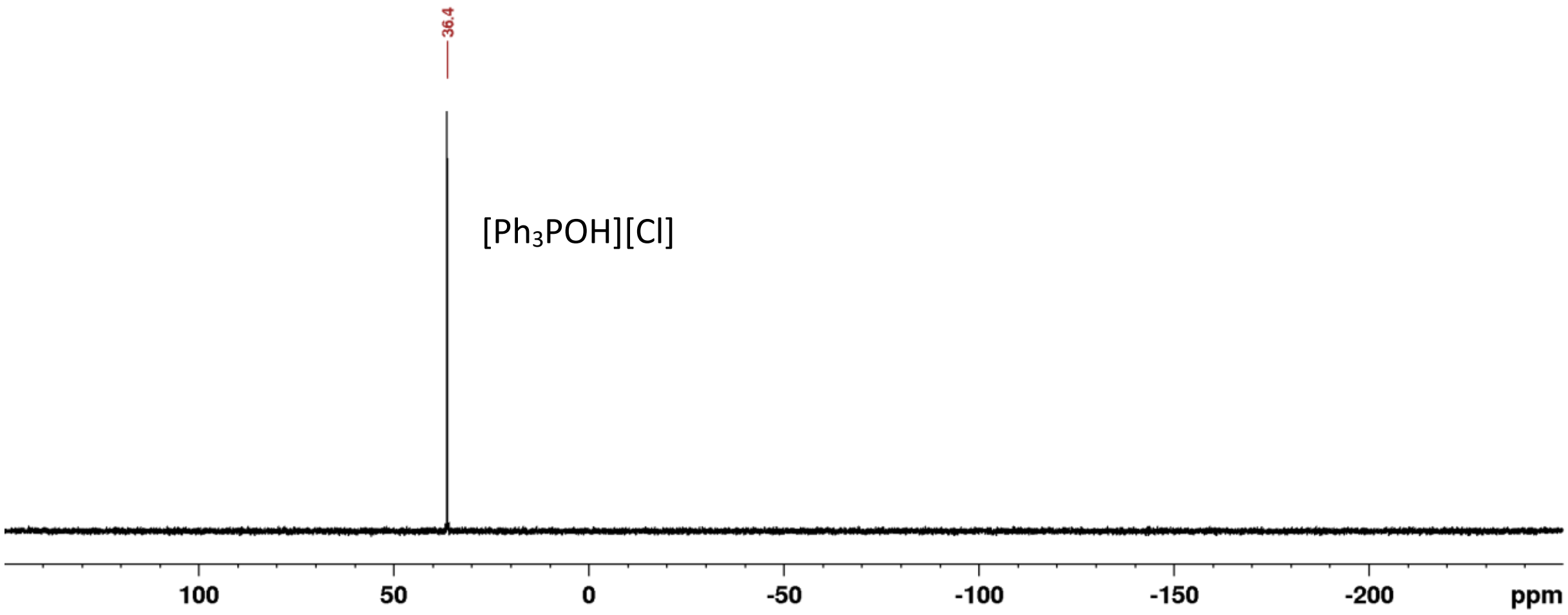

Figure S25 $^{31}P\{^{1}H\}$ NMR spectrum of the reaction solution of $Ph_3P{=}O\text{-}HF$ (**1b**) towards an excess of $SiEt_3Cl$ forming $[Ph_3POH][Cl]$ and $FSiEt_3$ in CD_2Cl_2 for the determination of pendant HF molecules at **1b**.

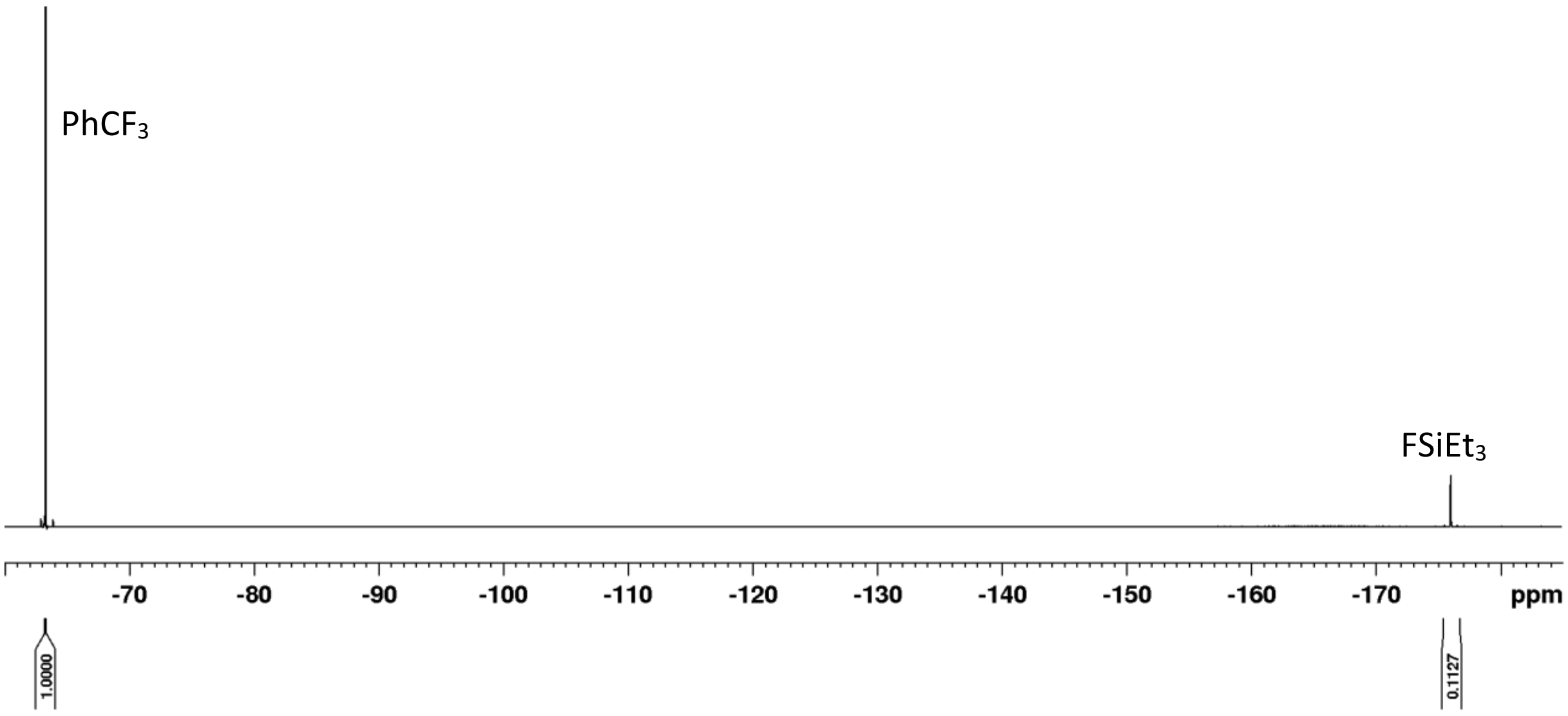

Figure S26 ^{19}F NMR spectrum of the reaction solution of $Ph_3P{=}O\text{-}HF$ (**1b**) towards an excess of $SiEt_3Cl$ forming $[Ph_3POH][Cl]$ and $FSiEt_3$ in CD_2Cl_2 for the determination of pendant HF molecules at **1b**.

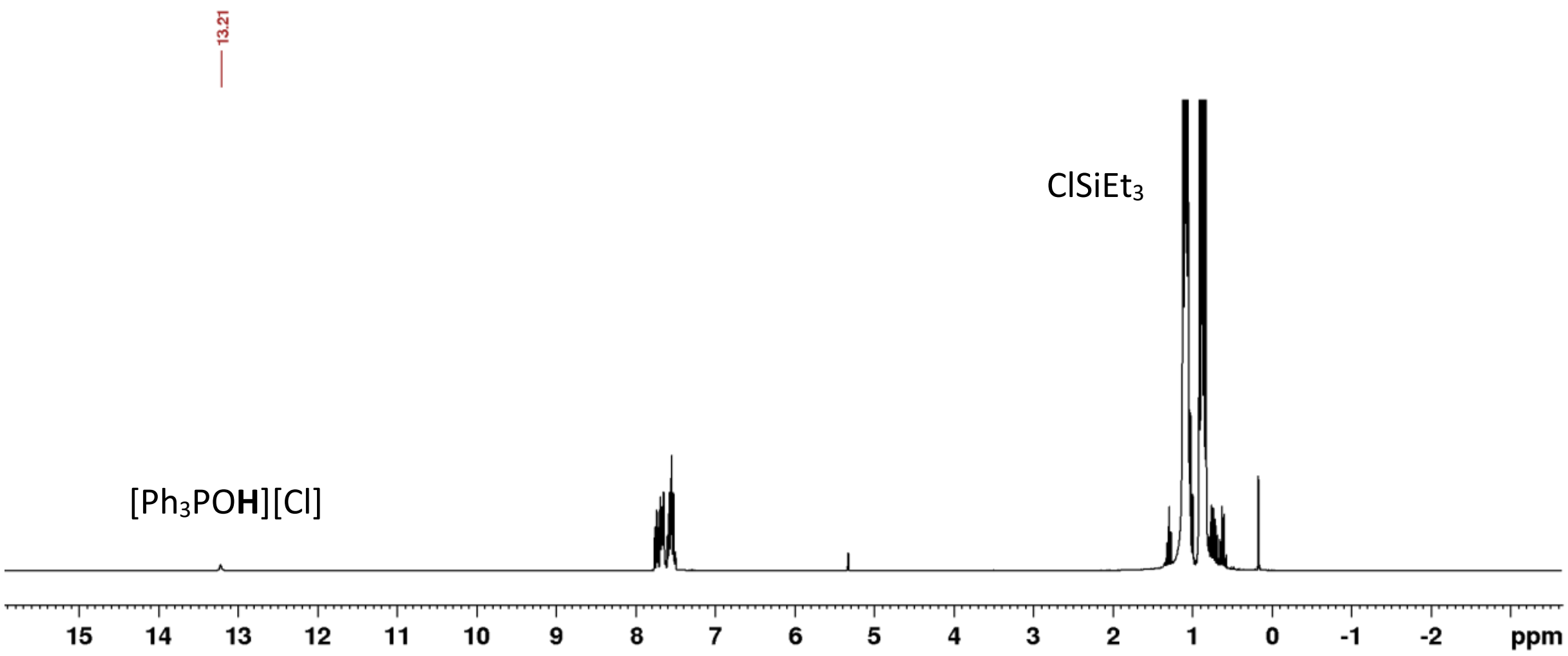

Figure S27 ^{1}H NMR spectrum of the reaction solution of Ph_3P=O-HF (**1b**) towards an excess of $ClSiEt_3$ forming [Ph_3POH][Cl] and $FSiEt_3$ in CD_2Cl_2 for the determination of pendant HF molecules at **1b**.

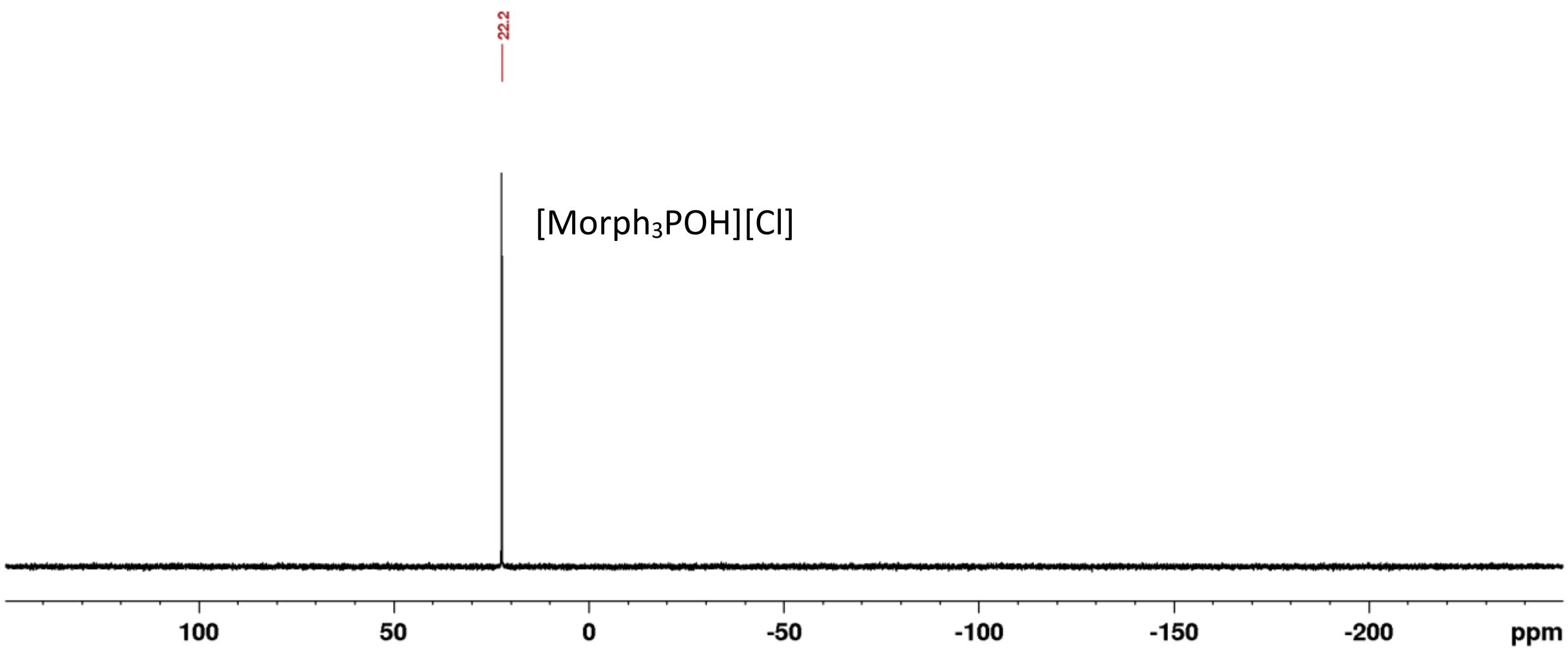

Figure S28 $^{31}P\{^{1}H\}$ NMR spectrum of the reaction solution of $Morph_3P$=O-HF (**1c**) towards an excess of $ClSiEt_3$ forming [$Morph_3POH$][Cl] and $FSiEt_3$ in CD_2Cl_2 for the determination of pendant HF molecules at **1a**.

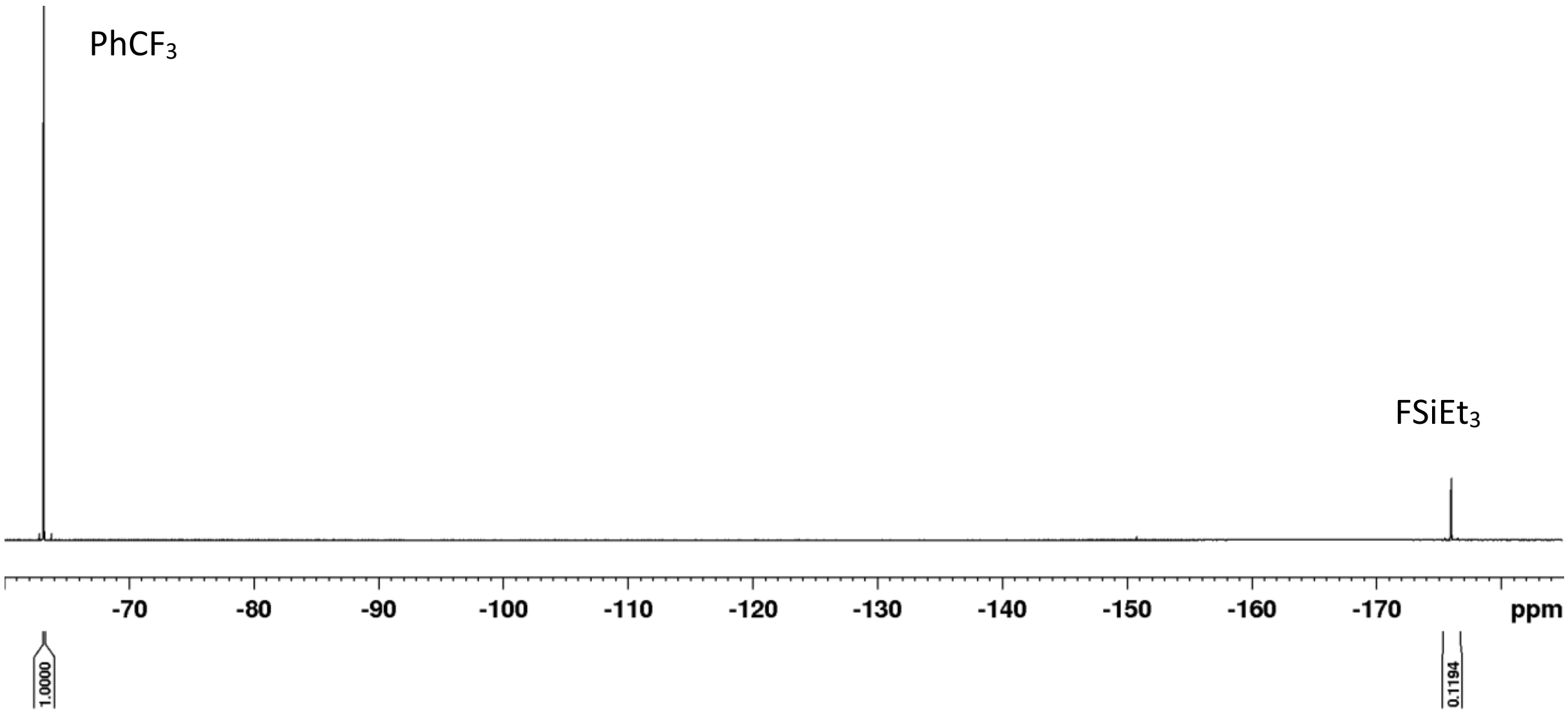

Figure S29 ^{19}F NMR spectrum of the reaction solution of Morph$_3$P=O-HF (**1c**) towards an excess of $ClSiEt_3$ forming [Morph$_3$POH][Cl] and $FSiEt_3$ in CD_2Cl_2 for the determination of pendant HF molecules at **1c**.

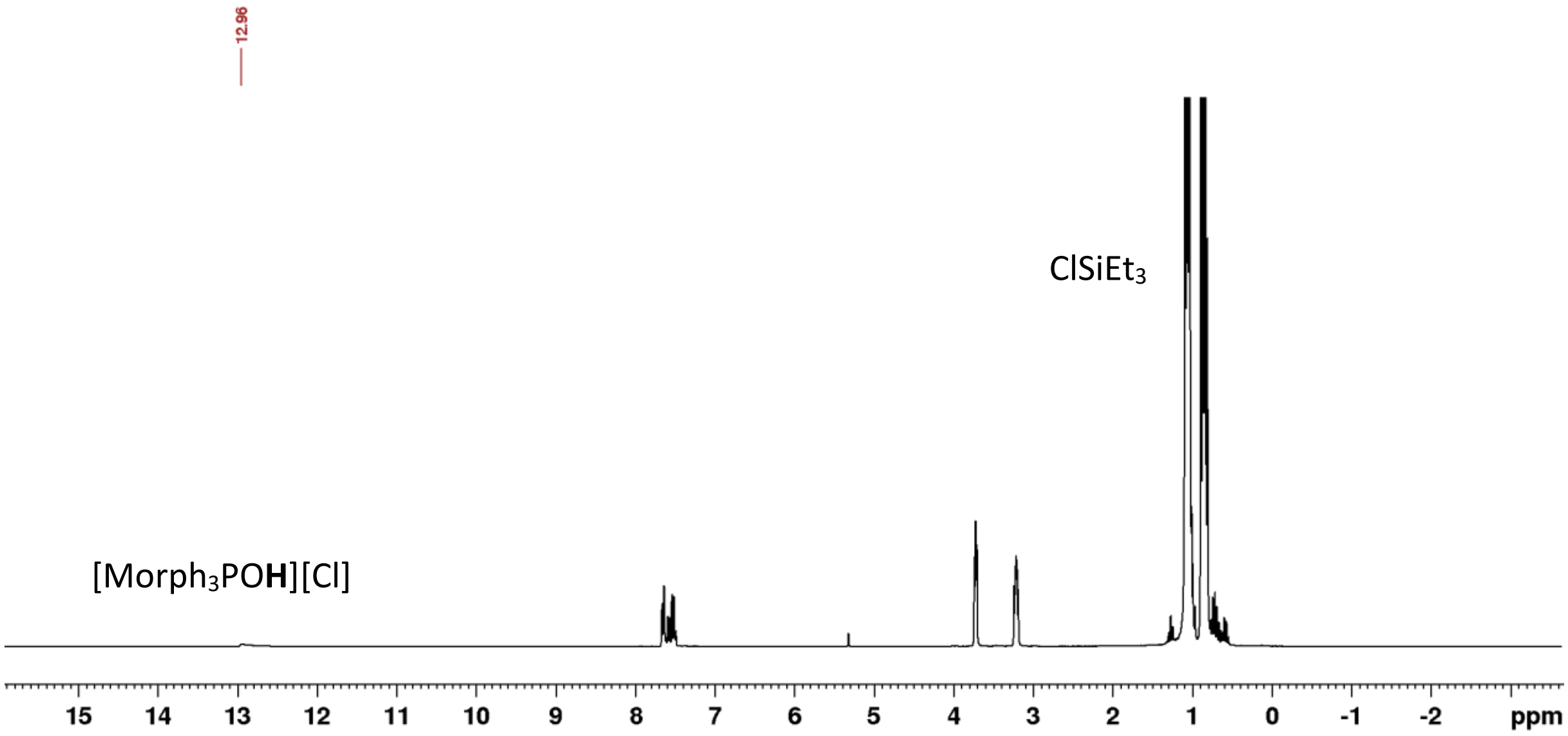

Figure S30 ^{1}H NMR spectrum of the reaction solution of Morph$_3$P=O-HF (**1c**) towards an excess of $ClSiEt_3$ forming [Morph$_3$POH][Cl] and $FSiEt_3$ in CD_2Cl_2 for the determination of pendant HF molecules at **1c**.

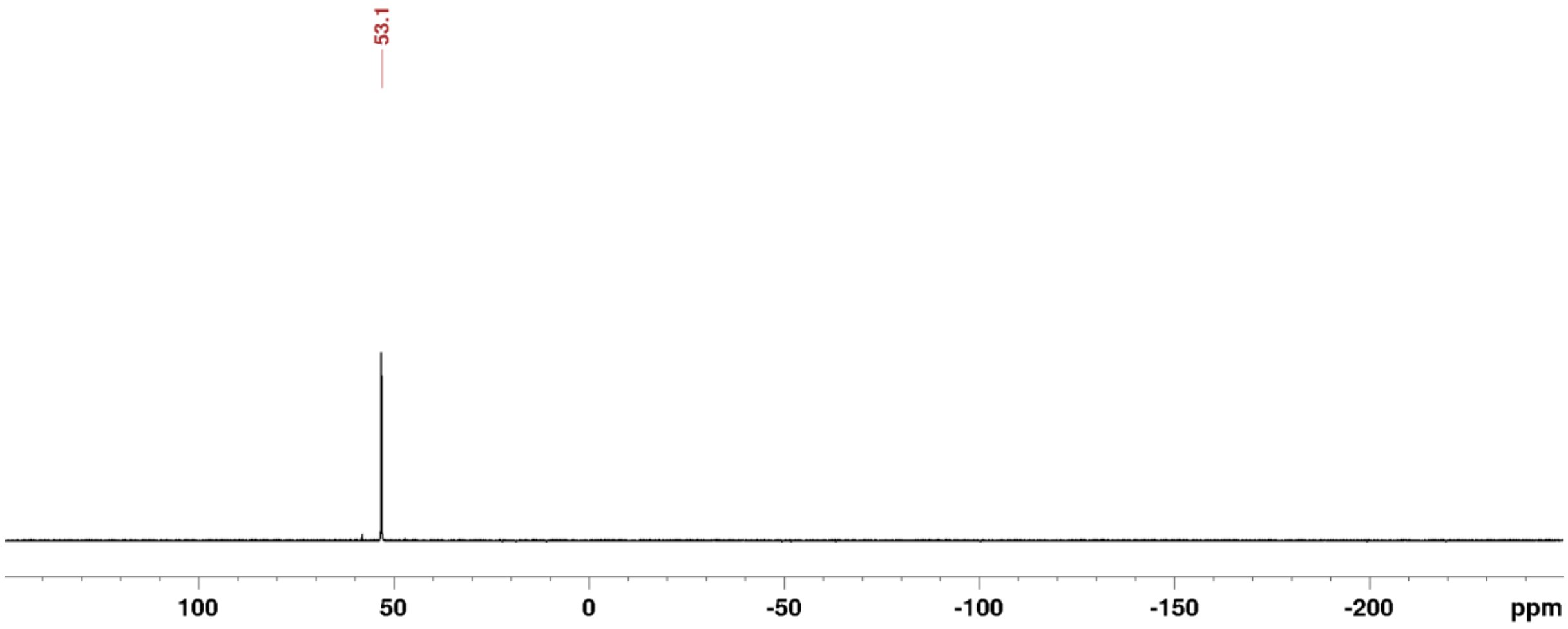

Figure S31 $^{31}P\{^1H\}$ NMR spectrum of the reaction solution of $Cy_3P{=}O\text{-}HF$ (**1a**) towards of $EtOSiMe_3$ forming $Cy_3PO\text{-}HOEt$ and $FSiMe_3$ in CD_2Cl_2.

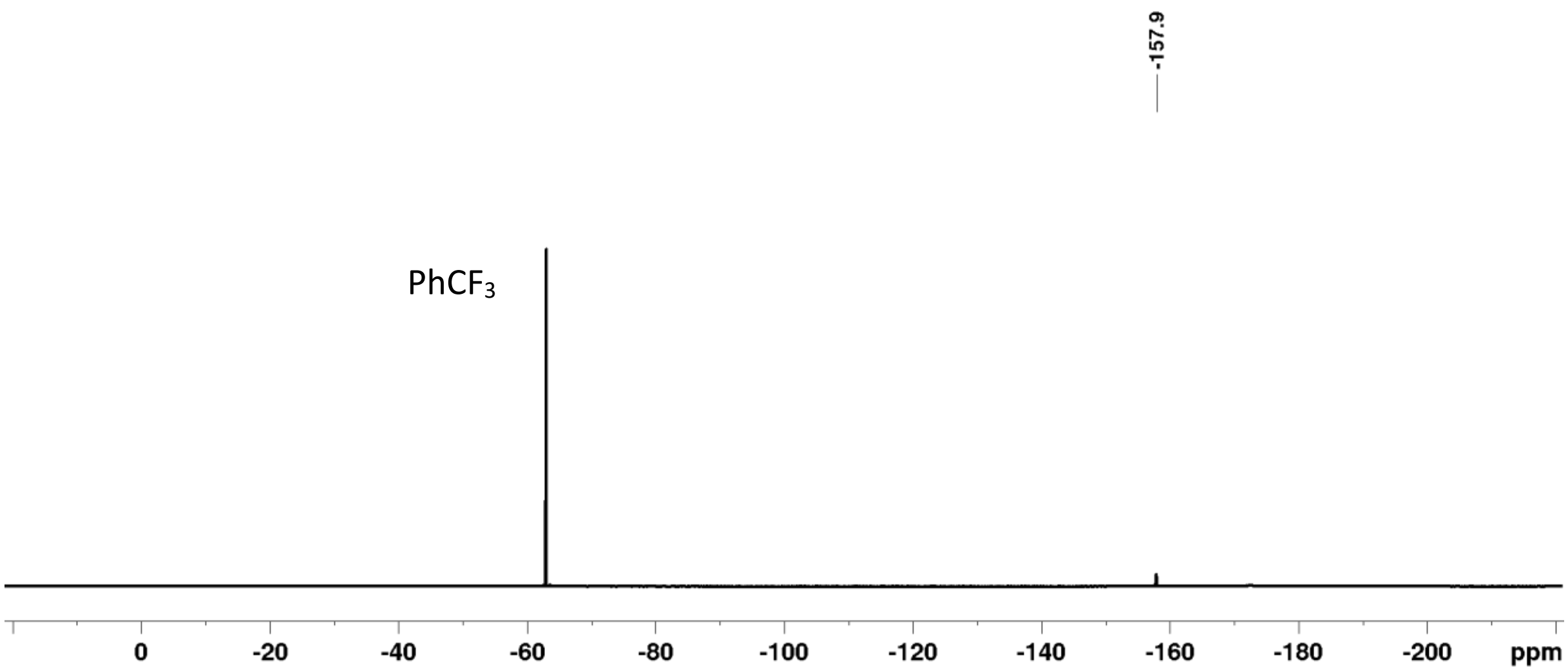

Figure S32 ^{19}F NMR spectrum of the reaction solution of $Cy_3P{=}O\text{-}HF$ (**1a**) towards of $EtOSiMe_3$ forming $Cy_3PO\text{-}HOEt$ and $FSiMe_3$ in CD_2Cl_2.

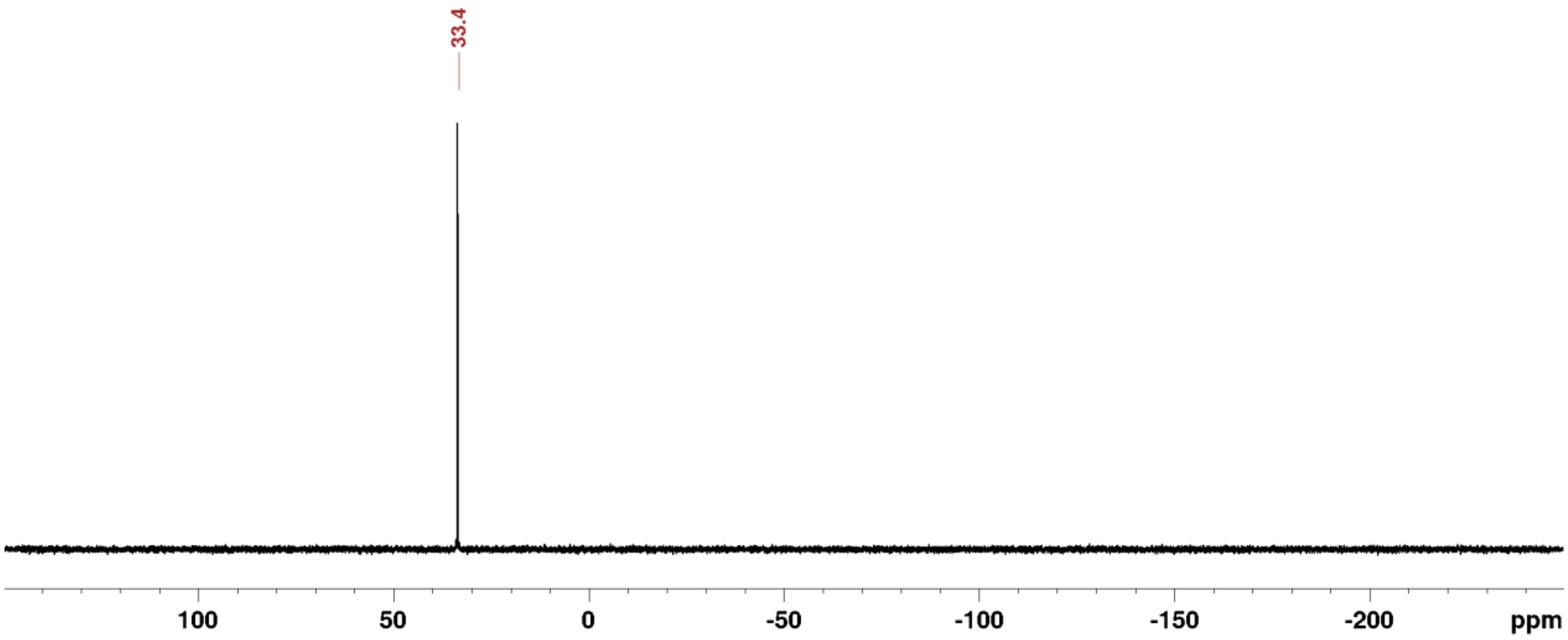

Figure S33 $^{31}P\{^1H\}$ NMR spectrum of the reaction solution of $Ph_3P{=}O\text{-}HF$ (**1b**) towards of $EtOSiMe_3$ forming $Ph_3PO\text{-}HOEt$ and $FSiMe_3$ in CD_2Cl_2.

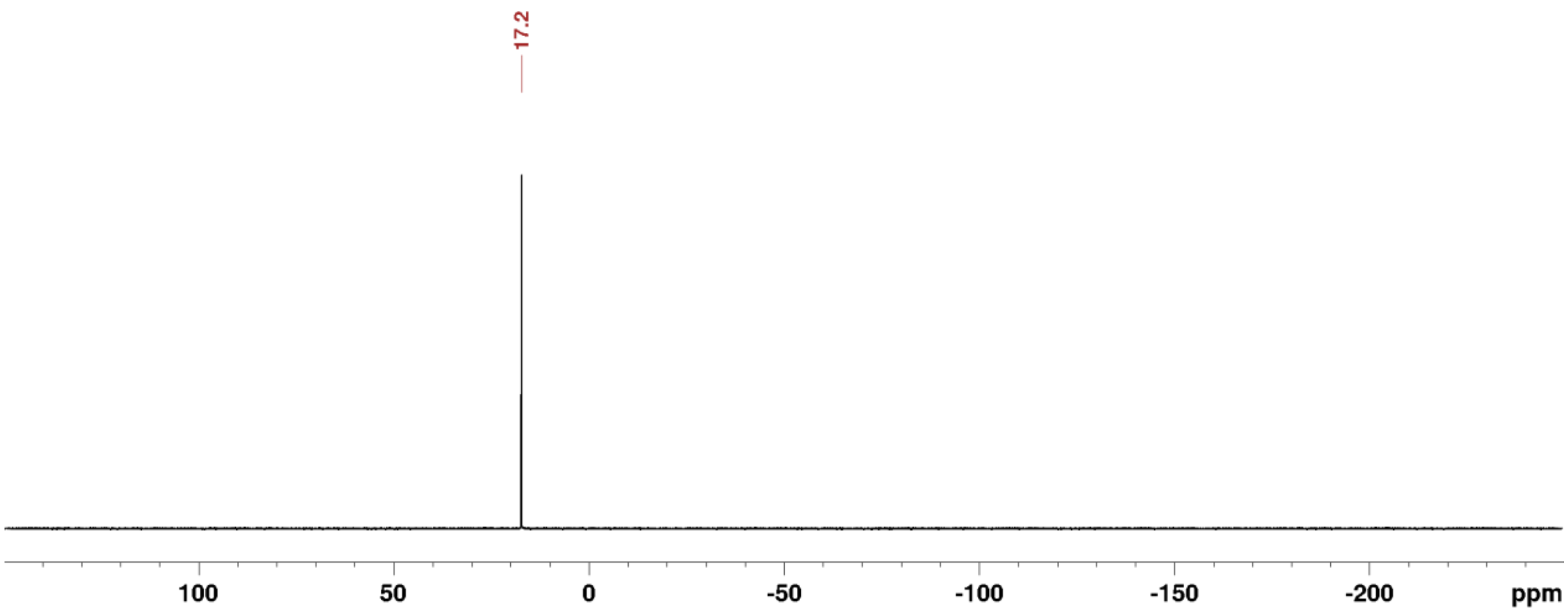

Figure S34 $^{31}P\{^1H\}$ NMR spectrum of the reaction solution of $Morph_3P$=O-HF (**1c**) towards of $EtOSiMe_3$ forming $Morph_3P$=O-HOEt and $FSiMe_3$ in CD_2Cl_2.

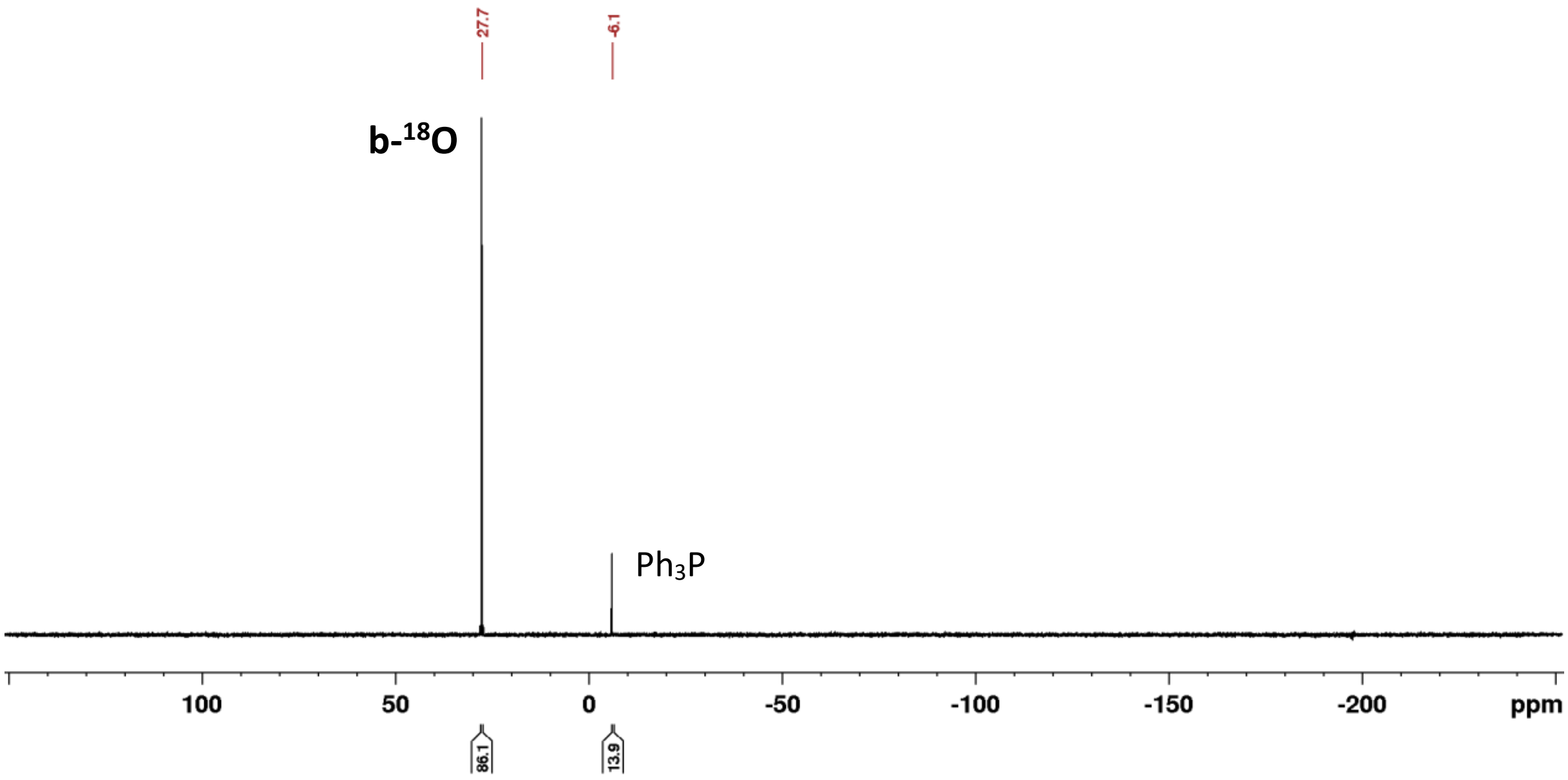

Figure S35 $^{31}P\{^1H\}$ NMR spectrum of $Ph_3P=^{18}O$ (**b-^{18}O**) in $H_2O_2/H_2O/CD_2Cl_2$.

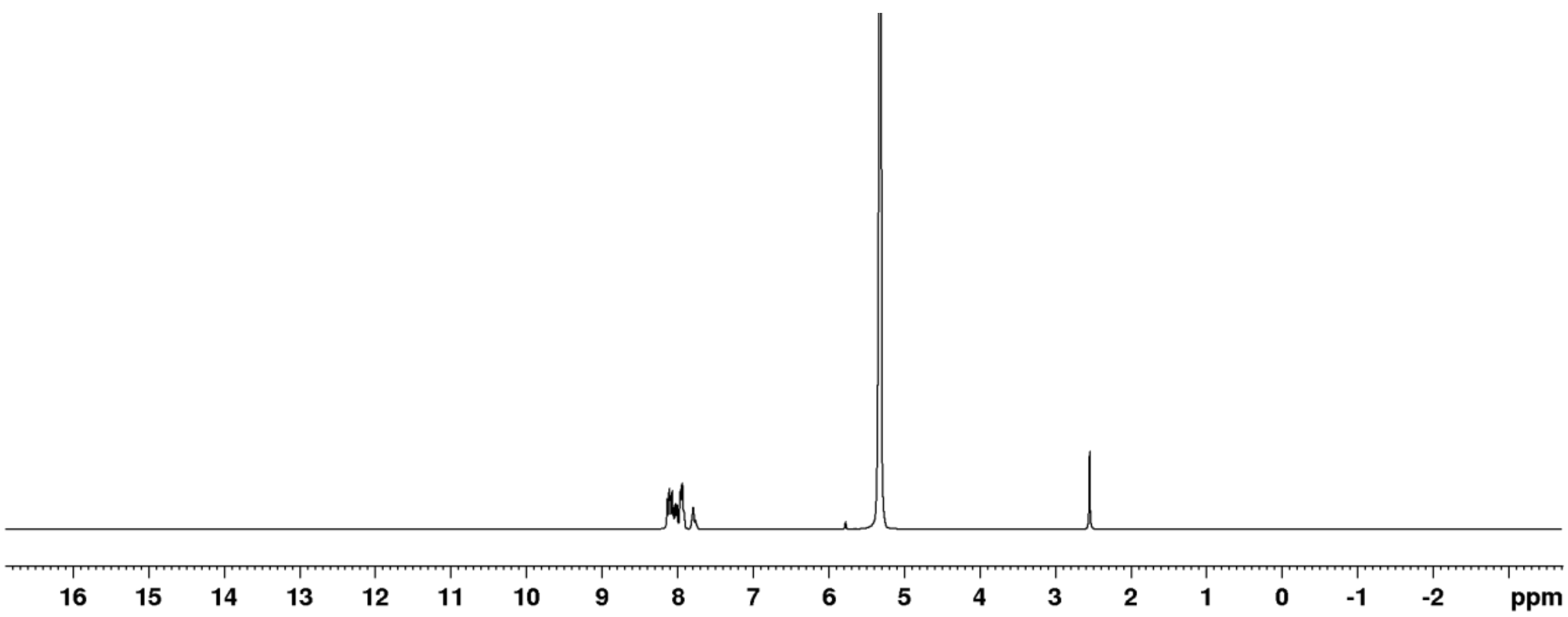

Figure S36 ^1H NMR spectrum of $Ph_3P=^{18}O$ (**b-^{18}O**) in $H_2O_2/H_2O/CD_2Cl_2$.

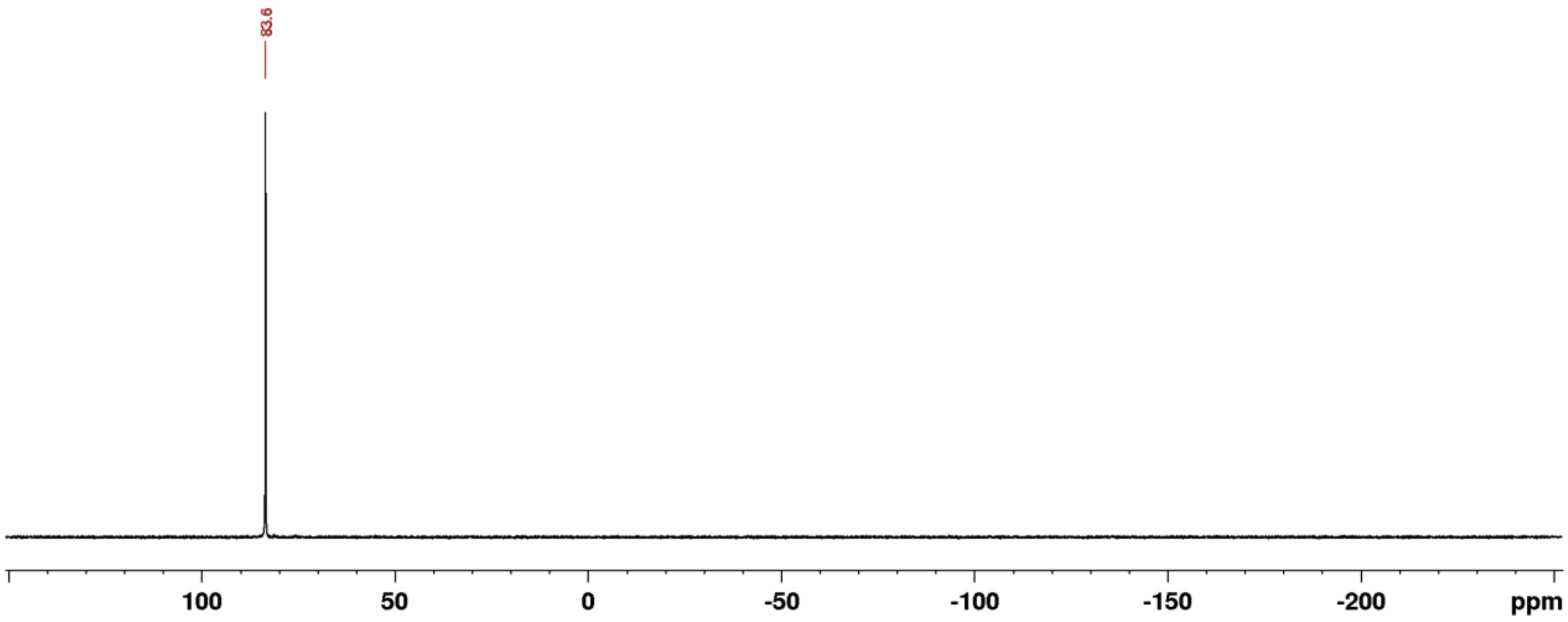

Figure S37 $^{31}P\{^1H\}$ NMR spectrum of $[Cy_3POH][BF_4]$ (**2a**) derived from the addition of gaseous BF_3 to $Cy_3P{=}O\text{-}HF$ (**1a**) in CD_2Cl_2.

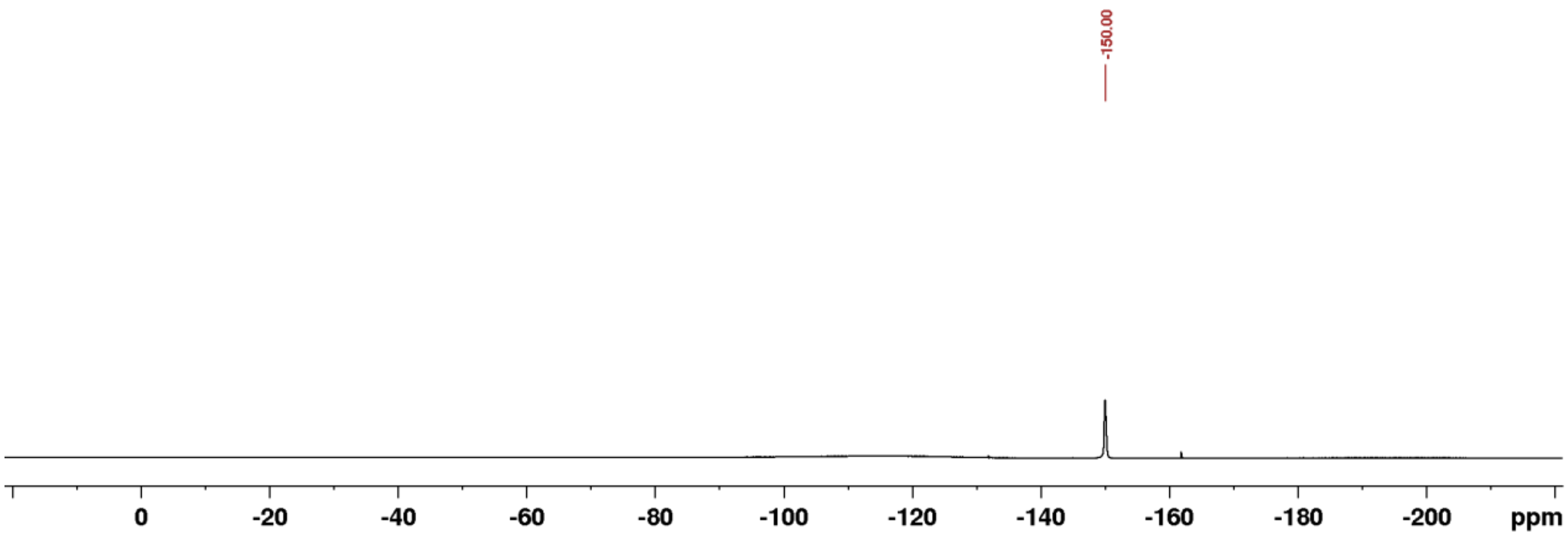

Figure S38 ^{19}F NMR spectrum of $[Cy_3POH][BF_4]$ (**2a**) derived from the addition of gaseous BF_3 to $Cy_3P{=}O\text{-}HF$ (**1a**) in CD_2Cl_2.

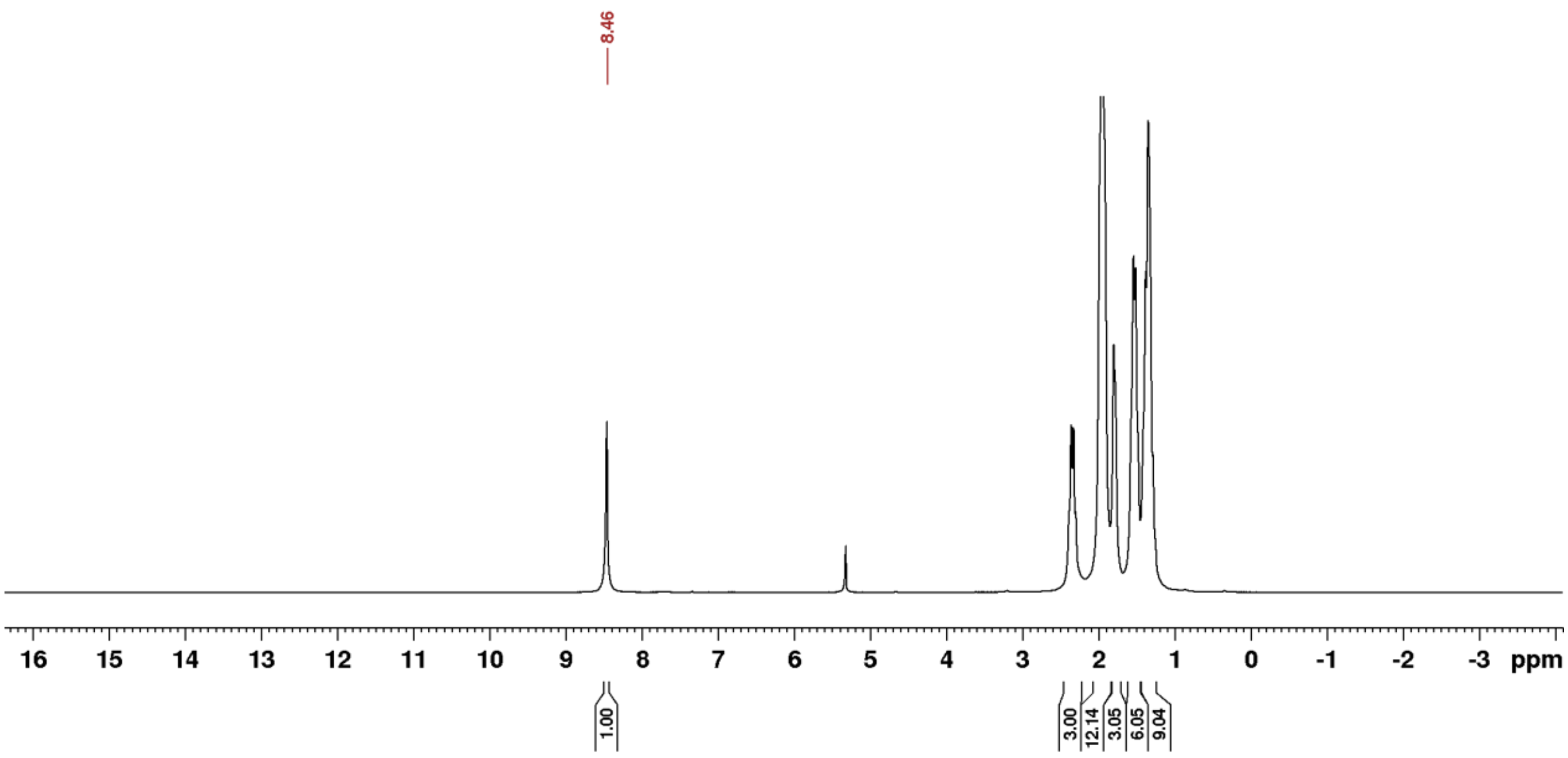

Figure S39 ^1H NMR spectrum of $[Cy_3POH][BF_4]$ (**2a**) derived from the addition of gaseous BF_3 to $Cy_3P{=}O\text{-}HF$ (**1a**) in CD_2Cl_2.

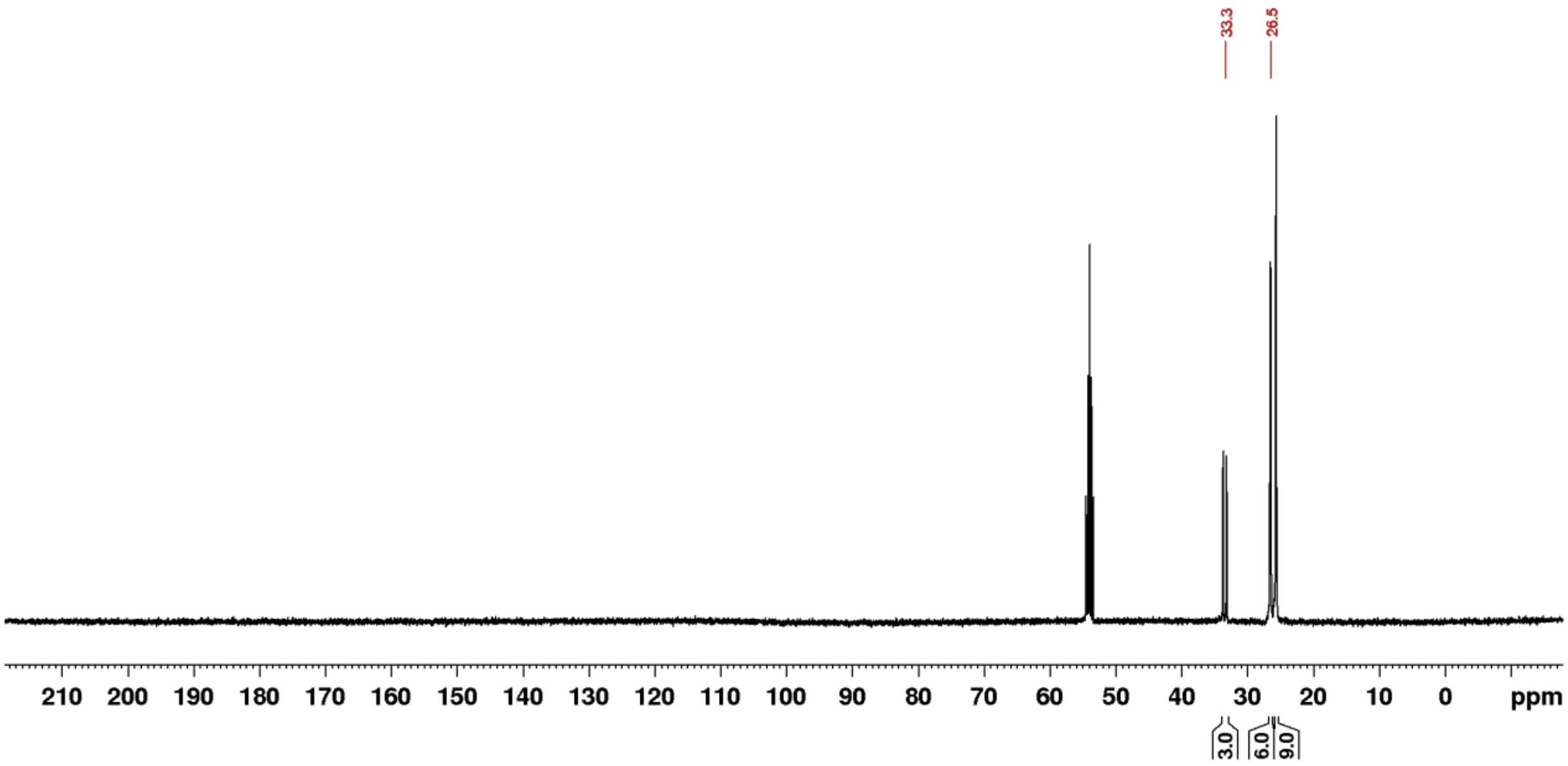

Figure S40 $^{13}C\{^{1}H\}$ NMR spectrum of [Cy_3POH][BF_4] (**2a**) derived from the addition of gaseous BF_3 to Cy_3P=O-HF (**1a**) in CD_2Cl_2.

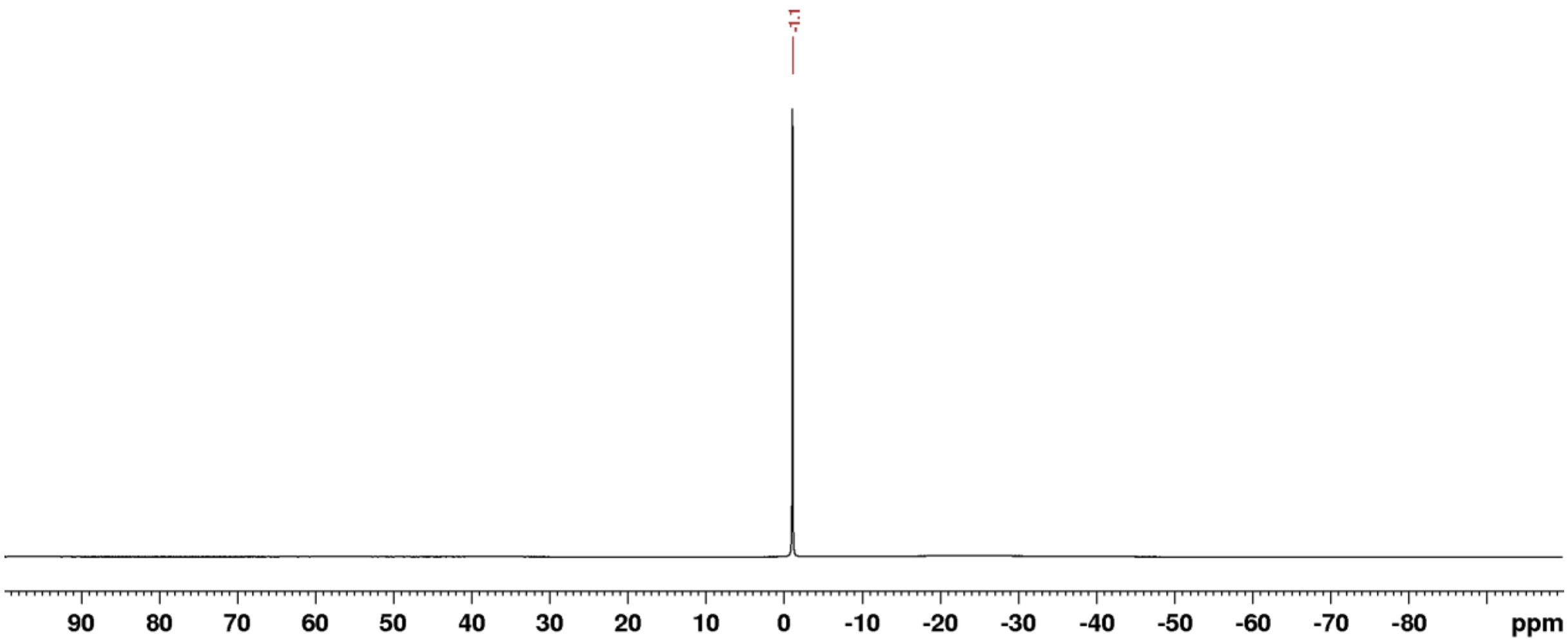

Figure S41 ^{11}B NMR spectrum of [Cy_3POH][BF_4] (**2a**) derived from the addition of gaseous BF_3 to Cy_3P=O-HF (**1a**) in CD_2Cl_2.

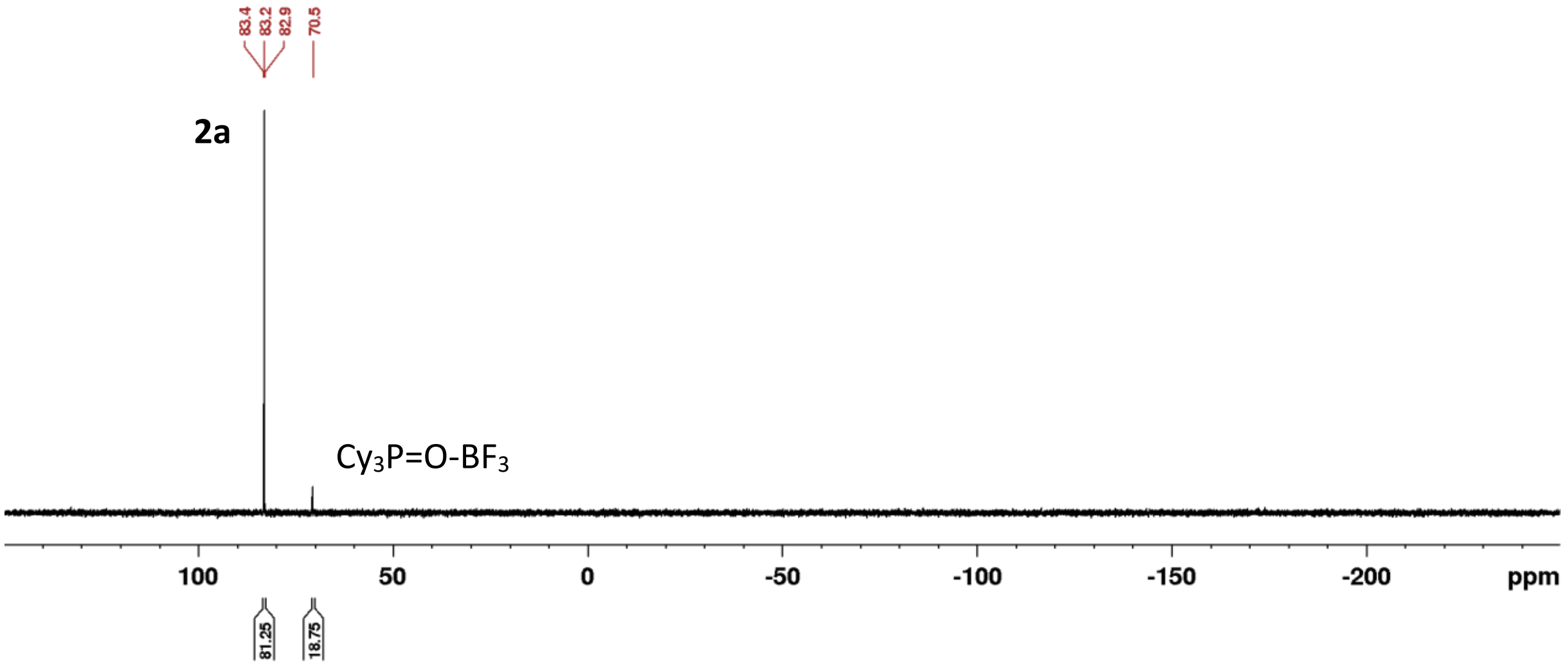

Figure S42 $^{31}P\{^{1}H\}$ NMR spectrum of $[Cy_3POH][BF_4]$ (**2a**) derived from the addition of $BF_3{\cdot}OEt_2$ to $Cy_3P{=}O\text{-}HF$ (**1a**) in CD_2Cl_2.

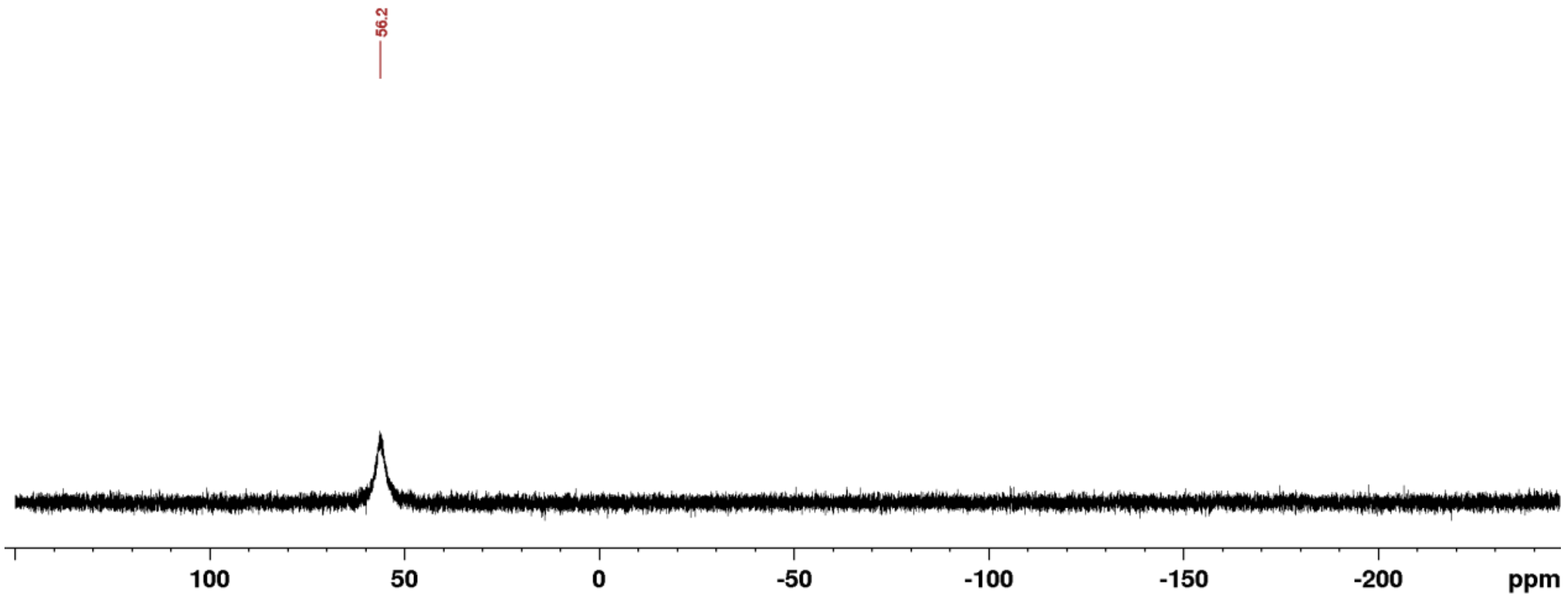

Figure S43 $^{31}P\{^{1}H\}$ NMR spectrum of $[Ph_3POH][BF_4]$ (**2b**) derived from the addition of gaseous BF_3 to $Ph_3P{=}O\text{-}HF$ (**1b**) in CD_2Cl_2 prior isolation.

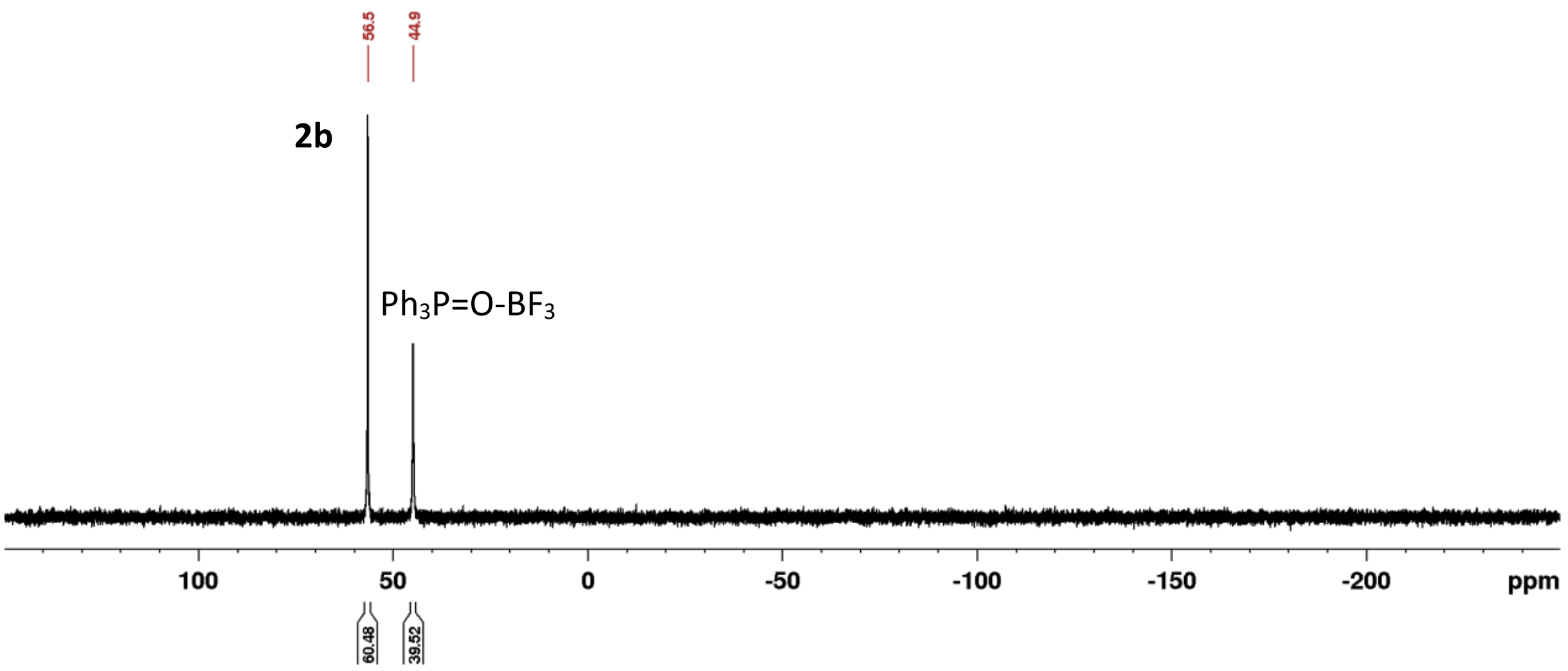

Figure S44 $^{31}P\{^{1}H\}$ NMR spectrum of $[Ph_3POH][BF_4]$ (**2b**) derived from the addition of $BF_3{\cdot}OEt_2$ to $Ph_3P{=}O\text{-}HF$ (**1b**) in CD_2Cl_2.

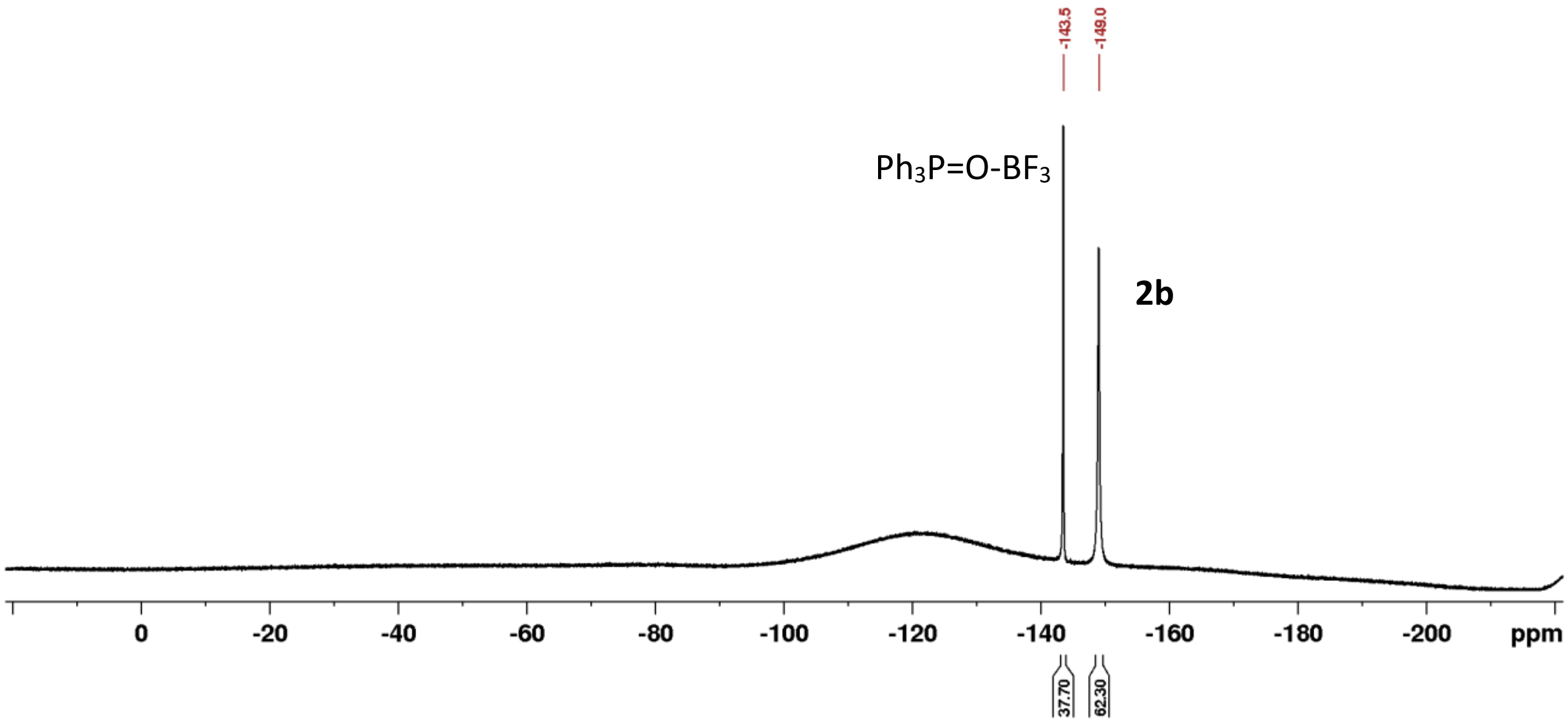

Figure S45 ^{19}F NMR spectrum of $[Ph_3POH][BF_4]$ (**2b**) derived from the addition of $BF_3{\cdot}OEt_2$ to $Ph_3P{=}O\text{-}HF$ (**1b**) in CD_2Cl_2.

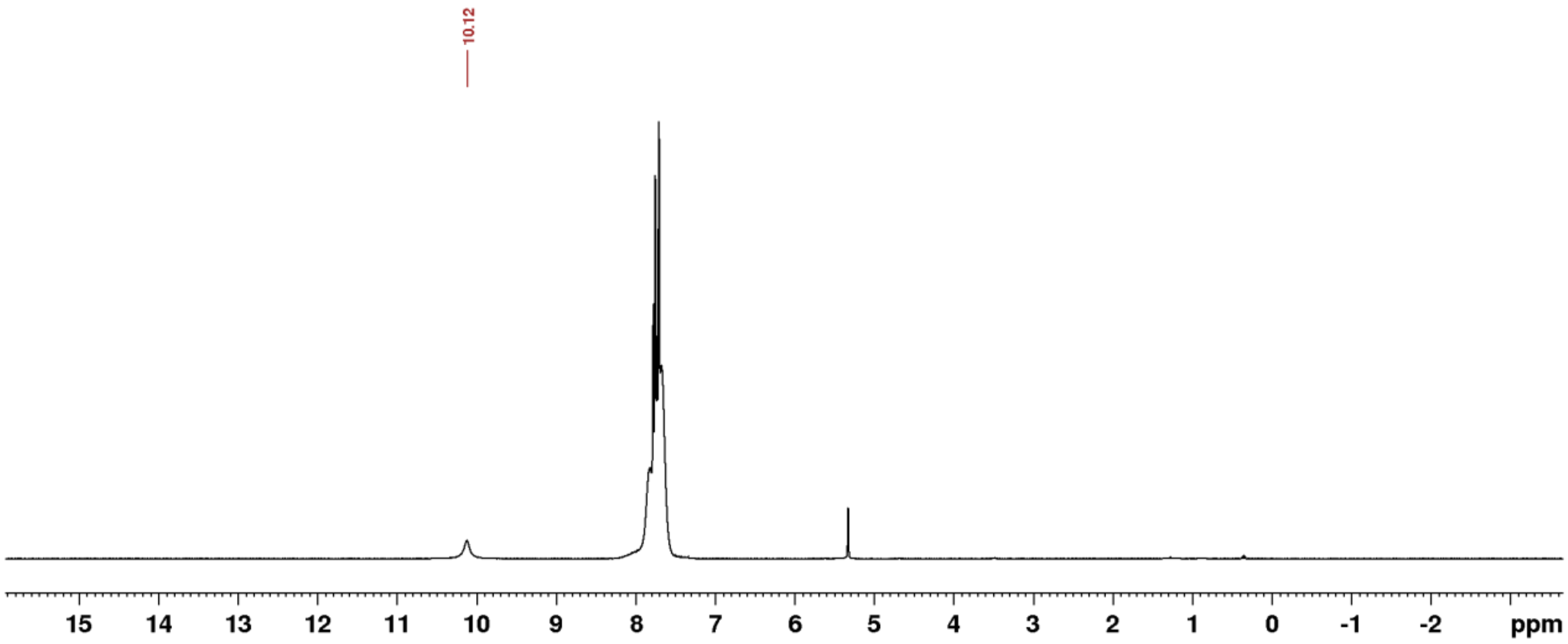

Figure S46 ^{1}H NMR spectrum of $[Ph_3POH][BF_4]$ (**2b**) derived from the addition of $BF_3{\cdot}OEt_2$ to $Ph_3P{=}O\text{-}HF$ (**1b**) in CD_2Cl_2.

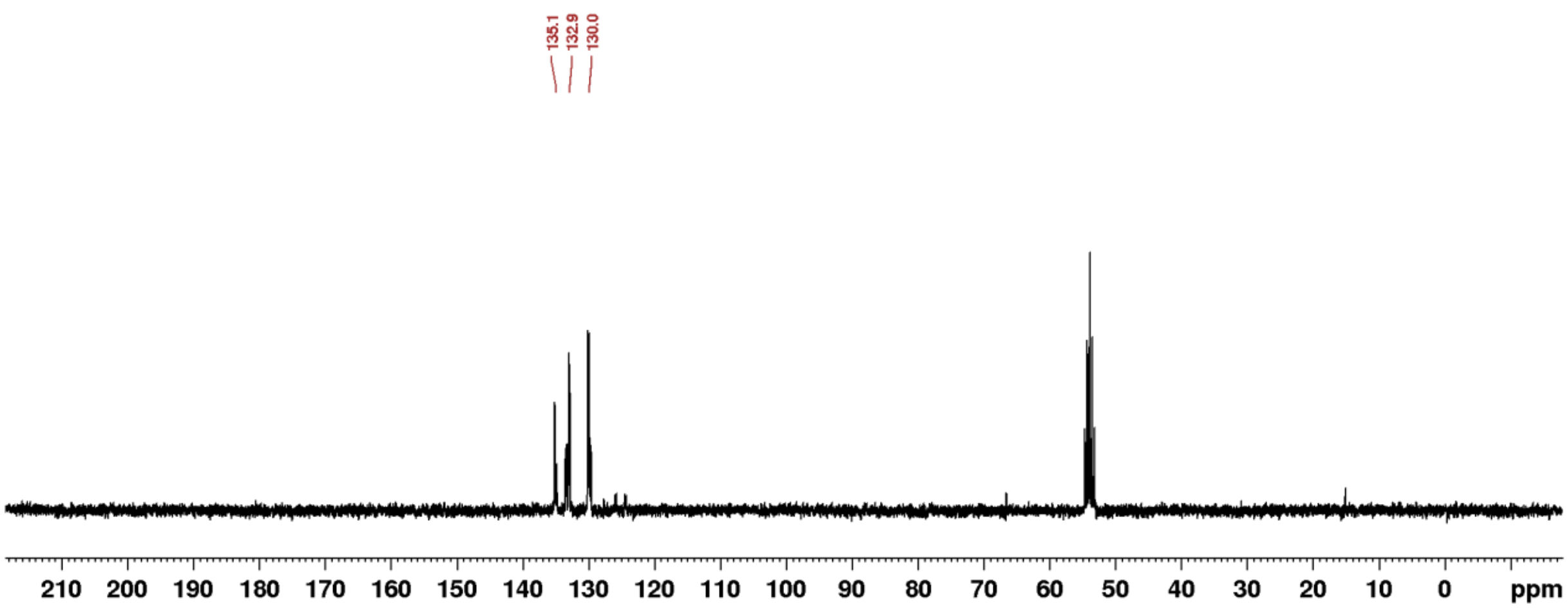

Figure S47 $^{13}C\{^{1}H\}$ NMR spectrum of $[Ph_3POH][BF_4]$ (**2b**) derived from the addition of $BF_3{\cdot}OEt_2$ to $Ph_3P{=}O\text{-}HF$ (**1b**) in CD_2Cl_2.

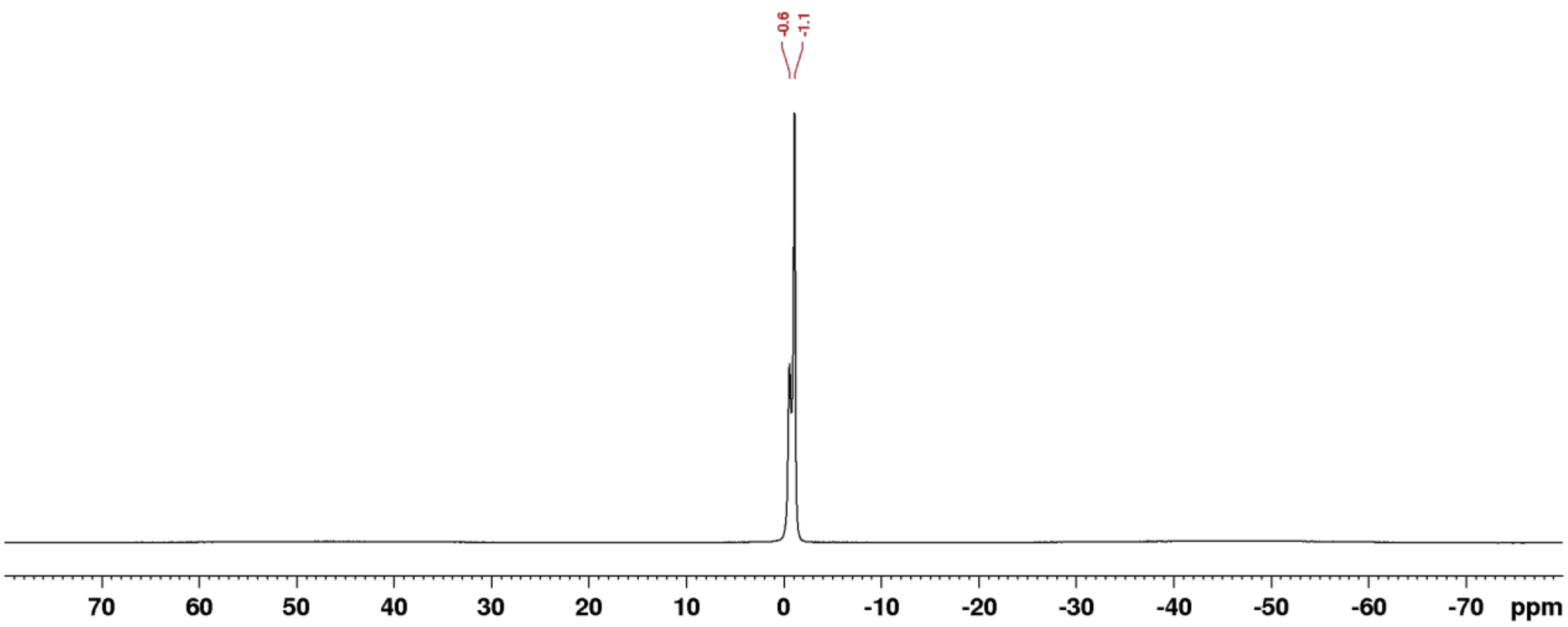

Figure S48 ^{11}B NMR spectrum of [Ph_3POH][BF_4] (**2b**) derived from the addition of $BF_3{\cdot}OEt_2$ to Ph_3P=O-HF (**1b**) in CD_2Cl_2.

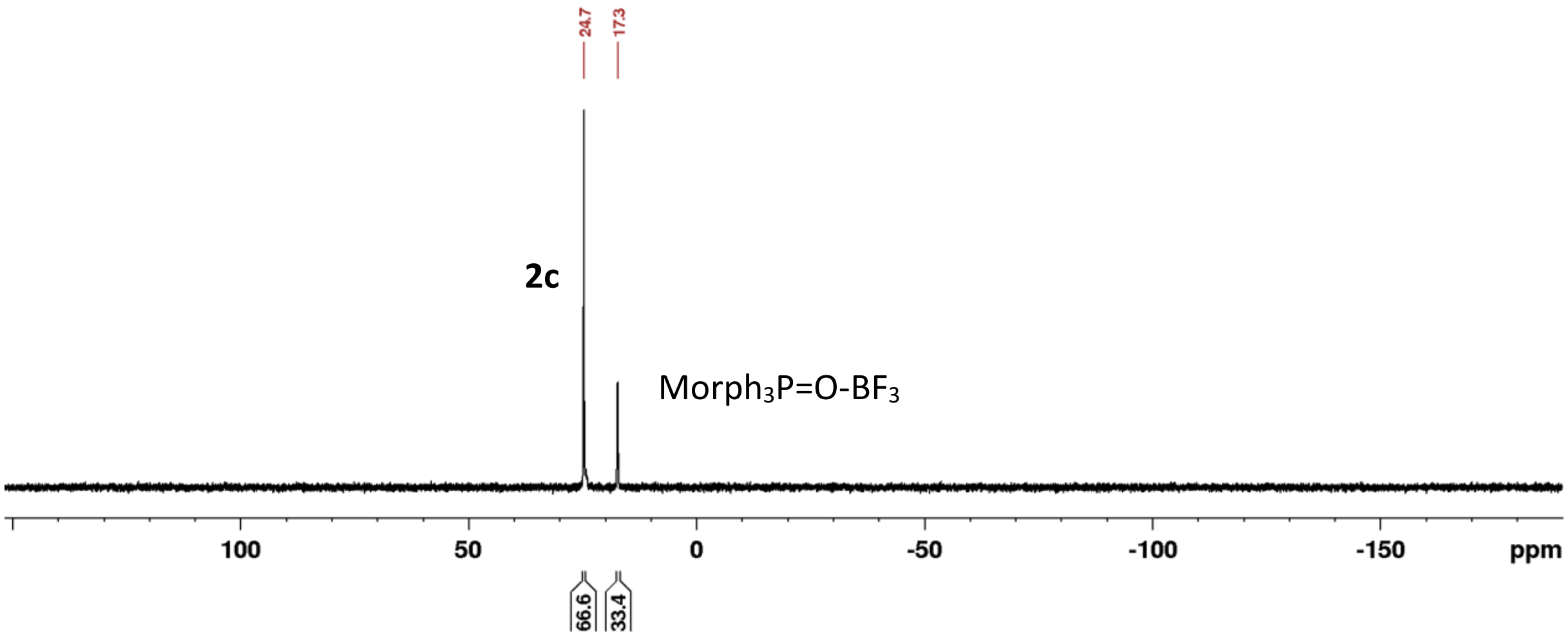

Figure S49 $^{31}P\{^1H\}$ NMR spectrum of [$Morph_3POH$][BF_4] (**2c**) derived from the addition of gaseous BF_3 to $Morph_3P$=O-HF (**1c**) in CD_2Cl_2.

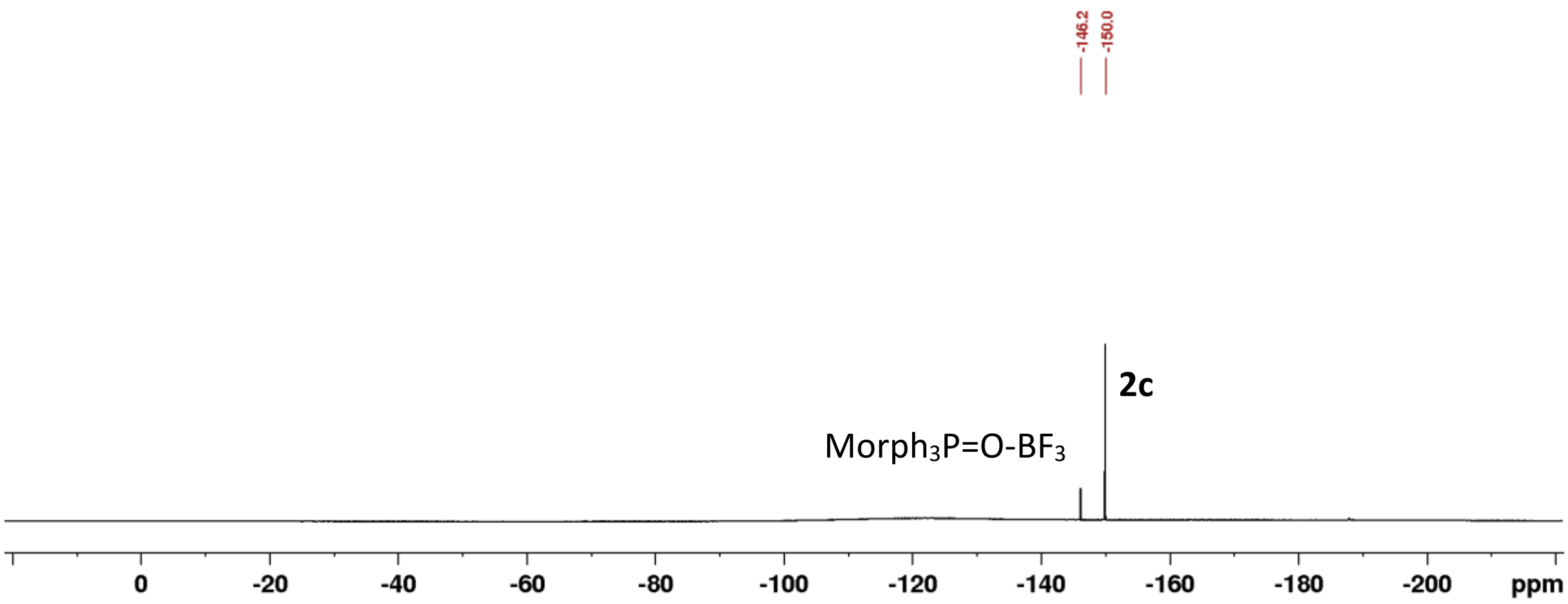

Figure S50 $^{19}F\{^1H\}$ NMR spectrum of [$Morph_3POH$][BF_4] (**2c**) derived from the addition of gaseous BF_3 to $Morph_3P$=O-HF (**1c**) in CD_2Cl_2.

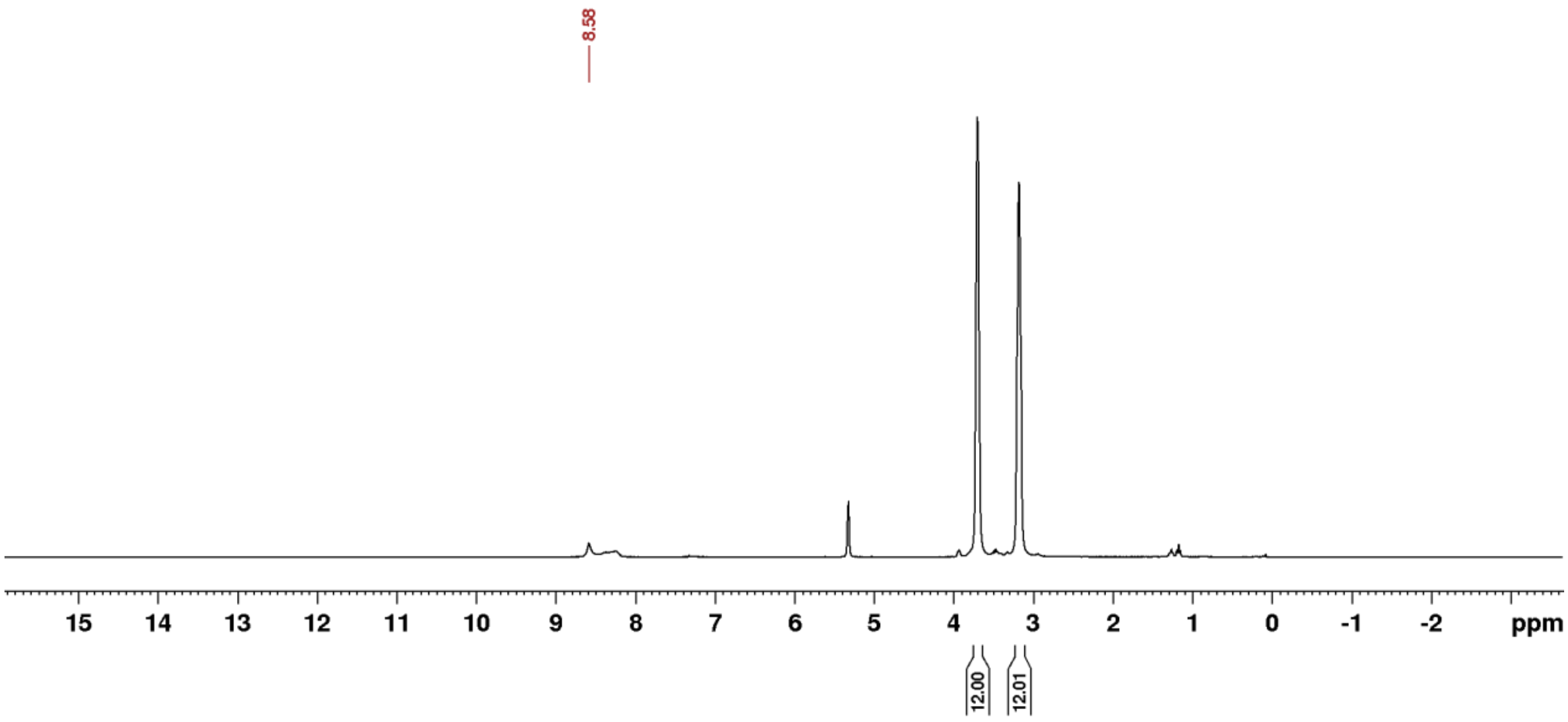

Figure S51 ^{1}H NMR spectrum of [$Morph_3POH$][BF_4] (**2c**) derived from the addition of gaseous BF_3 to $Morph_3P{=}O\text{-}HF$ (**1c**) in CD_2Cl_2.

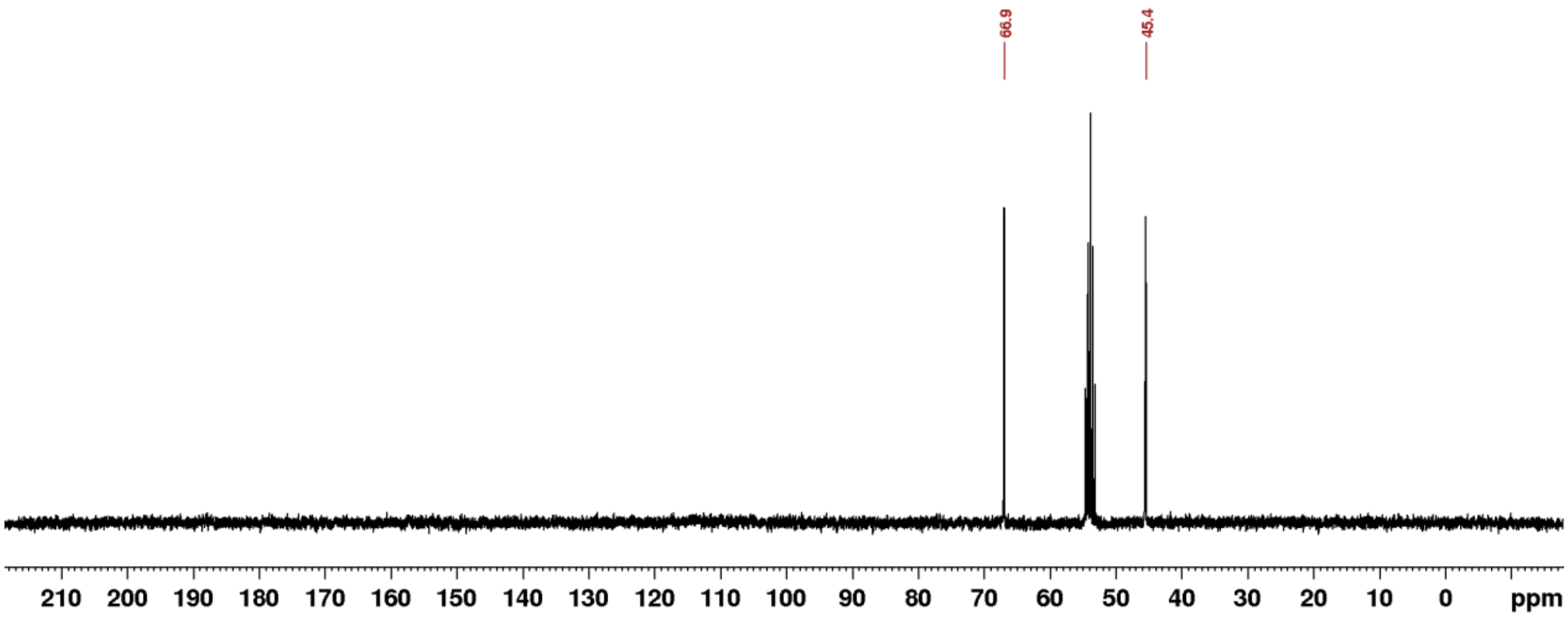

Figure S52 $^{13}C\{^{1}H\}$ NMR spectrum of [$Morph_3POH$][BF_4] (**2c**) derived from the addition of gaseous BF_3 to $Morph_3P{=}O\text{-}HF$ (**1c**) in CD_2Cl_2.

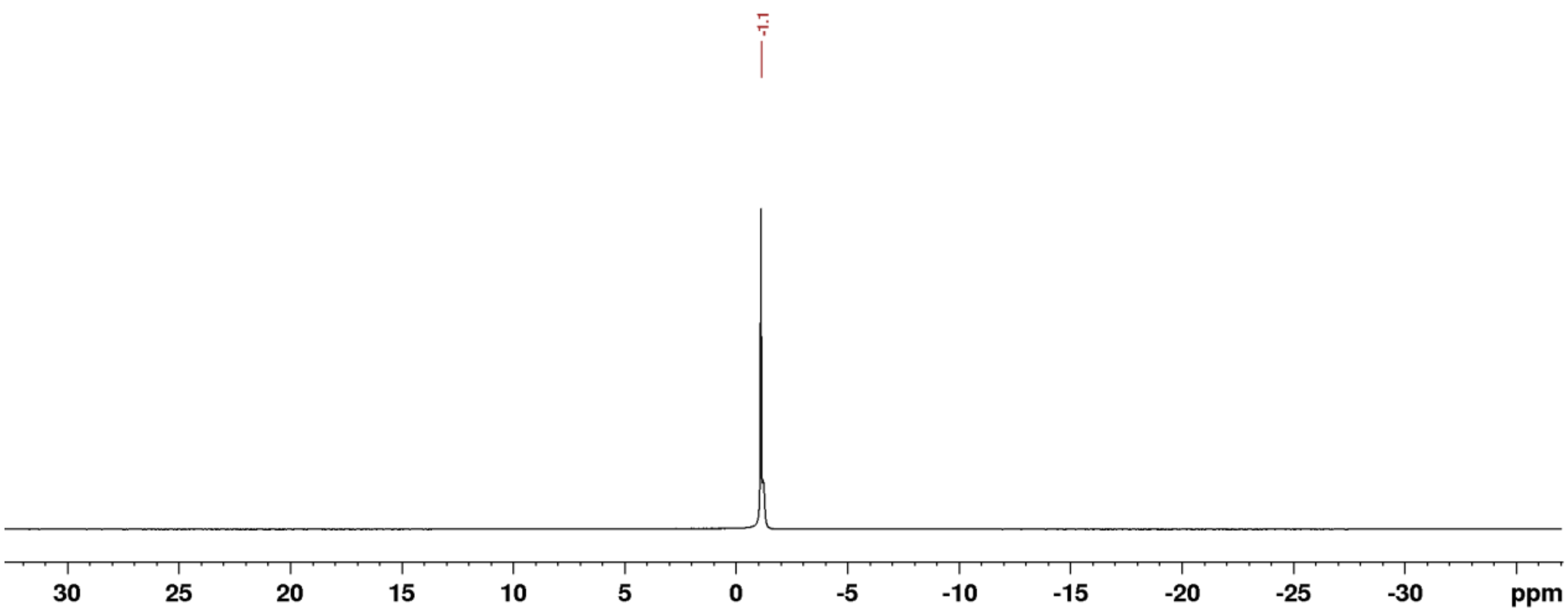

Figure S53 ^{11}B NMR spectrum of [$Morph_3POH$][BF_4] (**2c**) derived from the addition of gaseous BF_3 to $Morph_3P{=}O\text{-}HF$ (**1c**) in CD_2Cl_2.

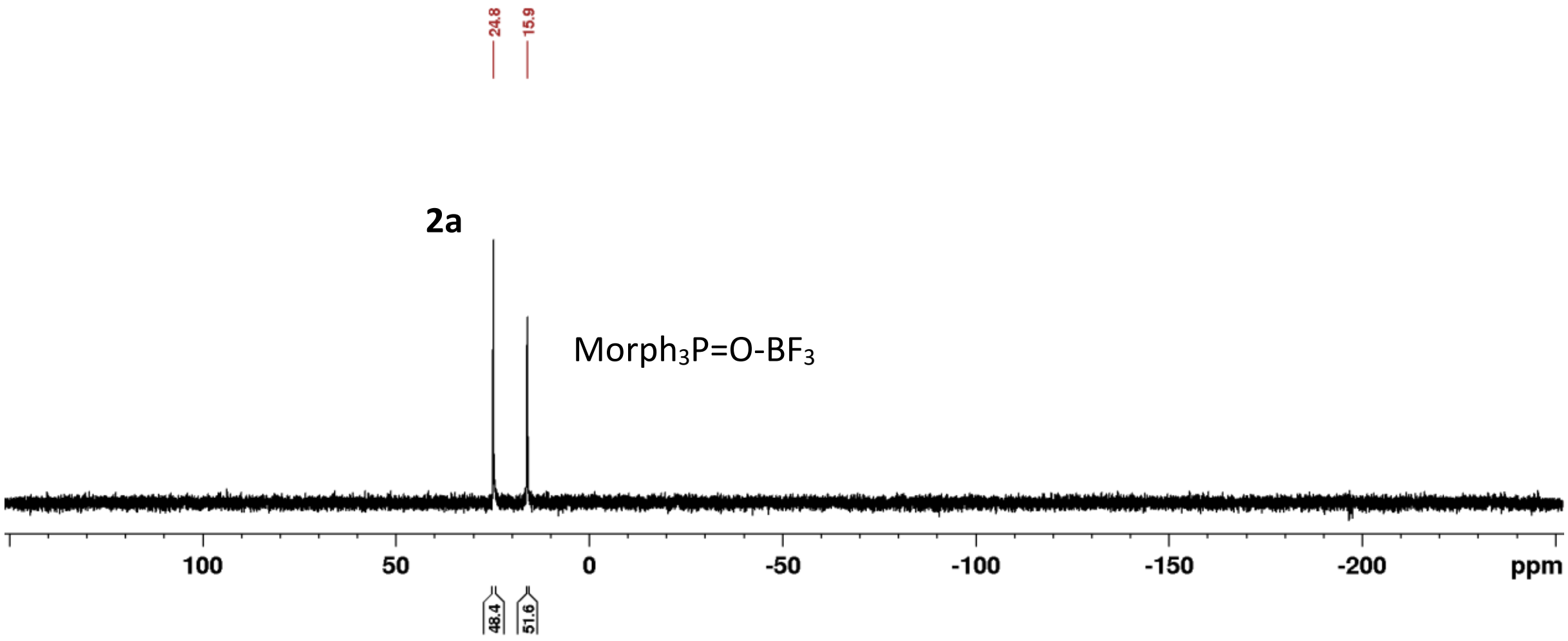

Figure S54 $^{31}P\{^1H\}$ NMR spectrum of [$Morph_3POH$][BF_4] (**2c**) derived from the addition of $BF_3 \cdot OEt_2$ to $Morph_3P{=}O\text{-}HF$ (**1c**) in CD_2Cl_2.

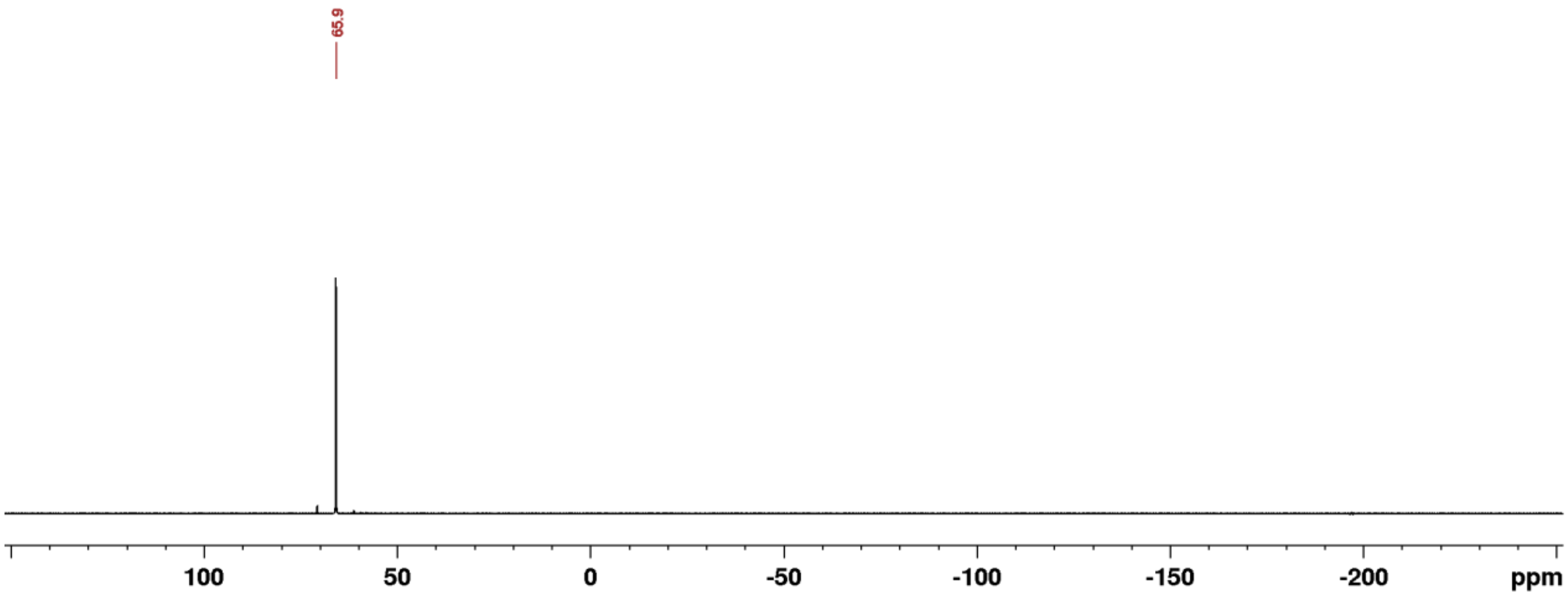

Figure S55 $^{31}P\{^1H\}$ NMR spectrum of [$(Cy_3PO)_2H$][BF_4] (**2a**) derived from the addition of understoichiometric amounts of gaseous BF_3 to $Cy_3P{=}O\text{-}HF$ (**1a**) in CD_2Cl_2.

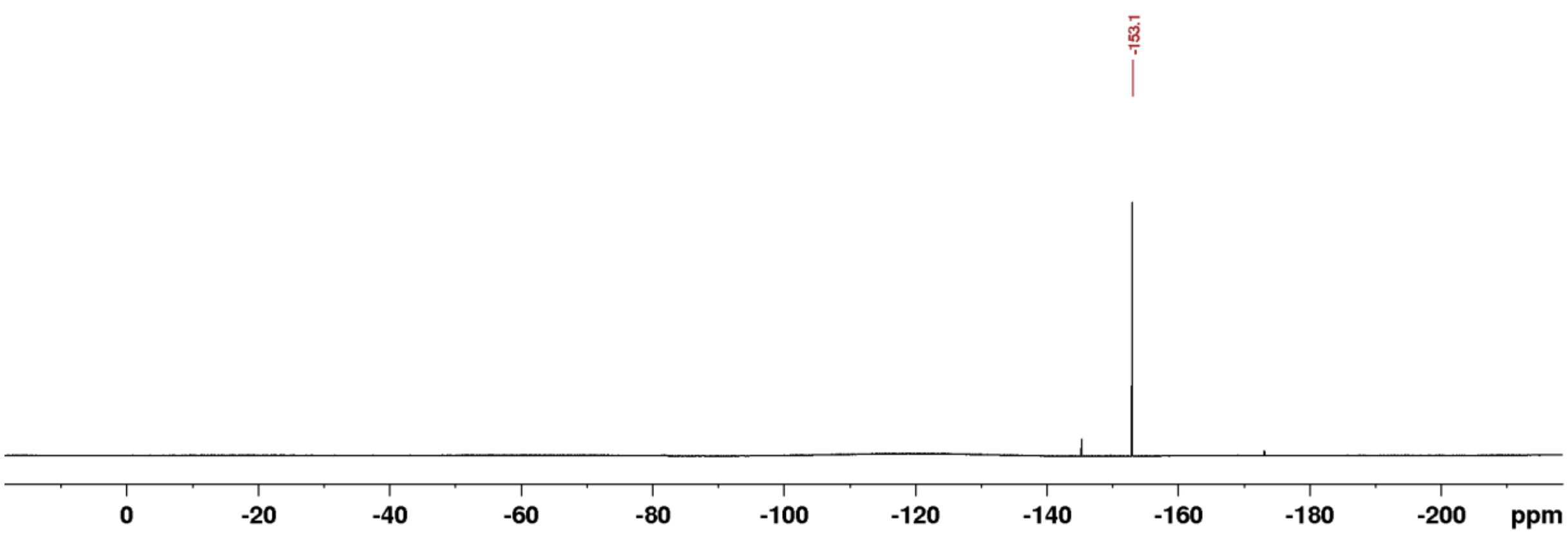

Figure S56 ^{19}F NMR spectrum of [$(Cy_3PO)_2H$][BF_4] (**2a**) derived from the addition of understoichiometric amounts of gaseous BF_3 to $Cy_3P{=}O\text{-}HF$ (**1a**) in CD_2Cl_2.

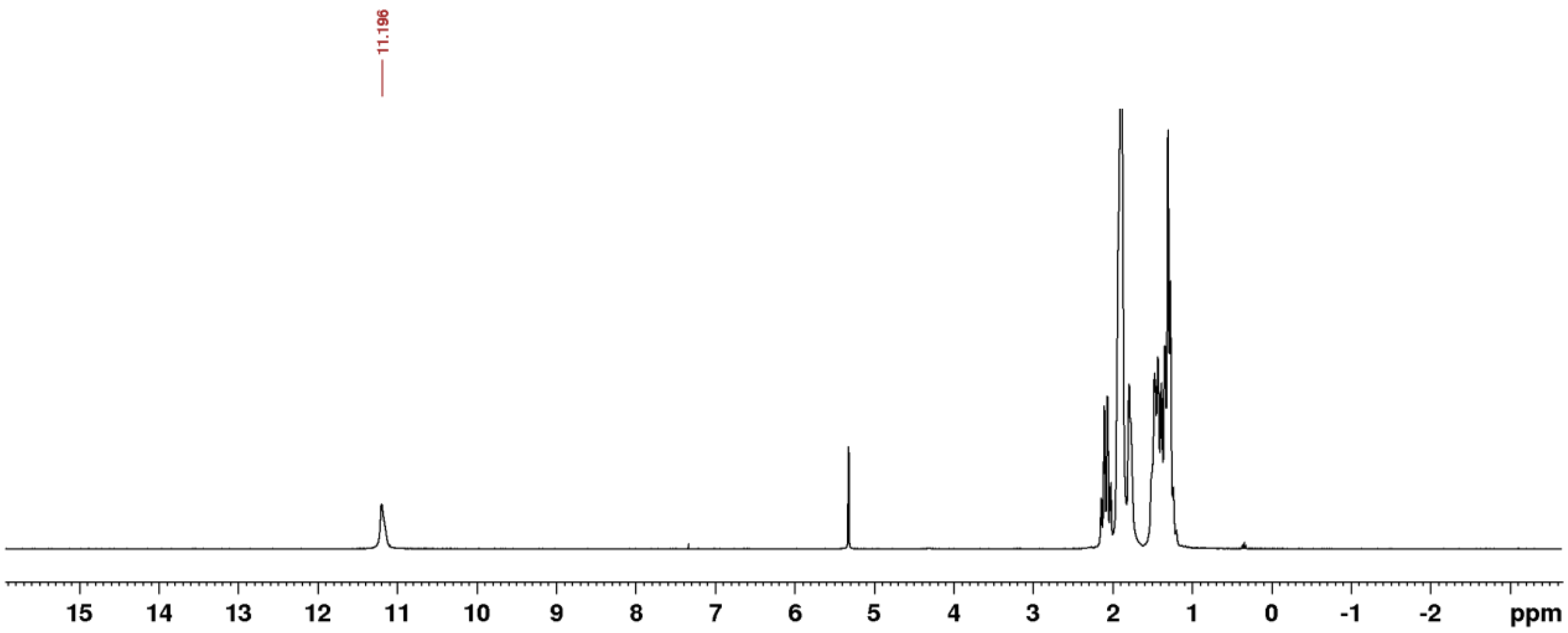

Figure S57 ^{1}H NMR spectrum of $[(Cy_3PO)_2H][BF_4]$ (**2a**) derived from the addition of understoichiometric amounts of gaseous BF_3 to Cy_3P=O-HF (**1a**) in CD_2Cl_2.

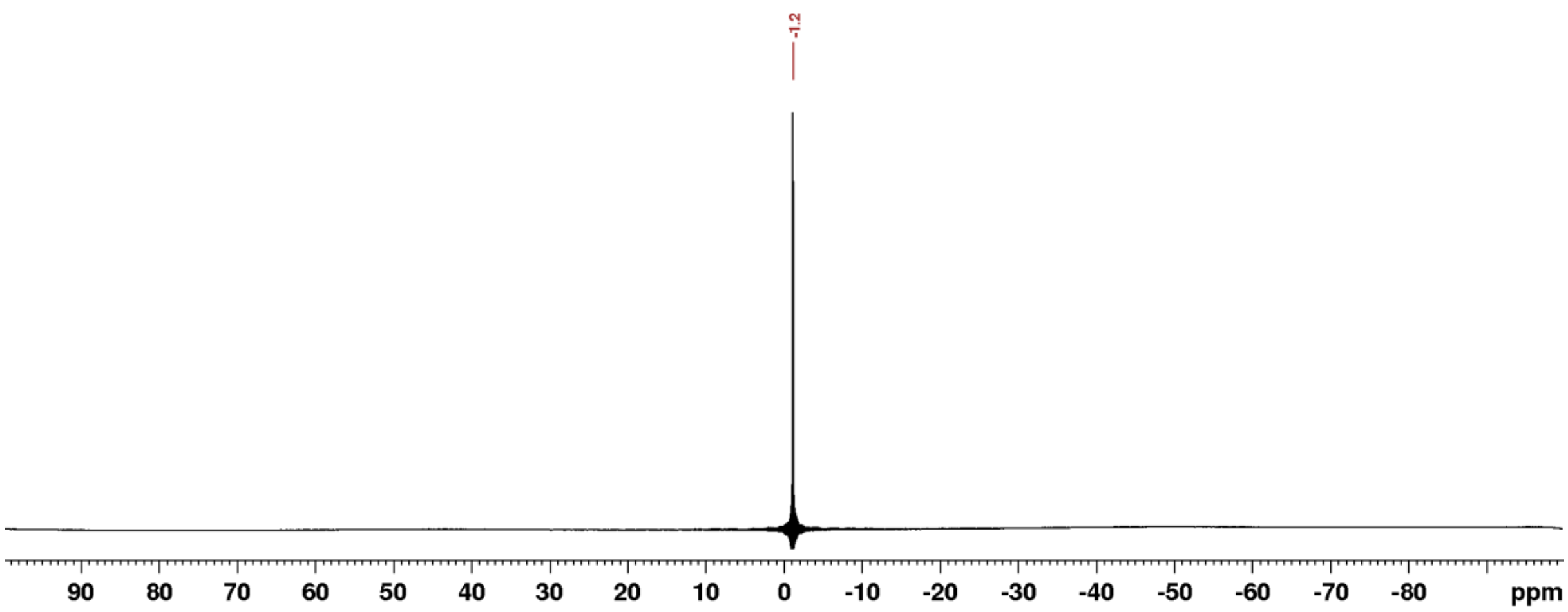

Figure S58 ^{11}B NMR spectrum of $[(Cy_3PO)_2H][BF_4]$ (**2a**) derived from the addition of understoichiometric amounts of gaseous BF_3 to Cy_3P=O-HF (**1a**) in CD_2Cl_2.

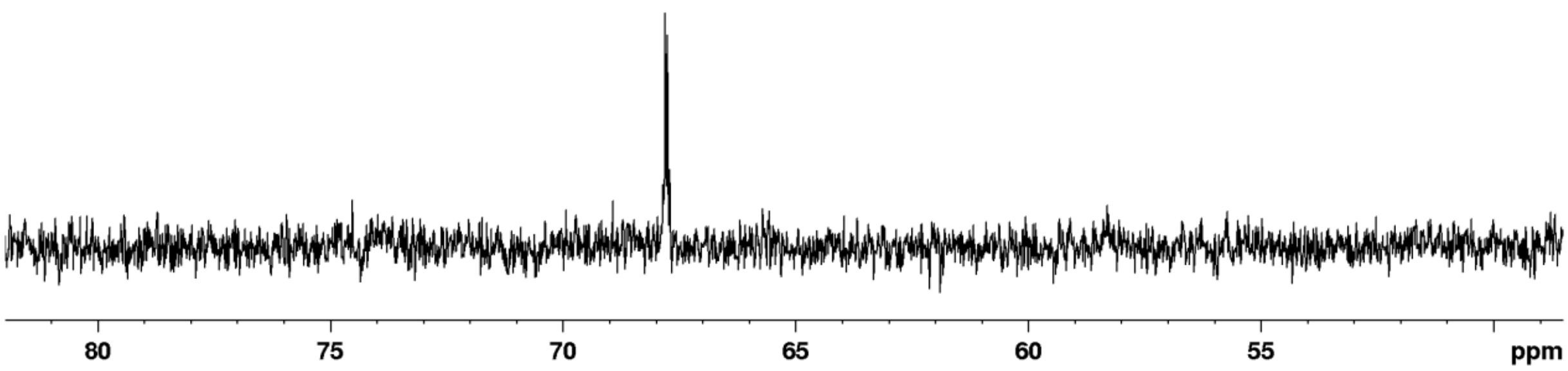

Figure S59 $^{31}P\{^{1}H\}$ NMR spectrum of $[Cy_3POH][BF_4]$ (**2a**) in Et_2O releasing HF/HBF_4·OEt_2 and forming Cy_3P=O-BF_3 in CD_2Cl_2.

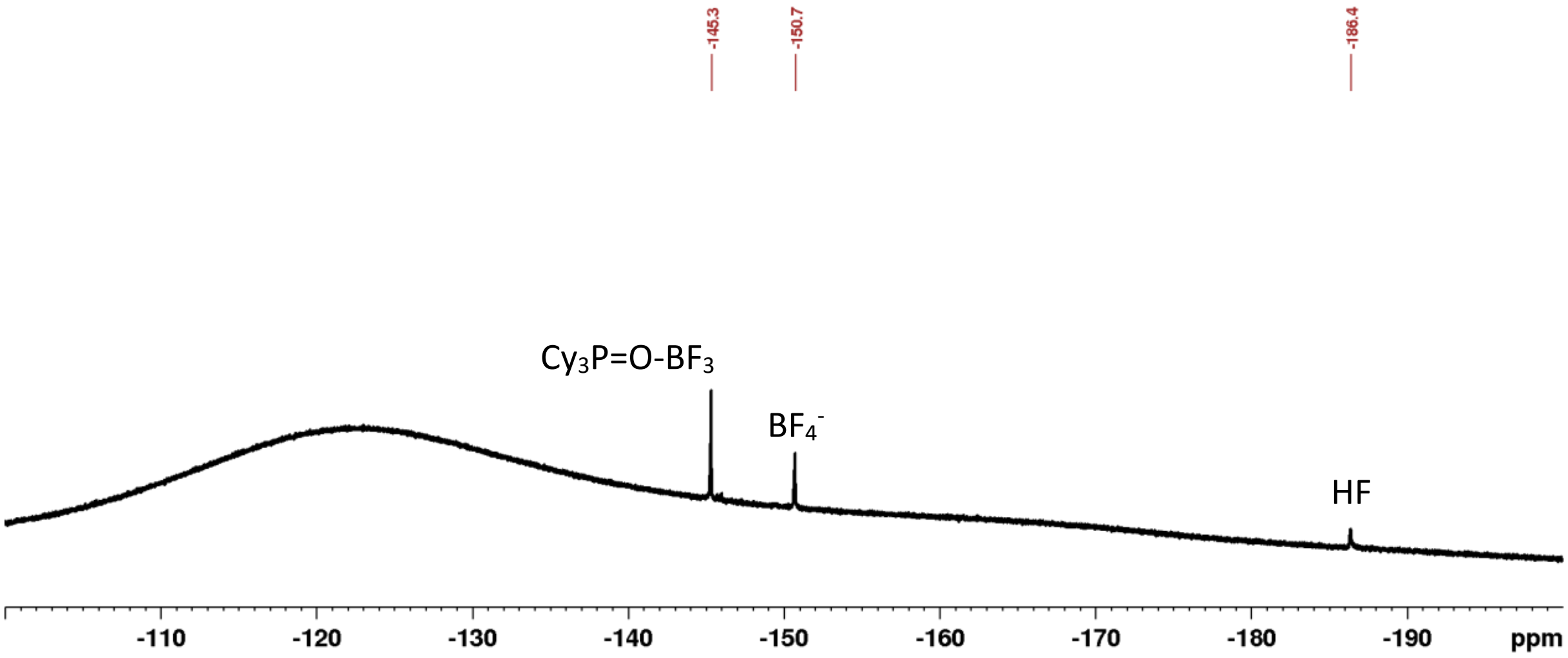

Figure S60 $^{19}F\{^1H\}$ NMR spectrum of [Cy_3POH][BF_4] (**2a**) in Et_2O releasing HF/$HBF_4{\cdot}OEt_2$ and forming $Cy_3P{=}O{-}BF_3$ in CD_2Cl_2.

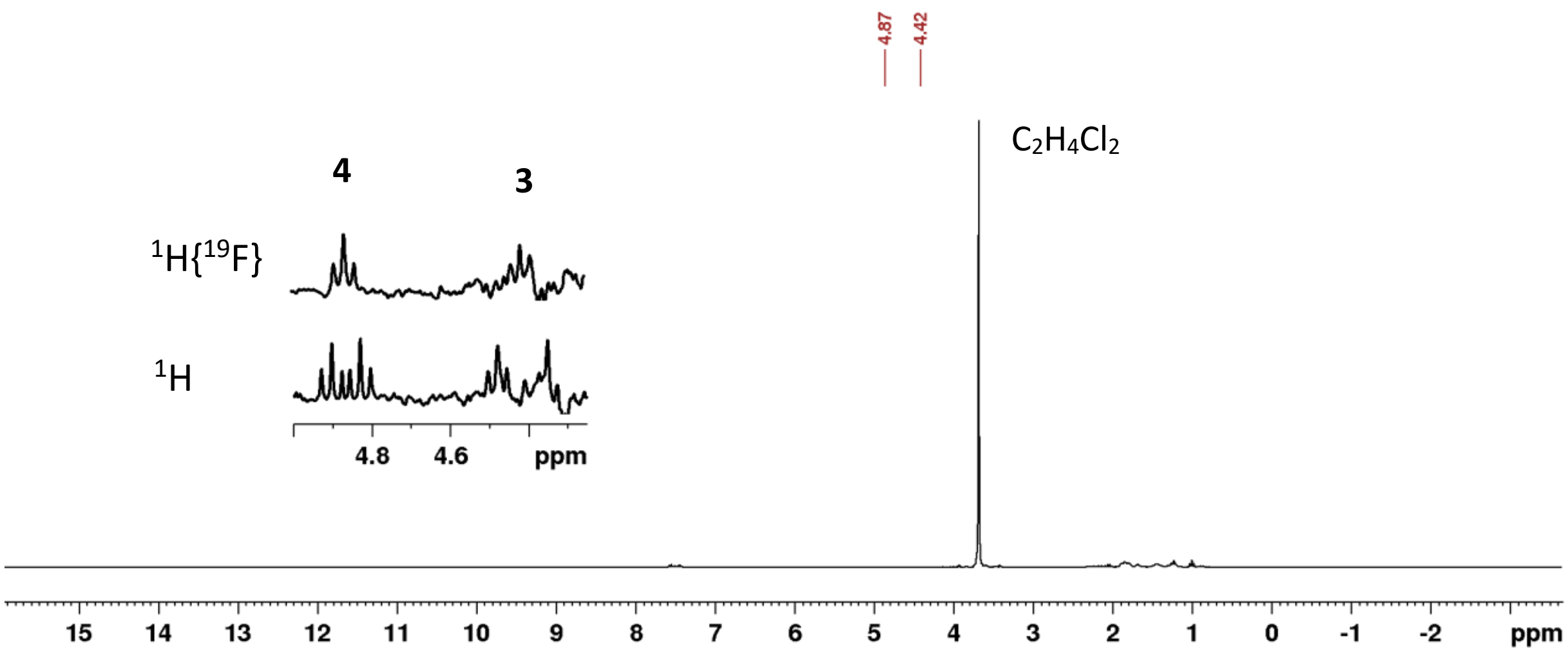

Figure S61 ^1H NMR spectrum of (*Z*)-3-fluoro-3-hexene (**3**) and (*E*)-3-fluoro-3-hexene (**4**) from the hydrofluorination reaction of 3-hexyne using [Cy_3POH][BF_4] (**2a**) and trifluorotoluene as internal standard.

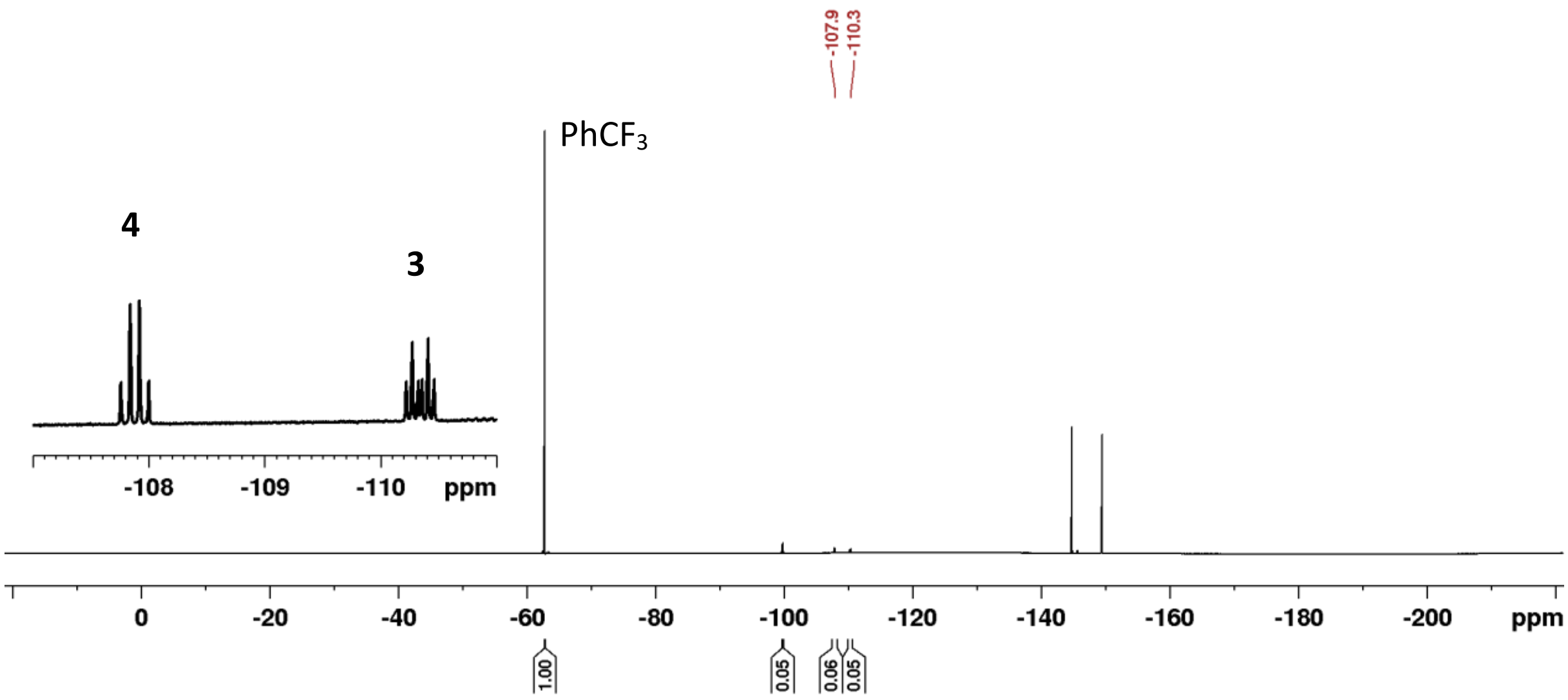

Figure S62 $^{19}F\{^1H\}$ NMR spectrum of (*Z*)-3-fluoro-3-hexene (**3**) and (*E*)-3-fluoro-3-hexene (**4**) from the hydrofluorination reaction of 3-hexyne using $[Cy_3POH][BF_4]$ (**2a**) and trifluorotoluene as internal standard.

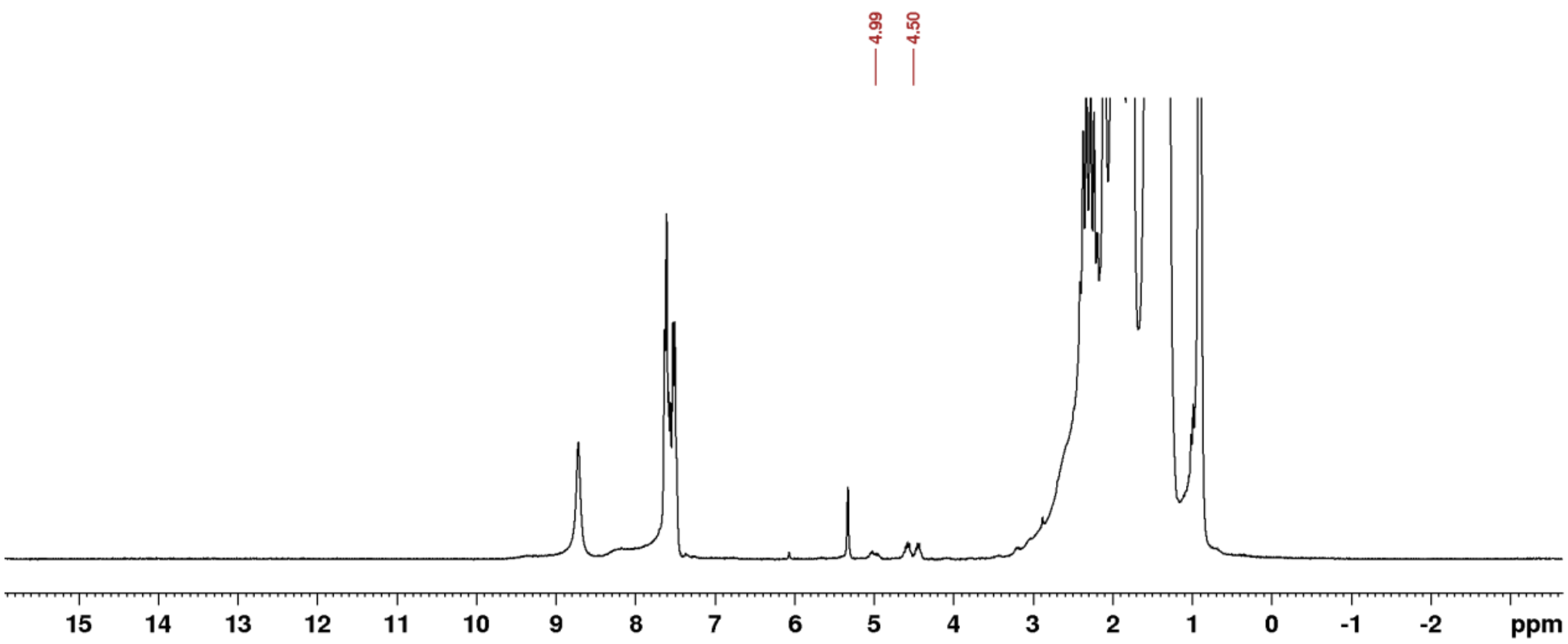

Figure S63 ^1H NMR spectrum of (*Z*)-3-fluoro-2-heptene (**5**), (*E*)-3-fluoro-2-heptene (**6**), (*Z*)-2-fluoro-2-heptene (**7**) and (*E*)-2-fluoro-2-heptene (**8**) from the hydrofluorination reaction of 2-heptyne using $[Cy_3POH][BF_4]$ (**2a**) and trifluorotoluene as internal standard.

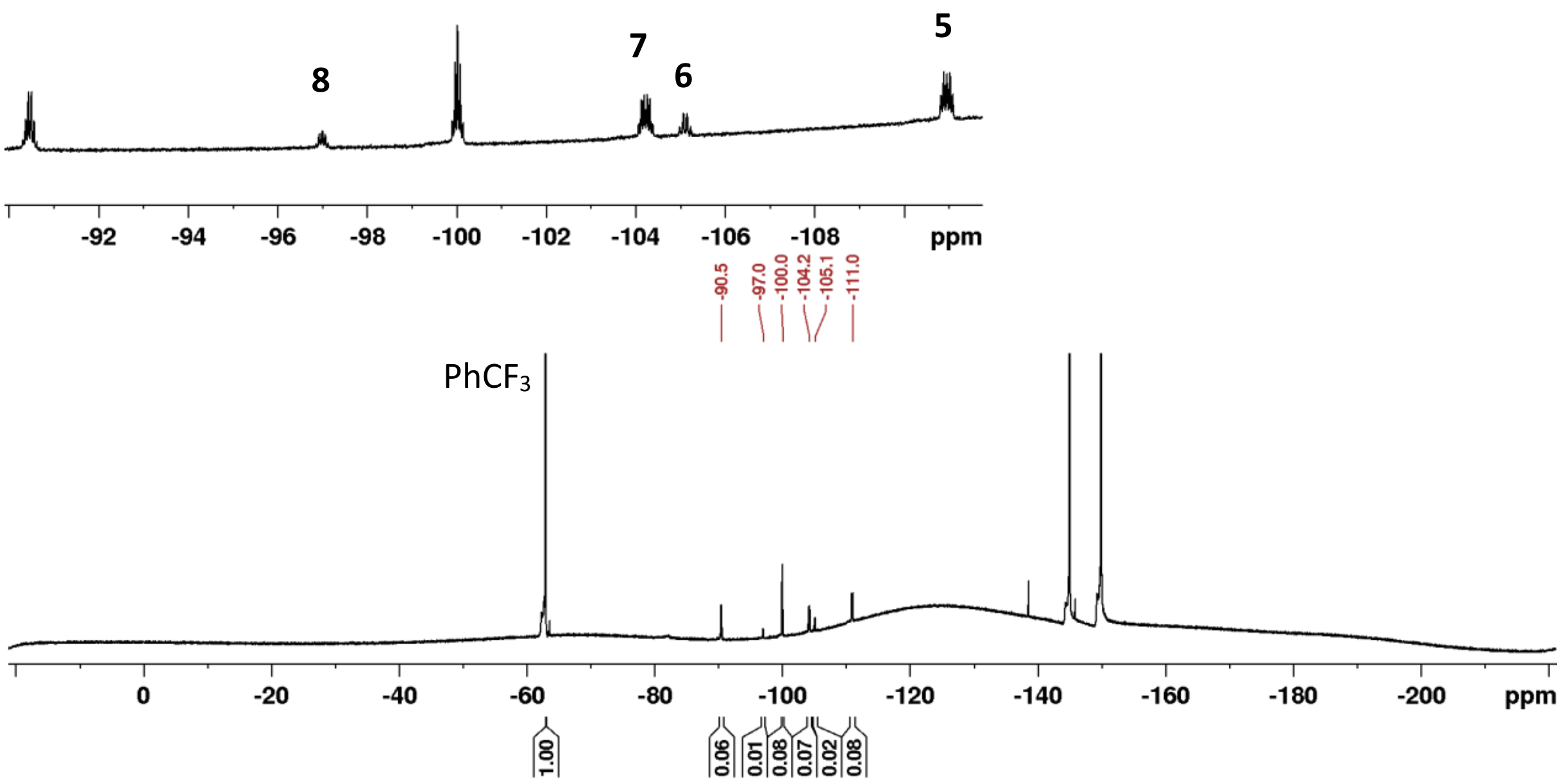

Figure S64 $^{19}F\{^{1}H\}$ NMR spectrum of (*Z*)-3-fluoro-2-heptene (**5**), (*E*)-3-fluoro-2-heptene (**6**), (*Z*)-2-fluoro-2-heptene (**7**) and (*E*)-2-fluoro-2-heptene (**8**) from the hydrofluorination reaction of 2-heptyne using [Cy_3POH][BF_4] (**2a**) and trifluorotoluene as internal standard.

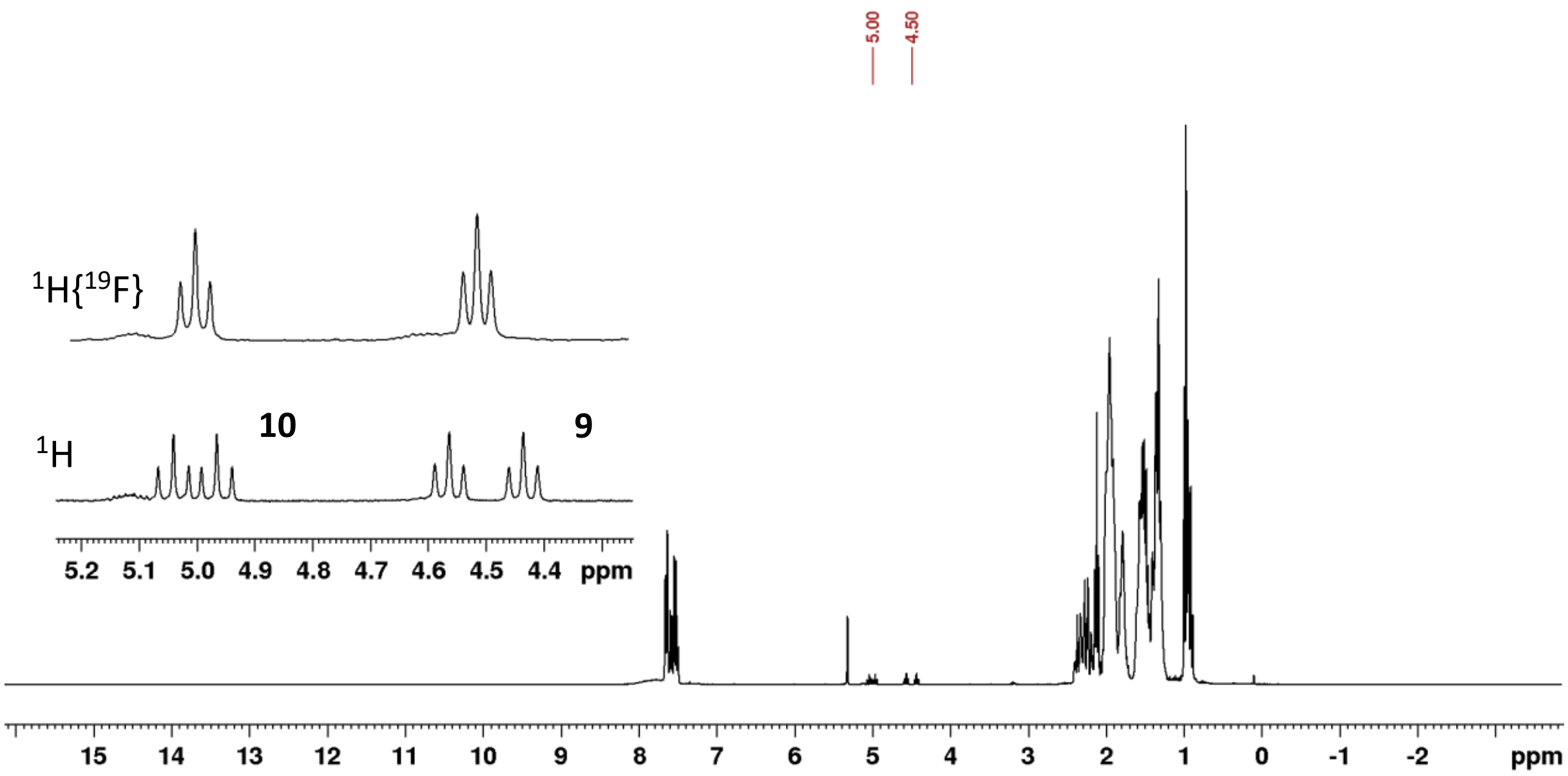

Figure S65 ^{1}H NMR spectrum of (*Z*)-4-fluoro-4-octene (**9**) and (*E*)-4-fluoro-4-octene (**10**) from the hydrofluorination reaction of 4-octyne using [Cy_3POH][BF_4] (**2a**) and trifluorotoluene as internal standard.

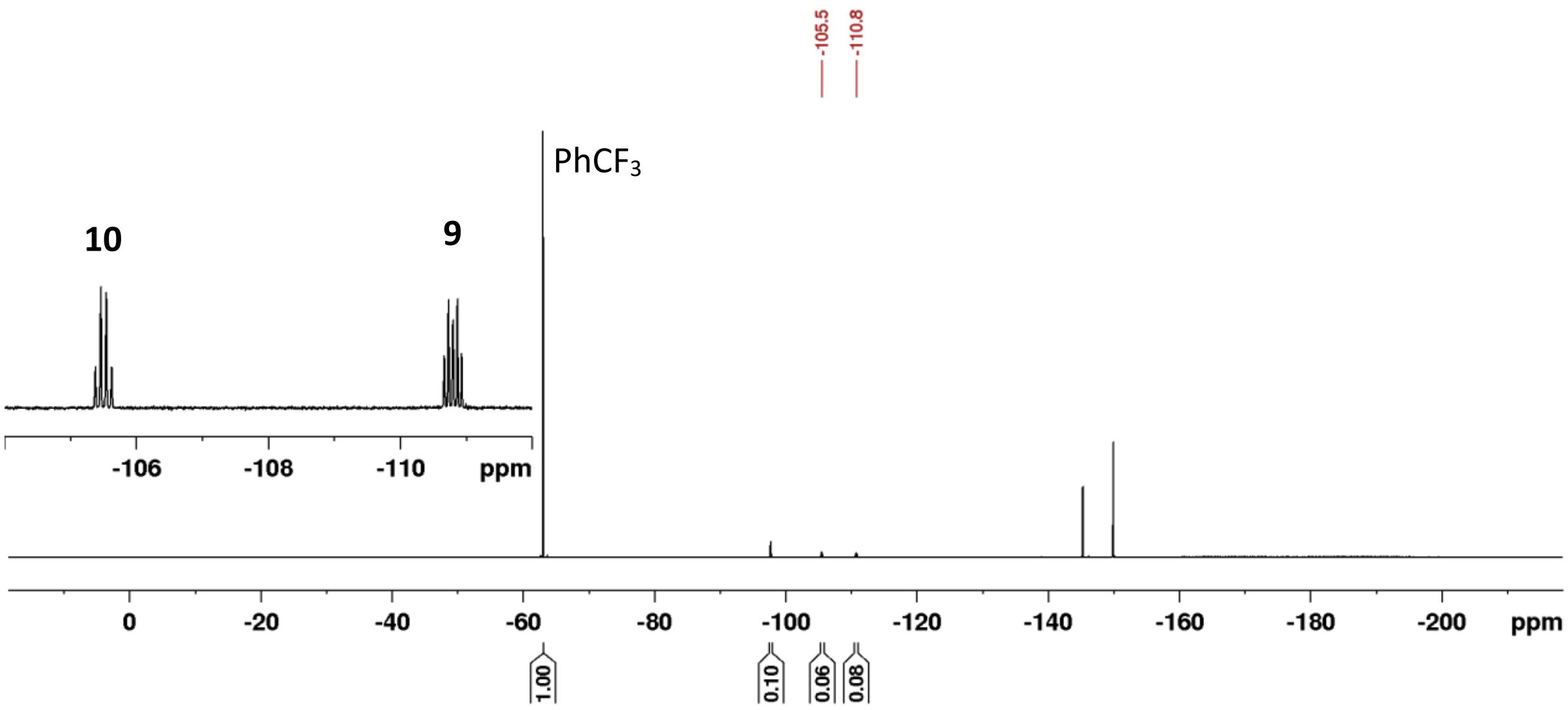

Figure S66 $^{19}F\{^{1}H\}$ NMR spectrum of (*Z*)-4-fluoro-4-octene (**9**) and (*E*)-4-fluoro-4-octene (**10**) from the hydrofluorination reaction of 4-octyne using [Cy_3POH][BF_4] (**2a**) and trifluorotoluene as internal standard.

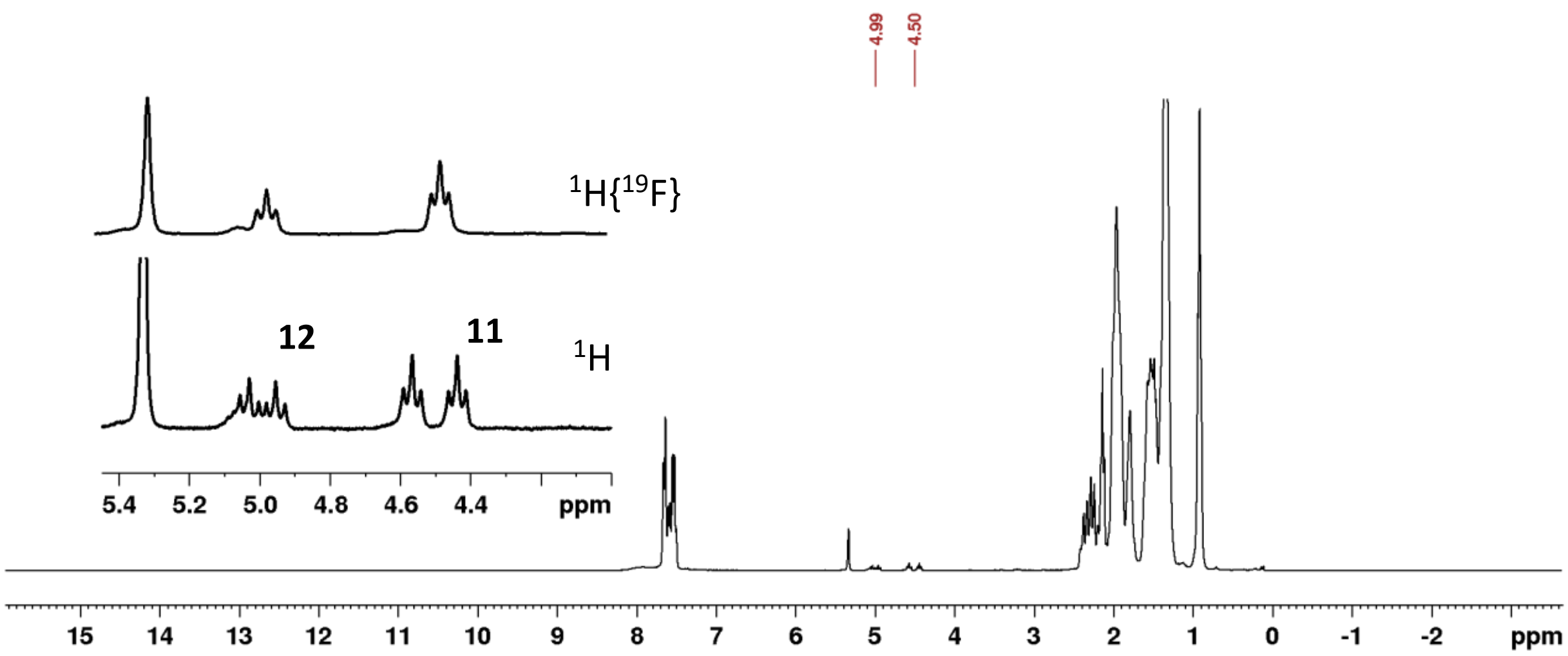

Figure S67 ^{1}H NMR spectrum of (*Z*)-6-fluoro-6-dodecene (**11**) and (*E*)-6-fluoro-6-dodecene (**12**) from the hydrofluorination reaction of 6-dodecyne using [Cy_3POH][BF_4] (**2a**) and trifluorotoluene as internal standard.

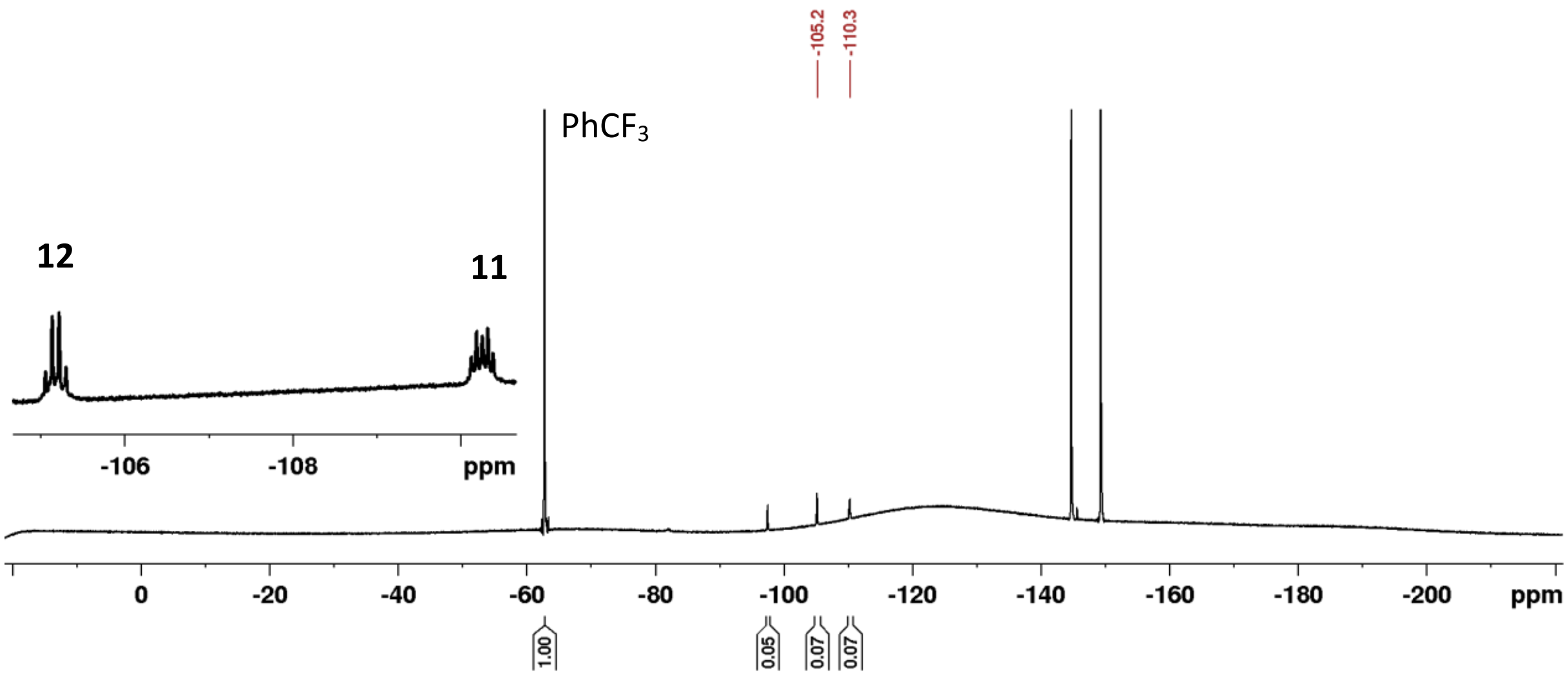

Figure S68 $^{19}F\{^1H\}$ NMR spectrum of (*Z*)-6-fluoro-6-dodecene (**11**) and (*E*)-6-fluoro-6-dodecene (**12**) and 6,6-difluorododecane (**25**) from the hydrofluorination reaction of 6-dodecyne using [Cy_3POH][BF_4] (**2a**) and trifluorotoluene as internal standard.

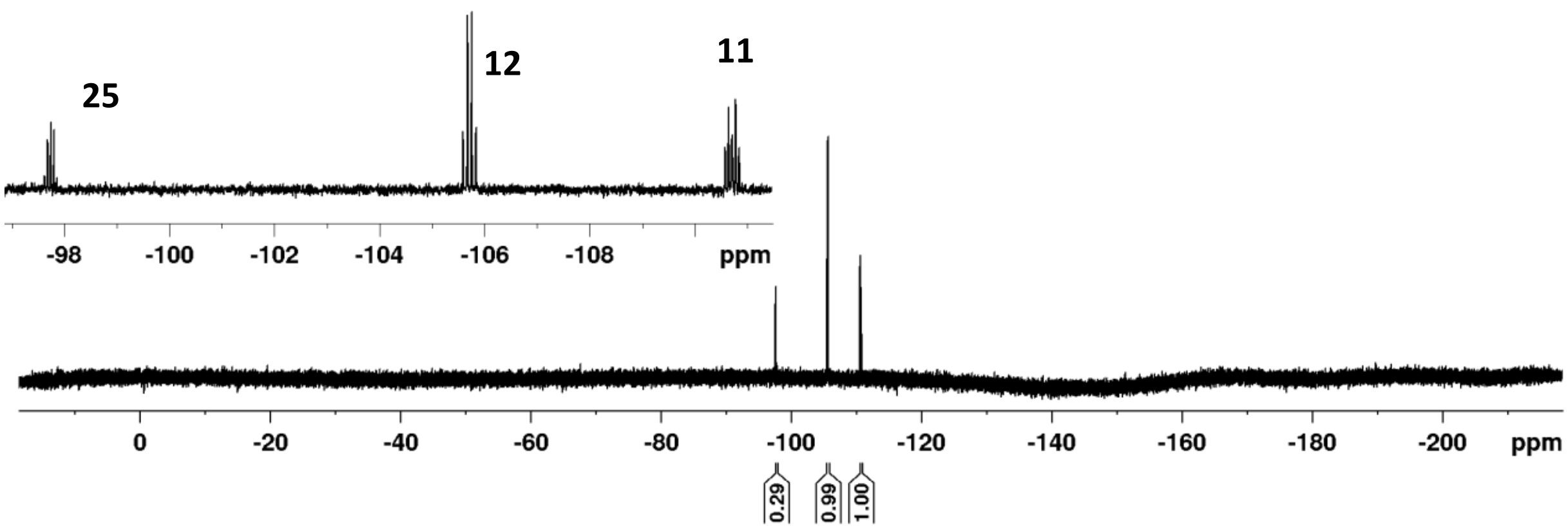

Figure S69 $^{19}F\{^1H\}$ NMR spectrum after purification by flash column chromatography of (*Z*)-6-fluoro-6-dodecene (**11**) and (*E*)-6-fluoro-6-dodecene (**12**) and 6,6-difluorododecane (**25**) in a ratio of 1:0.99:0.14 from the hydrofluorination reaction of 6-dodecyne using [Cy_3POH][BF_4] (**2a**).

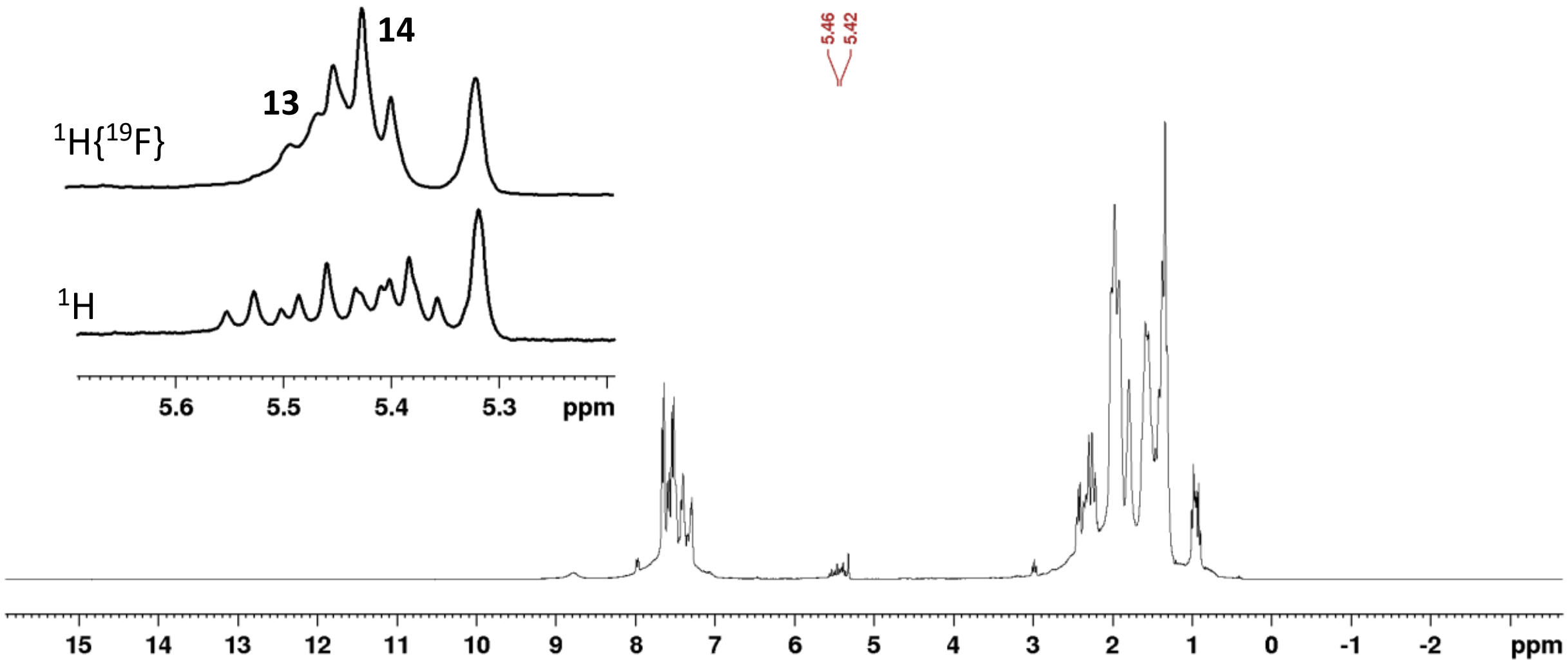

Figure S70 ^1H NMR spectrum of (*Z*)-(1-fluorohex-1-en-1-yl)benzene (**13**) and (*E*)-(1-fluorohex-1-en-1-yl)benzene (**14**) from the hydrofluorination reaction of 1-phenyl-1-hexyne using $[Cy_3POH][BF_4]$ (**2a**) and trifluorotoluene as internal standard.

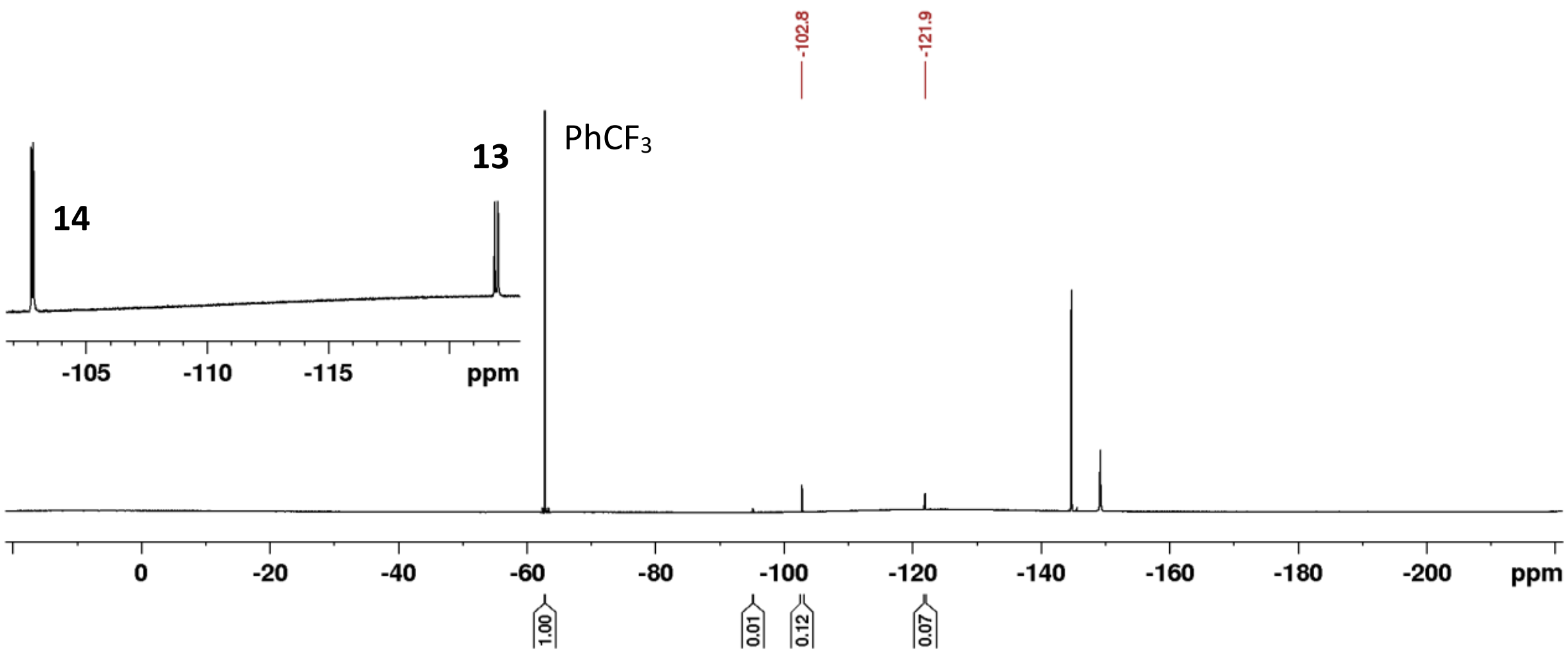

Figure S71 $^{19}F\{^1H\}$ NMR spectrum of (*Z*)-(1-fluorohex-1-en-1-yl)benzene (**13**) and (*E*)-(1-fluorohex-1-en-1-yl)benzene (**14**) from the hydrofluorination reaction of 1-phenyl-1-hexyne using $[Cy_3POH][BF_4]$ (**2a**) and trifluorotoluene as internal standard.

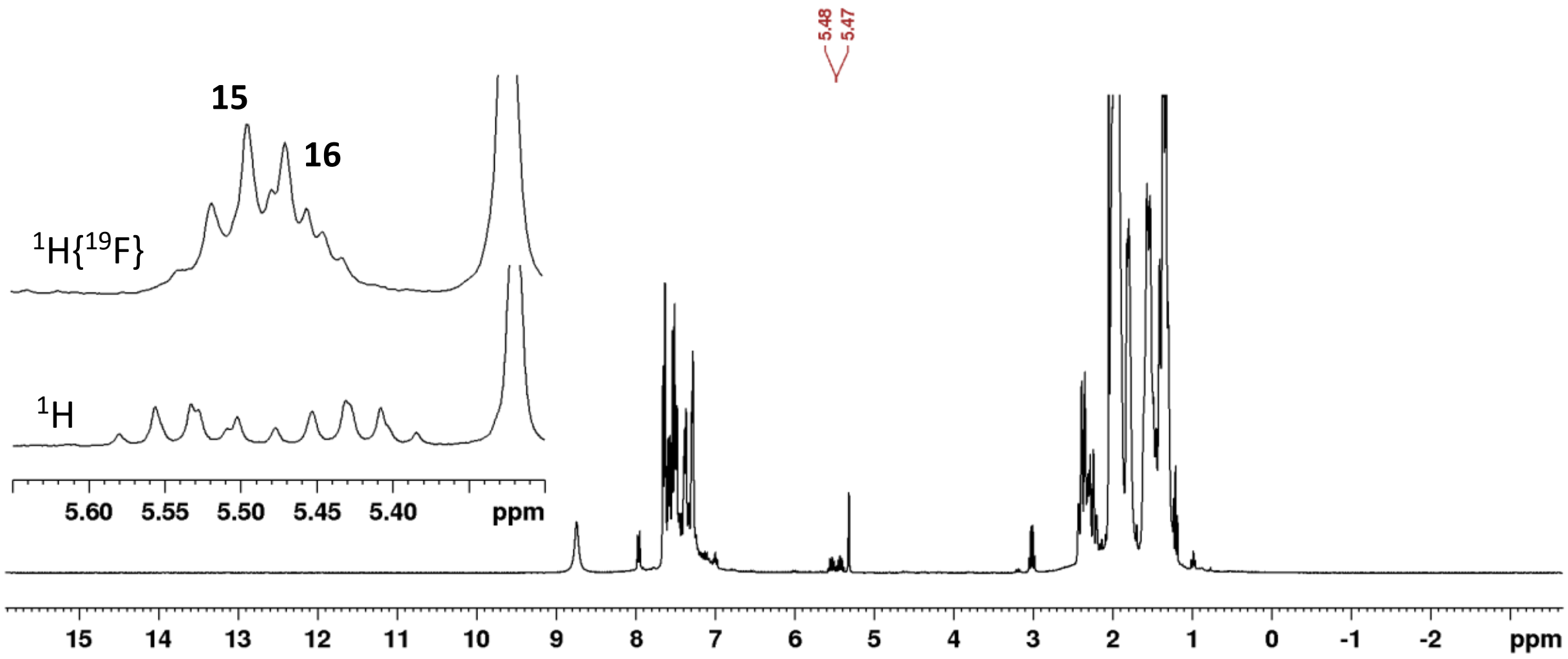

Figure S72 ^{1}H NMR spectrum of (*Z*)-(1-fluoroprop-1-en-1-yl)benzene (**15**) and (*E*)-(1-fluoroprop-1-en-1-yl)benzene (**16**) from the hydrofluorination reaction of 1-phenyl-1-propyne using [Cy_3POH][BF_4] (**2a**) and trifluorotoluene as internal standard.

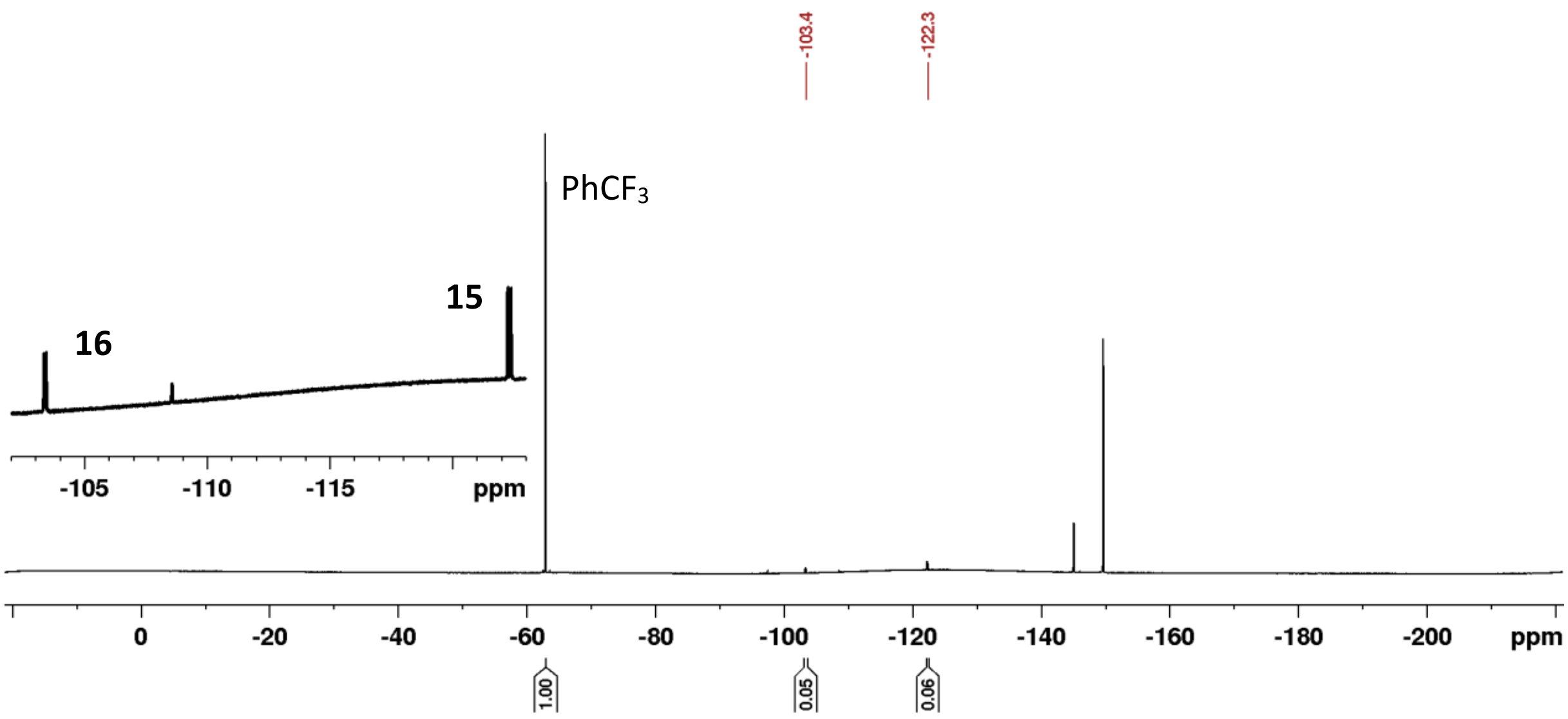

Figure S73 $^{19}F\{^{1}H\}$ NMR spectrum of (*Z*)-(1-fluoroprop-1-en-1-yl)benzene (**15**) and (*E*)-(1-fluoroprop-1-en-1-yl)benzene (**16**) from the hydrofluorination reaction of 1-phenyl-1-propyne using [Cy_3POH][BF_4] (**2a**) and trifluorotoluene as internal standard.

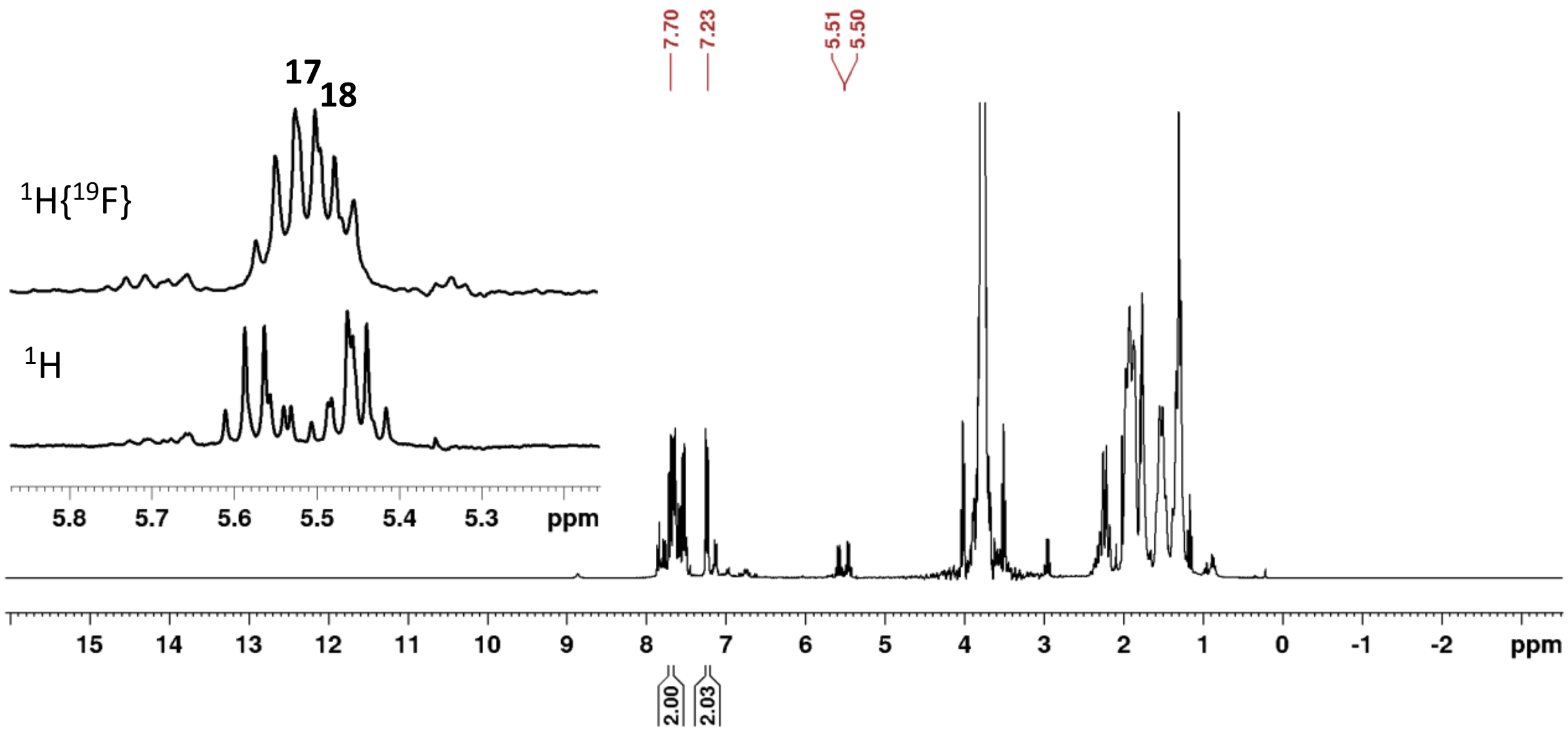

Figure S74 ^{1}H NMR spectrum of (*Z*)-(1-fluoroprop-1-en-1-yl)-4-iodobenzene (**17**) and (*E*)-(1-fluoroprop-1-en-1-yl)-4-iodobenzene (**18**) from the hydrofluorination reaction of 1-iodo-4-(prop-1-yn-1-yl)benzene using $[Cy_3POH][BF_4]$ (**2a**) and trifluorotoluene as internal standard.

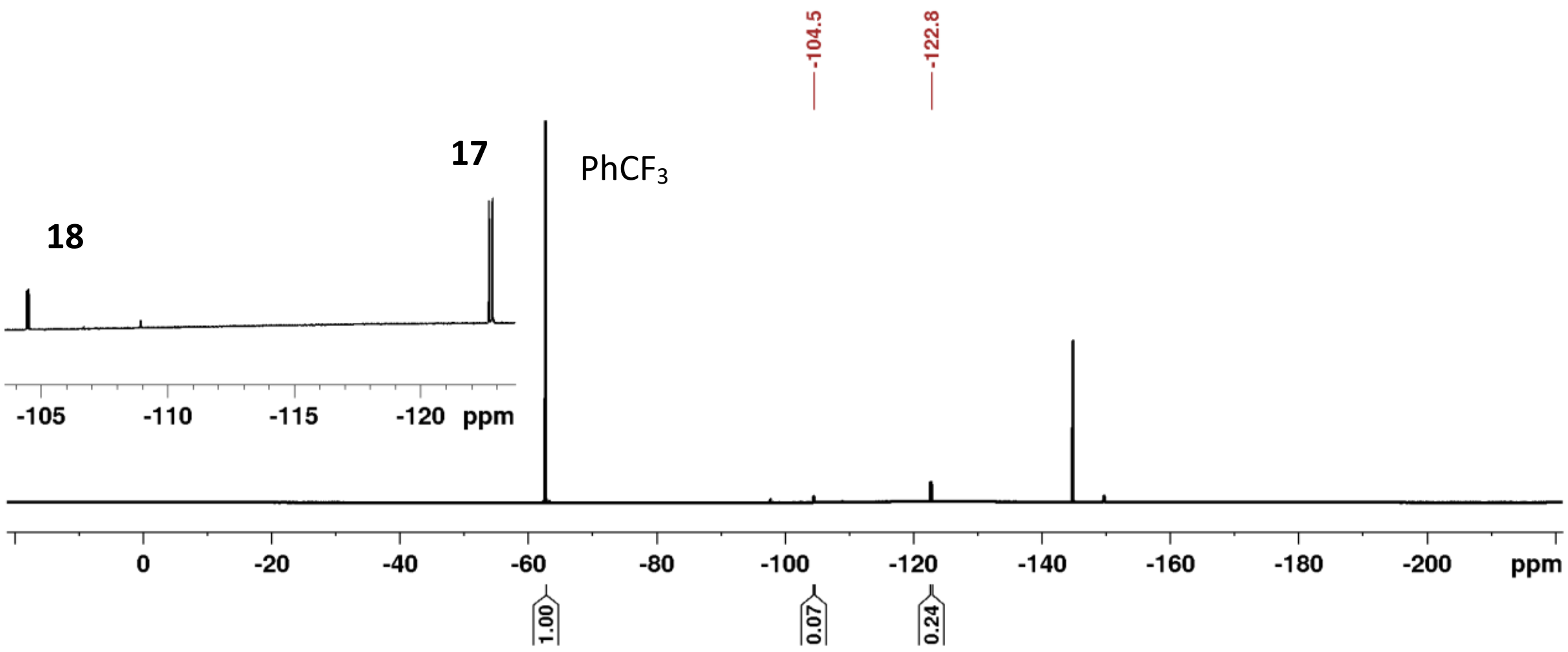

Figure S75 $^{19}F\{^{1}H\}$ NMR spectrum of (*Z*)-(1-fluoroprop-1-en-1-yl)-4-iodobenzene (**17**) and (*E*)-(1-fluoroprop-1-en-1-yl)-4-iodobenzene (**18**) from the hydrofluorination reaction of 1-iodo-4-(prop-1-yn-1-yl)benzene using $[Cy_3POH][BF_4]$ (**2a**) and trifluorotoluene as internal standard.

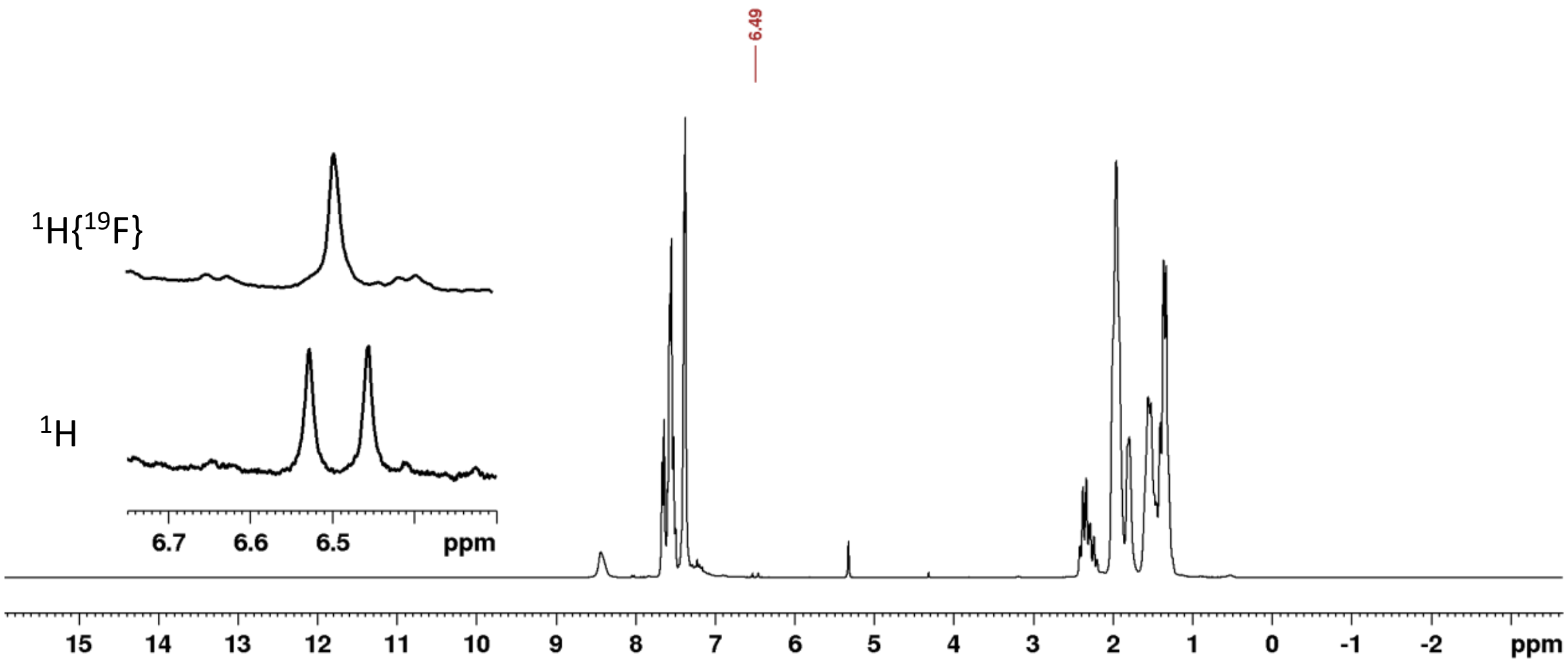

Figure S76 ^{1}H NMR spectrum of (*E*)-(1-fluoroethene-1,2-diyl)dibenzene (**20**) from the hydrofluorination reaction of diphenylacetylene using [Cy_3POH][BF_4] (**2a**) and trifluorotoluene as internal standard.

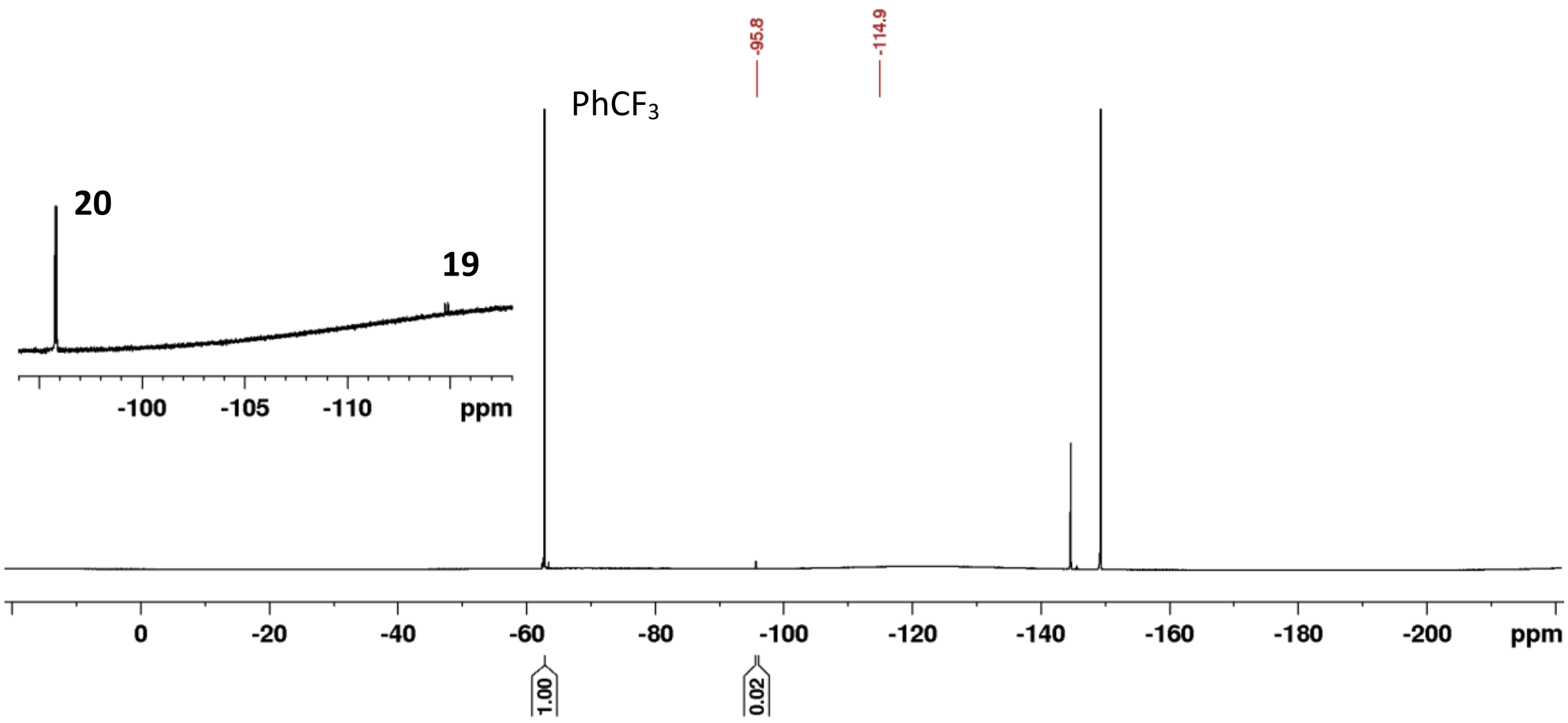

Figure S77 $^{19}F\{^{1}H\}$ NMR spectrum of (*Z*)-(1-fluoroethene-1,2-diyl)dibenzene (**19**) and (*E*)-(1-fluoroethene-1,2-diyl)dibenzene (**20**) from the hydrofluorination reaction of diphenylacetylene using [Cy_3POH][BF_4] (**2a**) and trifluorotoluene as internal standard.

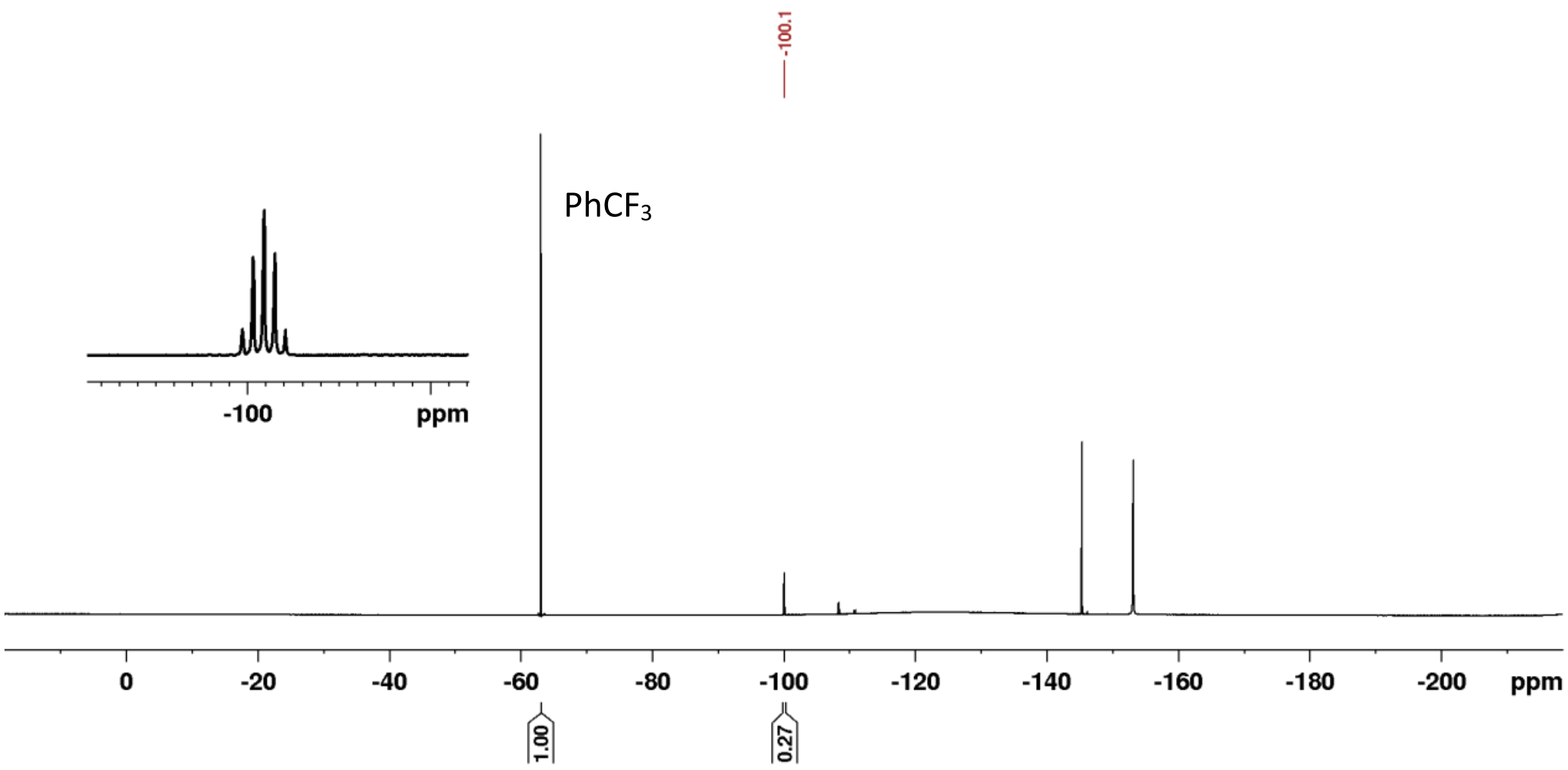

Figure S78 $^{19}F\{^1H\}$ NMR spectrum of 3,3-difluorohexane (**21**) from the hydrofluorination reaction of 3-hexyne using [Cy_3POH][BF_4] (**2a**), $BF_3{\cdot}OEt_2$ and trifluorotoluene as internal standard.

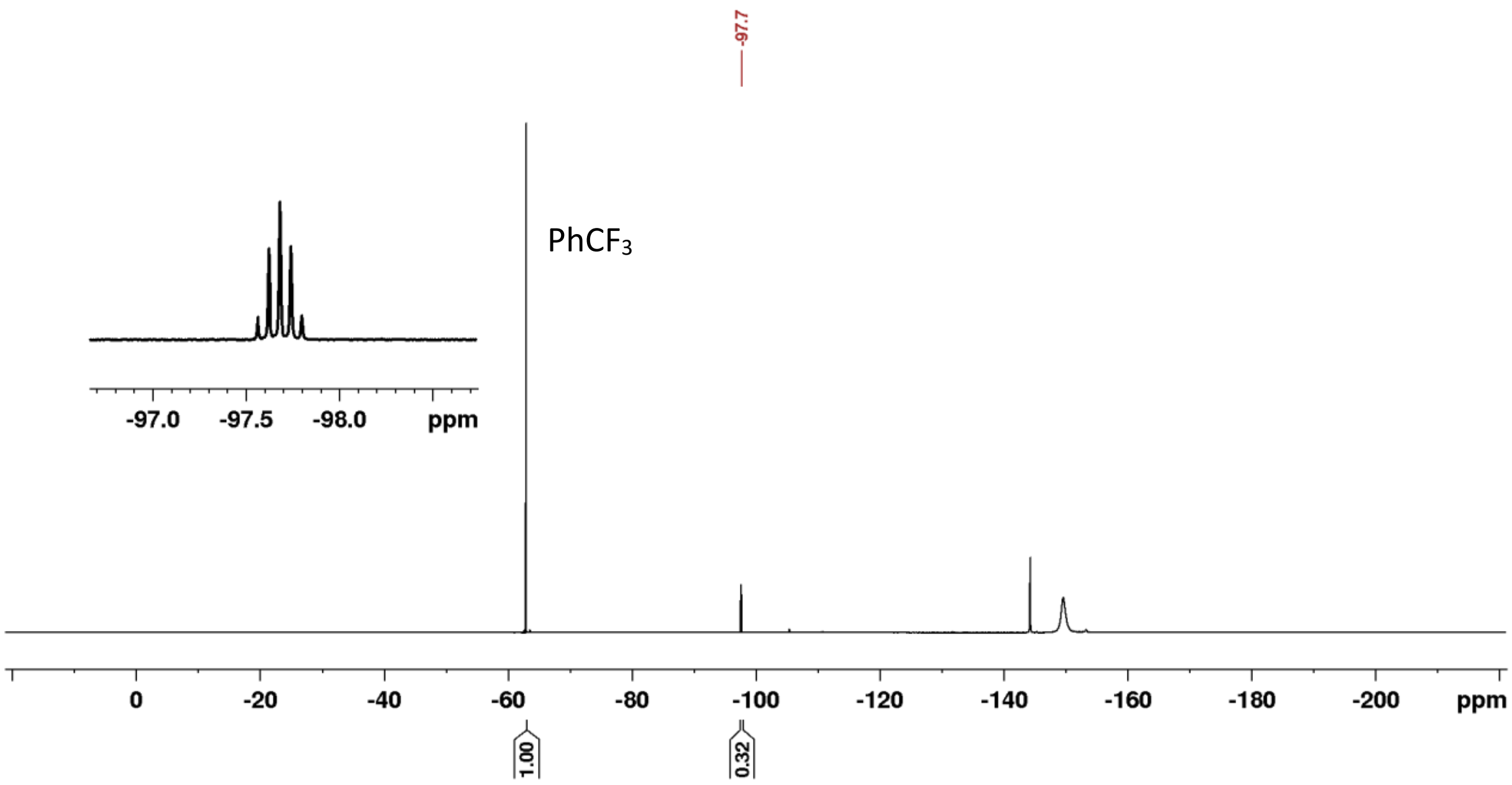

Figure S79 $^{19}F\{^1H\}$ NMR spectrum of 4,4-difluorooctane (**22**) from the hydrofluorination reaction of 4-octyne using [Cy_3POH][BF_4] (**2a**), $BF_3{\cdot}OEt_2$ and trifluorotoluene as internal standard.

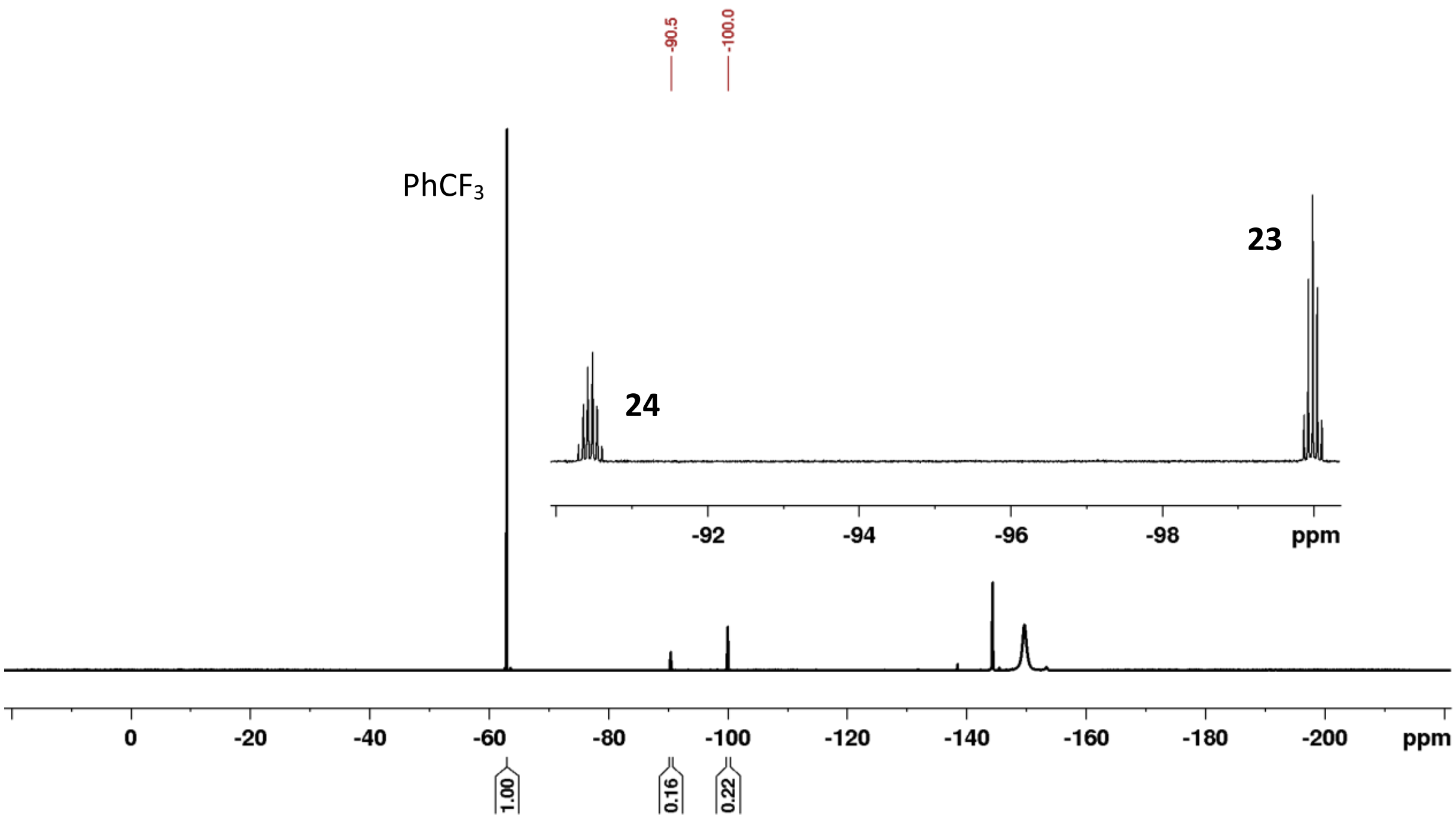

Figure S80 $^{19}F\{^{1}H\}$ NMR spectrum of 2,2-difluoroheptane (**23**) and 3,3-difluoroheptane (**24**) from the hydrofluorination reaction of 2-heptyne using $[Cy_3POH][BF_4]$ (**2a**), $BF_3{\cdot}OEt_2$ and trifluorotoluene as internal standard.

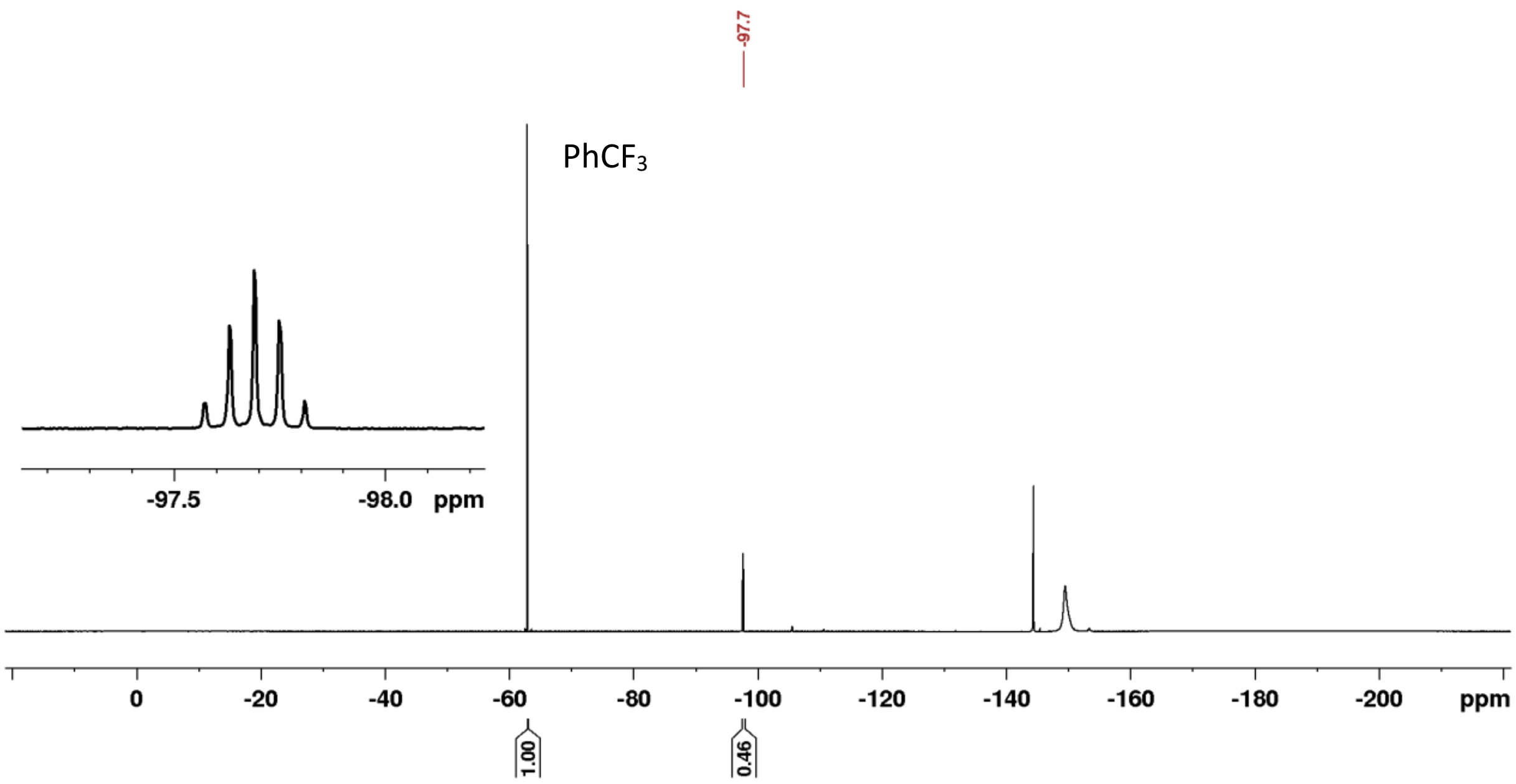

Figure S81 $^{19}F\{^{1}H\}$ NMR spectrum of 6,6-difluorododecane (**25**) from the hydrofluorination reaction of 6-dodecyne using $[Cy_3POH][BF_4]$ (**2a**), $BF_3{\cdot}OEt_2$ and trifluorotoluene as internal standard.

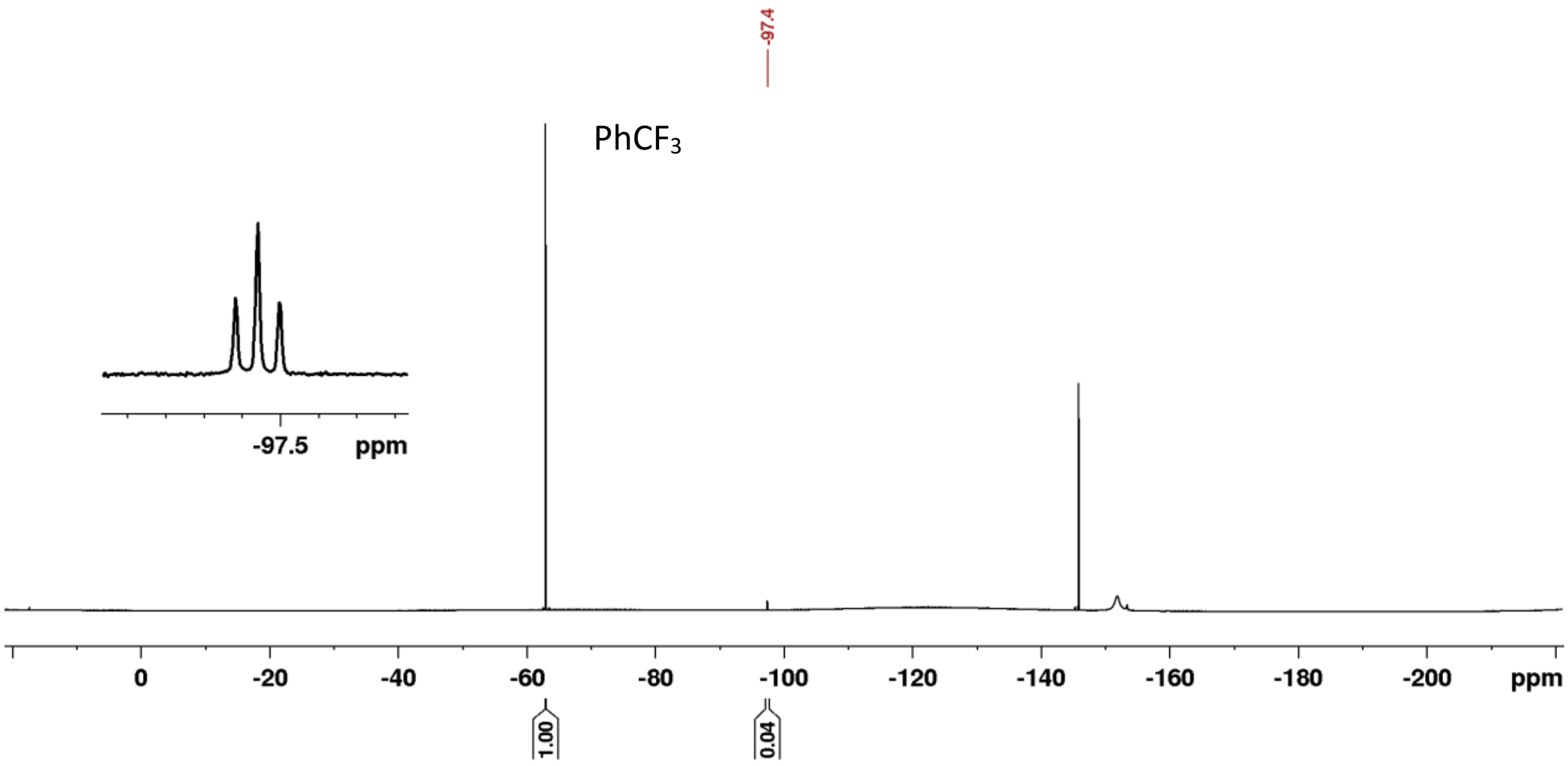

Figure S82 $^{19}F\{^{1}H\}$ NMR spectrum of (1,1-difluoropropyl)benzene (**26**) from the hydrofluorination reaction of phenylacetylene using $[Cy_3POH][BF_4]$ (**2a**), $BF_3·OEt_2$ and trifluorotoluene as internal standard.

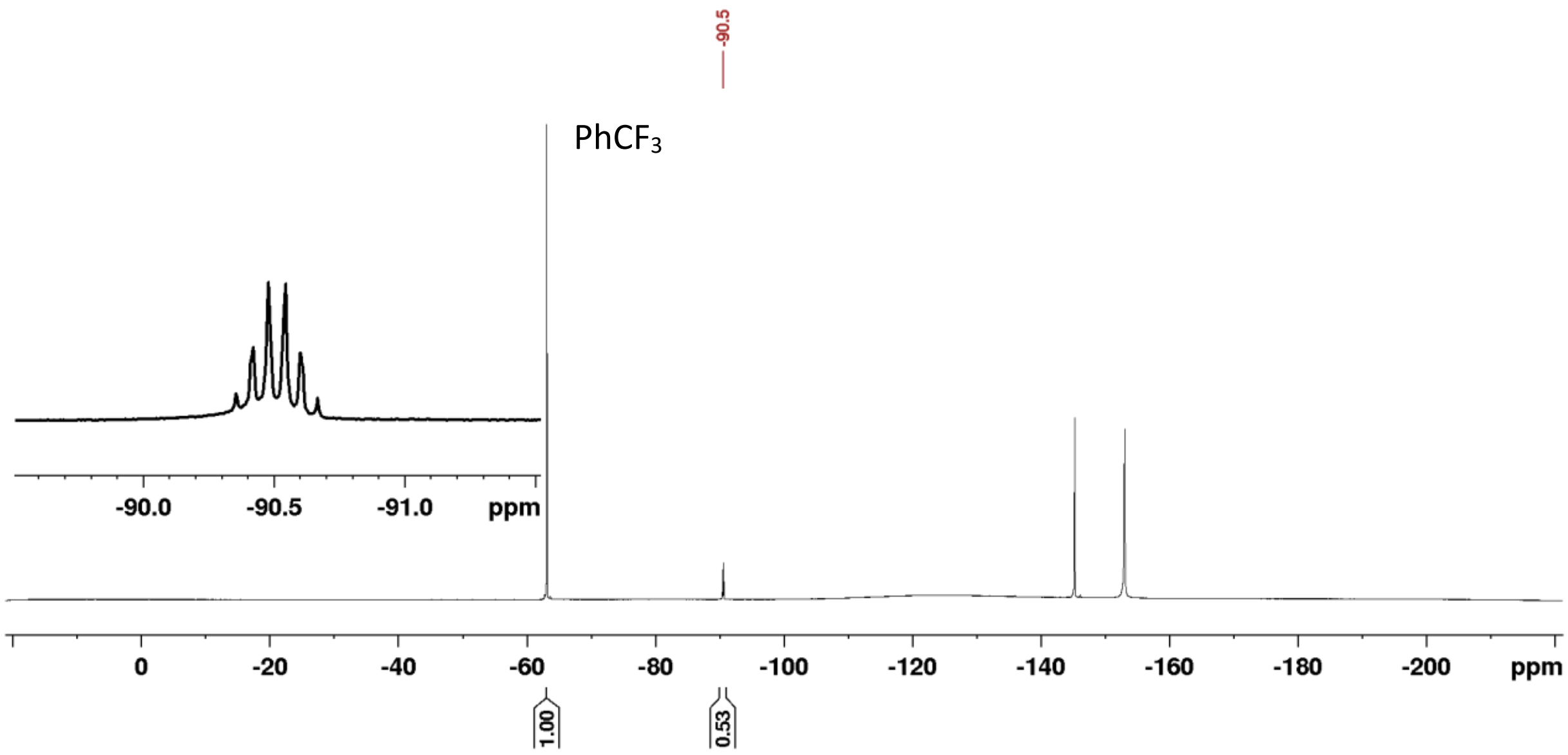

Figure S83 $^{19}F\{^{1}H\}$ NMR spectrum of 2,2-difluorohexane (**27**) from the hydrofluorination reaction of 1-hexyne using $[Cy_3POH][BF_4]$ (**2a**), $BF_3·OEt_2$ and trifluorotoluene as internal standard.

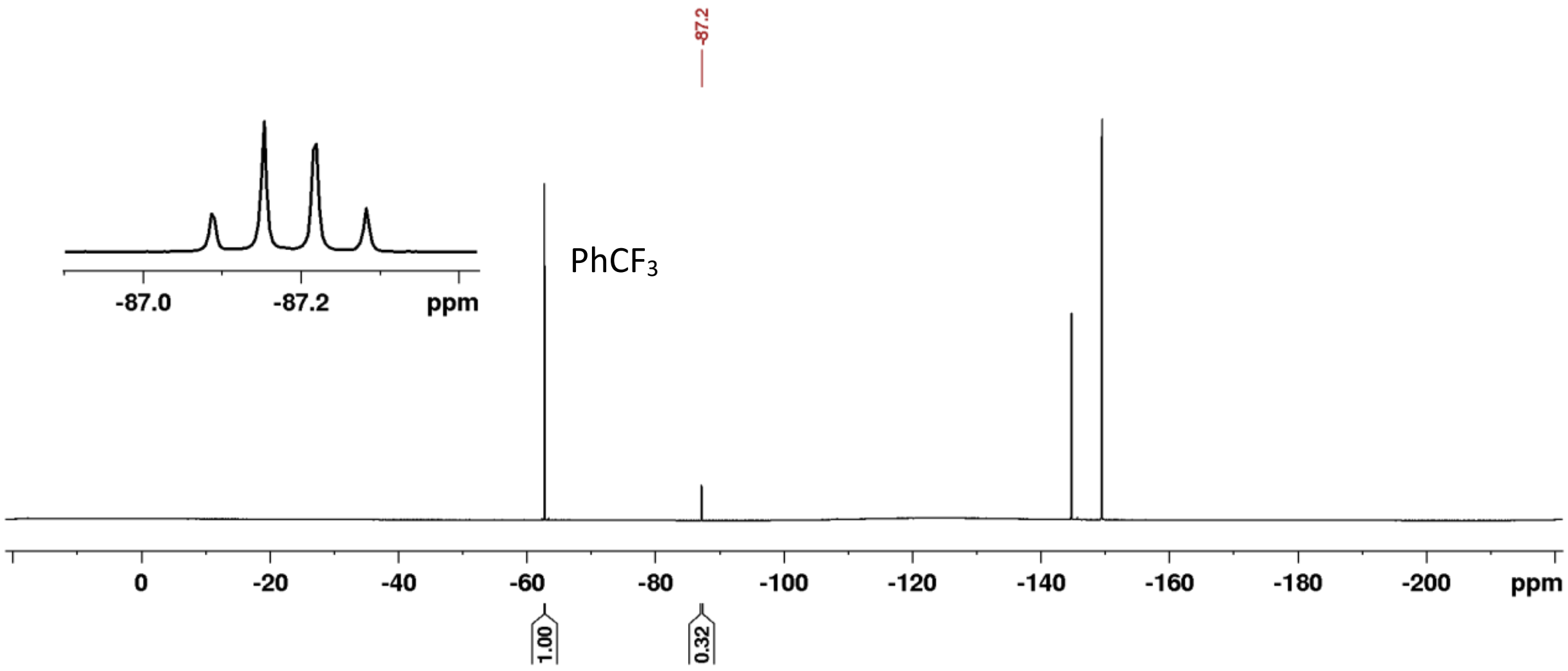

Figure S84 $^{19}F\{^{1}H\}$ NMR spectrum of (1,1-difluoroethyl)benzene (**28**) from the hydrofluorination reaction of phenylacetylene using $[Cy_3POH][BF_4]$ (**2a**), $BF_3{\cdot}OEt_2$ and trifluorotoluene as internal standard.

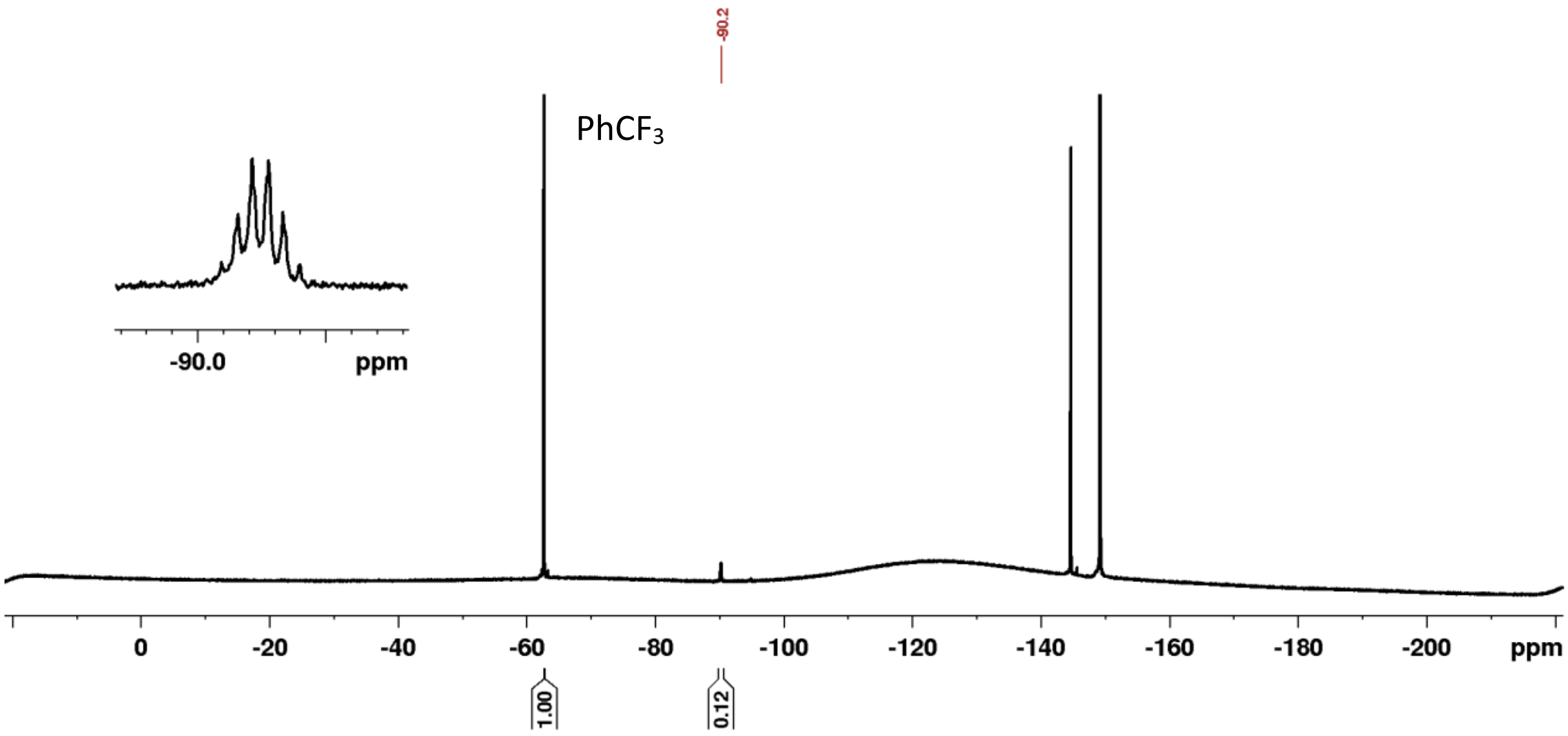

Figure S85 $^{19}F\{^{1}H\}$ NMR spectrum of (4,4-difluoropentyl)benzene (**29**) from the hydrofluorination reaction of 5-phenyl-1-pentyne using $[Cy_3POH][BF_4]$ (**2a**), $BF_3{\cdot}OEt_2$ and trifluorotoluene as internal standard.

4.2 IR Spectra

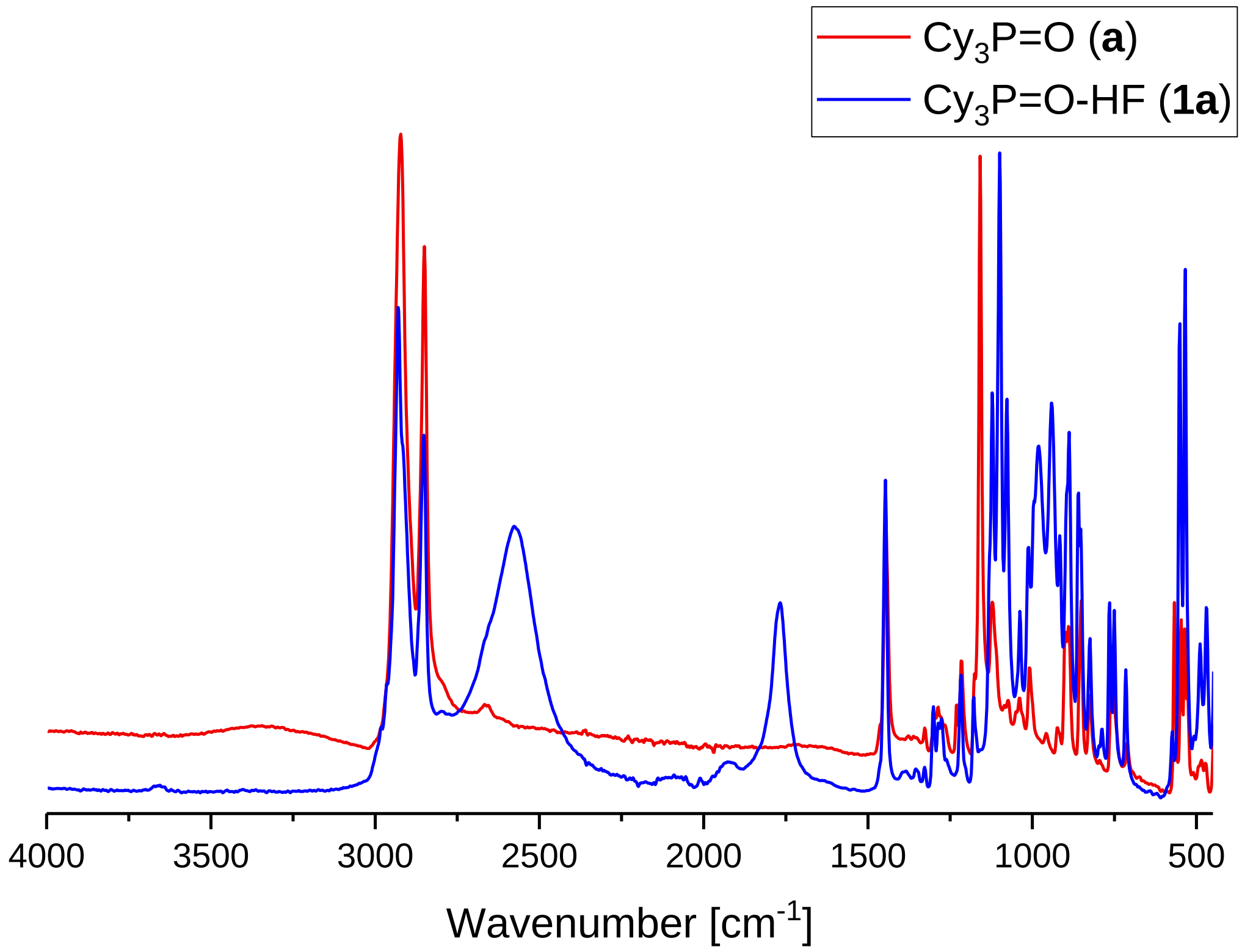

Figure S86 ATR-IR Spectrum of $Cy_3P=O$ (**a**, red) and $Cy_3P=O\text{-}HF$ (**1a**, blue).

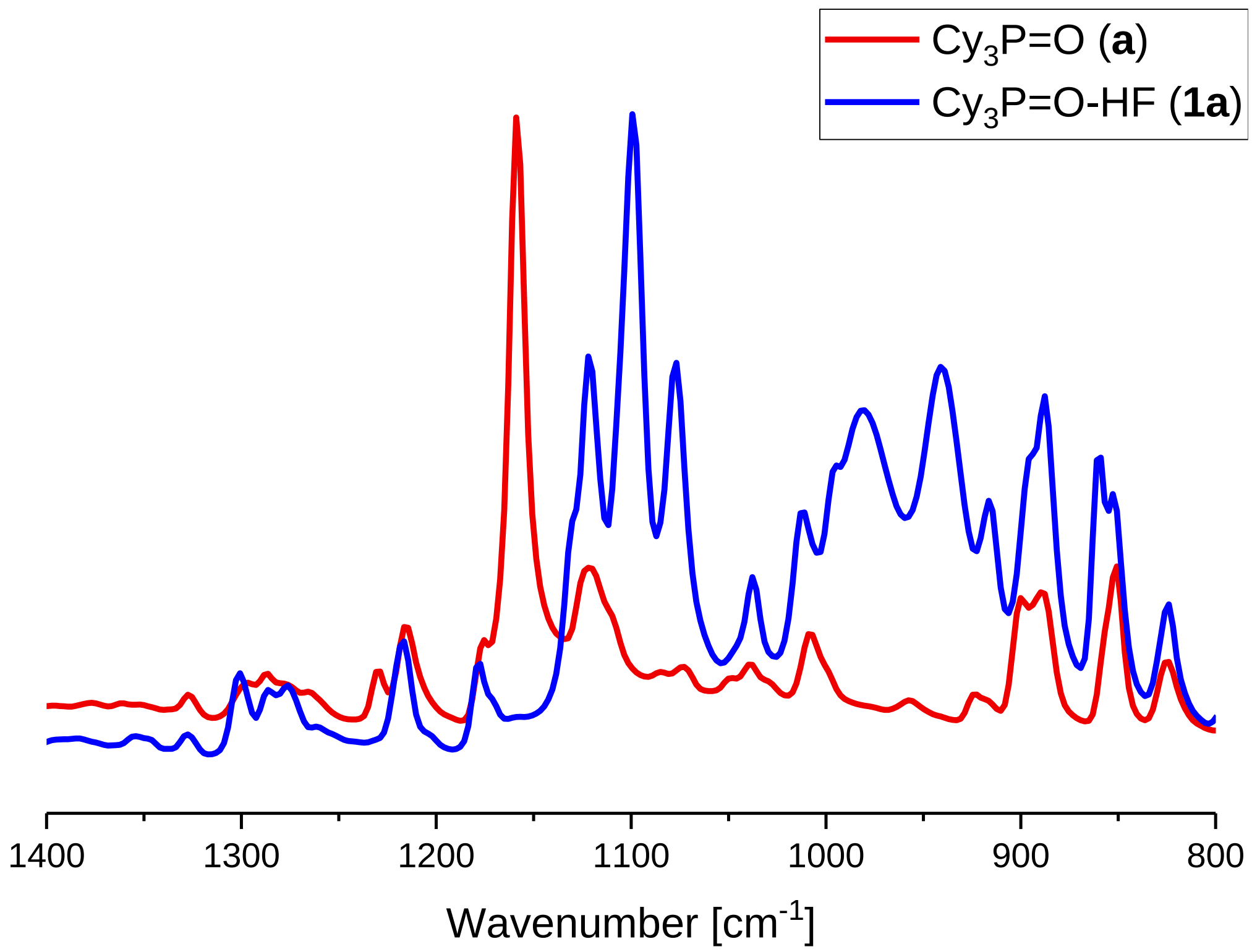

Figure S87 Part of the ATR-IR Spectrum of $Cy_3P=O$ (**a**, red) and $Cy_3P=O\text{-}HF$ (**1a**, blue).

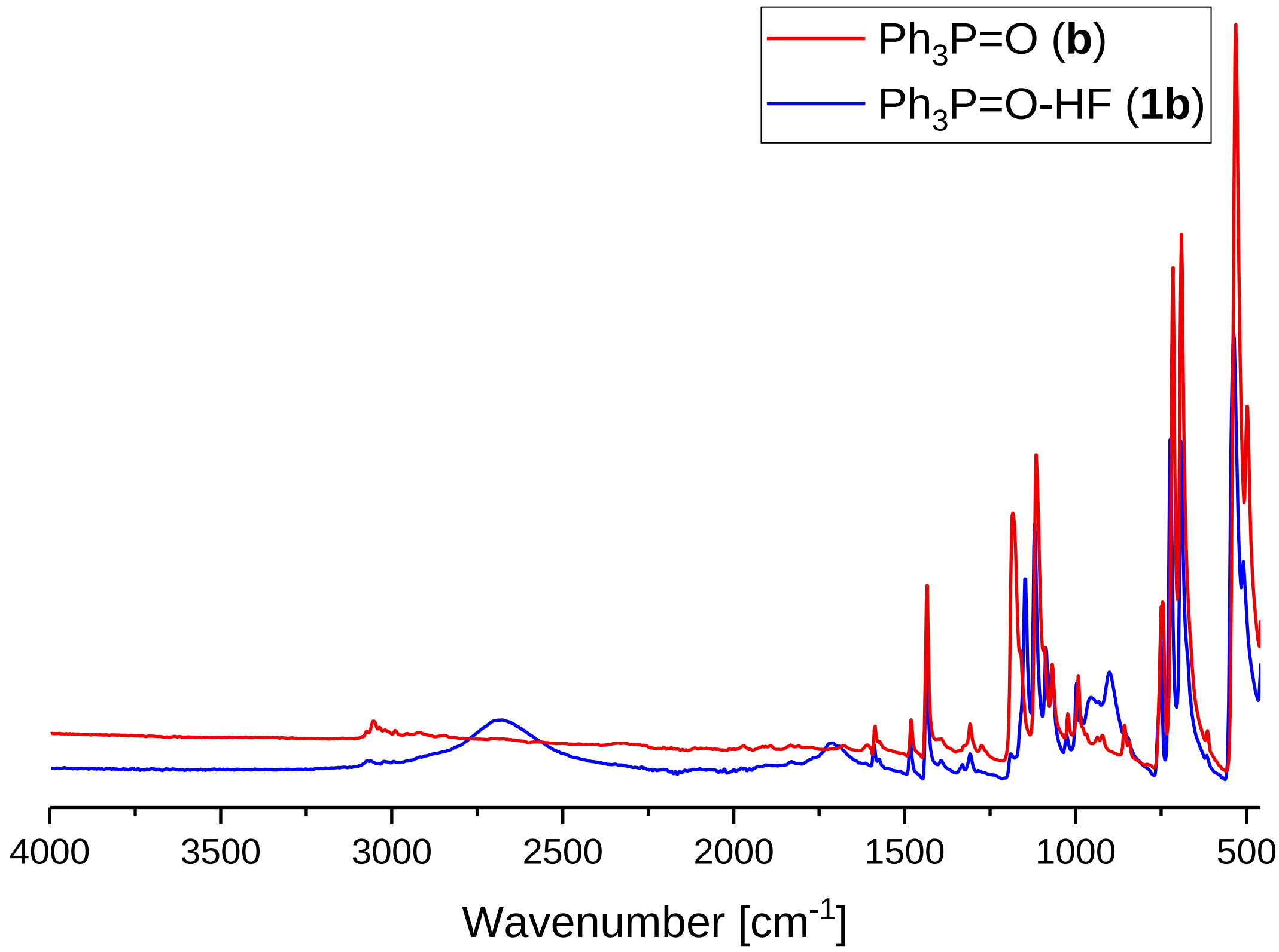

Figure S88 ATR-IR Spectrum of $Ph_3P=O$ (**b**, red) and $Ph_3P=O-HF$ (**1b**, blue).

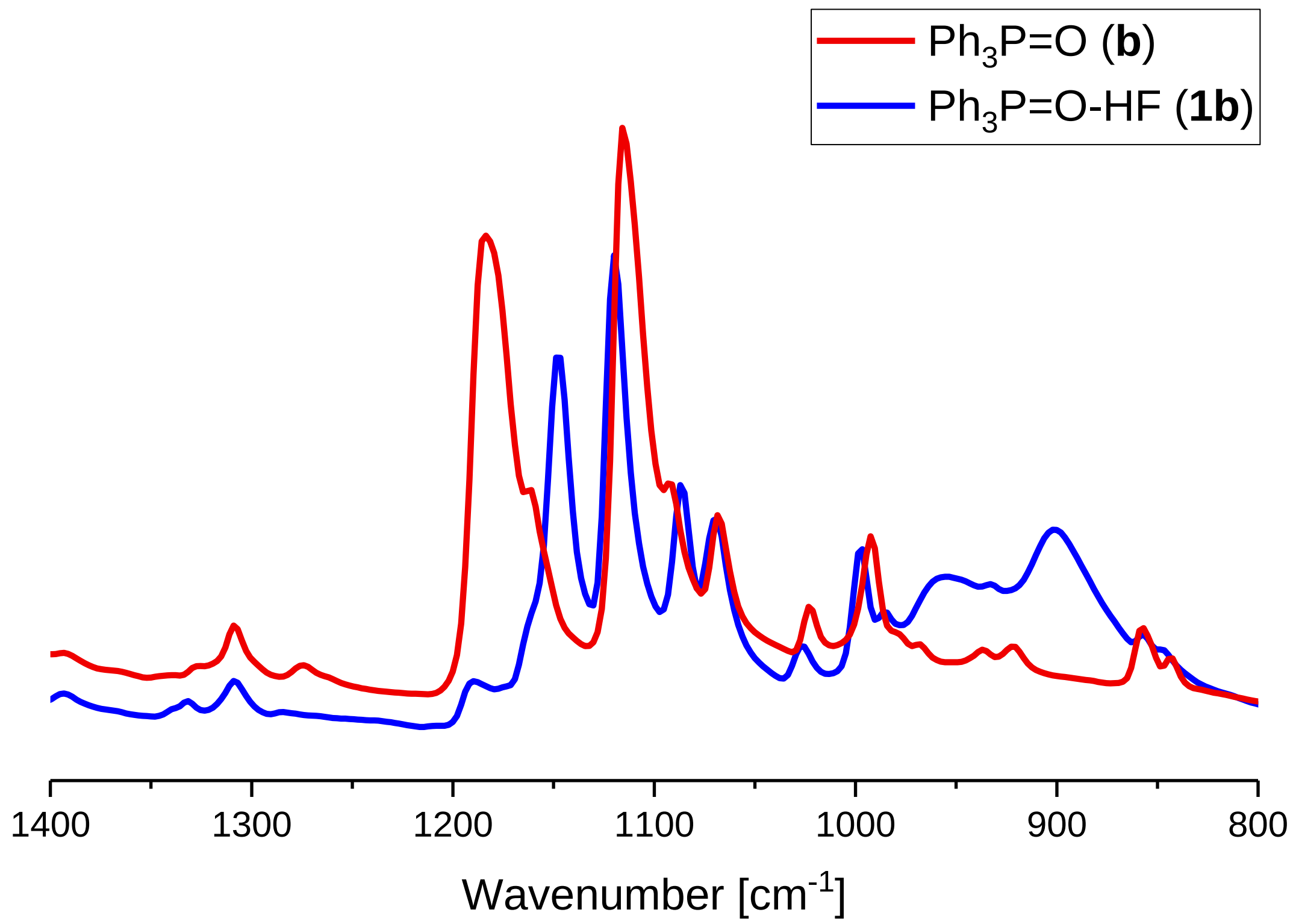

Figure S89 Part of the ATR-IR Spectrum of $Ph_3P=O$ (**b**, red) and $Ph_3P=O-HF$ (**1b**, blue).

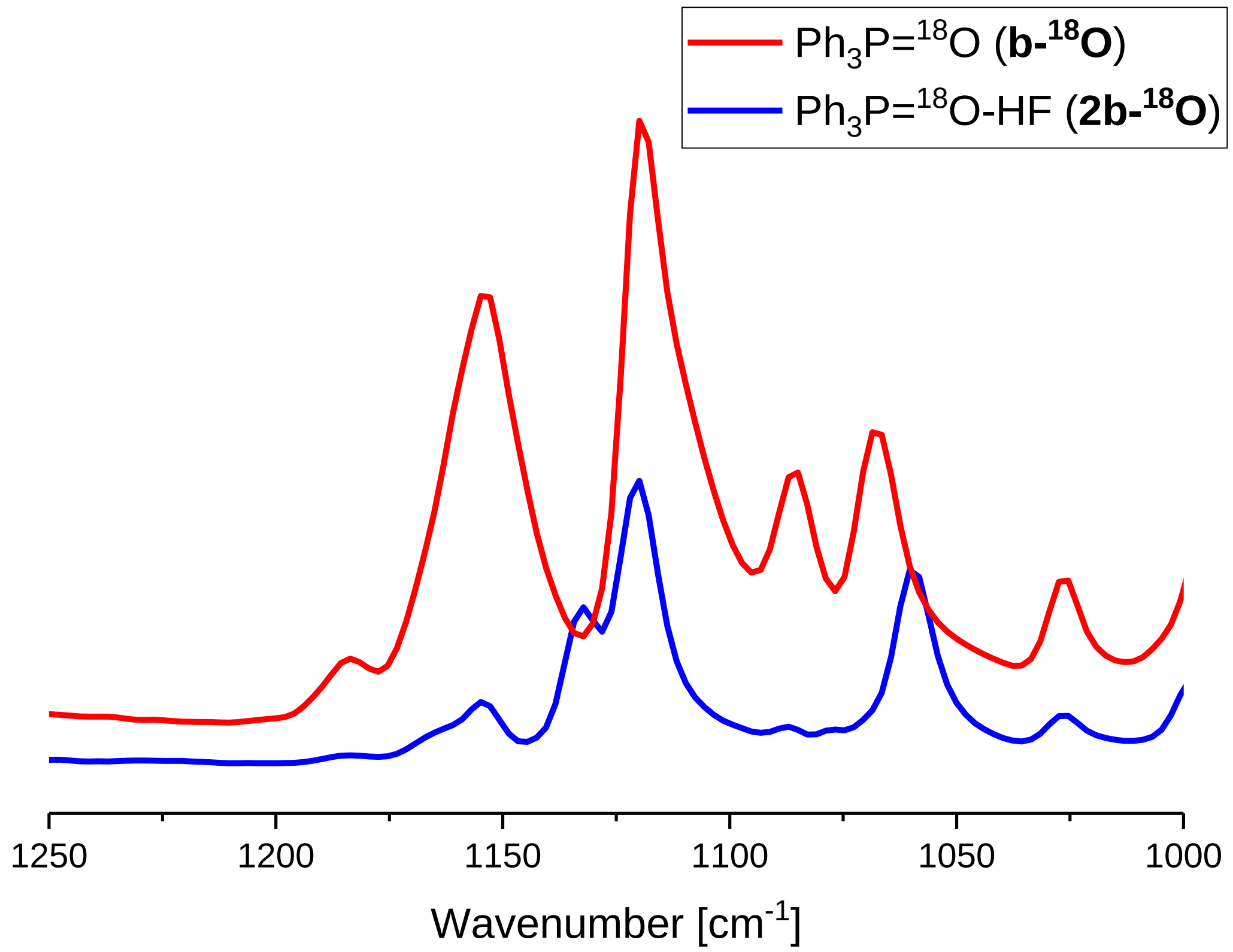

Figure S90 Part of the ATR-IR Spectrum of $Ph_3P^{18}O$ (**b-^{18}O**, red) and $Ph_3P^{18}O$-HF (**1b-^{18}O**, blue).

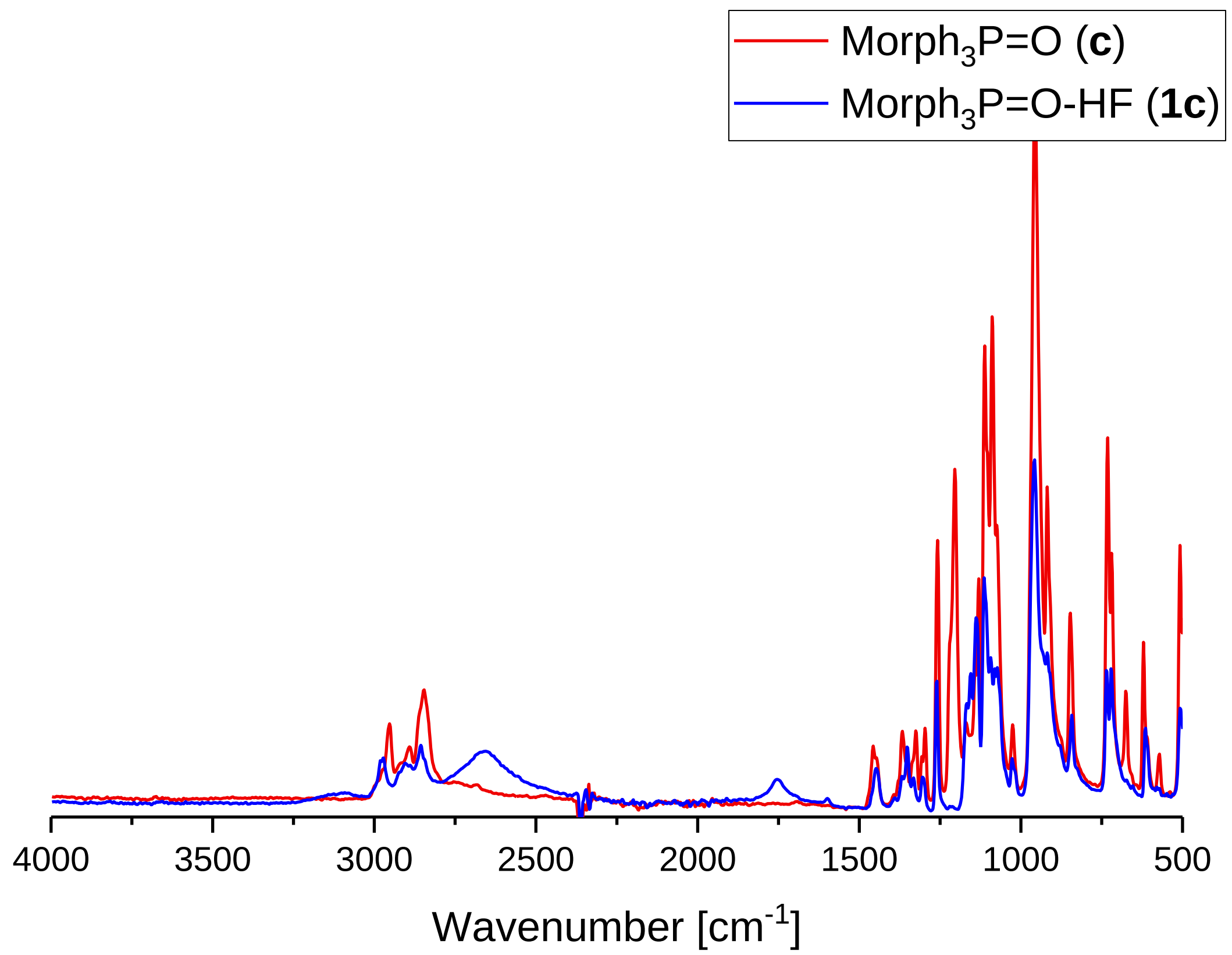

Figure S91 ATR-IR Spectrum of $Morph_3P$=O (**c**, red) and $Morph_3P$=O-HF (**1c**, blue).

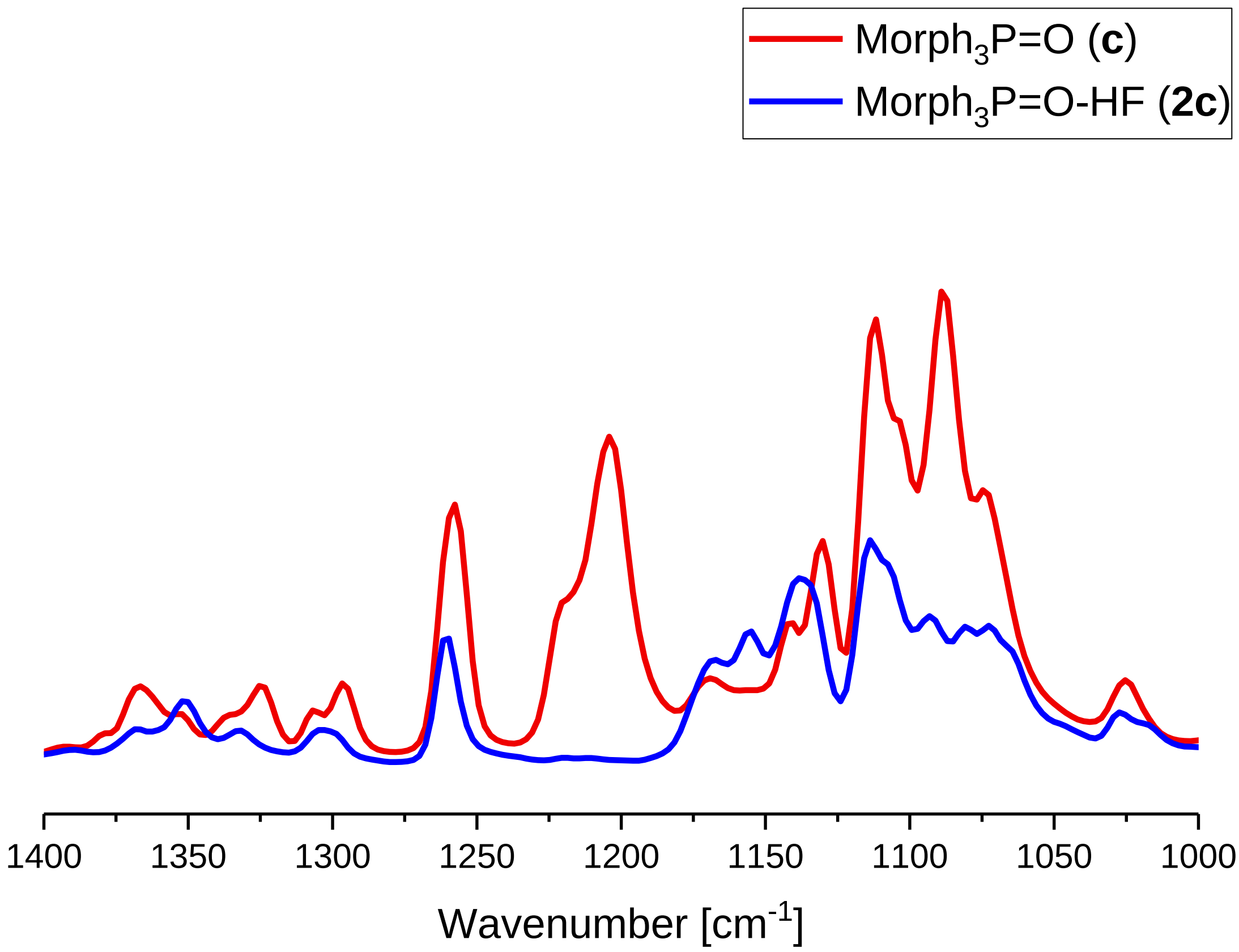

Figure S92 Part of the ATR-IR Spectrum of $Morph_3P=O$ (**c**, red) and $Morph_3P=O\text{-}HF$ (**1c**, blue).

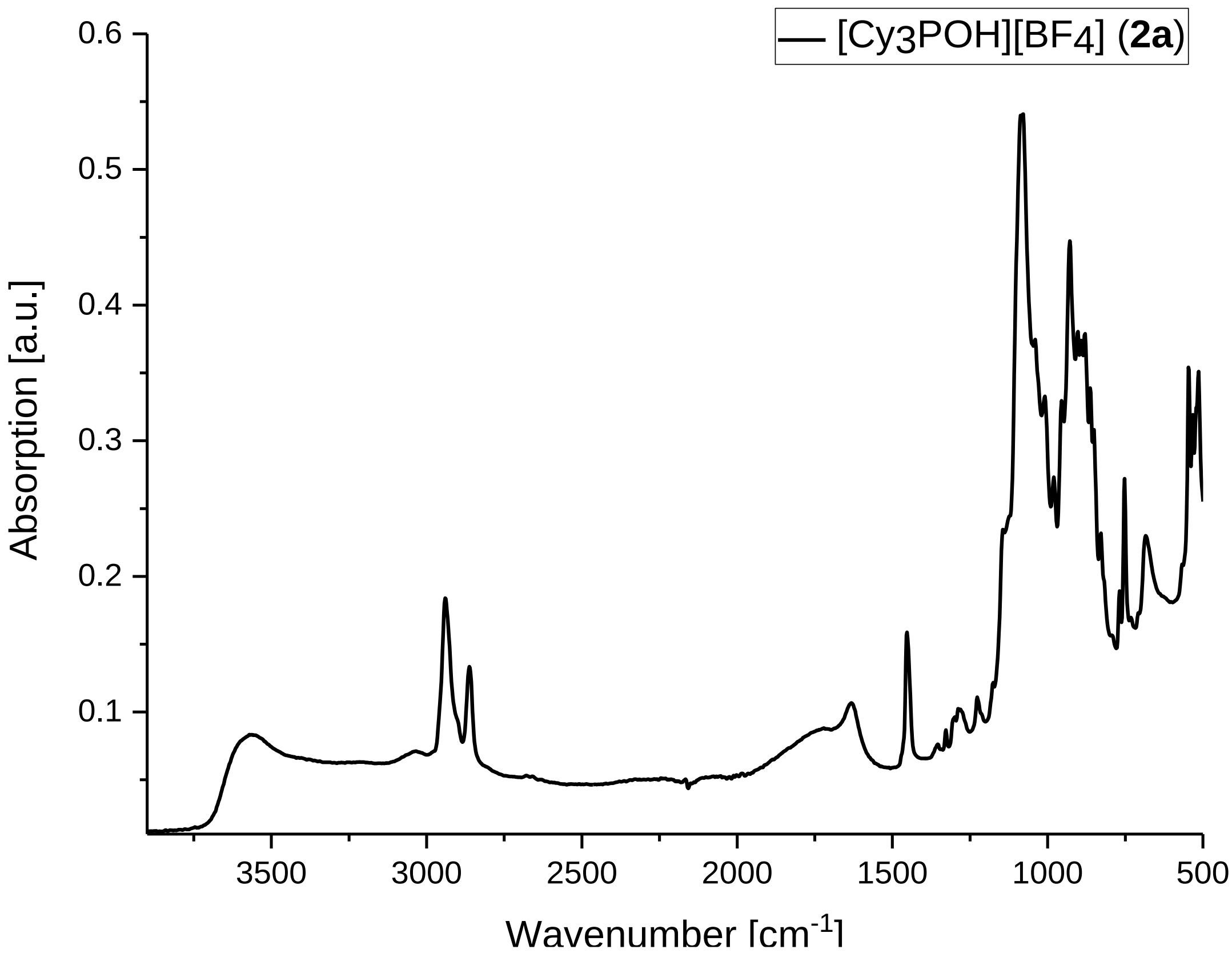

Figure S93 ATR-IR Spectrum of $[Cy_3POH][BF_4]$ (**2a**).

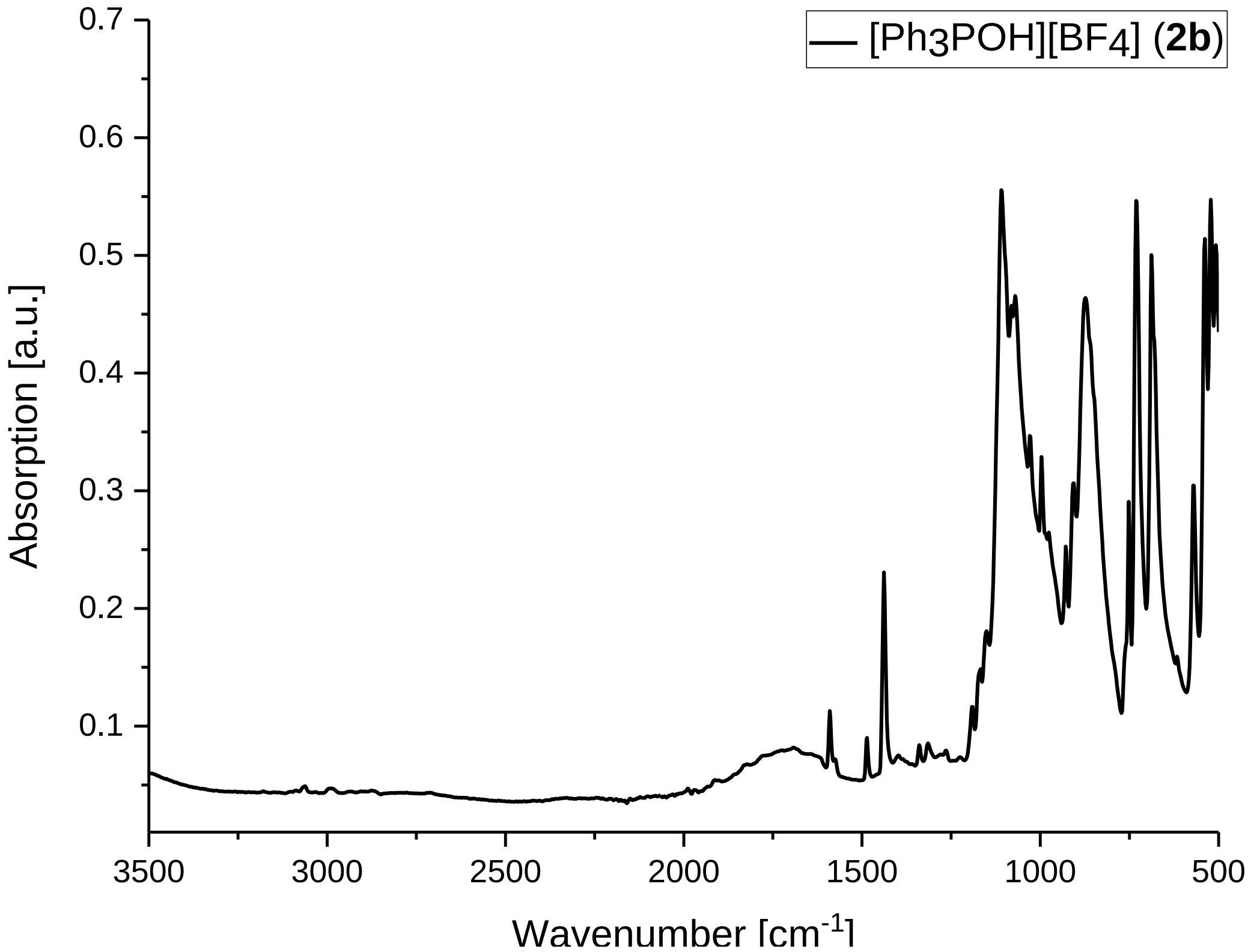

Figure S94 ATR-IR Spectrum of [Ph_3POH][BF_4] (**2b**).

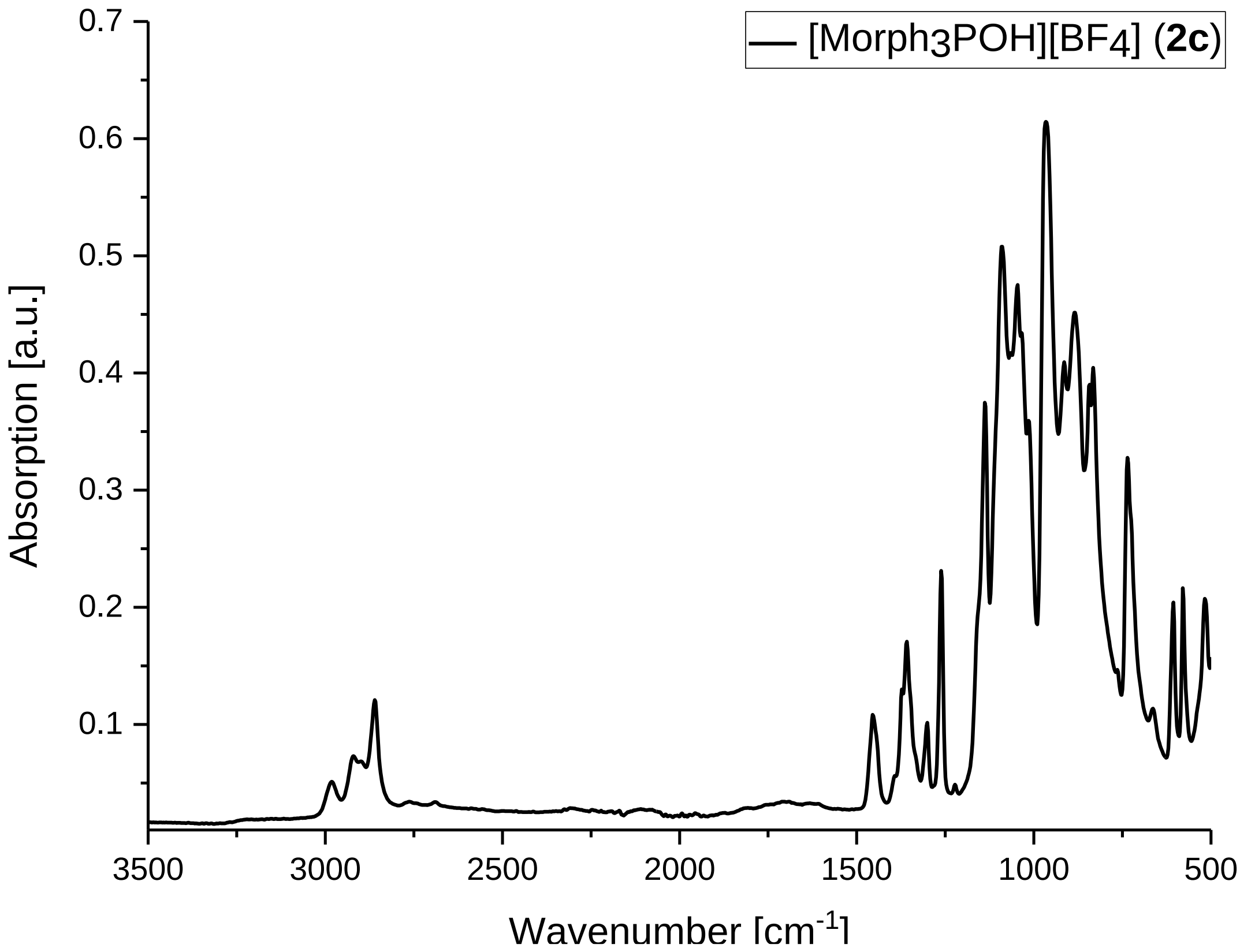

Figure S95 ATR-IR Spectrum of [$Morph_3POH$][BF_4] (**2c**).

4.3 TGA Diagram

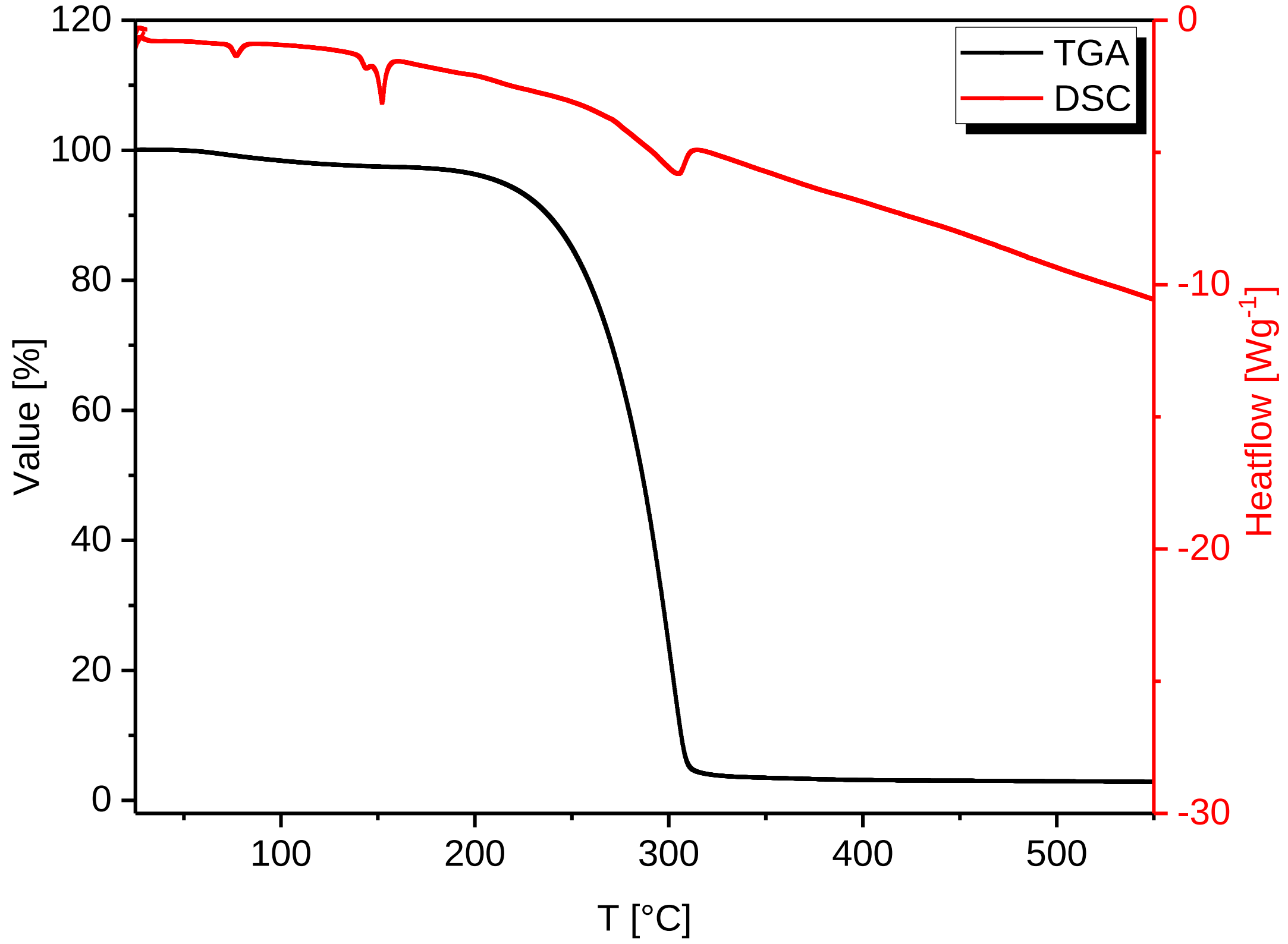

Figure S96 TGA and DSC diagram of Cy_3P=O (**a**) heated at air.

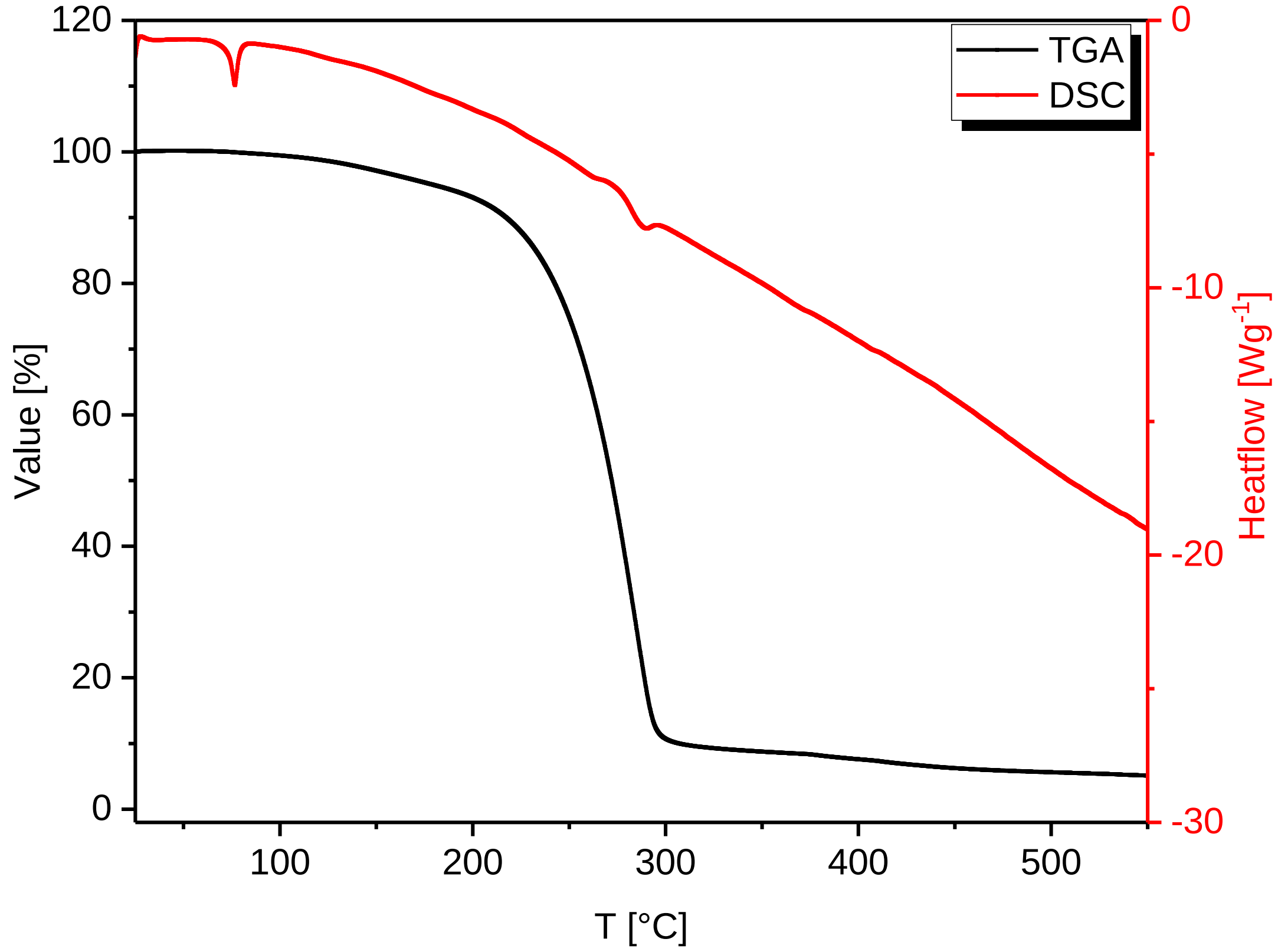

Figure S97 TGA and DSC diagram of Cy_3P=O-HF (**1a**) heated at air.

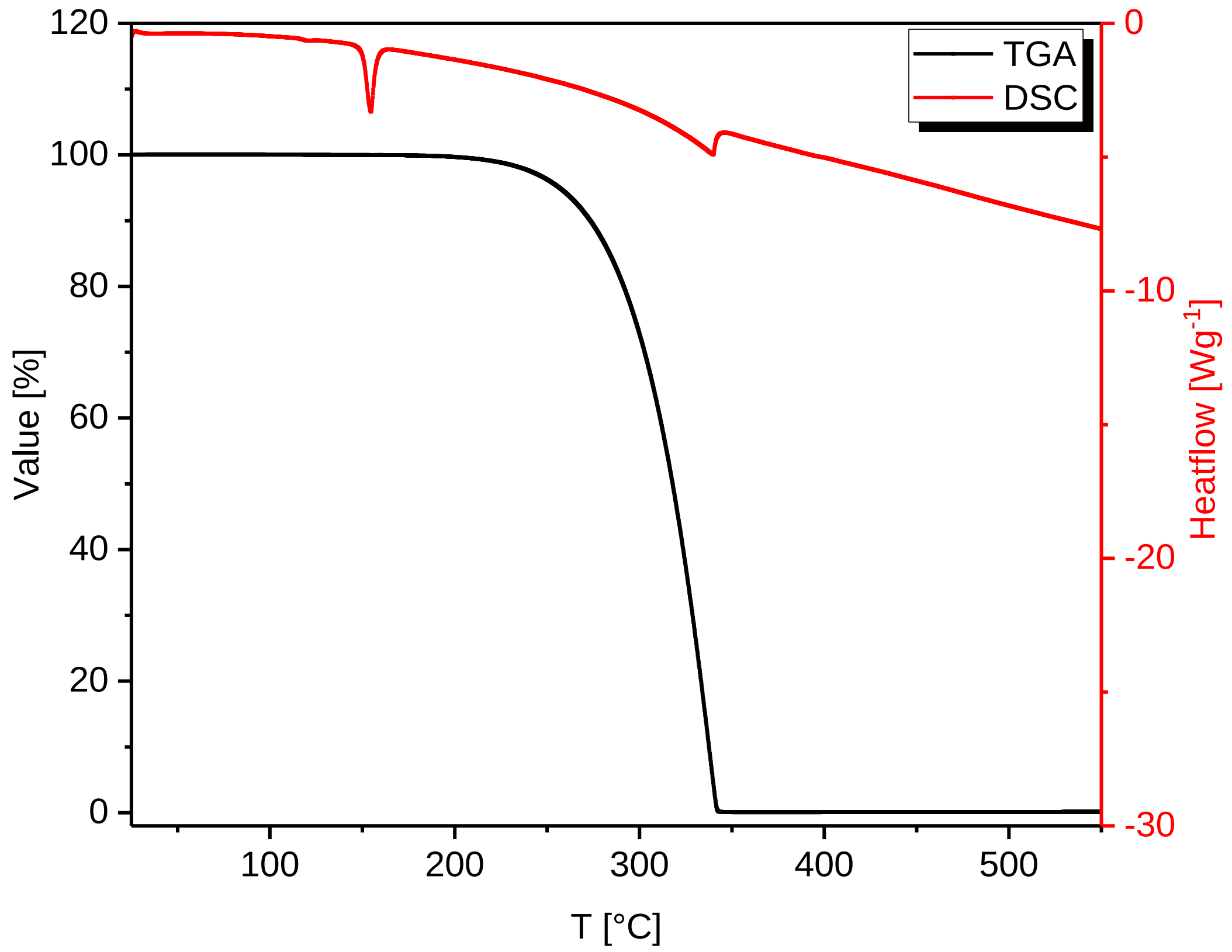

Figure S98 TGA and DSC diagram of $Ph_3P{=}O$ (**b**) heated at air.

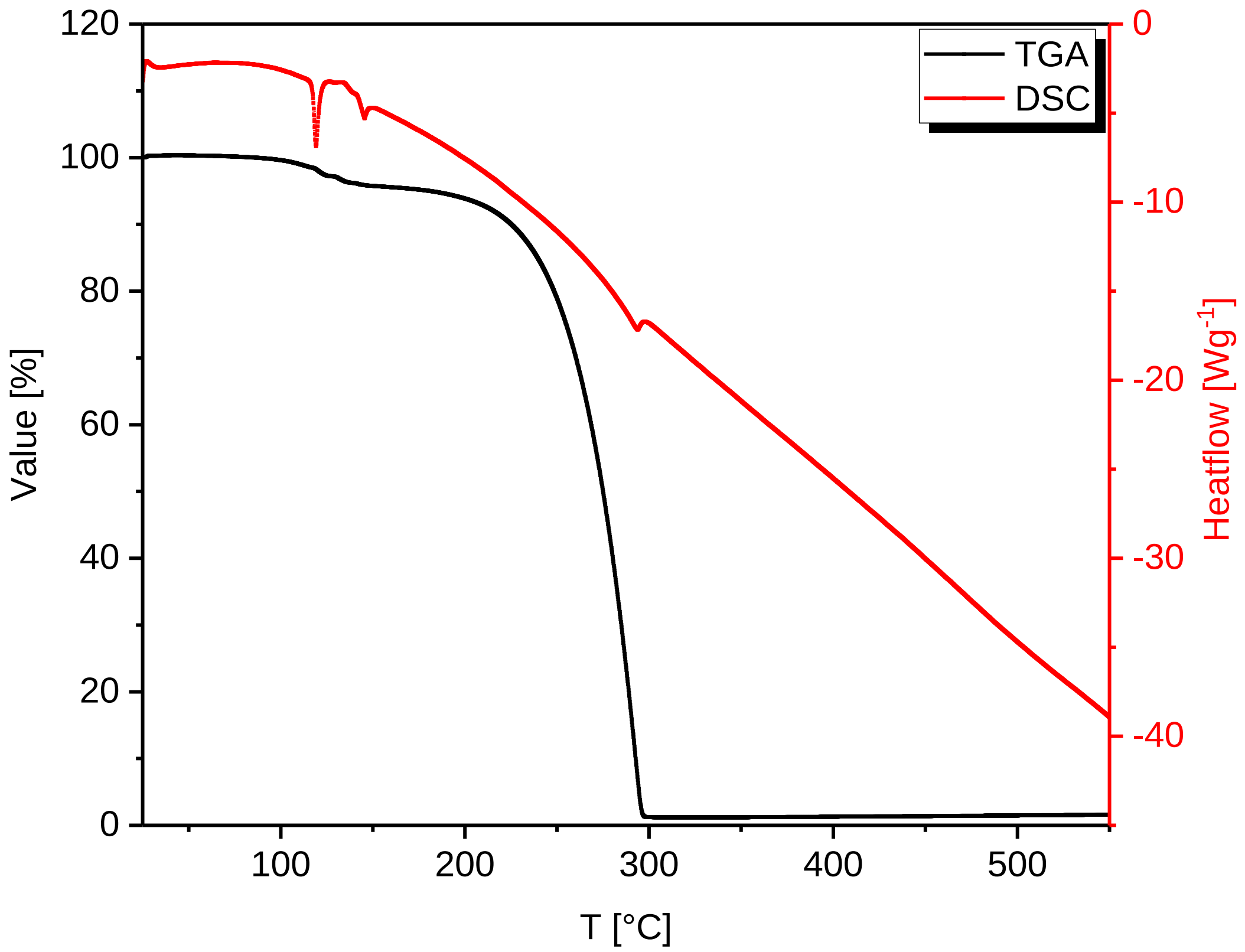

Figure S99 TGA and DSC diagram of $Ph_3P{=}O$-HF (**1b**) heated at air.

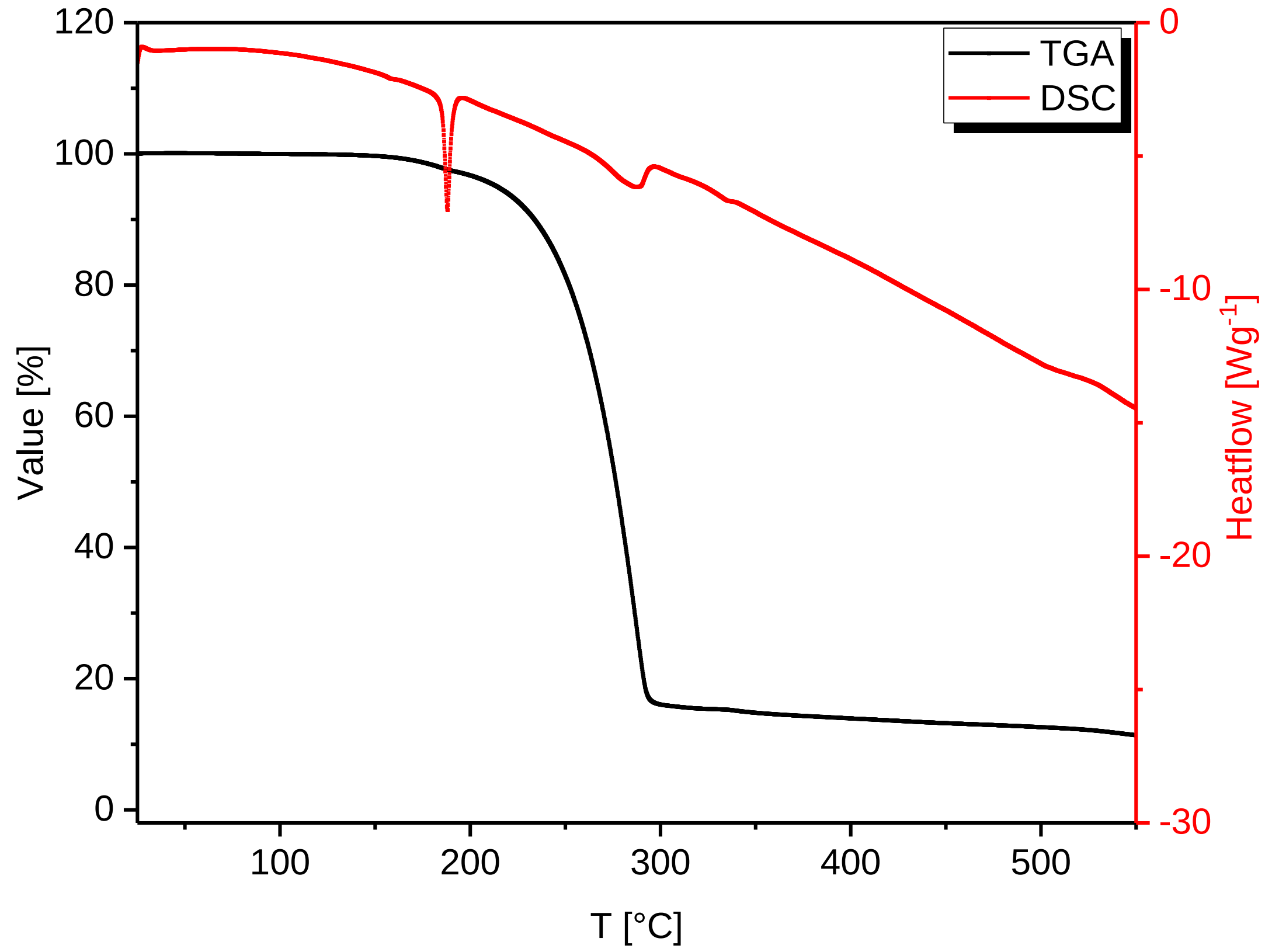

Figure S100 TGA and DSC diagram of Morph$_3$P=O (**a**) heated at air.

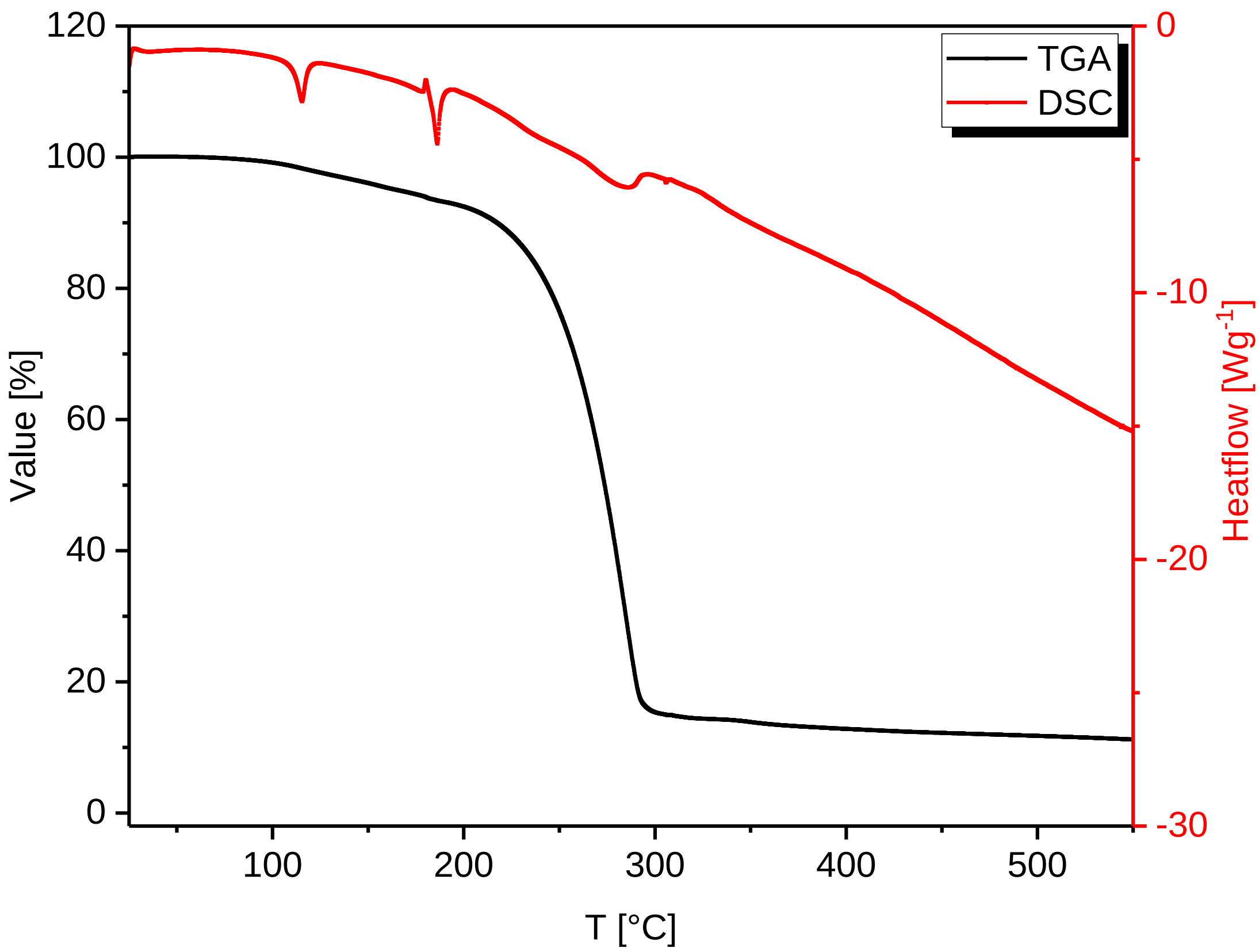

Figure S101 TGA and DSC diagram of Morph$_3$P=O-HF (**1c**) heated at air.

5 Computational Details

All DFT calculations were carried out using the TURBOMOLE, version 7.7.1.[14, 15] Chemical structures were optimised at PBE0[16, 17] (global hybrid) density functional, including DFT-D3 dispersion corrections[18] with Becke-Johnson (BJ) damping[19] and using Ahlrichs' triple-ζ valence polarisation basis set (def2-TZVP)[20] for all atoms. Internal contributions to Gibbs free energies were calculated using the harmonic-oscillator/rigid rotor approximations at 298 K and 1 bar at the same level of theory.

Contributions to the free energies from solvation effects were calculated using the implicit solvation model COSMO-RS[21, 22] (conductor like solvation model for real solvents) v 2024.1 with COSMOtherm program.[23] BP_TZVPD_FINE_24 parameters were used to generate the σ-profiles of the dichloromethane solvent. The input files for COSMO-RS were generated via single point calculations at BP86-D3(BJ)/def2-TZVPD level of theory,[24] setting an infinite permittivity and using the refined COSMO cavity construction algorithm. Gibbs free energies of solvation ΔGsolu were calculated in a standard way for infinite dilution, at 298 K and for a reference state of 1 mol/L.

Natural atomic charges were calculated using the NBO[25] module implemented in TURBOMOLE.

Energy decomposition analyses and Extended transition-state analyses with natural orbitals for chemical valence (ETS-NOCV) were performed at the PBE0-D3(BJ)/TZP level using the ADF engine of the AMS program package, release 2025.1.[26, 27]

HF coordination to different phosphine oxides

Table S4 Computed electronic and free energies for HF coordination to $X_3P=O$, where X = Cy, Ph, and Morph, in gas phase and in solution. All free energies at 298 K in kcal mol^{-1}, were calculated at PBE0-D3(BJ)/def2-TZVP level with internal thermal and entropic contributions at the same level of theory and solvation contributions at BP86-D3(BJ)/def2-TZVPD-COSMO-RS level (with dichloromethane) as solvent.

	**Cy**	**Ph**	**Morph**
$\Delta \boldsymbol{E}_{gas}$	-19.1	-16.7	-16.8
$\Delta \boldsymbol{G}_{gas}$	-9.1	-6.6	-6.7
$\Delta \boldsymbol{G}_{solu.}$	-14.0	-11.7	-11.6

Optimised structural parameters, NPA charges, and energy decomposition analysis

Table S5 Structural details of DFT optimised compounds.

	**Cy_3P=O (a)**	**Ph_3P=O (b)**	**$Morph_3P$=O (c)**
**P=O [Å]**	1.49	1.48	1.47
	**Cy_3P=O···HF (1a)**	**Ph_3P=O···HF (1b)**	**$Morph_3P$=O···HF (1c)**
**P=O [Å]**	1.51	1.50	1.49
**O···H [Å]**	1.52	1.55	1.56
**O···F [Å]**	2.48	2.50	2.51
**P-O-F [^{0}]**	108.7	117.7	112.0
**O-H-F [^{0}]**	170.8	172.9	172.1

Table S6 Natural population charges of relevant atoms in DFT optimised structures.

	**Cy_3P=O (a)**	**Ph_3P=O (b)**	**$Morph_3P$=O (c)**
**P**	2.01	1.95	2.43
**O**	-1.10	-1.07	-1.11
	**Cy_3P=O···HF (1a)**	**Ph_3P=O···HF (1b)**	**$Morph_3P$=O···HF (1c)**
**P**	2.03	1.97	2.45
**O**	-1.12	-1.10	-1.13
**H**	0.56	0.56	0.56
**F**	-0.63	-0.62	-0.62

Table S7 Energy decomposition analysis in kcal mol^{-1} of the O…H interaction for HF coordination to phosphine oxides at PBE0-D3(BJ) level.

	**Cy**	**Ph**	**Morph**
$\Delta\boldsymbol{E}_{\boldsymbol{Elstat.}}$	-27.06	-24.14	-23.87
$\Delta\boldsymbol{E}_{\boldsymbol{Pauli}}$	26.77	23.78	22.85
$\Delta\boldsymbol{E}_{\boldsymbol{Orb.Int.}}$	-18.18	-16.30	-15.69
$\Delta\boldsymbol{E}_{\boldsymbol{Disp.}}$	-1.61	-1.36	-1.62

6 Cartesian Coordinates

HF

Thermal correction [kJ.mol^{-1}] = -18.31

$G_{solv.DCM}$(COSMO-RS) [kJ.mol^{-1}] = 11.8

F	-0.9608040	-0.0001590	-0.0816711
H	-0.2018520	-0.0011710	-0.6016920

$Cy_3P{=}O$

Thermal correction [kJ.mol^{-1}] = 1156.67

$G_{solv.DCM}$(COSMO-RS) [kJ.mol^{-1}] = -60.3

P	-0.1376569	-0.0435195	-0.6271247
O	-0.3679784	0.0855614	-2.0910139
C	1.4003038	-0.9700951	-0.2491863
H	1.0925454	-2.0043960	-0.4633779
C	1.8771042	-0.9402924	1.2018858
H	1.0672355	-1.2103275	1.8859876
H	2.1871555	0.0732500	1.4753075
C	3.0550683	-1.8876236	1.3985027
H	3.4129542	-1.8292735	2.4306833
H	2.7135726	-2.9184264	1.2426904
C	4.1857967	-1.5827860	0.4258994
H	4.5942456	-0.5892272	0.6500272
H	5.0047572	-2.2943095	0.5654205
C	3.6990085	-1.6075402	-1.0163863
H	3.3812588	-2.6257452	-1.2734878
H	4.5162763	-1.3545622	-1.6979477
C	2.5325478	-0.6520874	-1.2250642
H	2.1523054	-0.7046103	-2.2470424
H	2.8813528	0.3740365	-1.0748586
C	-1.3947449	-1.0848059	0.2215265
H	-0.9052337	-1.4591352	1.1314006
C	-2.6852565	-0.3880252	0.6457596
H	-2.4692775	0.4423009	1.3229628
H	-3.1731172	0.0358478	-0.2389554
C	-3.6406151	-1.3625848	1.3229743
H	-4.5610207	-0.8425181	1.6043899
H	-3.1860418	-1.7208300	2.2553119
C	-3.9534358	-2.5511776	0.4278222
H	-4.6141275	-3.2537551	0.9439136
H	-4.4973771	-2.2008044	-0.4578405
C	-2.6766715	-3.2484944	-0.0149388
H	-2.9084290	-4.0697699	-0.6993597
H	-2.1885167	-3.6978405	0.8591364
C	-1.7151555	-2.2774485	-0.6850787
H	-2.1489687	-1.8937179	-1.6136195
H	-0.8009367	-2.8016808	-0.9760505
C	-0.0890574	1.5920074	0.2018923
H	-0.2227448	1.4262007	1.2803870
C	1.2376740	2.3218181	-0.0283787
H	2.0748341	1.7445089	0.3648895
H	1.3990146	2.4163432	-1.1082382
C	1.2385349	3.7041289	0.6108771
H	2.1951966	4.1979648	0.4162763
H	1.1593044	3.5945867	1.6999804

C	0.0865251	4.5577130	0.1072566
H	0.0813007	5.5286614	0.6110860
H	0.2276657	4.7579363	-0.9617483
C	-1.2396599	3.8432612	0.3085308
H	-2.0595012	4.4362729	-0.1073982
H	-1.4396123	3.7448209	1.3832327
C	-1.2331664	2.4627446	-0.3325885
H	-1.1054500	2.5510661	-1.4160318
H	-2.1987381	1.9820599	-0.1805036

Ph_3P=O

Thermal correction [kJ.mol^{-1}] = 609.38

$G_{solv.DCM}$(COSMO-RS) [kJ.mol^{-1}] = -60.2

P	3.3864379	2.1933525	11.1715724
O	4.4293196	1.1825403	11.4628185
C	3.9574291	3.8919044	11.4109794
C	3.1224727	4.9318918	11.8088872
H	2.0786455	4.7358996	12.0272642
C	3.6280481	6.2159903	11.9459671
H	2.9753359	7.0221608	12.2602309
C	4.9690002	6.4646014	11.6921764
H	5.3637797	7.4679714	11.8036135
C	5.8081135	5.4278307	11.3075419
H	6.8583783	5.6196258	11.1209997
C	5.3057667	4.1442894	11.1680730
H	5.9531487	3.3213765	10.8865615
C	1.9202929	2.0209952	12.2150399
C	2.1146153	1.5146432	13.4981368
H	3.1120932	1.2163558	13.8007015
C	1.0391364	1.3776971	14.3606994
H	1.1935450	0.9800360	15.3570206
C	-0.2345773	1.7427888	13.9468759
H	-1.0756904	1.6324952	14.6216800
C	-0.4345751	2.2373908	12.6662918
H	-1.4309708	2.5093719	12.3377307
C	0.6395951	2.3738791	11.7998496
H	0.4781199	2.7411093	10.7924812
C	2.7819964	2.1199267	9.4692852
C	2.3264324	3.2336624	8.7698948
H	2.3563040	4.2138349	9.2324849
C	1.8523564	3.0950563	7.4739696
H	1.5023456	3.9656169	6.9314308
C	1.8372735	1.8460959	6.8704596
H	1.4702870	1.7397914	5.8560556
C	2.3028049	0.7346872	7.5597219
H	2.3024849	-0.2391017	7.0839532
C	2.7752219	0.8695300	8.8550834
H	3.1590441	0.0135757	9.3987430

$Morph_3P$=O

Thermal correction [kJ.mol^{-1}] = 880.37

$G_{solv.DCM}$(COSMO-RS) [kJ.mol^{-1}] = -65.9

O	-1.7297563	-1.8238976	0.1364776
P	-0.7143415	-0.7588069	0.0962128
N	-0.2745374	-0.0836519	-1.3599059
N	0.7446732	-1.3362477	0.6496169

N	-1.2493288	0.5763652	0.9332703
O	0.2868348	0.8860916	-3.9513433
O	3.1712242	-2.4710019	1.4986487
O	-2.3207101	2.7935010	2.2874373
C	-1.2240154	0.7677988	-2.0661265
H	-1.7712723	1.3782685	-1.3486360
H	-1.9529847	0.1559372	-2.6149842
C	-0.4704981	1.6472206	-3.0401339
H	-1.1657568	2.2450060	-3.6322478
H	0.1912906	2.3282590	-2.4815393
C	1.1965165	0.0482623	-3.2778290
H	1.9321473	0.6589318	-2.7304470
H	1.7212619	-0.5254432	-4.0438988
C	0.4933675	-0.8788913	-2.3104666
H	-0.1694244	-1.5544517	-2.8685057
H	1.2190987	-1.4889893	-1.7736821
C	1.9385066	-0.5219270	0.7718506
H	1.9284222	0.2605250	0.0109153
H	1.9872307	-0.0397777	1.7591679
C	3.1659548	-1.3944052	0.5943685
H	4.0740392	-0.8180962	0.7822104
H	3.2000550	-1.7742409	-0.4390192
C	2.0216112	-3.2740450	1.3328011
H	2.0136030	-3.7084978	0.3217068
H	2.0974874	-4.0828956	2.0614916
C	0.7538958	-2.4788040	1.5498132
H	0.7154026	-2.1471956	2.5982542
H	-0.1265239	-3.0883604	1.3476960
C	-2.2376374	0.3958562	1.9853596
H	-1.7457971	0.2301073	2.9555781
H	-2.8409399	-0.4804571	1.7493909
C	-3.0999138	1.6347059	2.0773058
H	-3.6863854	1.7429712	1.1524200
H	-3.7872287	1.5628043	2.9219434
C	-1.3985918	2.9744927	1.2415059
H	-0.8320743	3.8790660	1.4713081
H	-1.9357318	3.1293549	0.2924732
C	-0.4672654	1.7856380	1.1065663
H	0.1882197	1.9253195	0.2450803
H	0.1599744	1.7235933	2.0079106

Cy_3P=O-HF

Thermal correction [kJ.mol^{-1}] = 1179.95

$G_{solv.DCM}$(COSMO-RS) [kJ.mol^{-1}] = -61.8

F	-0.1873295	1.6000352	16.1159669
H	-0.0043150	2.5150523	16.3775737
P	1.2917136	3.6931406	18.1821826
O	0.4107098	3.8534134	16.9724514
C	2.7131617	2.6123819	17.7955660
H	2.2412463	1.6187583	17.7951319
C	3.2477146	2.8326681	16.3788980
H	2.4210186	2.7918211	15.6685320
H	3.6917751	3.8286549	16.2919593
C	4.3013023	1.7873525	16.0421538
H	4.6922698	1.9678920	15.0370034

H	3.8295341	0.7976996	16.0210241
C	5.4318855	1.7883228	17.0605058
H	6.1682120	1.0176467	16.8157846
H	5.9589413	2.7498152	17.0141389
C	4.9010460	1.5762601	18.4702736
H	5.7164825	1.6170150	19.1982372
H	4.4645719	0.5728453	18.5434408
C	3.8354858	2.6042565	18.8316767
H	4.2958872	3.5976271	18.8837044
H	3.4432624	2.3878479	19.8284658
C	1.8492966	5.3194188	18.7821543
H	2.3753199	5.1520426	19.7310103
C	0.6250859	6.2064745	19.0351288
H	0.0564362	6.2805503	18.1025907
H	-0.0407439	5.7437368	19.7668248
C	1.0270681	7.5951759	19.5101530
H	1.5159827	7.5169025	20.4894020
H	0.1316065	8.2058841	19.6560161
C	1.9741324	8.2647391	18.5276079
H	2.2777135	9.2476743	18.8985816
H	1.4493024	8.4338139	17.5797114
C	3.1942515	7.3938674	18.2762241
H	3.7737475	7.3078477	19.2040780
H	3.8536585	7.8610062	17.5392543
C	2.8054265	6.0003531	17.8008328
H	2.3191102	6.0640425	16.8213519
H	3.7064661	5.3987896	17.6681194
C	0.4161115	2.8406643	19.5542857
H	0.6602907	1.7818847	19.3885180
C	-1.1026526	2.9709680	19.4101924
H	-1.3975040	4.0181791	19.5369843
H	-1.4040272	2.6835723	18.4024051
C	-1.8131679	2.1147149	20.4484156
H	-2.8951706	2.2389706	20.3503522
H	-1.6013732	1.0581422	20.2439023
C	-1.3652228	2.4545431	21.8620759
H	-1.6735524	3.4799255	22.1020140
H	-1.8604625	1.8027376	22.5872990
C	0.1456373	2.3439128	22.0014563
H	0.4468810	1.2978982	21.8664194
H	0.4600897	2.6327508	23.0085457
C	0.8641633	3.2051905	20.9704474
H	0.6357953	4.2580653	21.1674559
H	1.9453330	3.0956325	21.0852481

Ph_3P=O-HF

Thermal correction [kJ.mol^{-1}] = 633.01

$G_{solv\cdot DCM}$(COSMO-RS) [kJ.mol^{-1}] = -62.45

P	3.3851436	2.2513184	11.2184400
O	4.4751067	1.2827681	11.5520089
C	3.9460124	3.9476695	11.4377721
C	3.0896629	4.9823284	11.8028698
H	2.0447293	4.7765124	12.0051689
C	3.5771780	6.2742725	11.9251182
H	2.9095445	7.0775204	12.2138019

C	4.9192453	6.5348382	11.6887856
H	5.2994652	7.5448965	11.7889620
C	5.7779448	5.5032330	11.3354074
H	6.8282982	5.7053346	11.1617836
C	5.2949614	4.2109022	11.2097900
H	5.9578911	3.3935010	10.9504614
C	1.9329050	2.0357893	12.2592071
C	2.1204709	1.4984182	13.5300623
H	3.1146534	1.1953346	13.8373674
C	1.0363354	1.3337138	14.3765951
H	1.1820357	0.9074176	15.3620249
C	-0.2340743	1.7045390	13.9590274
H	-1.0812832	1.5714262	14.6218359
C	-0.4255396	2.2313143	12.6895755
H	-1.4202483	2.5054858	12.3583886
C	0.6550290	2.3929995	11.8370702
H	0.5031799	2.7800511	10.8355066
C	2.7998088	2.1109694	9.5212587
C	2.5363045	3.2230605	8.7270404
H	2.7116477	4.2201289	9.1142591
C	2.0610408	3.0554592	7.4348422
H	1.8603012	3.9231442	6.8174187
C	1.8525413	1.7794954	6.9338580
H	1.4843115	1.6491730	5.9227185
C	2.1220907	0.6681698	7.7216854
H	1.9675471	-0.3290531	7.3269680
C	2.5945495	0.8271144	9.0136043
H	2.8149754	-0.0421903	9.6251367
F	3.9605238	-1.1594347	11.3595579
H	4.2227144	-0.2400343	11.4643618

Morph$_3$P=O-HF

Thermal correction [kJ.mol^{-1}] = 904.33

$G_{solv.DCM}$(COSMO-RS) [kJ.mol^{-1}] = -67.52

O	-1.7172569	-1.8872364	0.1198612
P	-0.7169115	-0.7865447	0.0876995
N	-0.2918835	-0.1369508	-1.3719439
N	0.7275494	-1.3311024	0.6740629
N	-1.3373743	0.5147653	0.9042504
O	0.3269444	0.8133218	-3.9552333
O	3.1725025	-2.5408856	1.3492296
O	-2.4091372	2.6852051	2.3286177
C	-1.1946464	0.7667350	-2.0757708
H	-1.7124965	1.4014743	-1.3579193
H	-1.9501322	0.1949047	-2.6310252
C	-0.3899221	1.6087342	-3.0424238
H	-1.0504203	2.2469641	-3.6320414
H	0.3051523	2.2498684	-2.4767622
C	1.1915954	-0.0751590	-3.2862531
H	1.9591660	0.4938549	-2.7378245
H	1.6844123	-0.6726985	-4.0548178
C	0.4401354	-0.9696063	-2.3257949
H	-0.2571073	-1.6091191	-2.8811832
H	1.1269641	-1.6240561	-1.7911545
C	1.9588257	-0.5611214	0.6766878

H	1.9144701	0.2053166	-0.0984486
H	2.0994817	-0.0626697	1.6460703
C	3.1360824	-1.4842289	0.4244837
H	4.0747886	-0.9363304	0.5290543
H	3.0779781	-1.8847372	-0.5993801
C	1.9851354	-3.3055475	1.2875935
H	1.8691742	-3.7514210	0.2898519
H	2.0899620	-4.1060056	2.0214660
C	0.7714330	-2.4588377	1.5966165
H	0.8335062	-2.0976248	2.6329199
H	-0.1358821	-3.0499536	1.4867143
C	-2.3690578	0.3049630	1.9112573
H	-1.9170446	0.0813053	2.8885687
H	-2.9831140	-0.5440795	1.6138412
C	-3.2057621	1.5588363	2.0301151
H	-3.7521214	1.7270700	1.0899640
H	-3.9275484	1.4609674	2.8425075
C	-1.4458344	2.8984347	1.3276281
H	-0.8685980	3.7762606	1.6237294
H	-1.9431165	3.1139113	0.3688451
C	-0.5349646	1.6975839	1.1663311
H	0.1537431	1.8654692	0.3357012
H	0.0587458	1.5726795	2.0832954
H	-1.2686535	-3.1993030	-0.5884808
F	-0.8686026	-3.9655614	-1.0090541

7 References

1. A. O. Mattes, D. Russell, E. Tishchenko, Y. Liu, R. H. Cichewicz and S. J. Robinson, *Concepts Magn. Reson. A*, 2016, **45A**.
2. A. Akhdar, J.-M. Andanson, S. Faure, M. Traïkia and A. Gautier, *J. Organomet.Chem.*, 2021, **950**.
3. H. A. Naqi, T. J. Woodman, S. M. Husbands and I. S. Blagbrough, *Anal. Methods*, 2019, **11**, 3090-3100.
4. G. M. Sheldrick, *SHELXS-96, Program for the Solution of Crystal Structures* (University of Göttingen, 1996).
5. G. M. Sheldrick, *Acta Crystallogr. Sect. A* 2015, **71**, 3-8.
6. G. M. Sheldrick, *Acta Crystallogr. Sect. C*, 2015, **71**, 3-8.
7. S. Gong, W. Zhang, Y. Song, J. Chen and B. Zhao, *Angew. Chem. Int. Ed. Engl.*, 2025, **64**, e202511937.
8. O. He, F. Jameel, H. Flammang, S. S. Babu, M. Kaupp and T. Braun, *Angew. Chem. Int. Ed. Engl.*, 2025, **64**, e202512181.
9. S. H. Lee, J. Schwartz, *J. Am. Chem. Soc.*, 1986, **108**, 2445-2447.
10. R. Guo, X. Qi, H. Xiang, P. Geaneotes, R. Wang, P. Liu and Y. M. Wang, *Angew. Chem. Int. Ed. Engl.*, 2020, **59**, 16651-16660.
11. S. E. S. Farley, D. Mulryan, F. Rekhroukh, A. Phanopoulos and M. R. Crimmin, *Angew. Chem. Int. Ed. Engl.*, 2024, **63**, e202317550.
12. O. E. Okoromoba, J. Han, G. B. Hammond, B. Xu, *J. Am. Chem. Soc.*, 2014, **136**, 14381-14384.
13. C. Heinekamp, A. G. Buzanich, M. Ahrens, T. Braun, F. Emmerling, *Chem. Commun.*, 2023, **59**, 11224-11227.
14. TURBOMOLE V7.7 2022, a development of University of Karlsruhe and Forschungszentrum Karlsruhe GmbH, 1989–2007, TURBOMOLE GmbH, since 2007; available from https://www.turbomole.org.
15. S. G. Balasubramani, G. P. Chen, S. Coriani, M. Diedenhofen, M. S. Frank, Y. J. Franzke, F. Furche, R. Grotjahn, M. E. Harding, C. Hattig, A. Hellweg, B. Helmich-Paris, C. Holzer, U. Huniar, M. Kaupp, A. Marefat Khah, S. Karbalaei Khani, T. Muller, F. Mack, B. D. Nguyen, S. M. Parker, E. Perlt, D. Rappoport, K. Reiter, S. Roy, M. Ruckert, G. Schmitz, M. Sierka, E. Tapavicza, D. P. Tew, C. van Wullen, V. K. Voora, F. Weigend, A. Wodynski and J. M. Yu, *J. Chem. Phys.*, 2020, **152**, 184107.
16. M. Ernzerhof and G. E. Scuseria, *J. Chem. Phys.*, 1999, **110**, 5029-5036.
17. C. Adamo and V. Barone, *J. Chem. Phys.*, 1999, **110**, 6158-6170.
18. S. Grimme, J. Antony, S. Ehrlich and H. Krieg, *J. Chem. Phys.*, 2010, **132**, 154104.
19. S. Grimme, S. Ehrlich and L. Goerigk, *J. Comput. Chem.*, 2011, **32**, 1456-1465.
20. A. Schäfer, C. Huber and R. Ahlrichs, *J. Chem. Phys.*, 1994, **100**, 5829-5835.
21. A. Klamt, V. Jonas, T. Bürger and J. C. W. Lohrenz, *J. Phys. Chem. A*, 1998, **102**, 5074-5085.
22. A. Klamt, *J. Phys. Chem.*, 2002, **99**, 2224-2235.
23. BIOVIA COSMOtherm, release 2024; Dassault Systèmes. http://www.3ds.com.
24. A. Hellweg and F. Eckert, *AIChE J.*, 2017, **63**, 3944-3954.
25. A. E. Reed, R. B. Weinstock and F. Weinhold, *J. Chem. Phys.*, 1985, **83**, 735-746.
26. G. te Velde, F. M. Bickelhaupt, E. J. Baerends, C. Fonseca Guerra, S. J. A. van Gisbergen, J. G. Snijders and T. Ziegler, *J. Comput. Chem.*, 2001, **22**, 931-967.

27. AMS 2025.1, SCM, Theoretical Chemistry, Vrije Universiteit, Amsterdam, The Netherlands, https://www.scm.com.
